# Supplementary material for: A novel chloroplast super-complex consisting of the ATP synthase and photosystem I reaction center
Source: PLoS One. 2020 Aug 20;15(8):e0237569. doi: 10.1371/journal.pone.0237569 (PMC7444523; doi:10.1371/journal.pone.0237569)
Supplement: S1 File — (PDF) [file pone.0237569.s001.pdf]

MASCOT Search Results

Protein View: sp|P00825|ATPB_SPIOL

>sp|P00825|ATPB_SPIOL ATP synthase subunit beta, chloroplastic OS=Spinacia oleracea OX=3562 GN=atpB PE=1 SV=2

**Database:**	Uni-Spinach
**Score:**	2844
**Nominal mass (M_r):**	53768
**Calculated pI:**	5.22

Sequence similarity is available as **an NCBI BLAST search of sp|P00825|ATPB_SPIOL against nr**.

Search parameters

**MS data file:**	\\128.97.66.218\tank\windowsVM\Bill Cramer\MGF\wc_QE_021417_Cramer_Gel_1S_4a.mgf
**Enzyme:**	Trypsin/P: cuts C-term side of KR.
**Fixed modifications:**	Carbamidomethyl (C)
**Variable modifications:**	Oxidation (M)

Protein sequence coverage: 80%

Matched peptides shown in ***bold red***.

```
  1 MRINPTTSDP GVSTLEKKNL GRIAQIIGPV LDVAFPPGKM PNIYNALIVK
 51 GRDTAGQPMN VTCEVQQLLG NNRVRAVAMS ATDGLTRGME VIDTGAPLSV
101 PVGGATLGRI FNVLGEPVDN LGPVDTRTTS PIHRSAPAFT QLDTKLSIFE
151 TGIKVVDLLA PYRRGGKIGL FGGAGVGKTV LIMELINNIA KAHGGVSVFG
201 GVGERTREGN DLYMEMKESG VINEQNIAES KVALVYGQMN EPPGARMRVG
251 LTALTMAEYF RDVNEQDVLL FIDNIFRFVQ AGSEVSALLG RMPSAVGYQP
301 TLSTEMGSLQ ERITSTKEGS ITSIQAVYVP ADDLTDPAPA TTFAHLDATT
351 VLSRGLAAKG IYPAVDPLDS TSTMLQPRIV GEEHYEIAQR VKETLQRYKE
401 LQDIIAILGL DELSEEDRLT VARARKIERF LSQPFFVAEV FTGSPGKYVG
451 LAETIRGFQL ILSGELDSLP EQAFYLVGNI DEATAKAMNL EMESKLKK
```

Unformatted sequence string: **498 residues** (for pasting into other applications).

Sort peptides by ◉ Residue Number ○ Increasing Mass ○ Decreasing Mass

Show predicted peptides also

Query	Start – End	Observed	Mr(expt)	Mr(calc)	ppm	M	Score	Expect	Rank	U	Peptide
19674	3 – 17	779.9003	1557.7860	1557.7886	-1.65	0	25	0.0044	1	U	R.INPTTSDPGVSTLEK.K
19676	3 – 17	779.9009	1557.7872	1557.7886	-0.88	0	77	5.8e-08	1	U	R.INPTTSDPGVSTLEK.K
19677	3 – 17	779.9010	1557.7874	1557.7886	-0.75	0	2	0.62	1	U	R.INPTTSDPGVSTLEK.K
19678	3 – 17	520.2698	1557.7875	1557.7886	-0.69	0	38	0.00025	1	U	R.INPTTSDPGVSTLEK.K
21655	3 – 18	562.9681	1685.8824	1685.8836	-0.68	1	12	0.081	1	U	R.INPTTSDPGVSTLEKK.N
22326	23 – 39	579.0096	1734.0070	1734.0080	-0.57	0	46	5.6e-05	1	U	R.IAQIIGPVLDVAFPPGK.M
22327	23 – 39	868.0117	1734.0088	1734.0080	0.45	0	40	0.00019	1	U	R.IAQIIGPVLDVAFPPGK.M
22328	23 – 39	579.0106	1734.0100	1734.0080	1.14	0	37	0.00037	1	U	R.IAQIIGPVLDVAFPPGK.M
22329	23 – 39	868.0124	1734.0103	1734.0080	1.32	0	50	2e-05	1	U	R.IAQIIGPVLDVAFPPGK.M
13286	40 – 50	638.3597	1274.7048	1274.7056	-0.65	0	11	0.093	1	U	K.MPNIYNALIVK.G
13287	40 – 50	638.3609	1274.7073	1274.7056	1.33	0	9	0.14	1	U	K.MPNIYNALIVK.G
13666	40 – 50	646.3570	1290.6994	1290.7006	-0.92	0	22	0.0083	1	U	K.MPNIYNALIVK.G + Oxidation (M)
13667	40 – 50	646.3582	1290.7019	1290.7006	1.01	0	61	2.1e-06	1	U	K.MPNIYNALIVK.G + Oxidation (M)
28203	51 – 73	853.4109	2557.2110	2557.2126	-0.60	1	38	0.00025	1	U	K.GRDTAGQPMNVTCEVQQLLGNNR.V
28281	51 – 73	858.7432	2573.2079	2573.2075	0.16	1	44	7.1e-05	1	U	K.GRDTAGQPMNVTCEVQQLLGNNR.V + Oxidation (M)
27322	53 – 73	1173.0495	2344.0844	2344.0900	-2.36	0	81	2.3e-08	1	U	R.DTAGQPMNVTCEVQQLLGNNR.V
27323	53 – 73	782.3718	2344.0935	2344.0900	1.48	0	104	1.7e-10	1	U	R.DTAGQPMNVTCEVQQLLGNNR.V
10979	76 – 87	596.8029	1191.5913	1191.5918	-0.42	0	49	2.8e-05	1	U	R.AVAMSATDGLTR.G
10980	76 – 87	596.8030	1191.5914	1191.5918	-0.28	0	66	6.6e-07	1	U	R.AVAMSATDGLTR.G
10981	76 – 87	596.8034	1191.5923	1191.5918	0.48	0	7	0.2	1	U	R.AVAMSATDGLTR.G
10982	76 – 87	596.8040	1191.5934	1191.5918	1.41	0	23	0.0068	1	U	R.AVAMSATDGLTR.G
11442	76 – 87	604.8002	1207.5858	1207.5867	-0.69	0	5	0.37	1	U	R.AVAMSATDGLTR.G + Oxidation (M)
11443	76 – 87	604.8003	1207.5861	1207.5867	-0.49	0	55	6.9e-06	1	U	R.AVAMSATDGLTR.G + Oxidation (M)
11444	76 – 87	604.8007	1207.5869	1207.5867	0.18	0	53	1e-05	1	U	R.AVAMSATDGLTR.G + Oxidation (M)
11445	76 – 87	604.8015	1207.5885	1207.5867	1.53	0	36	0.00041	1	U	R.AVAMSATDGLTR.G + Oxidation (M)
25805	88 – 109	1049.0554	2096.0963	2096.0936	1.29	0	81	2.4e-08	1	U	R.GMEVIDTGAPLSVPVGGATLGR.I
25807	88 – 109	699.7061	2096.0966	2096.0936	1.42	0	36	0.00042	1	U	R.GMEVIDTGAPLSVPVGGATLGR.I
25909	88 – 109	1057.0517	2112.0889	2112.0885	0.19	0	75	8.8e-08	1	U	R.GMEVIDTGAPLSVPVGGATLGR.I + Oxidation (M)
25910	88 – 109	705.0370	2112.0892	2112.0885	0.32	0	42	0.00011	1	U	R.GMEVIDTGAPLSVPVGGATLGR.I + Oxidation (M)
25911	88 – 109	1057.0520	2112.0895	2112.0885	0.45	0	75	8.4e-08	1	U	R.GMEVIDTGAPLSVPVGGATLGR.I + Oxidation (M)
25912	88 – 109	705.0374	2112.0903	2112.0885	0.86	0	32	0.00098	1	U	R.GMEVIDTGAPLSVPVGGATLGR.I + Oxidation (M)
24712	110 – 127	978.0154	1954.0162	1954.0160	0.11	0	119	7.4e-12		U	R.IFNVLGEPVDNLGPVDTR.T

1

24713	110 – 127	652.3468	1954.0185	1954.0160	1.27	0	36	0.00039	1	U	R.IFNVLGEPVDNLGPVDTR.T
1870	128 – 134	406.2241	810.4337	810.4348	-1.42	0	30	0.0016	1	U	R.TTSPIHR.S
10555	135 – 145	589.8062	1177.5979	1177.5979	-0.025	0	62	1.6e-06	1	U	R.SAPAFTQLDTK.L
10556	135 – 145	589.8065	1177.5984	1177.5979	0.40	0	4	0.47	1	U	R.SAPAFTQLDTK.L
6059	146 – 154	504.2925	1006.5705	1006.5699	0.61	0	0	3.9	1	U	K.LSIFETGIK.V
6060	146 – 154	504.2926	1006.5706	1006.5699	0.70	0	22	0.026	1	U	K.LSIFETGIK.V
6857	155 – 163	523.3055	1044.5965	1044.5968	-0.23	0	44	0.0004	1	U	K.VVDLLAPYR.R
6858	155 – 163	523.3056	1044.5966	1044.5968	-0.13	0	41	0.00076	1	U	K.VVDLLAPYR.R
6859	155 – 163	523.3058	1044.5971	1044.5968	0.29	0	40	0.00074	1	U	K.VVDLLAPYR.R
11247	155 – 164	401.2395	1200.6967	1200.6979	-1.01	1	16	0.029	1	U	K.VVDLLAPYRR.G
11248	155 – 164	401.2402	1200.6987	1200.6979	0.72	1	7	0.22	1	U	K.VVDLLAPYRR.G
11250	155 – 164	601.3569	1200.6992	1200.6979	1.11	1	13	0.067	1	U	K.VVDLLAPYRR.G
5423	168 – 178	488.2842	974.5539	974.5549	-1.04	0	20	0.014	1		K.IGLFGGAGVGK.T
5424	168 – 178	488.2847	974.5549	974.5549	-0.064	0	3	0.53	1		K.IGLFGGAGVGK.T
17960	179 – 191	736.4309	1470.8473	1470.8479	-0.43	0	1	4.2	1	U	K.TVLIMELINNIAK.A
18301	179 – 191	496.6221	1486.8445	1486.8429	1.08	0	22	0.028	1	U	K.TVLIMELINNIAK.A + Oxidation (M)
18302	179 – 191	744.4298	1486.8451	1486.8429	1.50	0	64	1.7e-06	1	U	K.TVLIMELINNIAK.A + Oxidation (M)
14536	192 – 205	664.8390	1327.6634	1327.6633	0.021	0	26	0.0037	1	U	K.AHGGVSVFGGVGER.T
14537	192 – 205	443.5625	1327.6658	1327.6633	1.84	0	32	0.0011	1	U	K.AHGGVSVFGGVGER.T
14539	192 – 205	443.5627	1327.6661	1327.6633	2.12	0	52	1.4e-05	1	U	K.AHGGVSVFGGVGER.T
14540	192 – 205	664.8404	1327.6662	1327.6633	2.16	0	35	0.00052	1	U	K.AHGGVSVFGGVGER.T
18250	206 – 217	496.2259	1485.6559	1485.6592	-2.20	1	14	0.13	1	U	R.TREGNDLYMEMK.E
18251	206 – 217	496.2261	1485.6565	1485.6592	-1.81	1	11	0.28	1	U	R.TREGNDLYMEMK.E
18599	206 – 217	751.8334	1501.6522	1501.6541	-1.22	1	10	0.23	1	U	R.TREGNDLYMEMK.E + Oxidation (M)
18600	206 – 217	751.8334	1501.6522	1501.6541	-1.22	1	21	0.018	1	U	R.TREGNDLYMEMK.E + Oxidation (M)
18601	206 – 217	501.5580	1501.6523	1501.6541	-1.17	1	8	0.38	1	U	R.TREGNDLYMEMK.E + Oxidation (M)
18602	206 – 217	501.5583	1501.6531	1501.6541	-0.67	1	14	0.098	1	U	R.TREGNDLYMEMK.E + Oxidation (M)
18902	206 – 217	759.8315	1517.6485	1517.6490	-0.31	1	21	0.011	1	U	R.TREGNDLYMEMK.E + 2 Oxidation (M)
18904	206 – 217	506.8909	1517.6508	1517.6490	1.20	1	11	0.13	1	U	R.TREGNDLYMEMK.E + 2 Oxidation (M)
12478	208 – 217	623.2598	1244.5051	1244.5053	-0.13	0	5	0.31	1	U	R.EGNDLYMEMK.E + Oxidation (M)
12480	208 – 217	623.2609	1244.5072	1244.5053	1.57	0	7	0.19	1	U	R.EGNDLYMEMK.E + Oxidation (M)
18892	218 – 231	759.3757	1516.7368	1516.7369	-0.064	0	62	1.5e-06	1	U	K.ESGVINEQNIAESK.V
18893	218 – 231	506.5863	1516.7371	1516.7369	0.17	0	6	0.28	1	U	K.ESGVINEQNIAESK.V
18894	218 – 231	759.3761	1516.7376	1516.7369	0.47	0	33	0.00079	1	U	K.ESGVINEQNIAESK.V
20376	232 – 246	801.4065	1600.7984	1600.8031	-2.98	0	26	0.0033	1	U	K.VALVYGQMNEPPGAR.M
20381	232 – 246	801.4083	1600.8021	1600.8031	-0.67	0	67	4.8e-07	1	U	K.VALVYGQMNEPPGAR.M
20383	232 – 246	801.4091	1600.8036	1600.8031	0.30	0	71	2.1e-07	1	U	K.VALVYGQMNEPPGAR.M
20384	232 – 246	801.4095	1600.8045	1600.8031	0.82	0	59	2.8e-06	1	U	K.VALVYGQMNEPPGAR.M
20385	232 – 246	801.4096	1600.8046	1600.8031	0.90	0	71	2.2e-07	1	U	K.VALVYGQMNEPPGAR.M
20386	232 – 246	534.6089	1600.8049	1600.8031	1.08	0	11	0.1	1	U	K.VALVYGQMNEPPGAR.M
20387	232 – 246	534.6090	1600.8050	1600.8031	1.17	0	12	0.069	1	U	K.VALVYGQMNEPPGAR.M
20389	232 – 246	534.6092	1600.8058	1600.8031	1.65	0	12	0.074	1	U	K.VALVYGQMNEPPGAR.M
20390	232 – 246	801.4102	1600.8058	1600.8031	1.68	0	48	3.4e-05	1	U	K.VALVYGQMNEPPGAR.M
20391	232 – 246	801.4109	1600.8073	1600.8031	2.61	0	43	0.0001	1	U	K.VALVYGQMNEPPGAR.M
20393	232 – 246	801.4114	1600.8082	1600.8031	3.19	0	20	0.012	1	U	K.VALVYGQMNEPPGAR.M
20603	232 – 246	809.4053	1616.7959	1616.7981	-1.31	0	29	0.0029	1	U	K.VALVYGQMNEPPGAR.M + Oxidation (M)
20604	232 – 246	809.4054	1616.7962	1616.7981	-1.12	0	21	0.015	1	U	K.VALVYGQMNEPPGAR.M + Oxidation (M)
20605	232 – 246	809.4063	1616.7981	1616.7981	0.048	0	29	0.002	1	U	K.VALVYGQMNEPPGAR.M + Oxidation (M)
20606	232 – 246	539.9401	1616.7985	1616.7981	0.24	0	18	0.022	1	U	K.VALVYGQMNEPPGAR.M + Oxidation (M)
20608	232 – 246	809.4068	1616.7991	1616.7981	0.66	0	19	0.027	1	U	K.VALVYGQMNEPPGAR.M + Oxidation (M)
20609	232 – 246	539.9405	1616.7997	1616.7981	1.03	0	7	0.22	1	U	K.VALVYGQMNEPPGAR.M + Oxidation (M)
20610	232 – 246	539.9406	1616.7999	1616.7981	1.16	0	5	0.32	1	U	K.VALVYGQMNEPPGAR.M + Oxidation (M)
20611	232 – 246	809.4076	1616.8007	1616.7981	1.62	0	31	0.0011	1	U	K.VALVYGQMNEPPGAR.M + Oxidation (M)
17955	249 – 261	736.3845	1470.7544	1470.7541	0.21	0	48	2.9e-05	1	U	R.VGLTALTMAEYFR.D
18294	249 – 261	744.3816	1486.7487	1486.7490	-0.17	0	55	6.7e-06	1	U	R.VGLTALTMAEYFR.D + Oxidation (M)
17087	278 – 291	717.3901	1432.7656	1432.7674	-1.24	0	17	0.028	1	U	R.FVQAGSEVSALLGR.M
17088	278 – 291	717.3911	1432.7677	1432.7674	0.21	0	89	4.3e-09	1	U	R.FVQAGSEVSALLGR.M
17089	278 – 291	478.5971	1432.7694	1432.7674	1.35	0	49	2.8e-05	1	U	R.FVQAGSEVSALLGR.M
26944	292 – 312	761.3649	2281.0729	2281.0719	0.46	0	74	1.2e-07	1	U	R.MPSAVGYQPTLSTEMGSLQER.I
26945	292 – 312	1141.5446	2281.0746	2281.0719	1.19	0	85	9.8e-09	1	U	R.MPSAVGYQPTLSTEMGSLQER.I
26946	292 – 312	761.3658	2281.0756	2281.0719	1.64	0	74	1.1e-07	1	U	R.MPSAVGYQPTLSTEMGSLQER.I
27043	292 – 312	766.6957	2297.0653	2297.0668	-0.62	0	28	0.0025	1	U	R.MPSAVGYQPTLSTEMGSLQER.I + Oxidation (M)
27044	292 – 312	766.6966	2297.0678	2297.0668	0.46	0	75	9.5e-08	1	U	R.MPSAVGYQPTLSTEMGSLQER.I + Oxidation (M)
27045	292 – 312	1149.5419	2297.0692	2297.0668	1.07	0	99	5.7e-10	1	U	R.MPSAVGYQPTLSTEMGSLQER.I + Oxidation (M)
27046	292 – 312	766.6992	2297.0757	2297.0668	3.89	0	89	4.1e-09	1	U	R.MPSAVGYQPTLSTEMGSLQER.I + Oxidation (M)
27146	292 – 312	1157.5371	2313.0597	2313.0617	-0.87	0	42	0.00013	1	U	R.MPSAVGYQPTLSTEMGSLQER.I + 2 Oxidation (M)
27147	292 – 312	772.0278	2313.0616	2313.0617	-0.022	0	87	6.5e-09	1	U	R.MPSAVGYQPTLSTEMGSLQER.I + 2 Oxidation (M)
29980	318 – 354	1281.9831	3842.9276	3842.9214	1.60	0	61	2e-06	1	U	K.EGSITSIQAVYVPADDLTDPAPATTFAHLDATTVLSR.G
29981	318 – 354	961.7396	3842.9295	3842.9214	2.08	0	51	1.6e-05	1	U	K.EGSITSIQAVYVPADDLTDPAPATTFAHLDATTVLSR.G
25553	360 – 378	687.6824	2060.0253	2060.0248	0.21	0	43	8.6e-05	1	U	K.GIYPAVDPLDSTSTMLQPR.I
25554	360 – 378	1031.0218	2060.0290	2060.0248	2.03	0	87	1.9e-08	1	U	K.GIYPAVDPLDSTSTMLQPR.I
25654	360 – 378	1039.0162	2076.0179	2076.0198	-0.87	0	69	3.3e-07	1	U	K.GIYPAVDPLDSTSTMLQPR.I + Oxidation (M)
25655	360 – 378	693.0139	2076.0199	2076.0198	0.071	0	48	3.5e-05	1	U	K.GIYPAVDPLDSTSTMLQPR.I + Oxidation (M)
25656	360 – 378	1039.0217	2076.0288	2076.0198	4.33	0	37	0.00036	1	U	K.GIYPAVDPLDSTSTMLQPR.I + Oxidation (M)
17308	379 – 390	722.3638	1442.7130	1442.7154	-1.63	0	53	1e-05	1	U	R.IVGEEHYEIAQR.V
17310	379 – 390	481.9120	1442.7142	1442.7154	-0.80	0	32	0.00098	1	U	R.IVGEEHYEIAQR.V

17311	379 - 390	481.9132	1442.7178	1442.7154	1.68	0	32	0.0011	1	U	R.IVGEEHYEIAQR.V
17312	379 - 390	722.3663	1442.7181	1442.7154	1.87	0	40	0.00018	1	U	R.IVGEEHYEIAQR.V
28836	400 - 423	904.4896	2710.4469	2710.4388	2.97	1	43	0.00025	1	U	K.ELQDIIAILGLDELSEEDRLTVAR.A
24770	430 - 447	979.5073	1957.0001	1956.9986	0.78	0	67	5.7e-07	1	U	R.FLSQPFFVAEVFTGSPGK.Y
7013	487 - 495	526.7414	1051.4683	1051.4678	0.52	0	20	0.044	1	U	K.AMNLEMESK.L
7014	487 - 495	526.7415	1051.4685	1051.4678	0.74	0	13	0.059	1	U	K.AMNLEMESK.L
7442	487 - 495	534.7384	1067.4623	1067.4627	-0.41	0	7	0.55	1	U	K.AMNLEMESK.L + Oxidation (M)
7443	487 - 495	534.7385	1067.4624	1067.4627	-0.30	0	24	0.012	1	U	K.AMNLEMESK.L + Oxidation (M)

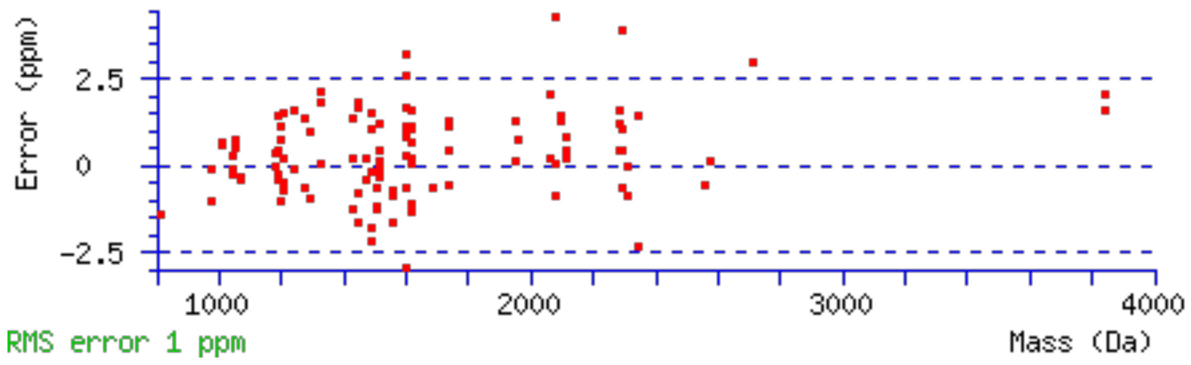

Mascot: http://www.matrixscience.com/

MATRIX SCIENCE MASCOT Search Results

Protein View: sp|P06512|PSAB_SPIOL

>sp|P06512|PSAB_SPIOL Photosystem I P700 chlorophyll a apoprotein A2 OS=Spinacia oleracea OX=3562 GN=psaB PE=3 SV=1

**Database:**	Uni-Spinach
**Score:**	2055
**Nominal mass (M_r):**	82491
**Calculated pI:**	6.72

Sequence similarity is available as **an NCBI BLAST search of sp|P06512|PSAB_SPIOL against nr**.

Search parameters

**MS data file:**	\\128.97.66.218\tank\windowsVM\Bill Cramer\MGF\wc_QE_021417_Cramer_Gel_1S_4a.mgf
**Enzyme:**	Trypsin/P: cuts C-term side of KR.
**Fixed modifications:**	**Carbamidomethyl (C)**
**Variable modifications:**	**Oxidation (M)**

Protein sequence coverage: 29%

Matched peptides shown in ***bold red***.

1	MALRFPR**FSQ**	**GLAQDPTTRR**	**IWFGIATAHD**	**FESHDDITEE**	**R**LYQNIFASH
51	FGQLAIIFLW	TSGNLFHVAW	QGNFESWVQD	PLHVR**PIAHA**	**IWDPHFGQPA**
101	**VEAFTR**GGAL	GPVNIAYSGV	YQWWYTIGLR	TNEDLYTGAL	FLLFLSVISL
151	LGGWLHLQPK	**WKPSVSWFKN**	**AESRLNHHLS**	**GLFGVSSLAW**	**TGHLVHVAIP**
201	**GSR**GEYVRWN	NFLDVLPHPQ	GLGPLFTGQW	NLYAQNPDSS	SHLFGTSQGA
251	GTAILTLLGG	FHPQTQSLWL	TDMAHHHLAI	AFVFLVAGHM	YR**TNFGIGHS**
301	**MKDLLEAHIP**	**PGGR**LGRGHK	GLYDTINNSL	HFQLGLALAS	LGVITSLVAQ
351	HMYSLPAYAF	IAQDFTTQAA	LYTHHQYIAG	FIMTGAFAHG	AIFFIR**DYNP**
401	**EQNEDNVLAR**	MLDHKEAIIS	HLSWASLFLG	FHTLGLYVHN	DVMLAFGTPE
451	K**QILIEPIFA**	**QWIQSAHGKT**	**SYGFDVLLSS**	**TSGPAFNAGR**	SIWLPGWLNA
501	VNENSNSLFL	TIGPGDFLVH	HAIALGLHTT	TLILVKGALD	ARGSK**LMPDK**
551	**KDFGYSFPCD**	**GPGR**GGTCDI	SAWDAFYLAV	FWMLNTIGWV	TFYWHWKHIT
601	LWQGNVSQFN	ESSTYLMGWL	RDYLWLNSSQ	LINGYNPFGM	NSLSVWAWMF
651	LFGHLVWATG	FMFLISWRGY	WQELIETLAW	AHER**TPLANL**	**IRWRDKPVAL**
701	**SIVQAR**LVGL	AHFSVGYIFT	YAAFLIASTS	GKFG	

Unformatted sequence string: **734 residues** (for pasting into other applications).

Sort peptides by ◉ Residue Number ○ Increasing Mass ○ Decreasing Mass

Show predicted peptides also

Query	Start – End	Observed	Mr(expt)	Mr(calc)	ppm	M	Score	Expect	Rank	U	Peptide
14391	8 – 19	660.8292	1319.6438	1319.6470	-2.41	0	19	0.018	1	U	R.FSQGLAQDPTTR.R
14392	8 – 19	660.8307	1319.6468	1319.6470	-0.12	0	38	0.00029	1	U	R.FSQGLAQDPTTR.R
14395	8 – 19	660.8308	1319.6471	1319.6470	0.099	0	7	0.22	1	U	R.FSQGLAQDPTTR.R
14396	8 – 19	660.8313	1319.6480	1319.6470	0.73	0	34	0.00072	1	U	R.FSQGLAQDPTTR.R
14397	8 – 19	660.8323	1319.6500	1319.6470	2.27	0	24	0.0058	1	U	R.FSQGLAQDPTTR.R
18046	8 – 20	738.8811	1475.7476	1475.7481	-0.33	1	13	0.59	1	U	R.FSQGLAQDPTTRR.I
18047	8 – 20	492.9233	1475.7481	1475.7481	0.012	1	5	0.37	2	U	R.FSQGLAQDPTTRR.I
18051	8 – 20	492.9253	1475.7540	1475.7481	3.99	1	4	0.45	1	U	R.FSQGLAQDPTTRR.I
28536	20 – 41	662.0650	2644.2309	2644.2306	0.10	1	49	2.4e-05	1	U	R.RIWFGIATAHDFESHDDITEER.L
28537	20 – 41	662.0651	2644.2313	2644.2306	0.24	1	31	0.0011	1	U	R.RIWFGIATAHDFESHDDITEER.L
28538	20 – 41	662.0651	2644.2313	2644.2306	0.26	1	67	5.8e-07	1	U	R.RIWFGIATAHDFESHDDITEER.L
28539	20 – 41	662.0657	2644.2335	2644.2306	1.10	1	69	3.6e-07	1	U	R.RIWFGIATAHDFESHDDITEER.L
28540	20 – 41	662.0659	2644.2345	2644.2306	1.45	1	45	6.5e-05	1	U	R.RIWFGIATAHDFESHDDITEER.L
28541	20 – 41	662.0661	2644.2351	2644.2306	1.69	1	22	0.0092	1	U	R.RIWFGIATAHDFESHDDITEER.L
28543	20 – 41	882.4218	2644.2436	2644.2306	4.90	1	25	0.0045	1	U	R.RIWFGIATAHDFESHDDITEER.L
28544	20 – 41	662.0684	2644.2446	2644.2306	5.28	1	16	0.033	1	U	R.RIWFGIATAHDFESHDDITEER.L
27939	21 – 41	830.3835	2488.1286	2488.1295	-0.36	0	70	4.1e-07	1	U	R.IWFGIATAHDFESHDDITEER.L
27940	21 – 41	623.0400	2488.1311	2488.1295	0.63	0	95	1.4e-09	1	U	R.IWFGIATAHDFESHDDITEER.L
27941	21 – 41	623.0407	2488.1336	2488.1295	1.63	0	35	0.00048	1	U	R.IWFGIATAHDFESHDDITEER.L
27942	21 – 41	623.0408	2488.1343	2488.1295	1.91	0	69	3.1e-07	1	U	R.IWFGIATAHDFESHDDITEER.L
27943	21 – 41	830.3855	2488.1347	2488.1295	2.09	0	20	0.013	1	U	R.IWFGIATAHDFESHDDITEER.L
27944	21 – 41	623.0420	2488.1387	2488.1295	3.70	0	58	4e-06	1	U	R.IWFGIATAHDFESHDDITEER.L
27945	21 – 41	623.0430	2488.1430	2488.1295	5.43	0	49	2.7e-05	1	U	R.IWFGIATAHDFESHDDITEER.L
27411	86 – 106	787.4016	2359.1829	2359.1862	-1.41	0	37	0.00035	1	U	R.PIAHAIWDPHFGQPAVEAFTR.G
27412	86 – 106	787.4024	2359.1853	2359.1862	-0.37	0	41	0.00015	1	U	R.PIAHAIWDPHFGQPAVEAFTR.G

27413	86 – 106	590.8039	2359.1866	2359.1862	0.17	0	51	1.5e-05	1	U	R.PIAHAIWDPHFGQPAVEAFTR.G
27414	86 – 106	590.8047	2359.1897	2359.1862	1.49	0	19	0.016	1	U	R.PIAHAIWDPHFGQPAVEAFTR.G
27415	86 – 106	590.8053	2359.1920	2359.1862	2.45	0	35	0.00057	1	U	R.PIAHAIWDPHFGQPAVEAFTR.G
27416	86 – 106	590.8055	2359.1927	2359.1862	2.78	0	52	1.3e-05	1	U	R.PIAHAIWDPHFGQPAVEAFTR.G
27417	86 – 106	590.8068	2359.1983	2359.1862	5.11	0	34	0.00061	1	U	R.PIAHAIWDPHFGQPAVEAFTR.G
10200	161 – 169	388.8778	1163.6116	1163.6128	-1.00	1	15	0.041	1	U	K.WKPSVSWFK.N
10201	161 – 169	582.8135	1163.6125	1163.6128	-0.21	1	12	0.071	1	U	K.WKPSVSWFK.N
10202	161 – 169	582.8137	1163.6129	1163.6128	0.12	1	9	0.14	1	U	K.WKPSVSWFK.N
10204	161 – 169	388.8787	1163.6142	1163.6128	1.25	1	6	0.28	1	U	K.WKPSVSWFK.N
10205	161 – 169	388.8788	1163.6146	1163.6128	1.58	1	2	0.59	1	U	K.WKPSVSWFK.N
22150	161 – 174	431.2246	1720.8695	1720.8685	0.55	2	16	0.032	1	U	K.WKPSVSWFKNAESR.L
29463	175 – 203	766.4159	3061.6343	3061.6363	-0.63	0	68	6.3e-07	1	U	R.LNHHLSGLFGVSSLAWTGHLVHVAIPGSR.G
29464	175 – 203	613.3346	3061.6364	3061.6363	0.060	0	73	1.9e-07	1	U	R.LNHHLSGLFGVSSLAWTGHLVHVAIPGSR.G
8144	293 – 302	546.2685	1090.5224	1090.5230	-0.49	0	18	0.023	1	U	R.TNFGIGHSMK.D
8146	293 – 302	546.2695	1090.5245	1090.5230	1.39	0	17	0.026	1	U	R.TNFGIGHSMK.D
8595	293 – 302	554.2654	1106.5163	1106.5179	-1.42	0	9	0.14	1	U	R.TNFGIGHSMK.D + Oxidation (M)
8597	293 – 302	369.8465	1106.5178	1106.5179	-0.080	0	2	0.61	1	U	R.TNFGIGHSMK.D + Oxidation (M)
27336	293 – 314	587.5544	2346.1886	2346.1903	-0.70	1	12	0.091	1	U	R.TNFGIGHSMKDLLEAHIPPGGR.L
27337	293 – 314	587.5547	2346.1897	2346.1903	-0.25	1	27	0.0035	1	U	R.TNFGIGHSMKDLLEAHIPPGGR.L
27338	293 – 314	587.5550	2346.1908	2346.1903	0.22	1	13	0.22	1	U	R.TNFGIGHSMKDLLEAHIPPGGR.L
27339	293 – 314	587.5553	2346.1922	2346.1903	0.84	1	2	0.63	1	U	R.TNFGIGHSMKDLLEAHIPPGGR.L
27340	293 – 314	783.0716	2346.1929	2346.1903	1.13	1	20	0.014	1	U	R.TNFGIGHSMKDLLEAHIPPGGR.L
27341	293 – 314	587.5558	2346.1940	2346.1903	1.57	1	17	0.025	1	U	R.TNFGIGHSMKDLLEAHIPPGGR.L
27342	293 – 314	587.5559	2346.1946	2346.1903	1.82	1	2	0.69	1	U	R.TNFGIGHSMKDLLEAHIPPGGR.L
27343	293 – 314	587.5560	2346.1948	2346.1903	1.91	1	15	0.038	1	U	R.TNFGIGHSMKDLLEAHIPPGGR.L
27344	293 – 314	587.5565	2346.1967	2346.1903	2.75	1	16	0.034	1	U	R.TNFGIGHSMKDLLEAHIPPGGR.L
27345	293 – 314	783.0729	2346.1968	2346.1903	2.77	1	32	0.0011	1	U	R.TNFGIGHSMKDLLEAHIPPGGR.L
27346	293 – 314	587.5569	2346.1984	2346.1903	3.46	1	6	0.43	1	U	R.TNFGIGHSMKDLLEAHIPPGGR.L
27429	293 – 314	591.5525	2362.1809	2362.1852	-1.82	1	4	0.64	1	U	R.TNFGIGHSMKDLLEAHIPPGGR.L + Oxidation (M)
27430	293 – 314	591.5527	2362.1817	2362.1852	-1.48	1	0	0.92	1	U	R.TNFGIGHSMKDLLEAHIPPGGR.L + Oxidation (M)
27433	293 – 314	591.5536	2362.1855	2362.1852	0.11	1	20	0.014	1	U	R.TNFGIGHSMKDLLEAHIPPGGR.L + Oxidation (M)
27435	293 – 314	788.4029	2362.1869	2362.1852	0.73	1	9	0.16	1	U	R.TNFGIGHSMKDLLEAHIPPGGR.L + Oxidation (M)
27437	293 – 314	591.5544	2362.1884	2362.1852	1.37	1	16	0.14	1	U	R.TNFGIGHSMKDLLEAHIPPGGR.L + Oxidation (M)
27438	293 – 314	591.5546	2362.1891	2362.1852	1.65	1	14	0.054	1	U	R.TNFGIGHSMKDLLEAHIPPGGR.L + Oxidation (M)
27439	293 – 314	591.5550	2362.1908	2362.1852	2.35	1	32	0.0014	1	U	R.TNFGIGHSMKDLLEAHIPPGGR.L + Oxidation (M)
27440	293 – 314	473.4455	2362.1911	2362.1852	2.51	1	10	0.11	1	U	R.TNFGIGHSMKDLLEAHIPPGGR.L + Oxidation (M)
27441	293 – 314	591.5557	2362.1937	2362.1852	3.60	1	3	0.58	1	U	R.TNFGIGHSMKDLLEAHIPPGGR.L + Oxidation (M)
13213	303 – 314	637.8452	1273.6758	1273.6779	-1.64	0	30	0.0014	1	U	K.DLLEAHIPPGGR.L
13214	303 – 314	637.8452	1273.6759	1273.6779	-1.56	0	39	0.00021	1	U	K.DLLEAHIPPGGR.L
13215	303 – 314	425.5662	1273.6767	1273.6779	-0.91	0	18	0.021	1	U	K.DLLEAHIPPGGR.L
13216	303 – 314	425.5662	1273.6768	1273.6779	-0.88	0	12	0.081	1	U	K.DLLEAHIPPGGR.L
13217	303 – 314	425.5663	1273.6769	1273.6779	-0.73	0	11	0.085	1	U	K.DLLEAHIPPGGR.L
13218	303 – 314	425.5663	1273.6769	1273.6779	-0.72	0	10	0.12	1	U	K.DLLEAHIPPGGR.L
13219	303 – 314	637.8458	1273.6770	1273.6779	-0.70	0	33	0.00078	1	U	K.DLLEAHIPPGGR.L
13220	303 – 314	637.8458	1273.6771	1273.6779	-0.60	0	33	0.00085	1	U	K.DLLEAHIPPGGR.L
13221	303 – 314	425.5664	1273.6773	1273.6779	-0.47	0	7	0.21	1	U	K.DLLEAHIPPGGR.L
13222	303 – 314	637.8459	1273.6773	1273.6779	-0.46	0	31	0.0013	1	U	K.DLLEAHIPPGGR.L
13223	303 – 314	637.8459	1273.6773	1273.6779	-0.46	0	43	9.4e-05	1	U	K.DLLEAHIPPGGR.L
13224	303 – 314	425.5664	1273.6773	1273.6779	-0.43	0	10	0.12	1	U	K.DLLEAHIPPGGR.L
13225	303 – 314	637.8460	1273.6775	1273.6779	-0.31	0	24	0.006	1	U	K.DLLEAHIPPGGR.L
13226	303 – 314	425.5665	1273.6775	1273.6779	-0.27	0	10	0.12	1	U	K.DLLEAHIPPGGR.L
13227	303 – 314	637.8461	1273.6776	1273.6779	-0.25	0	40	0.00018	1	U	K.DLLEAHIPPGGR.L
13228	303 – 314	425.5665	1273.6776	1273.6779	-0.24	0	0	0.98	1	U	K.DLLEAHIPPGGR.L
13230	303 – 314	637.8461	1273.6777	1273.6779	-0.17	0	17	0.028	1	U	K.DLLEAHIPPGGR.L
13231	303 – 314	637.8461	1273.6777	1273.6779	-0.12	0	40	0.00017	1	U	K.DLLEAHIPPGGR.L
13232	303 – 314	425.5666	1273.6779	1273.6779	0.022	0	8	0.18	1	U	K.DLLEAHIPPGGR.L
13233	303 – 314	425.5666	1273.6779	1273.6779	0.043	0	21	0.012	1	U	K.DLLEAHIPPGGR.L
13234	303 – 314	425.5666	1273.6780	1273.6779	0.074	0	12	0.082	1	U	K.DLLEAHIPPGGR.L
13236	303 – 314	637.8463	1273.6781	1273.6779	0.15	0	38	0.00029	1	U	K.DLLEAHIPPGGR.L
13237	303 – 314	425.5667	1273.6781	1273.6779	0.21	0	5	0.31	1	U	K.DLLEAHIPPGGR.L
13238	303 – 314	425.5667	1273.6782	1273.6779	0.24	0	16	0.03	1	U	K.DLLEAHIPPGGR.L
13239	303 – 314	425.5667	1273.6782	1273.6779	0.28	0	2	0.64	1	U	K.DLLEAHIPPGGR.L
13240	303 – 314	637.8464	1273.6783	1273.6779	0.32	0	34	0.00072	1	U	K.DLLEAHIPPGGR.L
13241	303 – 314	637.8465	1273.6784	1273.6779	0.39	0	40	0.00016	1	U	K.DLLEAHIPPGGR.L
13242	303 – 314	425.5668	1273.6784	1273.6779	0.43	0	17	0.023	1	U	K.DLLEAHIPPGGR.L
13243	303 – 314	637.8465	1273.6784	1273.6779	0.45	0	22	0.0081	1	U	K.DLLEAHIPPGGR.L
13244	303 – 314	637.8465	1273.6784	1273.6779	0.45	0	37	0.00035	1	U	K.DLLEAHIPPGGR.L
13245	303 – 314	425.5668	1273.6785	1273.6779	0.52	0	12	0.077	1	U	K.DLLEAHIPPGGR.L
13246	303 – 314	425.5668	1273.6786	1273.6779	0.57	0	4	0.38	1	U	K.DLLEAHIPPGGR.L

13247	303 – 314	425.5668	1273.6786	1273.6779	0.59	0	24	0.005	1	U	K.DLLEAHIPPGGR.L
13249	303 – 314	425.5668	1273.6787	1273.6779	0.62	0	8	0.2	1	U	K.DLLEAHIPPGGR.L
13250	303 – 314	637.8466	1273.6787	1273.6779	0.64	0	27	0.0029	1	U	K.DLLEAHIPPGGR.L
13251	303 – 314	637.8467	1273.6788	1273.6779	0.71	0	30	0.0015	1	U	K.DLLEAHIPPGGR.L
13252	303 – 314	425.5669	1273.6788	1273.6779	0.73	0	4	0.42	1	U	K.DLLEAHIPPGGR.L
13253	303 – 314	637.8467	1273.6788	1273.6779	0.76	0	37	0.00032	1	U	K.DLLEAHIPPGGR.L
13254	303 – 314	425.5669	1273.6789	1273.6779	0.81	0	5	0.36	1	U	K.DLLEAHIPPGGR.L
13255	303 – 314	637.8467	1273.6789	1273.6779	0.83	0	38	0.00026	1	U	K.DLLEAHIPPGGR.L
13256	303 – 314	425.5669	1273.6789	1273.6779	0.83	0	9	0.16	1	U	K.DLLEAHIPPGGR.L
13257	303 – 314	637.8467	1273.6789	1273.6779	0.84	0	42	0.00013	1	U	K.DLLEAHIPPGGR.L
13258	303 – 314	425.5669	1273.6790	1273.6779	0.88	0	11	0.085	1	U	K.DLLEAHIPPGGR.L
13259	303 – 314	425.5670	1273.6791	1273.6779	0.94	0	17	0.025	1	U	K.DLLEAHIPPGGR.L
13260	303 – 314	425.5670	1273.6791	1273.6779	0.96	0	11	0.086	1	U	K.DLLEAHIPPGGR.L
13261	303 – 314	637.8469	1273.6792	1273.6779	1.08	0	6	0.28	1	U	K.DLLEAHIPPGGR.L
13262	303 – 314	425.5670	1273.6793	1273.6779	1.12	0	20	0.015	1	U	K.DLLEAHIPPGGR.L
13263	303 – 314	637.8469	1273.6793	1273.6779	1.16	0	26	0.004	1	U	K.DLLEAHIPPGGR.L
13264	303 – 314	637.8470	1273.6794	1273.6779	1.18	0	36	0.00046	1	U	K.DLLEAHIPPGGR.L
13265	303 – 314	425.5671	1273.6795	1273.6779	1.29	0	7	0.22	1	U	K.DLLEAHIPPGGR.L
13266	303 – 314	637.8470	1273.6795	1273.6779	1.31	0	30	0.0014	1	U	K.DLLEAHIPPGGR.L
13267	303 – 314	637.8471	1273.6796	1273.6779	1.33	0	37	0.00033	1	U	K.DLLEAHIPPGGR.L
13268	303 – 314	425.5674	1273.6802	1273.6779	1.87	0	12	0.081	1	U	K.DLLEAHIPPGGR.L
13269	303 – 314	425.5674	1273.6803	1273.6779	1.88	0	11	0.088	1	U	K.DLLEAHIPPGGR.L
13270	303 – 314	637.8477	1273.6808	1273.6779	2.33	0	47	6.9e-05	1	U	K.DLLEAHIPPGGR.L
21515	397 – 410	838.8781	1675.7416	1675.7438	-1.26	0	79	4.4e-08	1	U	R.DYNPEQNEDNVLAR.M
21516	397 – 410	559.5879	1675.7420	1675.7438	-1.07	0	61	3.2e-06	1	U	R.DYNPEQNEDNVLAR.M
21517	397 – 410	559.5896	1675.7469	1675.7438	1.88	0	33	0.0022	1	U	R.DYNPEQNEDNVLAR.M
25676	452 – 469	693.7179	2078.1320	2078.1313	0.34	0	39	0.0002	1	U	K.QILIEPIFAQWIQSAHGK.T
25677	452 – 469	1040.0741	2078.1336	2078.1313	1.14	0	78	4.8e-08	1	U	K.QILIEPIFAQWIQSAHGK.T
25678	452 – 469	520.5408	2078.1343	2078.1313	1.45	0	19	0.017	1	U	K.QILIEPIFAQWIQSAHGK.T
25679	452 – 469	693.7192	2078.1357	2078.1313	2.15	0	45	5.9e-05	1	U	K.QILIEPIFAQWIQSAHGK.T
26146	470 – 490	1074.0241	2146.0337	2146.0331	0.28	0	103	2.2e-10	1	U	K.TSYGFDVLLSSTSGPAFNAGR.S
26147	470 – 490	537.5161	2146.0353	2146.0331	1.03	0	47	3.6e-05	1	U	K.TSYGFDVLLSSTSGPAFNAGR.S
26150	470 – 490	716.3532	2146.0377	2146.0331	2.15	0	68	4.3e-07	1	U	K.TSYGFDVLLSSTSGPAFNAGR.S
491	546 – 551	366.2095	730.4045	730.4047	-0.27	1	5	0.37	1	U	K.LMPDKK.D
678	546 – 551	374.2059	746.3972	746.3996	-3.25	1	2	0.68	1	U	K.LMPDKK.D + Oxidation (M)
20397	551 – 564	801.8541	1601.6936	1601.6933	0.22	1	20	0.021	1	U	K.KDFGYSFPCDGPGR.G
20398	551 – 564	534.9054	1601.6943	1601.6933	0.65	1	36	0.00066	1	U	K.KDFGYSFPCDGPGR.G
17987	552 – 564	737.8052	1473.5959	1473.5983	-1.67	0	37	0.0002	1	U	K.DFGYSFPCDGPGR.G
17988	552 – 564	492.2062	1473.5968	1473.5983	-1.02	0	20	0.011	1	U	K.DFGYSFPCDGPGR.G
17989	552 – 564	737.8060	1473.5975	1473.5983	-0.58	0	45	3.8e-05	1	U	K.DFGYSFPCDGPGR.G
17990	552 – 564	737.8079	1473.6013	1473.5983	2.02	0	36	0.00029	1	U	K.DFGYSFPCDGPGR.G
3708	685 – 692	449.2796	896.5446	896.5443	0.29	0	2	1.4	1	U	R.TPLANLIR.W
12352	685 – 694	413.9159	1238.7260	1238.7247	0.99	1	4	1.6	1	U	R.TPLANLIRWR.D
20959	693 – 706	546.9866	1637.9379	1637.9365	0.86	2	62	2.3e-06	1	U	R.WRDKPVALSIVQAR.L
20960	693 – 706	410.4918	1637.9381	1637.9365	0.92	2	23	0.016	1	U	R.WRDKPVALSIVQAR.L
13809	695 – 706	432.9258	1295.7557	1295.7561	-0.35	1	12	0.24	1	U	R.DKPVALSIVQAR.L
13810	695 – 706	648.8851	1295.7557	1295.7561	-0.33	1	39	0.00056	1	U	R.DKPVALSIVQAR.L
13811	695 – 706	432.9261	1295.7566	1295.7561	0.36	1	5	1.4	1	U	R.DKPVALSIVQAR.L
13813	695 – 706	432.9265	1295.7576	1295.7561	1.13	1	40	0.00038	1	U	R.DKPVALSIVQAR.L
13814	695 – 706	648.8861	1295.7576	1295.7561	1.14	1	56	1.2e-05	1	U	R.DKPVALSIVQAR.L
7053	697 – 706	527.3249	1052.6353	1052.6342	1.05	0	1	2.3	1	U	K.PVALSIVQAR.L

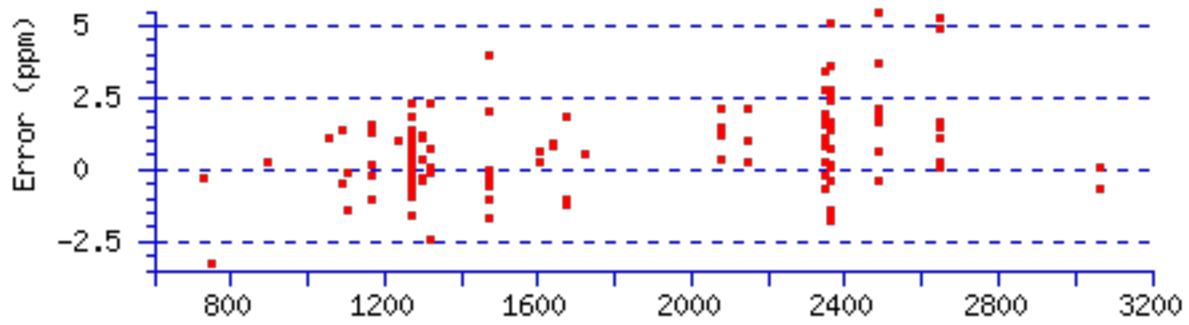

RMS error 1 ppm

Mascot: http://www.matrixscience.com/

MASCOT Search Results

Protein View: sp|P06450|ATPA_SPIOL

>sp|P06450|ATPA_SPIOL ATP synthase subunit alpha, chloroplastic OS=Spinacia oleracea OX=3562 GN=atpA PE=1 SV=1

**Database:**	Uni-Spinach
**Score:**	1295
**Nominal mass (M_r):**	55474
**Calculated pI:**	5.16

Sequence similarity is available as **an NCBI BLAST search of sp|P06450|ATPA_SPIOL against nr**.

Search parameters

**MS data file:**	\\128.97.66.218\tank\windowsVM\Bill Cramer\MGF\wc_QE_021417_Cramer_Gel_1S_4a.mgf
**Enzyme:**	Trypsin/P: cuts C-term side of KR.
**Fixed modifications:**	Carbamidomethyl (C)
**Variable modifications:**	Oxidation (M)

Protein sequence coverage: 45%

Matched peptides shown in ***bold red***.

1 MATIRADEIS KIIR**ERIEGY** **NREVKVVNTG** **TVLQVGDGIA** **R**IHGLDEVMA
51 GELVEFEEGT IGIALNLESN NVGVVLMGDG LMIQEGSSVK ATGR**IAQIPV**
101 **SEAYLGRVIN** **ALAKPIDGRG** **EITASESR**LI ESPAPGIMSR **RSVYEPLQTG**
151 **LIAIDAMIPV** **GR**GQR**ELIIG** **DR**QTGK**TAVA** **TDTILNQQGQ** **NVICVYVAIG**
201 **QKASSVAQVV** **TNFQER**GAME YTIVVAETAD SPATLQYLAP YTGAALAEYF
251 MYR**ERHTLII** **YDDLSK**QAQA YR**QMSLLLR**R PPGR**EAYPGD** **VFYLHSR**LLE
301 RAAKLSSLLG EGSMTALPIV ETQAGDVSAY IPTNVISITD GQIFLSADLF
351 NAGIR**PAINV** **GISVSRVGSA** **AQIKAMKK**VA GKLKLELAQF AELEAFAQFA
401 SDLDKATQNQ LARGQR**LREL** **LK**QPQSAPLT VEEQVMTIYT GTNGYLDSLE
451 LDQVR**KYLVE** **LR**TYVK**TNKP** **EFQEIISSTK** **TFTEEAEALL** **KEAIQEQMER**
501 FLLQEQA

Unformatted sequence string: **507 residues** (for pasting into other applications).

Sort peptides by ◉ Residue Number ○ Increasing Mass ○ Decreasing Mass

Show predicted peptides also

Query	Start – End	Observed	Mr(expt)	Mr(calc)	ppm	M	Score	Expect	Rank	U	Peptide
6665	15 – 22	518.7615	1035.5085	1035.5097	-1.17	1	23	0.058	1	U	R.ERIEGYNR.E
28734	17 – 41	672.6171	2686.4393	2686.4402	-0.35	2	16	0.034	1	U	R.IEGYNREVKVVNTGTVLQVGDGIAR.I
20324	26 – 41	533.6335	1597.8787	1597.8788	-0.044	0	41	0.00014	1	U	K.VVNTGTVLQVGDGIAR.I
20325	26 – 41	799.9466	1597.8787	1597.8788	-0.034	0	56	5.8e-06	1	U	K.VVNTGTVLQVGDGIAR.I
20326	26 – 41	799.9470	1597.8795	1597.8788	0.41	0	77	6.1e-08	1	U	K.VVNTGTVLQVGDGIAR.I
20327	26 – 41	799.9471	1597.8797	1597.8788	0.58	0	92	2.6e-09	1	U	K.VVNTGTVLQVGDGIAR.I
20328	26 – 41	533.6345	1597.8815	1597.8788	1.71	0	4	0.39	1	U	K.VVNTGTVLQVGDGIAR.I
16638	95 – 107	472.9332	1415.7778	1415.7772	0.42	0	40	0.00016	1	U	R.IAQIPVSEAYLGR.V
16639	95 – 107	708.8966	1415.7786	1415.7772	0.98	0	41	0.00013	1	U	R.IAQIPVSEAYLGR.V
419	108 – 114	364.7372	727.4598	727.4592	0.82	0	7	0.21	1	U	R.VINALAK.P
13032	108 – 119	422.9222	1265.7449	1265.7455	-0.51	1	14	0.14	1	U	R.VINALAKPIDGR.G
13033	108 – 119	633.8800	1265.7454	1265.7455	-0.11	1	46	8.6e-05	1	U	R.VINALAKPIDGR.G
13034	108 – 119	422.9226	1265.7460	1265.7455	0.37	1	8	0.55	1	U	R.VINALAKPIDGR.G
26436	108 – 128	733.0688	2196.1847	2196.1862	-0.69	2	19	0.016	1	U	R.VINALAKPIDGRGEITASESR.L
26437	108 – 128	550.0542	2196.1876	2196.1862	0.64	2	20	0.013	1	U	R.VINALAKPIDGRGEITASESR.L
26438	108 – 128	733.0699	2196.1878	2196.1862	0.74	2	10	0.1	1	U	R.VINALAKPIDGRGEITASESR.L
26439	108 – 128	550.0546	2196.1894	2196.1862	1.46	2	9	0.14	1	U	R.VINALAKPIDGRGEITASESR.L
18290	115 – 128	496.5859	1486.7359	1486.7376	-1.14	1	2	0.81	1	U	K.PIDGRGEITASESR.L
18291	115 – 128	496.5867	1486.7384	1486.7376	0.54	1	12	0.071	1	U	K.PIDGRGEITASESR.L
27602	141 – 162	1200.1607	2398.3069	2398.3042	1.12	1	10	0.36	1	U	R.RSVYEPLQTGLIAIDAMIPVGR.G
27603	141 – 162	800.4431	2398.3075	2398.3042	1.36	1	65	1.1e-06	1	U	R.RSVYEPLQTGLIAIDAMIPVGR.G
27604	141 – 162	600.5843	2398.3081	2398.3042	1.63	1	32	0.0027	1	U	R.RSVYEPLQTGLIAIDAMIPVGR.G
27665	141 – 162	805.7773	2414.3100	2414.2991	4.50	1	73	1.9e-07	1	U	R.RSVYEPLQTGLIAIDAMIPVGR.G + Oxidation (M)
26703	142 – 162	748.4108	2242.2106	2242.2031	3.32	0	74	1.1e-07	1	U	R.SVYEPLQTGLIAIDAMIPVGR.G
26811	142 – 162	753.7393	2258.1960	2258.1980	-0.92	0	72	1.7e-07	1	U	R.SVYEPLQTGLIAIDAMIPVGR.G + Oxidation (M)

26812	142 - 162	1130.1077	2258.2009	2258.1980	1.26	0	60	2.4e-06	1	U	R.SVYEPLQTGLIAIDAMIPVGR.G + Oxidation (M)
1952	166 - 172	408.2344	814.4542	814.4548	-0.75	0	27	0.0031	1	U	R.ELIIGDR.Q
1955	166 - 172	408.2353	814.4561	814.4548	1.51	0	11	0.098	1	U	R.ELIIGDR.Q
29086	177 - 202	935.4933	2803.4581	2803.4538	1.52	0	100	4.1e-10	1	U	K.TAVATDTILNQQGQNVICVYVAIGQK.A
29087	177 - 202	1402.7368	2803.4590	2803.4538	1.84	0	55	7.5e-06	1	U	K.TAVATDTILNQQGQNVICVYVAIGQK.A
19260	203 - 216	512.5983	1534.7732	1534.7740	-0.53	0	18	0.019	1	U	K.ASSVAQVVTNFQER.G
19261	203 - 216	768.3940	1534.7734	1534.7740	-0.39	0	71	2.1e-07	1	U	K.ASSVAQVVTNFQER.G
19263	203 - 216	768.3943	1534.7741	1534.7740	0.11	0	40	0.00019	1	U	K.ASSVAQVVTNFQER.G
19264	203 - 216	768.3949	1534.7753	1534.7740	0.90	0	66	6.2e-07	1	U	K.ASSVAQVVTNFQER.G
19265	203 - 216	768.3957	1534.7768	1534.7740	1.84	0	8	0.18	1	U	K.ASSVAQVVTNFQER.G
20410	254 - 266	534.9566	1601.8480	1601.8413	4.19	1	43	9.2e-05	1	U	R.ERHTLIIYDDLSK.Q
14309	256 - 266	659.3556	1316.6967	1316.6976	-0.67	0	41	0.00014	1	U	R.HTLIIYDDLSK.Q
14310	256 - 266	439.9066	1316.6978	1316.6976	0.18	0	39	0.00021	1	U	R.HTLIIYDDLSK.Q
14311	256 - 266	659.3570	1316.6994	1316.6976	1.33	0	35	0.0023	1	U	R.HTLIIYDDLSK.Q
14312	256 - 266	439.9073	1316.7001	1316.6976	1.91	0	27	0.0029	1	U	R.HTLIIYDDLSK.Q
2963	273 - 279	430.7544	859.4943	859.4949	-0.73	0	24	0.039	1	U	R.QMSLLLR.R
2964	273 - 279	430.7545	859.4944	859.4949	-0.58	0	33	0.0057	1	U	R.QMSLLLR.R
19582	285 - 297	777.3723	1552.7301	1552.7310	-0.60	0	45	6.1e-05	1	U	R.EAYPGDVFYLHSR.L
19583	285 - 297	777.3726	1552.7306	1552.7310	-0.27	0	46	4.8e-05	1	U	R.EAYPGDVFYLHSR.L
19584	285 - 297	518.5844	1552.7314	1552.7310	0.22	0	35	0.00052	1	U	R.EAYPGDVFYLHSR.L
19585	285 - 297	518.5852	1552.7339	1552.7310	1.86	0	13	0.062	1	U	R.EAYPGDVFYLHSR.L
19586	285 - 297	518.5856	1552.7348	1552.7310	2.45	0	36	0.00044	1	U	R.EAYPGDVFYLHSR.L
8717	356 - 366	556.8246	1111.6347	1111.6349	-0.20	0	9	0.16	1	U	R.PAINVGISVSR.V
8718	356 - 366	556.8248	1111.6351	1111.6349	0.16	0	47	4e-05	1	U	R.PAINVGISVSR.V
12552	367 - 378	416.5755	1246.7047	1246.7067	-1.58	2	0	0.92	1	U	R.VGSAAQIKAMKK.V + Oxidation (M)
1106	417 - 422	386.2573	770.5001	770.5014	-1.62	1	2	3.9	8	U	R.LRELLK.Q
4253	456 - 462	460.7821	919.5496	919.5491	0.53	1	9	0.36	1	U	R.KYLVELR.T
1496	457 - 462	396.7341	791.4536	791.4541	-0.70	0	15	0.24	1	U	K.YLVELR.T
1499	457 - 462	396.7341	791.4537	791.4541	-0.52	0	25	0.025	1	U	K.YLVELR.T
20686	467 - 480	811.4215	1620.8284	1620.8359	-4.58	1	7	0.24	1	U	K.TNKPEFQEIISSTK.T
20687	467 - 480	811.4217	1620.8289	1620.8359	-4.33	1	13	0.056	1	U	K.TNKPEFQEIISSTK.T
20689	467 - 480	541.2859	1620.8360	1620.8359	0.083	1	30	0.0015	1	U	K.TNKPEFQEIISSTK.T
20690	467 - 480	541.2894	1620.8463	1620.8359	6.44	1	46	4.8e-05	1	U	K.TNKPEFQEIISSTK.T
12628	481 - 491	417.8872	1250.6398	1250.6394	0.28	0	47	4.6e-05	1	U	K.TFTEEAEALLK.E
12629	481 - 491	626.3278	1250.6410	1250.6394	1.26	0	39	0.00022	1	U	K.TFTEEAEALLK.E
27456	481 - 500	789.3907	2365.1504	2365.1471	1.40	1	59	2.7e-06	1	U	K.TFTEEAEALLKEAIQEQMER.F
27516	481 - 500	794.7237	2381.1493	2381.1420	3.05	1	6	0.3	1	U	K.TFTEEAEALLKEAIQEQMER.F + Oxidation (M)
9278	492 - 500	567.2667	1132.5188	1132.5182	0.54	0	48	6.9e-05	1	U	K.EAIQEQMER.F
9279	492 - 500	567.2668	1132.5191	1132.5182	0.81	0	39	0.00054	1	U	K.EAIQEQMER.F
9744	492 - 500	575.2627	1148.5108	1148.5132	-2.06	0	17	0.067	1	U	K.EAIQEQMER.F + Oxidation (M)
9747	492 - 500	575.2641	1148.5137	1148.5132	0.51	0	9	0.4	1	U	K.EAIQEQMER.F + Oxidation (M)

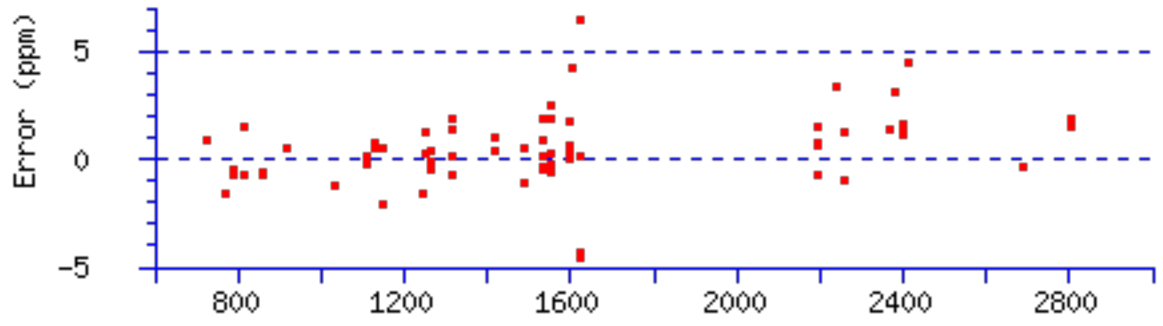

RMS error 1 ppm

Mascot: http://www.matrixscience.com/

MASCOT Search Results

Protein View: tr|A0A0K9R886|A0A0K9R886_SPIOL

>tr|A0A0K9R886|A0A0K9R886_SPIOL Uncharacterized protein OS=Spinacia oleracea OX=3562 GN=SOVF_102250 PE=4 SV=1

**Database:**	Uni-Spinach
**Score:**	1261
**Nominal mass (M_r):**	23145
**Calculated pI:**	9.76

Sequence similarity is available as **an NCBI BLAST search of tr|A0A0K9R886|A0A0K9R886_SPIOL against nr**.

Search parameters

**MS data file:**	\\128.97.66.218\tank\windowsVM\Bill Cramer\MGF\wc_QE_021417_Cramer_Gel_1S_4a.mgf
**Enzyme:**	Trypsin/P: cuts C-term side of KR.
**Fixed modifications:**	**Carbamidomethyl (C)**
**Variable modifications:**	**Oxidation (M)**

Protein sequence coverage: 68%

Matched peptides shown in ***bold red***.

```
  1 MAMATQATLF SPSSLSSAKP IDTRLTTSFK QPSAVTFASK PASRHHSIRA
 51 AAAAEGKAAA ATETKEAPKG FTPPELDPNT PSPIFAGSTG GLLRKAQVEE
101 FYVITWESPK EQIFEMPTGG AAIMREGPNL LKLARKEQCL ALGTRLRSKY
151 KIKYQFYRVF PSGEVQYLHP KDGVYPEKVN PGRQGVGLNM RSIGKNVSPI
201 EVKFTGKQPY DL
```

Unformatted sequence string: **212 residues** (for pasting into other applications).

Sort peptides by ◉ Residue Number ○ Increasing Mass ○ Decreasing Mass

Show predicted peptides also

Query	Start – End	Observed	Mr(expt)	Mr(calc)	ppm	M	Score	Expect	Rank	U	Peptide
10827	58 – 69	594.3154	1186.6162	1186.6193	-2.64	1	54	1e-05	1	U	K.AAAATETKEAPK.G
10828	58 – 69	396.5460	1186.6163	1186.6193	-2.54	1	44	7.4e-05	1	U	K.AAAATETKEAPK.G
28156	70 – 94	1271.1548	2540.2951	2540.2911	1.57	0	58	3.4e-06	1	U	K.GFTPPELDPNTPSPIFAGSTGGLLR.K
28157	70 – 94	847.7725	2540.2956	2540.2911	1.78	0	60	2.3e-06	1	U	K.GFTPPELDPNTPSPIFAGSTGGLLR.K
28158	70 – 94	636.0818	2540.2981	2540.2911	2.76	0	58	3.3e-06	1	U	K.GFTPPELDPNTPSPIFAGSTGGLLR.K
28671	70 – 95	668.1047	2668.3895	2668.3861	1.30	1	54	9.4e-06	1	U	K.GFTPPELDPNTPSPIFAGSTGGLLRK.A
28672	70 – 95	890.4709	2668.3908	2668.3861	1.77	1	45	6.1e-05	1	U	K.GFTPPELDPNTPSPIFAGSTGGLLRK.A
24696	95 – 110	489.2546	1952.9893	1952.9884	0.48	1	15	0.036	1	U	R.KAQVEEFYVITWESPK.E
24698	95 – 110	977.5026	1952.9906	1952.9884	1.13	1	104	1.9e-10	1	U	R.KAQVEEFYVITWESPK.E
24699	95 – 110	977.5026	1952.9906	1952.9884	1.16	1	60	2.4e-06	1	U	R.KAQVEEFYVITWESPK.E
24700	95 – 110	652.0044	1952.9915	1952.9884	1.61	1	38	0.00027	1	U	R.KAQVEEFYVITWESPK.E
24701	95 – 110	652.0047	1952.9924	1952.9884	2.05	1	52	1.3e-05	1	U	R.KAQVEEFYVITWESPK.E
24703	95 – 110	652.0066	1952.9980	1952.9884	4.96	1	32	0.001	1	U	R.KAQVEEFYVITWESPK.E
23417	96 – 110	913.4569	1824.8992	1824.8934	3.15	0	93	1.7e-09	1	U	K.AQVEEFYVITWESPK.E
23418	96 – 110	609.3073	1824.9001	1824.8934	3.67	0	72	2e-07	1	U	K.AQVEEFYVITWESPK.E
21131	111 – 125	825.9026	1649.7906	1649.7905	0.048	0	64	9.5e-07	1	U	K.EQIFEMPTGGAAIMR.E
21132	111 – 125	825.9031	1649.7917	1649.7905	0.72	0	27	0.0029	1	U	K.EQIFEMPTGGAAIMR.E
21133	111 – 125	550.9379	1649.7918	1649.7905	0.78	0	49	2.4e-05	1	U	K.EQIFEMPTGGAAIMR.E
21365	111 – 125	833.8991	1665.7836	1665.7854	-1.13	0	24	0.0061	1	U	K.EQIFEMPTGGAAIMR.E + Oxidation (M)
21366	111 – 125	833.8991	1665.7837	1665.7854	-1.05	0	74	1.2e-07	1	U	K.EQIFEMPTGGAAIMR.E + Oxidation (M)
21367	111 – 125	833.8992	1665.7838	1665.7854	-0.99	0	20	0.012	1	U	K.EQIFEMPTGGAAIMR.E + Oxidation (M)
21368	111 – 125	833.8993	1665.7840	1665.7854	-0.87	0	20	0.012	1	U	K.EQIFEMPTGGAAIMR.E + Oxidation (M)
21370	111 – 125	556.2688	1665.7847	1665.7854	-0.44	0	25	0.0041	1	U	K.EQIFEMPTGGAAIMR.E + Oxidation (M)
21372	111 – 125	556.2695	1665.7868	1665.7854	0.79	0	32	0.001	1	U	K.EQIFEMPTGGAAIMR.E + Oxidation (M)
21597	111 – 125	841.8967	1681.7788	1681.7804	-0.94	0	16	0.029	1	U	K.EQIFEMPTGGAAIMR.E + 2 Oxidation (M)
21598	111 – 125	841.8973	1681.7801	1681.7804	-0.17	0	20	0.013	1	U	K.EQIFEMPTGGAAIMR.E + 2 Oxidation (M)

21599	111 - 125	561.6008	1681.7806	1681.7804	0.15	0	7	0.23	1	U	K.EQIFEMPTGGAAIMR.E + 2 Oxidation (M)
21600	111 - 125	841.8978	1681.7811	1681.7804	0.45	0	31	0.0014	1	U	K.EQIFEMPTGGAAIMR.E + 2 Oxidation (M)
21602	111 - 125	841.8986	1681.7827	1681.7804	1.37	0	13	0.058	1	U	K.EQIFEMPTGGAAIMR.E + 2 Oxidation (M)
1080	126 - 132	385.7240	769.4335	769.4334	0.19	0	9	0.64	2	U	R.EGPNLLK.L
8677	126 - 135	555.8351	1109.6556	1109.6557	-0.026	1	38	0.00028	1	U	R.EGPNLLKLAR.K
18863	133 - 145	505.9521	1514.8345	1514.8351	-0.41	2	6	0.26	1	U	K.LARKEQCLALGTR.L
10497	136 - 145	392.5448	1174.6125	1174.6128	-0.24	1	23	0.0073	1	U	R.KEQCLALGTR.L
10498	136 - 145	588.3136	1174.6127	1174.6128	-0.12	1	61	8.3e-06	1	U	R.KEQCLALGTR.L
10499	136 - 145	392.5451	1174.6136	1174.6128	0.65	1	35	0.0023	1	U	R.KEQCLALGTR.L
10500	136 - 145	588.3142	1174.6139	1174.6128	0.89	1	39	0.00021	1	U	R.KEQCLALGTR.L
17345	136 - 147	361.9572	1443.7998	1443.7980	1.28	2	2	0.67	1	U	R.KEQCLALGTRLR.S
6905	137 - 145	524.2664	1046.5182	1046.5179	0.32	0	21	0.011	1	U	K.EQCLALGTR.L
6906	137 - 145	524.2668	1046.5190	1046.5179	1.12	0	30	0.0015	1	U	K.EQCLALGTR.L
27984	152 - 171	625.5867	2498.3177	2498.3110	2.67	2	2	0.61	1	U	K.IKYQFYRVFPSGEVQYLHPK.D
1198	154 - 158	388.6888	775.3630	775.3653	-2.92	0	9	0.54	1	U	K.YQFYR.V
1199	154 - 158	388.6898	775.3650	775.3653	-0.45	0	4	0.42	1	U	K.YQFYR.V
1201	154 - 158	388.6904	775.3662	775.3653	1.18	0	3	0.51	1	U	K.YQFYR.V
1202	154 - 158	388.6904	775.3662	775.3653	1.21	0	0	0.97	1	U	K.YQFYR.V
26802	154 - 171	565.2913	2257.1359	2257.1320	1.74	1	4	0.41	1	U	K.YQFYRVFPSGEVQYLHPK.D
18554	159 - 171	750.8949	1499.7752	1499.7773	-1.41	0	30	0.0014	1	U	R.VFPSGEVQYLHPK.D
18555	159 - 171	750.8951	1499.7756	1499.7773	-1.14	0	39	0.00024	1	U	R.VFPSGEVQYLHPK.D
18556	159 - 171	750.8955	1499.7765	1499.7773	-0.50	0	42	0.00011	1	U	R.VFPSGEVQYLHPK.D
18557	159 - 171	750.8958	1499.7770	1499.7773	-0.15	0	18	0.022	1	U	R.VFPSGEVQYLHPK.D
18558	159 - 171	750.8958	1499.7771	1499.7773	-0.087	0	39	0.00024	1	U	R.VFPSGEVQYLHPK.D
18559	159 - 171	500.9330	1499.7773	1499.7773	0.015	0	6	0.29	1	U	R.VFPSGEVQYLHPK.D
18560	159 - 171	500.9332	1499.7777	1499.7773	0.27	0	7	0.24	1	U	R.VFPSGEVQYLHPK.D
18561	159 - 171	500.9332	1499.7778	1499.7773	0.33	0	13	0.056	1	U	R.VFPSGEVQYLHPK.D
18562	159 - 171	750.8962	1499.7779	1499.7773	0.40	0	41	0.00015	1	U	R.VFPSGEVQYLHPK.D
18563	159 - 171	500.9334	1499.7784	1499.7773	0.74	0	10	0.1	1	U	R.VFPSGEVQYLHPK.D
18564	159 - 171	750.8965	1499.7785	1499.7773	0.81	0	41	0.00016	1	U	R.VFPSGEVQYLHPK.D
18565	159 - 171	750.8965	1499.7785	1499.7773	0.84	0	36	0.0004	1	U	R.VFPSGEVQYLHPK.D
18567	159 - 171	750.8967	1499.7789	1499.7773	1.10	0	31	0.0011	1	U	R.VFPSGEVQYLHPK.D
18568	159 - 171	500.9336	1499.7790	1499.7773	1.15	0	20	0.014	1	U	R.VFPSGEVQYLHPK.D
18569	159 - 171	500.9337	1499.7793	1499.7773	1.36	0	19	0.017	1	U	R.VFPSGEVQYLHPK.D
18570	159 - 171	500.9338	1499.7796	1499.7773	1.54	0	21	0.01	1	U	R.VFPSGEVQYLHPK.D
18572	159 - 171	750.8980	1499.7814	1499.7773	2.79	0	1	0.82	1	U	R.VFPSGEVQYLHPK.D
18573	159 - 171	500.9345	1499.7818	1499.7773	3.00	0	9	0.13	1	U	R.VFPSGEVQYLHPK.D
14585	172 - 183	665.8410	1329.6674	1329.6677	-0.24	1	38	0.00029	1	U	K.DGVYPEKVNPGR.Q
14586	172 - 183	444.2299	1329.6678	1329.6677	0.075	1	30	0.0014	1	U	K.DGVYPEKVNPGR.Q
14587	172 - 183	444.2304	1329.6695	1329.6677	1.33	1	29	0.0019	1	U	K.DGVYPEKVNPGR.Q
26372	172 - 191	547.2836	2185.1053	2185.1062	-0.42	2	10	0.11	1	U	K.DGVYPEKVNPGRQGVGLNMR.S
26373	172 - 191	729.3762	2185.1068	2185.1062	0.25	2	10	0.13	1	U	K.DGVYPEKVNPGRQGVGLNMR.S
3259	184 - 191	437.7326	873.4506	873.4491	1.81	0	2	0.7	1	U	R.QGVGLNMR.S
13125	192 - 203	424.2496	1269.7269	1269.7292	-1.83	1	2	0.76	1	U	R.SIGKNVSPIEVK.F
13127	192 - 203	635.8723	1269.7301	1269.7292	0.65	1	22	0.04	1	U	R.SIGKNVSPIEVK.F
13129	192 - 203	424.2509	1269.7308	1269.7292	1.23	1	4	0.58	1	U	R.SIGKNVSPIEVK.F
21891	192 - 207	568.6610	1702.9613	1702.9618	-0.27	2	33	0.002	1	U	R.SIGKNVSPIEVKFTGK.Q
14346	196 - 207	440.2511	1317.7314	1317.7292	1.62	1	28	0.0023	1	U	K.NVSPIEVKFTGK.Q

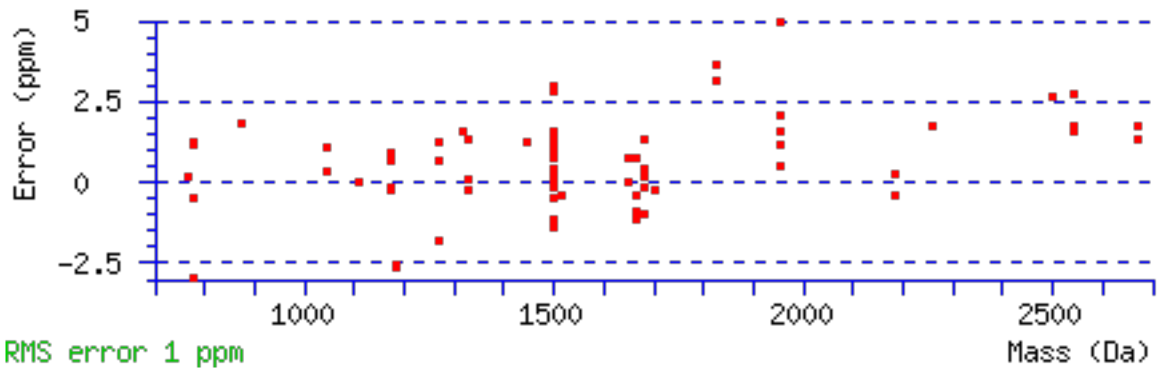

Mascot: http://www.matrixscience.com/

MASCOT Search Results

Protein View: tr|A0A0K9RFL8|A0A0K9RFL8_SPIOL

>tr|A0A0K9RFL8|A0A0K9RFL8_SPIOL AAA domain-containing protein OS=Spinacia oleracea OX=3562 GN=SOVF_078600 PE=3 SV=1

**Database:**	Uni-Spinach
**Score:**	753
**Nominal mass (M_r):**	75763
**Calculated pI:**	5.65

Sequence similarity is available as an NCBI BLAST search of tr|A0A0K9RFL8|A0A0K9RFL8_SPIOL against nr.

Search parameters

**MS data file:**	\\128.97.66.218\tank\windowsVM\Bill Cramer\MGF\wc_QE_021417_Cramer_Gel_1S_4a.mgf
**Enzyme:**	Trypsin/P: cuts C-term side of KR.
**Fixed modifications:**	Carbamidomethyl (C)
**Variable modifications:**	Oxidation (M)

Protein sequence coverage: 44%

Matched peptides shown in ***bold red***.

1 MAANPLLSSS FLGNGVLFYP PTPKTTKPIL PSSSRRKQFI ITQSLIPNSQ
51 NPKFLSKSAL TALLLSSTLA SQQAFAADNL SPPPQSPQVI EAQPTNPGLP
101 NSSPFSQNLV LNAPKPLPDP LPEGSQWRYS EFLNAVKKGK VERVRFSK**DG**
151 **SVLQLTAVDG** **KR**ASVVVPND PDLIDILAMN GVDISVSEGE GGNGLFGFIG
201 NLLFPLLAFA GLFFLFR**RAQ** **GGPGGPGGLG** **GPMDFGR**SK**S** **KFQEVPETGV**
251 **SFADVAGADQ** **AKLELQEVVD** **FLKNPDKYTA** **LGAK**IPKGCL LVGPPGTGKT
301 LLARAVAGEA GTPFFSCAAS EFVELFVGVG ASRVRDLFEK AKSK**APCIVF**
351 **IDEIDAVGR**Q R**GAGMGGGND** **ER**EQTINQLL TEMDGFSGNS GVIVLAATNR
401 PDVLDSALLR PGRFDR**QVTV** **DRPDVAGR**VK ILQVHSRGKA LAK**DVDFDK**V
451 AR**RTPGFTGA** **DLQNLMNEAA** **ILAAR**RELKE **ISKDEISDAL** **ER**IIAGPEKK
501 NAVVSEEKKR **LVAYHEAGHA** **LVGALMPEYD** **PVAK**ISIIPR **GQAGGLTFFA**
551 **PSEERLESGL** **YSRSYLENQM** **AVALGGRVAE** **EVIFGENNVT** **TGASSDFMQV**
601 **SR**VARQMVER FGFSK**KIGQL** **AVGGAGGNPF** **LGQQMSSAKD** **YSMATADIVD**
651 **GEVRELVEVA** **YKRATEIINT** **HIDILHKLAQ** **LLIEK**ETIDG EEFMSLFIDG
701 QAELYVS

Unformatted sequence string: 707 residues (for pasting into other applications).

Sort peptides by ◉ Residue Number ○ Increasing Mass ○ Decreasing Mass

Show predicted peptides also

Query	Start – End	Observed	Mr(expt)	Mr(calc)	ppm	M	Score	Expect	Rank	U	Peptide
13944	149 – 161	651.8497	1301.6849	1301.6827	1.72	0	31	0.0013	1	U	K.DGSVLQLTAVDGK.R
17664	149 – 162	729.8987	1457.7829	1457.7838	-0.63	1	55	7.3e-06	1	U	K.DGSVLQLTAVDGKR.A
17665	149 – 162	486.9351	1457.7834	1457.7838	-0.31	1	41	0.00015	1	U	K.DGSVLQLTAVDGKR.A
23599	218 – 237	614.3002	1839.8788	1839.8799	-0.60	1	21	0.012	1	U	R.RAQGGPGGPGGLGGPMDFGR.S
21626	219 – 237	842.8973	1683.7801	1683.7788	0.78	0	33	0.00077	1	U	R.AQGGPGGPGGLGGPMDFGR.S
21628	219 – 237	562.2681	1683.7823	1683.7788	2.11	0	18	0.021	1	U	R.AQGGPGGPGGLGGPMDFGR.S
21836	219 – 237	850.8931	1699.7716	1699.7737	-1.21	0	27	0.0098	1	U	R.AQGGPGGPGGLGGPMDFGR.S + Oxidation (M)
21838	219 – 237	567.5985	1699.7737	1699.7737	-0.015	0	10	0.53	1	U	R.AQGGPGGPGGLGGPMDFGR.S + Oxidation (M)
21839	219 – 237	850.8942	1699.7738	1699.7737	0.089	0	16	0.12	1	U	R.AQGGPGGPGGLGGPMDFGR.S + Oxidation (M)
21840	219 – 237	850.8956	1699.7766	1699.7737	1.69	0	37	0.00036	1	U	R.AQGGPGGPGGLGGPMDFGR.S + Oxidation (M)
27512	240 – 262	794.3942	2380.1608	2380.1547	2.56	1	44	7e-05	1	U	K.SKFQEVPETGVSFADVAGADQAK.L
14628	263 – 273	666.8759	1331.7372	1331.7337	2.65	0	50	1.9e-05	1	U	K.LELQEVVDFLK.N
10535	274 – 284	393.2115	1176.6128	1176.6139	-0.90	1	18	0.024	1	U	K.NPDKYTALGAK.I
10536	274 – 284	589.3141	1176.6137	1176.6139	-0.15	1	38	0.00025	1	U	K.NPDKYTALGAK.I
21493	345 – 359	837.9295	1673.8444	1673.8447	-0.19	0	75	8.6e-08	1	U	K.APCIVFIDEIDAVGR.Q
6334	362 – 372	510.7114	1019.4083	1019.4091	-0.71	0	15	0.034	1	U	R.GAGMGGGNDER.E
14175	417 – 428	438.2371	1311.6893	1311.6895	-0.15	1	18	0.02	1	U	R.QVTVDRPDVAGR.V
14176	417 – 428	438.2372	1311.6899	1311.6895	0.27	1	11	0.086	1	U	R.QVTVDRPDVAGR.V
583	444 – 449	369.6687	737.3228	737.3232	-0.56	0	3	1.9	1	U	K.DVDFDK.V

27721	453 - 475	810.7591	2429.2554	2429.2485	2.83	1	84	1.2e-08	1	U	R.RTPGFTGADLQNLMNEAAILAAR.R
26901	454 - 475	758.7256	2273.1550	2273.1474	3.36	0	86	9e-09	1	U	R.TPGFTGADLQNLMNEAAILAAR.R
18646	480 - 492	752.8787	1503.7428	1503.7416	0.81	1	25	0.0045	1	U	K.EISKDEISDALER.I
18647	480 - 492	502.2550	1503.7431	1503.7416	0.95	1	19	0.026	1	U	K.EISKDEISDALER.I
28182	511 - 534	638.5794	2550.2885	2550.2941	-2.16	0	16	0.034	1	U	R.LVAYHEAGHALVGALMPEYDPVAK.I
28243	511 - 534	856.4387	2566.2942	2566.2890	2.04	0	7	0.21	1	U	R.LVAYHEAGHALVGALMPEYDPVAK.I + Oxidation (M)
28244	511 - 534	642.5809	2566.2946	2566.2890	2.18	0	29	0.0017	1	U	R.LVAYHEAGHALVGALMPEYDPVAK.I + Oxidation (M)
19778	541 - 555	783.8810	1565.7475	1565.7474	0.081	0	52	1.3e-05	1	U	R.GQAGGLTFFAPSEER.L
4329	556 - 563	462.7432	923.4718	923.4712	0.67	0	5	0.37	1	U	R.LESGLYSR.S
18719	564 - 577	754.8790	1507.7434	1507.7453	-1.22	0	62	1.6e-06	1	U	R.SYLENQMAVALGGR.V
19022	564 - 577	762.8778	1523.7410	1523.7402	0.55	0	83	1.5e-08	1	U	R.SYLENQMAVALGGR.V + Oxidation (M)
19023	564 - 577	762.8781	1523.7416	1523.7402	0.95	0	64	9e-07	1	U	R.SYLENQMAVALGGR.V + Oxidation (M)
28805	578 - 602	901.7558	2702.2455	2702.2494	-1.45	0	19	0.018	1	U	R.VAEEVIFGENNVTTGASSDFMQVSR.V + Oxidation (M)
27171	616 - 639	772.7439	2315.2100	2315.2056	1.89	1	11	0.092	1	U	K.KIGQLAVGGAGGNPFLGQQMSSAK.D
20999	640 - 654	821.3748	1640.7350	1640.7352	-0.13	0	91	2.9e-09	1	U	K.DYSMATADIVDGEVR.E
4906	655 - 662	475.7643	949.5140	949.5120	2.13	0	10	0.33	1	U	R.ELVEVAYK.R
8576	655 - 663	553.8130	1105.6115	1105.6131	-1.45	1	17	0.025	1	U	R.ELVEVAYKR.A
20621	664 - 677	405.2299	1616.8904	1616.8886	1.14	0	13	0.059	1	U	R.ATEIINTHIDILHK.L
4373	678 - 685	464.2978	926.5811	926.5800	1.20	0	15	0.095	1	U	K.LAQLLIEK.E

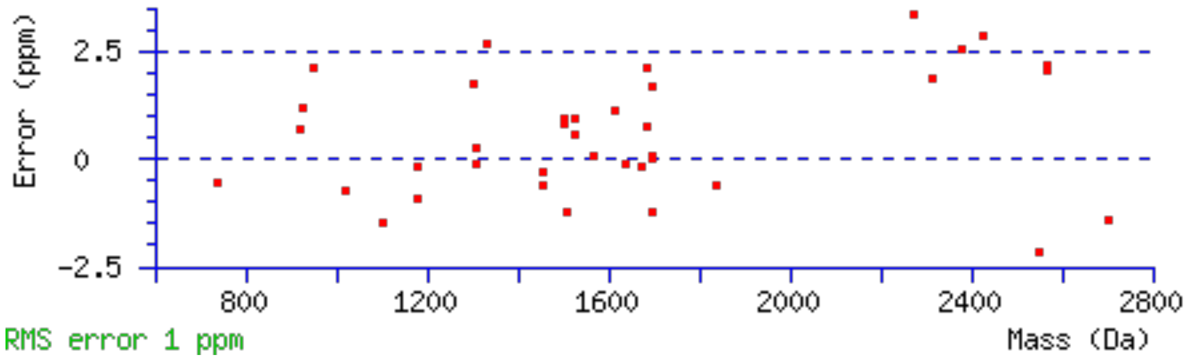

MASCOT Search Results

Protein View: tr|A0A0K9R1K7|A0A0K9R1K7_SPIOL

>tr|A0A0K9R1K7|A0A0K9R1K7_SPIOL Chlorophyll a-b binding protein, chloroplastic OS=Spinacia oleracea OX=3562 GN=SOVF_123890 PE=3 SV=1

**Database:**	Uni-Spinach
**Score:**	677
**Nominal mass (M_r):**	29569
**Calculated pI:**	7.85

Sequence similarity is available as **an NCBI BLAST search of tr|A0A0K9R1K7|A0A0K9R1K7_SPIOL against nr**.

Search parameters

**MS data file:**	\\128.97.66.218\tank\windowsVM\Bill Cramer\MGF\wc_QE_021417_Cramer_Gel_1S_4a.mgf
**Enzyme:**	Trypsin/P: cuts C-term side of KR.
**Fixed modifications:**	Carbamidomethyl (C)
**Variable modifications:**	Oxidation (M)

Protein sequence coverage: 44%

Matched peptides shown in ***bold red***.

1 MATQALVSSS LTSSVETARQ ILGARSGLAP SSLRKNSFVV RAASTPPVK**Q**
51 **NANRPLWFAS KQSLSYLDGS LPGDFGFDPL GLSDPEGTGG FIEPRWLAYG**
101 **EIINGRYAML GAVGAIAPEI LGK**AGLIPQE TALPWFQTGV IPPAGTYNYW
151 ADPFTLFVFE MALMGFAEHR **RLQDWYNPGS MGKQYFLGLE KGFAGSGEPA**
201 **YPGGPIFNPL GFGKDEK**SLK ELKLKEVKNG RLAMLAILGY FIQGLVTGVG
251 PYQNLLDHLA DPVNNNILTS LKFH

Unformatted sequence string: **274 residues** (for pasting into other applications).

Sort peptides by ● Residue Number ○ Increasing Mass ○ Decreasing Mass

Show predicted peptides also

Query	Start – End	Observed	Mr(expt)	Mr(calc)	ppm	M	Score	Expect	Rank	U	Peptide
17019	50 – 61	477.9211	1430.7415	1430.7419	-0.26	1	14	0.052	1	U	K.QNANRPLWFASK.Q
17020	50 – 61	716.3783	1430.7421	1430.7419	0.19	1	20	0.012	1	U	K.QNANRPLWFASK.Q
17022	50 – 61	716.3790	1430.7434	1430.7419	1.12	1	28	0.0022	1	U	K.QNANRPLWFASK.Q
17024	50 – 61	477.9221	1430.7443	1430.7419	1.73	1	10	0.11	1	U	K.QNANRPLWFASK.Q
2699	55 – 61	424.7377	847.4608	847.4592	1.82	0	4	0.4	1	U	R.PLWFASK.Q
29904	62 – 95	1180.9011	3539.6814	3539.6733	2.30	0	70	2.6e-07	1	U	K.QSLSYLDGSLPGDFGFDPLGLSDPEGTGGFIEPR.W
13661	96 – 106	431.2313	1290.6721	1290.6720	0.011	0	26	0.0039	1	U	R.WLAYGEIINGR.Y
13662	96 – 106	646.3434	1290.6723	1290.6720	0.16	0	41	0.00013	1	U	R.WLAYGEIINGR.Y
29324	96 – 123	982.8704	2945.5895	2945.5837	1.98	1	14	0.17	1	U	R.WLAYGEIINGRYAMLGAVGAIAPEILGK.A
21473	107 – 123	837.4688	1672.9230	1672.9222	0.46	0	74	1.1e-07	1	U	R.YAMLGAVGAIAPEILGK.A
21474	107 – 123	558.6486	1672.9240	1672.9222	1.10	0	34	0.00066	1	U	R.YAMLGAVGAIAPEILGK.A
21475	107 – 123	837.4701	1672.9257	1672.9222	2.09	0	13	0.062	1	U	R.YAMLGAVGAIAPEILGK.A
21698	107 – 123	563.9779	1688.9119	1688.9171	-3.06	0	29	0.0019	1	U	R.YAMLGAVGAIAPEILGK.A + Oxidation (M)
21700	107 – 123	845.4679	1688.9212	1688.9171	2.41	0	84	1.4e-08	1	U	R.YAMLGAVGAIAPEILGK.A + Oxidation (M)
19541	171 – 183	776.3715	1550.7285	1550.7300	-0.98	1	50	1.9e-05	1	U	R.RLQDWYNPGSMGK.Q
19542	171 – 183	517.9170	1550.7292	1550.7300	-0.52	1	10	0.11	1	U	R.RLQDWYNPGSMGK.Q
19543	171 – 183	776.3722	1550.7299	1550.7300	-0.048	1	32	0.001	1	U	R.RLQDWYNPGSMGK.Q
19545	171 – 183	517.9176	1550.7309	1550.7300	0.58	1	35	0.00057	1	U	R.RLQDWYNPGSMGK.Q
19795	171 – 183	784.3695	1566.7244	1566.7249	-0.32	1	42	0.00012	1	U	R.RLQDWYNPGSMGK.Q + Oxidation (M)
19797	171 – 183	523.2489	1566.7250	1566.7249	0.048	1	31	0.0012	1	U	R.RLQDWYNPGSMGK.Q + Oxidation (M)
19803	171 – 183	784.3735	1566.7325	1566.7249	4.83	1	22	0.0091	1	U	R.RLQDWYNPGSMGK.Q + Oxidation (M)
16144	172 – 183	698.3213	1394.6281	1394.6289	-0.56	0	47	9.6e-05	1	U	R.LQDWYNPGSMGK.Q
16146	172 – 183	698.3216	1394.6286	1394.6289	-0.17	0	53	2.4e-05	1	U	R.LQDWYNPGSMGK.Q
16148	172 – 183	698.3244	1394.6342	1394.6289	3.85	0	4	0.38	1	U	R.LQDWYNPGSMGK.Q
16520	172 – 183	706.3194	1410.6242	1410.6238	0.26	0	38	0.00064	1	U	R.LQDWYNPGSMGK.Q + Oxidation (M)
16521	172 – 183	706.3197	1410.6249	1410.6238	0.80	0	47	8.7e-05	1	U	R.LQDWYNPGSMGK.Q + Oxidation (M)
16523	172 – 183	471.2156	1410.6251	1410.6238	0.93	0	12	0.27	1	U	R.LQDWYNPGSMGK.Q + Oxidation (M)
27560	172 – 191	797.3864	2389.1374	2389.1413	-1.60	1	12	0.081	1	U	R.LQDWYNPGSMGKQYFLGLEK.G + Oxidation (M)
5853	184 – 191	499.2718	996.5290	996.5280	0.94	0	5	0.32	1	U	K.QYFLGLEK.G
5856	184 – 191	499.2723	996.5301	996.5280	2.07	0	17	0.026	1	U	K.QYFLGLEK.G
5857	184 – 191	499.2723	996.5301	996.5280	2.07	0	3	0.5	1	U	K.QYFLGLEK.G
26657	192 – 214	746.3722	2236.0949	2236.0953	-0.21	0	63	1.1e-06	1	U	K.GFAGSGEPAYPGGPIFNPLGFGK.D
28415	192 – 217	870.4267	2608.2583	2608.2598	-0.58	1	43	9.3e-05	1	U	K.GFAGSGEPAYPGGPIFNPLGFGKDEK.S
28416	192 – 217	653.0726	2608.2612	2608.2598	0.55	1	11	0.095	1	U	K.GFAGSGEPAYPGGPIFNPLGFGKDEK.S
28417	192 – 217	870.4278	2608.2616	2608.2598	0.68	1	51	1.7e-05	1	U	K.GFAGSGEPAYPGGPIFNPLGFGKDEK.S
28418	192 – 217	1305.1392	2608.2638	2608.2598	1.54	1	64	9.8e-07	1	U	K.GFAGSGEPAYPGGPIFNPLGFGKDEK.S

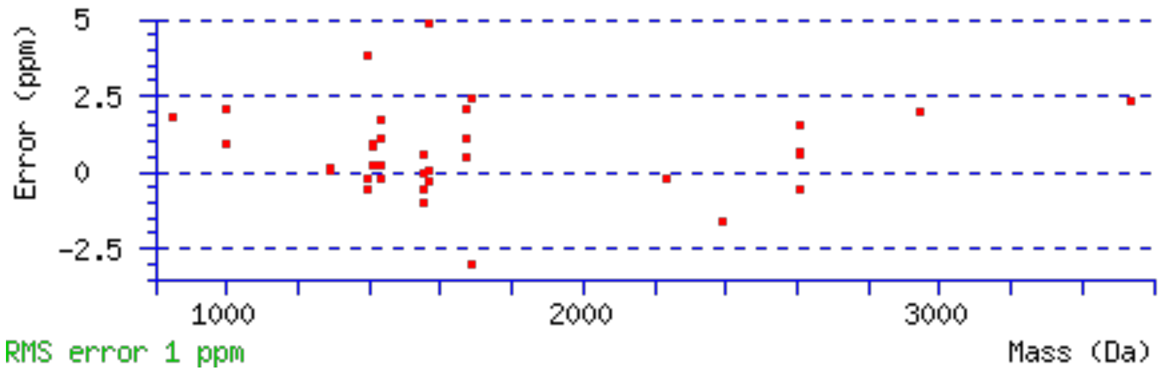

Mascot: http://www.matrixscience.com/

MASCOT Search Results

Protein View: tr|A0A0K9RRT4|A0A0K9RRT4_SPIOL

>tr|A0A0K9RRT4|A0A0K9RRT4_SPIOL Chlorophyll a-b binding protein, chloroplastic OS=Spinacia oleracea OX=3562 GN=SOVF_041770 PE=3 SV=1

**Database:**	Uni-Spinach
**Score:**	664
**Nominal mass (M_r):**	26370
**Calculated pI:**	5.97

Sequence similarity is available as **an NCBI BLAST search of tr|A0A0K9RRT4|A0A0K9RRT4_SPIOL against nr**.

Search parameters

**MS data file:**	\\128.97.66.218\tank\windowsVM\Bill Cramer\MGF\wc_QE_021417_Cramer_Gel_1S_4a.mgf
**Enzyme:**	Trypsin/P: cuts C-term side of KR.
**Fixed modifications:**	Carbamidomethyl (C)
**Variable modifications:**	Oxidation (M)

Protein sequence coverage: 30%

Matched peptides shown in ***bold red***.

```
  1 MASNALMSCG IAAVFPSSLS SSKSKFAASV PLGNVSCNAS SRFTMSAEWM
 51 PGQPRPAHLD GSAPGDFGFD PLGLGEVPEN LERFKESELI HCRWAMLAVP
101 GILVPEALGL GNWVKAQEWA ALPGGQATYL GNPVPWGNLP TILAIEFLAI
151 AFVEHQRSME KDSEKKKYPG GAFDPLGYSK DPKKFEELKL KEIKNGRLAL
201 LAFVGFCIQQ SAYPGTGPLE NLATHLADPW HNNIGDIVIP RAL
```

Unformatted sequence string: **243 residues** (for pasting into other applications).

Sort peptides by ◉ Residue Number ○ Increasing Mass ○ Decreasing Mass

Show predicted peptides also

Query	Start – End	Observed	Mr(expt)	Mr(calc)	ppm	M	Score	Expect	Rank	U	Peptide
19576	43 – 55	777.3475	1552.6804	1552.6803	0.082	0	22	0.018	1	U	R.FTMSAEWMPGQPR.P + Oxidation (M)
19577	43 – 55	777.3508	1552.6871	1552.6803	4.42	0	10	0.33	1	U	R.FTMSAEWMPGQPR.P + Oxidation (M)
19578	43 – 55	777.3510	1552.6874	1552.6803	4.57	0	37	0.0006	1	U	R.FTMSAEWMPGQPR.P + Oxidation (M)
19831	43 – 55	785.3464	1568.6783	1568.6752	2.00	0	1	2.2	1	U	R.FTMSAEWMPGQPR.P + 2 Oxidation (M)
29244	56 – 83	969.4677	2905.3811	2905.3883	-2.45	0	73	1.4e-07	1	U	R.PAHLDGSAPGDFGFDPLGLGEVPENLER.F
29245	56 – 83	969.4703	2905.3892	2905.3883	0.32	0	86	8.3e-09	1	U	R.PAHLDGSAPGDFGFDPLGLGEVPENLER.F
29246	56 – 83	1453.7034	2905.3923	2905.3883	1.41	0	116	1.2e-11	1	U	R.PAHLDGSAPGDFGFDPLGLGEVPENLER.F
29247	56 – 83	727.3558	2905.3941	2905.3883	1.99	0	67	5.3e-07	1	U	R.PAHLDGSAPGDFGFDPLGLGEVPENLER.F
14328	84 – 93	440.2234	1317.6484	1317.6499	-1.17	1	22	0.0091	1	U	R.FKESELIHCR.W
14329	84 – 93	659.8317	1317.6488	1317.6499	-0.89	1	42	0.00011	1	U	R.FKESELIHCR.W
14330	84 – 93	440.2243	1317.6510	1317.6499	0.83	1	15	0.042	1	U	R.FKESELIHCR.W
14331	84 – 93	659.8333	1317.6520	1317.6499	1.60	1	21	0.017	1	U	R.FKESELIHCR.W
14332	84 – 93	440.2249	1317.6528	1317.6499	2.14	1	40	0.00018	1	U	R.FKESELIHCR.W
6784	86 – 93	522.2498	1042.4851	1042.4866	-1.38	0	21	0.03	1	U	K.ESELIHCR.W
6785	86 – 93	522.2506	1042.4866	1042.4866	0.076	0	20	0.031	1	U	K.ESELIHCR.W
20773	166 – 180	814.4268	1626.8390	1626.8406	-0.95	2	32	0.001	1	U	K.KKYPGGAFDPLGYSK.D
20774	166 – 180	543.2876	1626.8408	1626.8406	0.15	2	24	0.006	1	U	K.KKYPGGAFDPLGYSK.D
20775	166 – 180	543.2876	1626.8408	1626.8406	0.16	2	14	0.053	1	U	K.KKYPGGAFDPLGYSK.D
20776	166 – 180	814.4281	1626.8417	1626.8406	0.70	2	37	0.00033	1	U	K.KKYPGGAFDPLGYSK.D
20777	166 – 180	407.7177	1626.8419	1626.8406	0.81	2	2	0.7	1	U	K.KKYPGGAFDPLGYSK.D
20778	166 – 180	543.2888	1626.8445	1626.8406	2.40	2	33	0.00078	1	U	K.KKYPGGAFDPLGYSK.D
20779	166 – 180	543.2889	1626.8447	1626.8406	2.55	2	5	0.35	1	U	K.KKYPGGAFDPLGYSK.D
18530	167 – 180	750.3799	1498.7453	1498.7456	-0.22	1	66	7e-07	1	U	K.KYPGGAFDPLGYSK.D
18531	167 – 180	750.3800	1498.7454	1498.7456	-0.17	1	50	2e-05	1	U	K.KYPGGAFDPLGYSK.D
18532	167 – 180	500.5892	1498.7458	1498.7456	0.11	1	26	0.0035	1	U	K.KYPGGAFDPLGYSK.D
18533	167 – 180	500.5897	1498.7474	1498.7456	1.20	1	25	0.0046	1	U	K.KYPGGAFDPLGYSK.D
18534	167 – 180	750.3810	1498.7475	1498.7456	1.23	1	2	0.65	1	U	K.KYPGGAFDPLGYSK.D
18536	167 – 180	500.5906	1498.7501	1498.7456	2.96	1	33	0.00081	1	U	K.KYPGGAFDPLGYSK.D
23583	167 – 183	920.4655	1838.9164	1838.9203	-2.11	2	24	0.0056	1	U	K.KYPGGAFDPLGYSKDPK.K
23585	167 – 183	613.9810	1838.9212	1838.9203	0.51	2	12	0.08	1	U	K.KYPGGAFDPLGYSKDPK.K
23586	167 – 183	613.9813	1838.9221	1838.9203	1.00	2	9	0.14	1	U	K.KYPGGAFDPLGYSKDPK.K

15537	168 - 180	686.3344	1370.6542	1370.6507	2.61	0	54	9.3e-06	1	U	K.YPGGAFDPLGYSK.D
15538	168 - 180	686.3347	1370.6547	1370.6507	2.98	0	58	3.8e-06	1	U	K.YPGGAFDPLGYSK.D
21995	168 - 183	571.2823	1710.8250	1710.8253	-0.19	1	10	0.11	1	U	K.YPGGAFDPLGYSKDPK.K
21996	168 - 183	856.4205	1710.8264	1710.8253	0.60	1	11	0.098	1	U	K.YPGGAFDPLGYSKDPK.K
1520	184 - 189	397.2263	792.4380	792.4381	-0.22	1	5	0.31	1	U	K.KFEELK.L

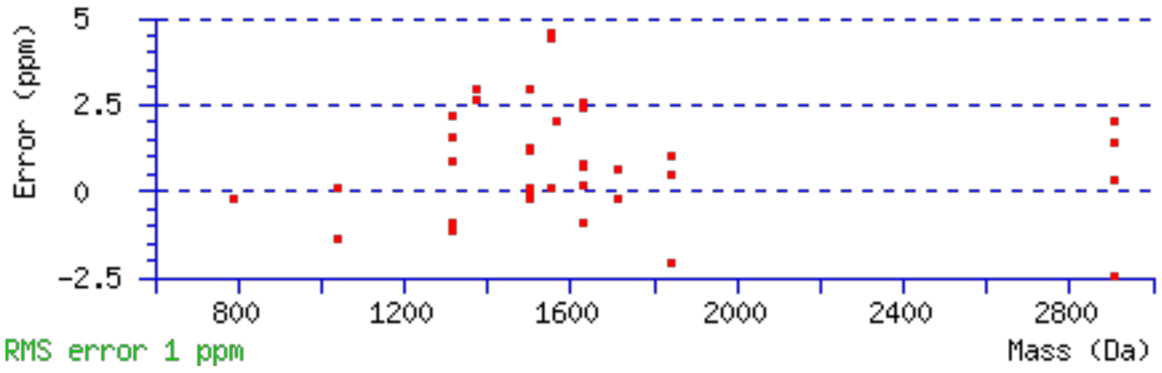

Mascot: http://www.matrixscience.com/

MASCOT Search Results

Protein View: tr|A0A0K9QGT7|A0A0K9QGT7_SPIOL

>tr|A0A0K9QGT7|A0A0K9QGT7_SPIOL Ferredoxin--NADP reductase, chloroplastic OS=Spinacia oleracea OX=3562 GN=SOVF_180280 PE=3 SV=1

**Database:**	Uni-Spinach
**Score:**	652
**Nominal mass (M_r):**	41399
**Calculated pI:**	8.67

Sequence similarity is available as an NCBI BLAST search of tr|A0A0K9QGT7|A0A0K9QGT7_SPIOL against nr.

Search parameters

**MS data file:**	\\128.97.66.218\tank\windowsVM\Bill Cramer\MGF\wc_QE_021417_Cramer_Gel_1S_4a.mgf
**Enzyme:**	Trypsin/P: cuts C-term side of KR.
**Fixed modifications:**	Carbamidomethyl (C)
**Variable modifications:**	Oxidation (M)

Protein sequence coverage: 48%

Matched peptides shown in ***bold red***.

**1**	MTTAVTAAVS	FPSTKTTSLS	ARSSSVISPD	KISYKKVPLY	YRNVSATGKM
**51**	GPIRAQIASD	VEAPPPAPAK	VEKHSK**KMEE**	**GITVNK**FKPK	TPYVGR**CLLN**
**101**	**TKITGDDAPG**	**ETWHMVFSHE**	**GEIPYREGQS**	**VGVIPDGEDK**	NGKPHKLR**LY**
**151**	**SIASSALGDF**	**GDAK**SVSLCV	K**RLIYTNDAG**	**ETIKGVCSNF**	**LCDLKPGAEV**
**201**	**K**LTGPVGK**EM**	**LMPKDPNATI**	**IMLGTGTGIA**	**PFR**SFLWKMF	FEKHDDYKFN
**251**	GLAWLFLGVP	TSSSLLYKEE	FEKMKEK**APD**	**NFRLDFAVSR**	EQTNEKGEK**M**
**301**	**YIQTRMAQYA**	**VELWEMLKKD**	**NTYVYMCGLK**	GMEKGIDDIM	VSLAAAEGID
**351**	WIEYKRQLK**K**	**AEQWNVEVY**			

Unformatted sequence string: 369 residues (for pasting into other applications).

Sort peptides by ◉ Residue Number ○ Increasing Mass ○ Decreasing Mass

Show predicted peptides also

Query	Start – End	Observed	Mr(expt)	Mr(calc)	ppm	M	Score	Expect	Rank	U	Peptide
9729	77 – 86	383.5376	1147.5908	1147.5907	0.13	1	12	0.068	1	U	K.KMEEGITVNK.F
10196	77 – 86	388.8691	1163.5855	1163.5856	-0.068	1	1	0.72	1	U	K.KMEEGITVNK.F + Oxidation (M)
696	97 – 102	374.7050	747.3955	747.3949	0.76	0	4	0.38	1	U	R.CLLNTK.I
28928	103 – 126	915.4185	2743.2338	2743.2337	0.047	0	52	2.6e-05	1	U	K.ITGDDAPGETWHMVFSHEGEIPYR.E
28929	103 – 126	686.8164	2743.2364	2743.2337	1.01	0	52	2.8e-05	1	U	K.ITGDDAPGETWHMVFSHEGEIPYR.E
28975	103 – 126	920.7496	2759.2269	2759.2286	-0.60	0	41	0.00028	1	U	K.ITGDDAPGETWHMVFSHEGEIPYR.E + Oxidation (M)
28976	103 – 126	920.7501	2759.2286	2759.2286	-0.0098	0	21	0.026	1	U	K.ITGDDAPGETWHMVFSHEGEIPYR.E + Oxidation (M)
16965	127 – 140	715.3443	1428.6740	1428.6733	0.52	0	33	0.00077	1	U	R.EGQSVGVIPDGEDK.N
20560	149 – 164	807.9057	1613.7969	1613.7937	1.98	0	69	3.5e-07	1	U	R.LYSIASSALGDFGDAK.S
20561	149 – 164	538.9404	1613.7994	1613.7937	3.52	0	28	0.0024	1	U	R.LYSIASSALGDFGDAK.S
18418	172 – 184	747.4005	1492.7864	1492.7885	-1.39	1	18	0.042	1	U	K.RLIYTNDAGETIK.G
18419	172 – 184	498.6029	1492.7869	1492.7885	-1.11	1	9	0.14	1	U	K.RLIYTNDAGETIK.G
14724	173 – 184	669.3516	1336.6887	1336.6874	0.99	0	52	1.2e-05	1	U	R.LIYTNDAGETIK.G
24143	185 – 201	631.9782	1892.9128	1892.9125	0.16	1	41	0.00014	1	U	K.GVCSNFLCDLKPGAEVK.L
24144	185 – 201	947.4642	1892.9138	1892.9125	0.71	1	20	0.014	1	U	K.GVCSNFLCDLKPGAEVK.L
975	209 – 214	382.6879	763.3613	763.3608	0.62	0	2	0.65	1	U	K.EMLMPK.D + Oxidation (M)
24618	215 – 233	973.0141	1944.0136	1944.0139	-0.14	0	105	1.6e-10	1	U	K.DPNATIIMLGTGTGIAPFR.S
24619	215 – 233	649.0121	1944.0145	1944.0139	0.33	0	64	1.1e-06	1	U	K.DPNATIIMLGTGTGIAPFR.S
24796	215 – 233	981.0129	1960.0113	1960.0088	1.29	0	99	5e-10	1	U	K.DPNATIIMLGTGTGIAPFR.S + Oxidation (M)
24797	215 – 233	654.3453	1960.0140	1960.0088	2.67	0	43	8.7e-05	1	U	K.DPNATIIMLGTGTGIAPFR.S + Oxidation (M)
264	278 – 283	360.1768	718.3390	718.3398	-1.12	0	7	0.3	1	U	K.APDNFR.L
266	278 – 283	360.1774	718.3402	718.3398	0.58	0	8	0.19	1	U	K.APDNFR.L
1789	284 – 290	404.2219	806.4292	806.4286	0.66	0	10	0.12	1	U	R.LDFAVSR.E
1867	300 – 305	406.2103	810.4060	810.4058	0.19	0	13	0.057	1	U	K.MYIQTR.M
2183	300 – 305	414.2071	826.3996	826.4007	-1.34	0	17	0.072	1	U	K.MYIQTR.M + Oxidation (M)

20521	306 - 318	806.4007	1610.7869	1610.7836	2.01	0	62	1.4e-06	1	U	R.MAQYAVELWEMLK.K
20522	306 - 318	537.9363	1610.7869	1610.7836	2.06	0	37	0.00038	1	U	R.MAQYAVELWEMLK.K
20771	306 - 318	814.3979	1626.7813	1626.7785	1.68	0	53	1.1e-05	1	U	R.MAQYAVELWEMLK.K + Oxidation (M)
22590	306 - 319	585.9660	1754.8762	1754.8735	1.54	1	20	0.013	1	U	R.MAQYAVELWEMLKK.D + Oxidation (M)
18357	319 - 330	497.9041	1490.6905	1490.6898	0.48	1	22	0.0086	1	U	K.KDNTYVYMCGLK.G
18359	319 - 330	746.3532	1490.6918	1490.6898	1.40	1	23	0.0071	1	U	K.KDNTYVYMCGLK.G
15361	320 - 330	682.3044	1362.5943	1362.5948	-0.34	0	24	0.0091	1	U	K.DNTYVYMCGLK.G
15751	320 - 330	690.3031	1378.5916	1378.5897	1.40	0	9	0.27	1	U	K.DNTYVYMCGLK.G + Oxidation (M)
12999	360 - 369	633.3132	1264.6118	1264.6088	2.39	1	27	0.0033	1	U	K.KAEQWNVEVY.-
9408	361 - 369	569.2656	1136.5166	1136.5138	2.47	0	8	0.16	1	U	K.AEQWNVEVY.-

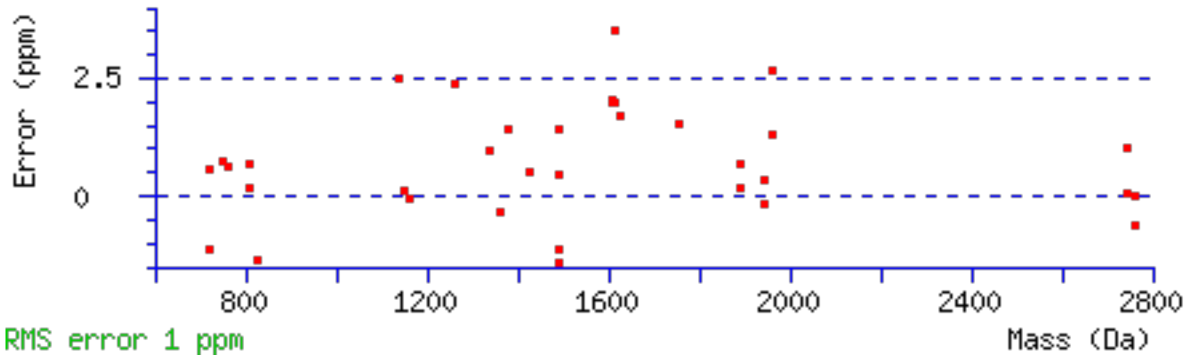

Mascot: http://www.matrixscience.com/

MASCOT Search Results

Protein View: tr|A0A0K9QPJ7|A0A0K9QPJ7_SPIOL

>tr|A0A0K9QPJ7|A0A0K9QPJ7_SPIOL Uncharacterized protein OS=Spinacia oleracea OX=3562 GN=SOVF_155930 PE=3 SV=1

**Database:**	Uni-Spinach
**Score:**	634
**Nominal mass (M_r):**	40391
**Calculated pI:**	5.95

Sequence similarity is available as an NCBI BLAST search of tr|A0A0K9QPJ7|A0A0K9QPJ7_SPIOL against nr.

Search parameters

**MS data file:**	\\128.97.66.218\tank\windowsVM\Bill Cramer\MGF\wc_QE_021417_Cramer_Gel_1S_4a.mgf
**Enzyme:**	Trypsin/P: cuts C-term side of KR.
**Fixed modifications:**	Carbamidomethyl (C)
**Variable modifications:**	Oxidation (M)

Protein sequence coverage: 38%

Matched peptides shown in ***bold red***.

```
  1 MACSLSFSSS VSTFHLPTTT QSTQAPPNNA TTLPTTNPIQ CANLRELRDR
 51 IGSVKNTQKI TEAMKLVAAA KVRRAQEAVV NGRPFSETLV EVLYNMNEQL
101 QTEDVDVPLT KIRTVKKVAL MVVTGDRGLC GGFNNMLLKK AESRIAELKK
151 LGVDYTIISI GKKGNTYFIR RPEIPVDRYF DGTNLPTAKE AQAIADDVFS
201 LFVSEEVDKV EMLYTKFVSL VKSDPVIHTL LPLSPKGEIC DINGKCVDAA
251 EDELFRLTTK EGKLTVERDM IKTETPAFSP ILEFEQDPAQ ILDALLPLYL
301 NSQILRALQE SLASELAARM TAMSNATDNA NELKKTLSIN YNRARQAKIT
351 GEILEIVAGA NACV
```

Unformatted sequence string: 364 residues (for pasting into other applications).

Sort peptides by ◉ Residue Number ○ Increasing Mass ○ Decreasing Mass

Show predicted peptides also

Query	Start – End	Observed	Mr(expt)	Mr(calc)	ppm	M	Score	Expect	Rank	U	Peptide
10869	117 – 127	594.8418	1187.6691	1187.6696	-0.46	1	42	0.00036	1	U	K.KVALMVVTGDR.G
10870	117 – 127	396.8972	1187.6699	1187.6696	0.19	1	31	0.0012	1	U	K.KVALMVVTGDR.G
11346	117 – 127	402.2282	1203.6627	1203.6646	-1.56	1	31	0.0039	1	U	K.KVALMVVTGDR.G + Oxidation (M)
11347	117 – 127	402.2282	1203.6627	1203.6646	-1.53	1	12	0.069	1	U	K.KVALMVVTGDR.G + Oxidation (M)
11348	117 – 127	602.8392	1203.6638	1203.6646	-0.58	1	35	0.00054	1	U	K.KVALMVVTGDR.G + Oxidation (M)
11349	117 – 127	602.8397	1203.6648	1203.6646	0.20	1	2	0.72	1	U	K.KVALMVVTGDR.G + Oxidation (M)
7247	118 – 127	530.7944	1059.5743	1059.5747	-0.34	0	21	0.01	1	U	K.VALMVVTGDR.G
7680	118 – 127	538.7920	1075.5695	1075.5696	-0.053	0	30	0.0015	1	U	K.VALMVVTGDR.G + Oxidation (M)
7681	118 – 127	538.7935	1075.5725	1075.5696	2.72	0	22	0.009	1	U	K.VALMVVTGDR.G + Oxidation (M)
14439	128 – 139	662.3338	1322.6530	1322.6475	4.18	0	36	0.00042	1	U	R.GLCGGFNNMLLK.K
14758	128 – 139	670.3292	1338.6438	1338.6424	1.06	0	31	0.0012	1	U	R.GLCGGFNNMLLK.K + Oxidation (M)
16414	150 – 162	469.6127	1405.8162	1405.8181	-1.29	1	34	0.0016	1	U	K.KLGVDYTIISIGK.K
16415	150 – 162	703.9155	1405.8164	1405.8181	-1.16	1	62	2.6e-06	1	U	K.KLGVDYTIISIGK.K
19247	150 – 163	512.3116	1533.9129	1533.9130	-0.050	2	37	0.00022	1	U	K.KLGVDYTIISIGKK.G
13345	151 – 162	639.8693	1277.7241	1277.7231	0.76	0	79	4.3e-08	1	U	K.LGVDYTIISIGK.K
16416	151 – 163	703.9166	1405.8187	1405.8181	0.46	1	20	0.027	1	U	K.LGVDYTIISIGKK.G
5872	163 – 170	499.7748	997.5351	997.5345	0.63	1	0	4.5	1	U	K.KGNTYFIR.R
3144	164 – 170	435.7267	869.4389	869.4395	-0.74	0	11	0.097	1	U	K.GNTYFIR.R

5529	171 - 178	491.2775	980.5405	980.5403	0.21	1	21	0.012	1	U	R.RPEIPVDR.Y
11936	179 - 189	409.5397	1225.5973	1225.5979	-0.51	0	3	0.55	1	U	R.YFDGTNLPTAK.E
11937	179 - 189	613.8061	1225.5977	1225.5979	-0.13	0	26	0.0036	1	U	R.YFDGTNLPTAK.E
18881	223 - 236	758.9410	1515.8674	1515.8661	0.89	0	50	2e-05	1	U	K.SDPVIHTLLPLSPK.G
18883	223 - 236	506.2965	1515.8677	1515.8661	1.05	0	40	0.00017	1	U	K.SDPVIHTLLPLSPK.G
14460	246 - 256	662.7969	1323.5792	1323.5765	2.00	0	67	6.5e-07	1	U	K.CVDAAEDELFR.L
15234	307 - 319	679.8662	1357.7178	1357.7201	-1.71	0	71	2e-07	1	U	R.ALQESLASELAAR.M
15235	307 - 319	453.5800	1357.7180	1357.7201	-1.52	0	43	9.1e-05	1	U	R.ALQESLASELAAR.M
15240	307 - 319	679.8687	1357.7228	1357.7201	1.99	0	9	0.2	1	U	R.ALQESLASELAAR.M
20512	320 - 334	805.8618	1609.7091	1609.7076	0.98	0	6	0.92	1	U	R.MTAMSNATDNANELK.K
22391	320 - 335	580.2761	1737.8065	1737.8025	2.27	1	63	1.2e-06	1	U	R.MTAMSNATDNANELKK.T
22571	320 - 335	585.6060	1753.7961	1753.7974	-0.79	1	50	2.3e-05	1	U	R.MTAMSNATDNANELKK.T + Oxidation (M)
22572	320 - 335	877.9055	1753.7964	1753.7974	-0.57	1	3	2.7	1	U	R.MTAMSNATDNANELKK.T + Oxidation (M)
22574	320 - 335	585.6068	1753.7985	1753.7974	0.62	1	5	0.38	1	U	R.MTAMSNATDNANELKK.T + Oxidation (M)
22777	320 - 335	590.9379	1769.7919	1769.7924	-0.27	1	29	0.0059	1	U	R.MTAMSNATDNANELKK.T + 2 Oxidation (M)
22779	320 - 335	590.9390	1769.7952	1769.7924	1.63	1	38	0.0008	1	U	R.MTAMSNATDNANELKK.T + 2 Oxidation (M)
5512	336 - 343	490.7614	979.5083	979.5087	-0.36	0	3	0.51	1	U	K.TLSINYNR.A
5513	336 - 343	490.7618	979.5090	979.5087	0.31	0	5	0.5	1	U	K.TLSINYNR.A
20808	349 - 364	815.4301	1628.8457	1628.8443	0.84	0	20	0.013	1	U	K.ITGEILEIVAGANACV.-
20809	349 - 364	543.9563	1628.8472	1628.8443	1.74	0	20	0.015	1	U	K.ITGEILEIVAGANACV.-

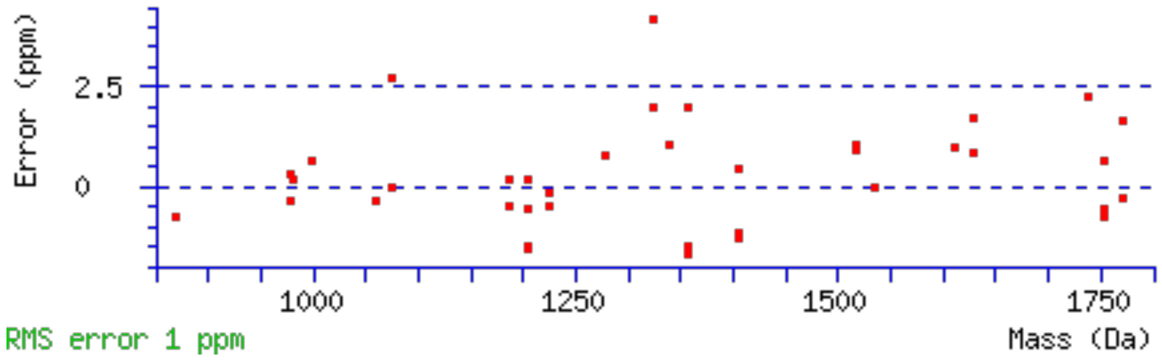

Mascot: http://www.matrixscience.com/

MASCOT Search Results

Protein View: tr|A0A0K9RD58|A0A0K9RD58_SPIOL

>tr|A0A0K9RD58|A0A0K9RD58_SPIOL AAA domain-containing protein OS=Spinacia oleracea OX=3562 GN=SOVF_080200 PE=3 SV=1

**Database:**	Uni-Spinach
**Score:**	613
**Nominal mass (M_r):**	73817
**Calculated pI:**	5.70

Sequence similarity is available as an NCBI BLAST search of tr|A0A0K9RD58|A0A0K9RD58_SPIOL against nr.

Search parameters

**MS data file:**	\\128.97.66.218\tank\windowsVM\Bill Cramer\MGF\wc_QE_021417_Cramer_Gel_1S_4a.mgf
**Enzyme:**	Trypsin/P: cuts C-term side of KR.
**Fixed modifications:**	Carbamidomethyl (C)
**Variable modifications:**	Oxidation (M)

Protein sequence coverage: 33%

Matched peptides shown in ***bold red***.

```
  1 MAASSACLLG HGSSTSSSKT ILKERLFHGH LFAGRSVLLK SQKAFIVKAS
 51 SDKAQSDGRR GFLKLLLGNA GVASTLVASG NANADEQGVS SSRMSYSRFL
101 EYLDKDRVQK VDLFENGTIA IVEAISPELG NRVQRVRVQL PGLSQELLQK
151 LREKNIDFAA HNAQEDSGSV LFNLIGNLAF PLILIGGLFL LSRRSGGGMG
201 GPGGPGNPLA FGQSKAKFQM EPNTGVTFDD VAGVDEAKQD FMEVVEFLKK
251 PERFTAVGAR IPKGVLLVGP PGTGKTLLAK AIAGEAGVPF FSISGSEFVE
301 MFVGVGASRV RDLFKKAKEN APCIVFVDEI DAVGRSRGTG IGGGNDEREQ
351 TLNQLLTEMD GFEGNTGIIV VAATNRADIL DSALLRPGRF DRQVSVDVPD
401 VRGRTDILKV HASNKKFDGD VSLEVIAMRT PGFSGADLAN LLNEAAILAG
451 RRGKTAICSK EIDDSIDRIV AGMEGTVMTD SKSKSLVAYH EVGHAICGTL
501 TPGHDAVQKV TLVPRGQARG LTWFIPSDDP TLISKQQLFA RIVGGLGGRA
551 AEEIIFGESE VTTGAAGDLQ QITGLAKQMV TTFGMSDIGP WSLMDASAQS
601 GDVIMRMMAR NSMSERLAED IDNAVKKITD EAYQIALTHI RNNREAIDKI
651 VDVLLETETV SGDEFRTLLA EFTEIPVENR VPAAAASPVT V
```

Unformatted sequence string: 691 residues (for pasting into other applications).

Sort peptides by ◉ Residue Number ○ Increasing Mass ○ Decreasing Mass

Show predicted peptides also

Query	Start – End	Observed	Mr(expt)	Mr(calc)	ppm	M	Score	Expect	Rank	U	Peptide
11161	99 – 107	400.2081	1197.6023	1197.6030	-0.52	1	27	0.0031	1	U	R.FLEYLDKDR.V
11162	99 – 107	599.8089	1197.6033	1197.6030	0.24	1	39	0.00021	1	U	R.FLEYLDKDR.V
17543	138 – 150	726.9242	1451.8339	1451.8348	-0.62	0	29	0.0049	1	U	R.VQLPGLSQELLQK.L
17544	138 – 150	484.9529	1451.8369	1451.8348	1.48	0	5	1.2	1	U	R.VQLPGLSQELLQK.L
23457	195 – 215	915.4342	1828.8538	1828.8527	0.65	0	26	0.004	1	U	R.SGGGMGGPGGPGNPLAFGQSK.A
23458	195 – 215	610.6258	1828.8556	1828.8527	1.59	0	18	0.019	1	U	R.SGGGMGGPGGPGNPLAFGQSK.A
301	254 – 260	361.2031	720.3916	720.3919	-0.32	0	3	0.48	1	U	R.FTAVGAR.I
8241	264 – 275	547.8326	1093.6507	1093.6496	1.06	0	20	0.023	1	U	K.GVLLVGPPGTGK.T
8242	264 – 275	547.8327	1093.6508	1093.6496	1.14	0	24	0.0099	1	U	K.GVLLVGPPGTGK.T
16182	377 – 389	466.2685	1395.7836	1395.7834	0.17	1	9	0.59	1	U	R.ADILDSALLRPGR.F
8734	393 – 402	557.2979	1112.5813	1112.5826	-1.22	0	19	0.015	1	U	R.QVSVDVPDVR.G
20017	416 – 429	790.4101	1578.8057	1578.8076	-1.21	1	67	5.8e-07	1	U	K.KFDGDVSLEVIAMR.T
20018	416 – 429	527.2765	1578.8076	1578.8076	0.018	1	46	5.1e-05	1	U	K.KFDGDVSLEVIAMR.T
20279	416 – 429	532.6090	1594.8051	1594.8025	1.65	1	31	0.0012	1	U	K.KFDGDVSLEVIAMR.T + Oxidation (M)
5129	461 – 468	481.7247	961.4349	961.4352	-0.36	0	3	2.5	1	U	K.EIDDSIDR.I
17196	469 – 482	719.8468	1437.6790	1437.6844	-3.75	0	31	0.0012	1	U	R.IVAGMEGTVMTDSK.S
17580	469 – 482	727.8475	1453.6805	1453.6793	0.82	0	45	5.6e-05	1	U	R.IVAGMEGTVMTDSK.S + Oxidation (M)
17931	469 – 482	735.8436	1469.6726	1469.6742	-1.05	0	32	0.0026	1	U	R.IVAGMEGTVMTDSK.S + 2 Oxidation (M)
28610	485 – 509	665.8372	2659.3199	2659.3177	0.82	0	28	0.0026	1	U	K.SLVAYHEVGHAICGTLTPGHDAVQK.V
22989	520 – 535	895.4724	1788.9302	1788.9298	0.21	0	28	0.0022	1	U	R.GLTWFIPSDDPTLISK.Q

932	536 - 541	381.7157	761.4168	761.4184	-2.05	0	2	0.61	1	U	K.QQLFAR.I
11636	617 - 627	608.3320	1214.6494	1214.6506	-1.00	1	4	0.4	1	U	R.LAEDIDNAVKK.I
22801	627 - 641	443.7496	1770.9692	1770.9628	3.62	1	25	0.0045	1	U	K.KITDEAYQIALTHIR.N
22802	627 - 641	591.3313	1770.9722	1770.9628	5.29	1	57	4.2e-06	1	U	K.KITDEAYQIALTHIR.N
21028	628 - 641	822.4413	1642.8680	1642.8678	0.095	0	46	4.8e-05	1	U	K.ITDEAYQIALTHIR.N
21029	628 - 641	548.6300	1642.8681	1642.8678	0.15	0	41	0.00015	1	U	K.ITDEAYQIALTHIR.N
27896	645 - 666	826.7605	2477.2598	2477.2537	2.44	1	61	2e-06	1	U	R.EAIDKIVDVLLETETVSGDEFR.T
24400	650 - 666	961.4915	1920.9684	1920.9680	0.18	0	110	4.5e-11	1	U	K.IVDVLLETETVSGDEFR.T
20829	667 - 680	544.6273	1630.8601	1630.8566	2.16	0	43	9.5e-05	1	U	R.TLLAEFTEIPVENR.V
20830	667 - 680	816.4374	1630.8603	1630.8566	2.24	0	76	7.8e-08	1	U	R.TLLAEFTEIPVENR.V

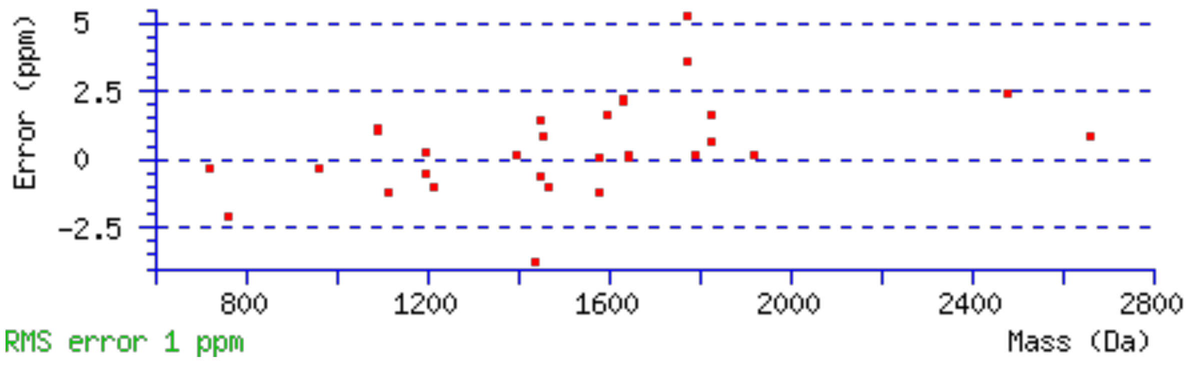

Mascot: http://www.matrixscience.com/

MASCOT Search Results

Protein View: sp|P00833|ATPE_SPIOL

>sp|P00833|ATPE_SPIOL ATP synthase epsilon chain, chloroplastic OS=Spinacia oleracea OX=3562 GN=atpE PE=1 SV=1

**Database:**	Uni-Spinach
**Score:**	591
**Nominal mass (M_r):**	14748
**Calculated pI:**	6.59

Sequence similarity is available as an NCBI BLAST search of sp|P00833|ATPE_SPIOL against nr.

Search parameters

**MS data file:**	\\128.97.66.218\tank\windowsVM\Bill Cramer\MGF\wc_QE_021417_Cramer_Gel_1S_4a.mgf
**Enzyme:**	Trypsin/P: cuts C-term side of KR.
**Fixed modifications:**	Carbamidomethyl (C)
**Variable modifications:**	Oxidation (M)

Protein sequence coverage: 85%

Matched peptides shown in ***bold red***.

```
  1 MTLNLCVLTP NRSIWNSEVK  EIILSTNSGQ IGVLPNHAPT ATAVDIGILR
 51 IRLNDQWLTL ALMGGFARIG NNEITILVND AERGSDIDPQ EAQQTLEIAE
101 ANLRKAEGKR  QKIEANLALR RARTRVEASN TISS
```

Unformatted sequence string: 134 residues (for pasting into other applications).

Sort peptides by ◉ Residue Number ○ Increasing Mass ○ Decreasing Mass

Show predicted peptides also

Query	Start – End	Observed	Mr(expt)	Mr(calc)	ppm	M	Score	Expect	Rank	U	Peptide
13894	2 – 12	650.8539	1299.6932	1299.6969	-2.81	0	53	1.2e-05	1	U	M.TLNLCVLTPNR.S
13897	2 – 12	650.8560	1299.6975	1299.6969	0.43	0	48	3.2e-05	1	U	M.TLNLCVLTPNR.S
29471	21 – 50	1024.2346	3069.6820	3069.6822	-0.083	0	46	6.7e-05	1	U	K.EIILSTNSGQIGVLPNHAPTATAVDIGILR.I
23195	53 – 68	903.4733	1804.9321	1804.9294	1.50	0	77	6.3e-08	1	U	R.LNDQWLTLALMGGFAR.I
23196	53 – 68	602.6519	1804.9337	1804.9294	2.39	0	44	7.8e-05	1	U	R.LNDQWLTLALMGGFAR.I
23384	53 – 68	911.4696	1820.9246	1820.9243	0.13	0	86	8.2e-09	1	U	R.LNDQWLTLALMGGFAR.I + Oxidation (M)
23385	53 – 68	607.9826	1820.9260	1820.9243	0.90	0	41	0.00014	1	U	R.LNDQWLTLALMGGFAR.I + Oxidation (M)
21418	69 – 83	557.6291	1669.8655	1669.8635	1.20	0	69	3.6e-07	1	U	R.IGNNEITILVNDAER.G
21419	69 – 83	835.9403	1669.8660	1669.8635	1.53	0	103	2.3e-10	1	U	R.IGNNEITILVNDAER.G
27047	84 – 104	766.7118	2297.1135	2297.1135	0.0061	0	70	3e-07	1	U	R.GSDIDPQEAQQTLEIAEANLR.K
27048	84 – 104	1149.5640	2297.1135	2297.1135	0.015	0	99	4.7e-10	1	U	R.GSDIDPQEAQQTLEIAEANLR.K
9962	111 – 120	385.8996	1154.6771	1154.6771	-0.046	1	15	0.074	1	U	R.QKIEANLALR.R
9963	111 – 120	578.3464	1154.6782	1154.6771	0.95	1	38	0.00029	1	U	R.QKIEANLALR.R
14146	111 – 121	437.9343	1310.7809	1310.7782	2.08	2	10	0.35	1	U	R.QKIEANLALRR.A
10191	124 – 134	582.7961	1163.5776	1163.5782	-0.54	1	15	0.042	1	U	R.TRVEASNTISS.-

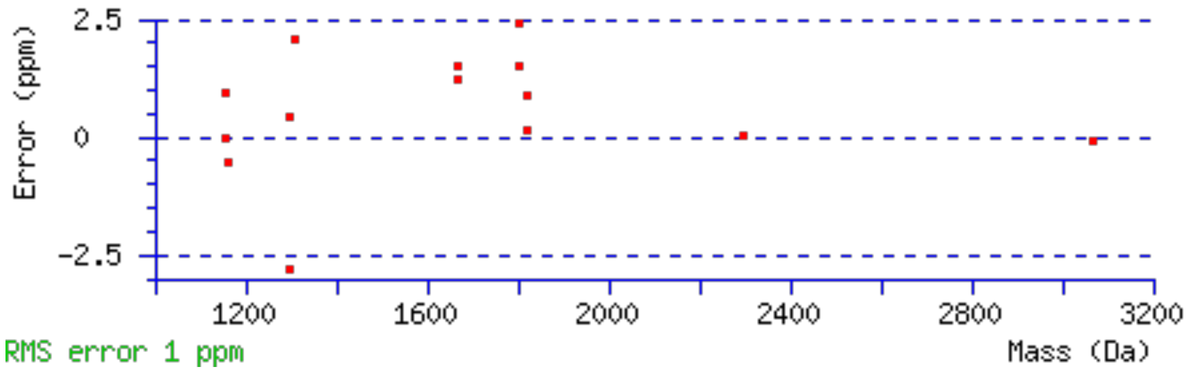

Mascot: http://www.matrixscience.com/

MASCOT Search Results

Protein View: tr|A0A0K9QKT8|A0A0K9QKT8_SPIOL

>tr|A0A0K9QKT8|A0A0K9QKT8_SPIOL Uncharacterized protein OS=Spinacia oleracea OX=3562 GN=SOVF_167830 PE=4 SV=1

**Database:**	Uni-Spinach
**Score:**	559
**Nominal mass (M_r):**	40479
**Calculated pI:**	6.17

Sequence similarity is available as an NCBI BLAST search of tr|A0A0K9QKT8|A0A0K9QKT8_SPIOL against nr.

Search parameters

**MS data file:**	\\128.97.66.218\tank\windowsVM\Bill Cramer\MGF\wc_QE_021417_Cramer_Gel_1S_4a.mgf
**Enzyme:**	Trypsin/P: cuts C-term side of KR.
**Fixed modifications:**	Carbamidomethyl (C)
**Variable modifications:**	Oxidation (M)

Protein sequence coverage: 51%

Matched peptides shown in ***bold red***.

```
  1 MASLLPFSLL KPTTICKASS TAAPPAALVE SLNDQFARKG INFLESTDTS
 51 TNISSPIVEL SVRNGSSLKL QLSNAHVTSY KPKVYWKDDG FEEVLYTLPL
101 SKGGIGLVLN DVTQPVTTTK KPDPFRRSEP AAAAAAKGSL LAGAEWSVRD
151 VDSDSFDAVQ VELSCTSGSL EITYVVSLYP ESMASAVLVK NNGNKAIGLT
201 SAILSHIQFK KRSGSGIQGL QGCSYCSHPP LSSPFEIVSP AEAMKAEDPG
251 MFSFSSESPS KLGEWTTQEV PITILKNKLS RVYTAPPSER SKKFYRTTPS
301 KYETVDQGRE LVFRVIRMGY DDILVSSPGS LSEKYGRDYF ICTGPASILV
351 PVTVNPGEEW RGAQVIEHDN LT
```

Unformatted sequence string: 372 residues (for pasting into other applications).

Sort peptides by ◉ Residue Number ○ Increasing Mass ○ Decreasing Mass

Show predicted peptides also

Query	Start – End	Observed	Mr(expt)	Mr(calc)	ppm	M	Score	Expect	Rank	U	Peptide
28819	39 – 63	903.1438	2706.4096	2706.4076	0.75	1	83	1.8e-08	1	U	R.KGINFLESTDTSTNISSPIVELSVR.N
28295	40 – 63	860.4461	2578.3164	2578.3126	1.46	0	78	5.1e-08	1	U	K.GINFLESTDTSTNISSPIVELSVR.N
20119	70 – 83	529.2953	1584.8641	1584.8624	1.12	1	9	0.15	1	U	K.LQLSNAHVTSYKPK.V
20120	70 – 83	397.2233	1584.8642	1584.8624	1.16	1	2	0.71	1	U	K.LQLSNAHVTSYKPK.V
27085	84 – 102	768.0619	2301.1638	2301.1569	3.01	1	24	0.0051	1	U	K.VYWKDDGFEEVLYTLPLSK.G
22208	88 – 102	863.4355	1724.8564	1724.8509	3.23	0	59	2.9e-06	1	U	K.DDGFEEVLYTLPLSK.G
23290	103 – 120	907.0056	1811.9966	1811.9993	-1.50	0	43	8.6e-05	1	U	K.GGIGLVLNDVTQPVTTTK.K
23291	103 – 120	605.0080	1812.0020	1811.9993	1.50	0	36	0.0004	1	U	K.GGIGLVLNDVTQPVTTTK.K
887	121 – 126	380.2105	758.4064	758.4075	-1.47	1	1	0.78	1	U	K.KPDPFR.R
6763	127 – 137	521.7847	1041.5548	1041.5567	-1.74	1	2	0.74	1	U	R.RSEPAAAAAAK.G
12490	138 – 149	623.3308	1244.6470	1244.6513	-3.45	0	30	0.0016	1	U	K.GSLLAGAEWSVR.D
20329	196 – 210	799.9679	1597.9213	1597.9192	1.34	0	63	2e-06	1	U	K.AIGLTSAILSHIQFK.K
20330	196 – 210	533.6481	1597.9225	1597.9192	2.09	0	9	0.49	1	U	K.AIGLTSAILSHIQFK.K
24034	196 – 212	471.5379	1882.1224	1882.1152	3.84	2	25	0.0037	1	U	K.AIGLTSAILSHIQFKKR.S
21861	246 – 261	851.8680	1701.7214	1701.7192	1.28	0	48	3.3e-05	1	U	K.AEDPGMFSFSSESPSK.L
22102	246 – 261	859.8636	1717.7126	1717.7141	-0.86	0	54	5.9e-06	1	U	K.AEDPGMFSFSSESPSK.L + Oxidation (M)
22237	262 – 276	864.4824	1726.9502	1726.9505	-0.17	0	55	7.3e-06	1	U	K.LGEWTTQEVPITILK.N
6318	282 – 290	510.2616	1018.5087	1018.5083	0.31	0	22	0.0082	1	U	R.VYTAPPSER.S
6319	282 – 290	510.2629	1018.5112	1018.5083	2.83	0	19	0.018	1	U	R.VYTAPPSER.S
18173	297 – 309	741.3648	1480.7150	1480.7158	-0.50	1	16	0.035	1	U	R.TTPSKYETVDQGR.E
18175	297 – 309	494.5795	1480.7167	1480.7158	0.63	1	31	0.0012	1	U	R.TTPSKYETVDQGR.E
5246	302 – 309	484.2282	966.4419	966.4407	1.23	0	16	0.033	1	U	K.YETVDQGR.E
23094	318 – 334	899.4330	1796.8514	1796.8502	0.65	0	63	1.2e-06	1	U	R.MGYDDILVSSPGSLSEK.Y
23095	318 – 334	599.9583	1796.8529	1796.8502	1.51	0	32	0.0011	1	U	R.MGYDDILVSSPGSLSEK.Y
23297	318 – 334	907.4302	1812.8458	1812.8451	0.39	0	48	3.1e-05	1	U	R.MGYDDILVSSPGSLSEK.Y + Oxidation (M)

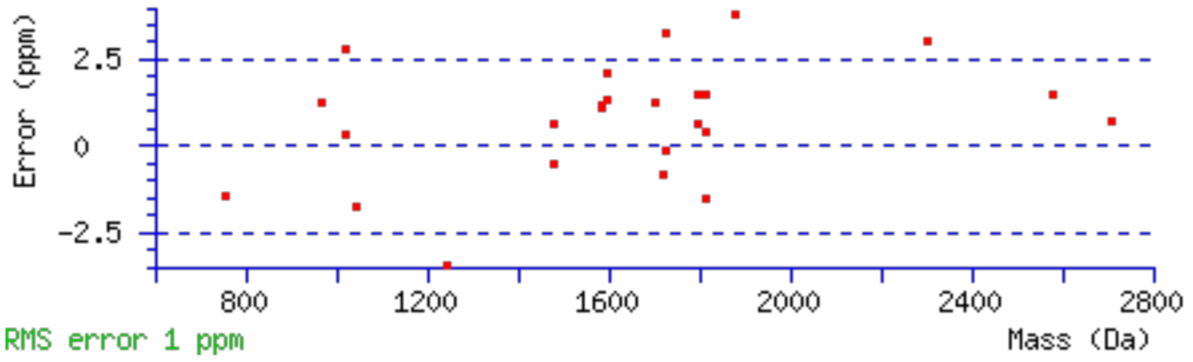

Mascot: http://www.matrixscience.com/

MASCOT Search Results

Protein View: sp|P10098|PSAC_SPIOL

>sp|P10098|PSAC_SPIOL Photosystem I iron-sulfur center OS=Spinacia oleracea OX=3562 GN=psaC PE=1 SV=3

**Database:**	Uni-Spinach
**Score:**	503
**Nominal mass (M_r):**	9531
**Calculated pI:**	6.67

Sequence similarity is available as an NCBI BLAST search of sp|P10098|PSAC_SPIOL against nr.

Search parameters

**MS data file:**	\\128.97.66.218\tank\windowsVM\Bill Cramer\MGF\wc_QE_021417_Cramer_Gel_1S_4a.mgf
**Enzyme:**	Trypsin/P: cuts C-term side of KR.
**Fixed modifications:**	Carbamidomethyl (C)
**Variable modifications:**	Oxidation (M)

Protein sequence coverage: 96%

Matched peptides shown in ***bold red***.

```
 1 MSHSVKIYDT CIGCTQCVRA CPTDVLEMIP WDGCKAKQIA SAPRTEDCVG
51 CKRCESACPT DFLSVRVYLW HETTRSMGLG Y
```

Unformatted sequence string: 81 residues (for pasting into other applications).

Sort peptides by ◉ Residue Number ◯ Increasing Mass ◯ Decreasing Mass

Show predicted peptides also

Query	Start – End	Observed	Mr(expt)	Mr(calc)	ppm	M	Score	Expect	Rank	U	Peptide
26350	2 – 19	728.6715	2182.9926	2182.9922	0.20	1	74	1.1e-07	1	U	M.SHSVKIYDTCIGCTQCVR.A
21048	7 – 19	823.3593	1644.7040	1644.7059	-1.10	0	60	1.9e-06	1	U	K.IYDTCIGCTQCVR.A
21049	7 – 19	549.2422	1644.7048	1644.7059	-0.66	0	35	0.00053	1	U	K.IYDTCIGCTQCVR.A
21050	7 – 19	823.3598	1644.7051	1644.7059	-0.47	0	28	0.003	1	U	K.IYDTCIGCTQCVR.A
21051	7 – 19	549.2430	1644.7071	1644.7059	0.73	0	15	0.054	1	U	K.IYDTCIGCTQCVR.A
24115	20 – 35	631.2836	1890.8290	1890.8314	-1.28	0	41	0.00025	1	U	R.ACPTDVLEMIPWDGCK.A
24116	20 – 35	946.4226	1890.8307	1890.8314	-0.40	0	56	7.5e-06	1	U	R.ACPTDVLEMIPWDGCK.A
24271	20 – 35	954.4191	1906.8237	1906.8264	-1.39	0	77	4.1e-08	1	U	R.ACPTDVLEMIPWDGCK.A + Oxidation (M)
606	38 – 44	371.7139	741.4133	741.4133	-0.026	0	18	0.066	1	U	K.QIASAPR.T
5268	45 – 52	484.6937	967.3728	967.3739	-1.20	0	25	0.0028	1	U	R.TEDCVGCK.R
8996	45 – 53	375.4984	1123.4734	1123.4750	-1.46	1	22	0.017	1	U	R.TEDCVGCKR.C
8997	45 – 53	562.7443	1123.4740	1123.4750	-0.88	1	42	0.00016	1	U	R.TEDCVGCKR.C
21776	53 – 66	849.3903	1696.7660	1696.7661	-0.082	1	14	0.13	1	U	K.RCESACPTDFLSVR.V
21777	53 – 66	566.5960	1696.7661	1696.7661	-0.021	1	15	0.13	1	U	K.RCESACPTDFLSVR.V
21779	53 – 66	566.5972	1696.7698	1696.7661	2.19	1	46	9.5e-05	1	U	K.RCESACPTDFLSVR.V
19395	54 – 66	771.3389	1540.6632	1540.6650	-1.16	0	39	0.00036	1	U	R.CESACPTDFLSVR.V
19396	54 – 66	771.3397	1540.6649	1540.6650	-0.083	0	70	3.6e-07	1	U	R.CESACPTDFLSVR.V
19397	54 – 66	771.3416	1540.6686	1540.6650	2.34	0	66	7.6e-07	1	U	R.CESACPTDFLSVR.V
19398	54 – 66	514.5638	1540.6694	1540.6650	2.86	0	23	0.017	1	U	R.CESACPTDFLSVR.V
11332	67 – 75	602.8087	1203.6029	1203.6037	-0.61	0	29	0.0021	1	U	R.VYLWHETTR.S
11334	67 – 75	402.2088	1203.6045	1203.6037	0.67	0	15	0.041	1	U	R.VYLWHETTR.S
11335	67 – 75	602.8101	1203.6056	1203.6037	1.65	0	26	0.004	1	U	R.VYLWHETTR.S
11336	67 – 75	402.2092	1203.6057	1203.6037	1.68	0	13	0.062	1	U	R.VYLWHETTR.S
23284	67 – 81	604.9624	1811.8654	1811.8665	-0.60	1	0	0.94	1	U	R.VYLWHETTRSMGLGY.-
23443	67 – 81	914.9380	1827.8614	1827.8614	0.024	1	12	0.079	1	U	R.VYLWHETTRSMGLGY.- + Oxidation (M)

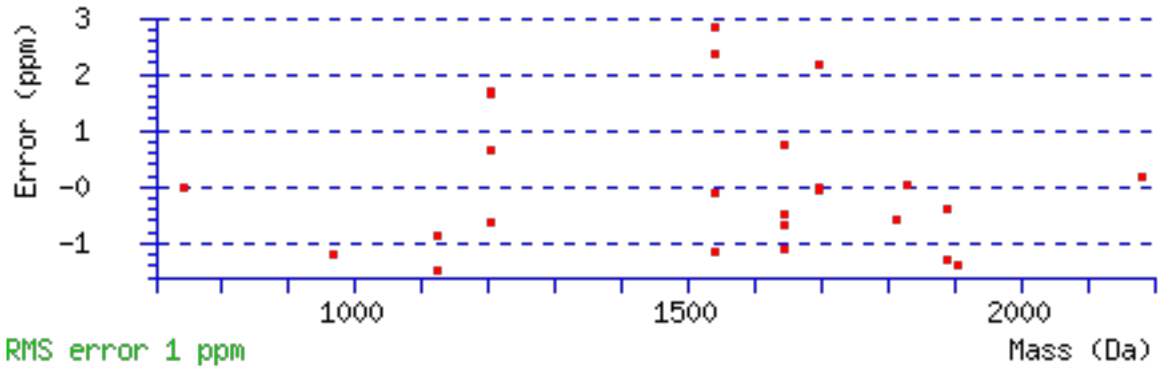

Mascot: http://www.matrixscience.com/

MASCOT Search Results

Protein View: sp|P06511|PSAA_SPIOL

>sp|P06511|PSAA_SPIOL Photosystem I P700 chlorophyll a apoprotein A1 OS=Spinacia oleracea OX=3562 GN=psaA PE=3 SV=1

**Database:**	Uni-Spinach
**Score:**	493
**Nominal mass (M_r):**	83161
**Calculated pI:**	6.74

Sequence similarity is available as **an NCBI BLAST search of sp|P06511|PSAA_SPIOL against nr**.

Search parameters

**MS data file:**	\\128.97.66.218\tank\windowsVM\Bill Cramer\MGF\wc_QE_021417_Cramer_Gel_1S_4a.mgf
**Enzyme:**	Trypsin/P: cuts C-term side of KR.
**Fixed modifications:**	Carbamidomethyl (C)
**Variable modifications:**	Oxidation (M)

Protein sequence coverage: 18%

Matched peptides shown in ***bold red***.

1 MIIR**SPEPEV** **KILVDRDPVK** **TSFEAWAKPG** **HFSR**TIAKGP ETTTWIWNLH
51 ADAHDFDSHT SDLEEISRKI FSAHFGQLSI IFLWLSGMYF HGARFSNYEA
101 WLSDPTHIGP SAQVVWPIVG QEILNGDVGG GFR**GIQITSG** **FFQIWR**ASGI
151 TSELQLYCTA IGALVFAALM LFAGWFHYHK AAPKLAWFQD VESMLNHHLA
201 GLLGLGSLSW AGHQIHVSLP INQFLNAGVD PKEIPLPHEL ILNRDLLAQL
251 YPSFAEGATP FFTLNWSK**YA** **DFLTFR**GGLD PVTGGLWLTD TAHHHLAIAI
301 LFLIAGHMYR **TNWGIGHGLK** **DILEAHKGPF** **TGQGHK**GLYE ILTTSWHAQL
351 ALNLAMLGSL TIVVAHHMYA MPPYPYLATD YGTQLSLFTH HMWIGGFLIV
401 GAAAHAAIFM VR**DYDPTTRY** **NDLLDRVLR**H RDAIISHLNW ACIFLGFHSF
451 GLYIHNDTMS ALGRPQDMFS DTAIQLQPVF AQWIQNTHAL APSATAPGAT
501 ASTSLTWGGS DLVAVGGKVA LLPIPLGTAD FLVHHIHAFT IHVTVLILLK
551 GVLFARSSR**L** **IPDKANLGFR** FPCDGPGRGG TCQVSAWDHV FLGLFWMYNS
601 ISVVIFHFSW KMQSDVWGSI SDQGVVTHIT GGNFAQSSIT INGWLRDFLW
651 AQASQVIQSY GSSLSAYGLF FLGAHFVWAF SLMFLFSGR**G** **YWQELIESIV**
701 **WAHNKLKVAP** **ATQPRALSIV** **QGR**AVGVTHY LLGGIATTWA FFLARIIAVG

Unformatted sequence string: **750 residues** (for pasting into other applications).

Sort peptides by ◉ Residue Number ○ Increasing Mass ○ Decreasing Mass

Show predicted peptides also

Query	Start – End	Observed	Mr(expt)	Mr(calc)	ppm	M	Score	Expect	Rank	U	Peptide
1347	5 – 11	393.2053	784.3961	784.3967	-0.67	0	8	0.43	1	U	R.SPEPEVK.I
7077	12 – 20	527.8159	1053.6172	1053.6182	-0.98	1	4	1.2	1	U	K.ILVDRDPVK.T
7078	12 – 20	527.8162	1053.6178	1053.6182	-0.46	1	4	1.2	1	U	K.ILVDRDPVK.T
7080	12 – 20	527.8166	1053.6187	1053.6182	0.45	1	13	0.16	1	U	K.ILVDRDPVK.T
24947	12 – 28	659.0279	1974.0618	1974.0574	2.21	2	0	0.93	1	U	K.ILVDRDPVKTSFEAWAK.P
20652	21 – 34	810.8988	1619.7831	1619.7845	-0.86	1	19	0.016	1	U	K.TSFEAWAKPGHFSR.T
20653	21 – 34	405.9531	1619.7832	1619.7845	-0.80	1	6	0.27	1	U	K.TSFEAWAKPGHFSR.T
20656	21 – 34	540.9358	1619.7856	1619.7845	0.72	1	6	0.25	1	U	K.TSFEAWAKPGHFSR.T
20657	21 – 34	405.9537	1619.7857	1619.7845	0.77	1	3	0.58	1	U	K.TSFEAWAKPGHFSR.T
20658	21 – 34	405.9541	1619.7874	1619.7845	1.81	1	14	0.049	1	U	K.TSFEAWAKPGHFSR.T
20659	21 – 34	405.9542	1619.7876	1619.7845	1.94	1	25	0.0041	1	U	K.TSFEAWAKPGHFSR.T
20660	21 – 34	540.9367	1619.7883	1619.7845	2.38	1	15	0.04	1	U	K.TSFEAWAKPGHFSR.T
20662	21 – 34	540.9368	1619.7886	1619.7845	2.55	1	20	0.012	1	U	K.TSFEAWAKPGHFSR.T
20663	21 – 34	540.9369	1619.7889	1619.7845	2.74	1	30	0.0016	1	U	K.TSFEAWAKPGHFSR.T
14	29 – 34	350.6795	699.3444	699.3453	-1.27	0	14	0.11	1	U	K.PGHFSR.T
19564	134 – 146	776.9176	1551.8207	1551.8198	0.59	0	84	1.2e-08	1	U	R.GIQITSGFFQIWR.A
19565	134 – 146	518.2815	1551.8226	1551.8198	1.79	0	63	1.3e-06	1	U	R.GIQITSGFFQIWR.A
6599	269 – 276	516.7616	1031.5086	1031.5076	0.94	0	44	7.9e-05	1	U	K.YADFLTFR.G
6600	269 – 276	516.7618	1031.5091	1031.5076	1.43	0	3	0.51	1	U	K.YADFLTFR.G
7858	311 – 320	541.7899	1081.5653	1081.5669	-1.46	0	38	0.00027	1	U	R.TNWGIGHGLK.D
7861	311 – 320	361.5297	1081.5674	1081.5669	0.48	0	11	0.09	1	U	R.TNWGIGHGLK.D
24092	311 – 327	945.0054	1887.9963	1887.9955	0.40	1	51	1.7e-05	1	U	R.TNWGIGHGLKDILEAHK.G
24094	311 – 327	630.3399	1887.9979	1887.9955	1.26	1	31	0.0012	1	U	R.TNWGIGHGLKDILEAHK.G

24095	311 – 327	378.6069	1887.9984	1887.9955	1.51	1	14	0.048	1	U	R.TNWGIGHGLKDILEAHK.G
24096	311 – 327	473.0071	1887.9991	1887.9955	1.91	1	48	3e-05	1	U	R.TNWGIGHGLKDILEAHK.G
24098	311 – 327	473.0077	1888.0016	1887.9955	3.23	1	19	0.015	1	U	R.TNWGIGHGLKDILEAHK.G
29069	311 – 336	700.3670	2797.4387	2797.4412	-0.89	2	19	0.018	1	U	R.TNWGIGHGLKDILEAHKGPFTGQGHK.G
29070	311 – 336	560.4961	2797.4439	2797.4412	0.96	2	5	0.36	1	U	R.TNWGIGHGLKDILEAHKGPFTGQGHK.G
29071	311 – 336	560.4967	2797.4473	2797.4412	2.16	2	1	0.78	1	U	R.TNWGIGHGLKDILEAHKGPFTGQGHK.G
22312	321 – 336	867.9479	1733.8813	1733.8849	-2.07	1	8	0.18	1	U	K.DILEAHKGPFTGQGHK.G
22313	321 – 336	867.9492	1733.8838	1733.8849	-0.62	1	1	0.74	1	U	K.DILEAHKGPFTGQGHK.G
22314	321 – 336	578.9687	1733.8841	1733.8849	-0.45	1	34	0.00071	1	U	K.DILEAHKGPFTGQGHK.G
22315	321 – 336	578.9689	1733.8848	1733.8849	-0.052	1	32	0.00098	1	U	K.DILEAHKGPFTGQGHK.G
22316	321 – 336	434.4786	1733.8854	1733.8849	0.27	1	31	0.0012	1	U	K.DILEAHKGPFTGQGHK.G
22317	321 – 336	578.9692	1733.8858	1733.8849	0.53	1	12	0.093	1	U	K.DILEAHKGPFTGQGHK.G
22318	321 – 336	434.4788	1733.8860	1733.8849	0.62	1	11	0.095	1	U	K.DILEAHKGPFTGQGHK.G
22320	321 – 336	578.9696	1733.8870	1733.8849	1.21	1	13	0.061	1	U	K.DILEAHKGPFTGQGHK.G
22322	321 – 336	434.4795	1733.8889	1733.8849	2.32	1	25	0.0047	1	U	K.DILEAHKGPFTGQGHK.G
22595	413 – 426	586.2776	1755.8110	1755.8064	2.61	1	13	0.088	1	U	R.DYDPTTRYNDLLDR.V
3975	420 – 426	454.7268	907.4391	907.4399	-0.96	0	13	0.48	1	U	R.YNDLLDR.V
3979	420 – 426	454.7274	907.4403	907.4399	0.40	0	3	0.47	3	U	R.YNDLLDR.V
13308	420 – 429	426.2385	1275.6937	1275.6935	0.11	1	37	0.00054	1	U	R.YNDLLDRVLR.H
12450	560 – 570	622.3612	1242.7079	1242.7084	-0.40	1	19	0.015	1	U	R.LIPDKANLGFR.F
12451	560 – 570	415.2433	1242.7080	1242.7084	-0.38	1	48	3.2e-05	1	U	R.LIPDKANLGFR.F
12452	560 – 570	415.2437	1242.7091	1242.7084	0.55	1	11	0.1	1	U	R.LIPDKANLGFR.F
12453	560 – 570	622.3622	1242.7099	1242.7084	1.16	1	35	0.00048	1	U	R.LIPDKANLGFR.F
12454	560 – 570	415.2440	1242.7102	1242.7084	1.39	1	22	0.0082	1	U	R.LIPDKANLGFR.F
24926	690 – 705	658.3377	1971.9913	1971.9843	3.58	0	40	0.00019	1	U	R.GYWQELIESIVWAHNK.L
7795	706 – 715	540.8299	1079.6453	1079.6451	0.18	1	9	0.32	2	U	K.LKVAPATQPR.A
2459	708 – 715	420.2400	838.4654	838.4661	-0.84	0	5	0.31	1	U	K.VAPATQPR.A
2460	708 – 715	420.2404	838.4662	838.4661	0.10	0	10	0.1	1	U	K.VAPATQPR.A
2570	716 – 723	422.2561	842.4976	842.4974	0.25	0	9	0.15	1	U	R.ALSIVQGR.A

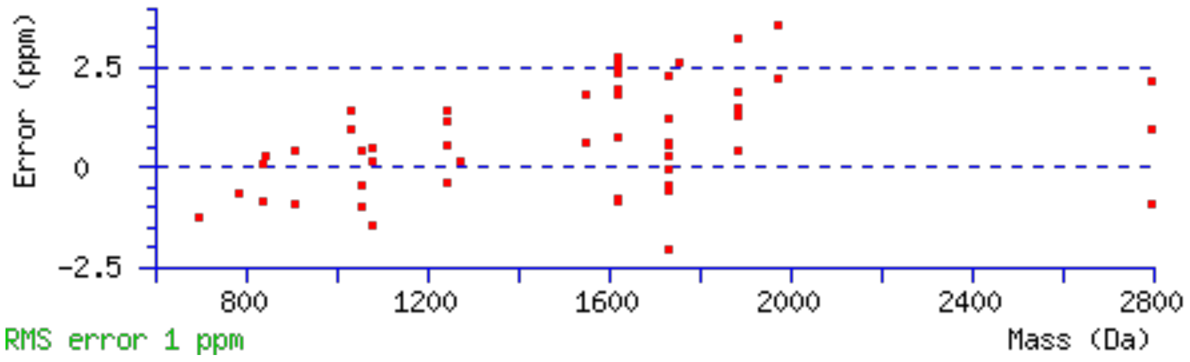

MASCOT Search Results

Protein View: tr|A0A0K9RU56|A0A0K9RU56_SPIOL

>tr|A0A0K9RU56|A0A0K9RU56_SPIOL Chlorophyll a-b binding protein, chloroplastic OS=Spinacia oleracea OX=3562 GN=SOVF_028160 PE=3 SV=1

**Database:**	Uni-Spinach
**Score:**	449
**Nominal mass (M_r):**	29397
**Calculated pI:**	6.90

Sequence similarity is available as **an NCBI BLAST search of tr|A0A0K9RU56|A0A0K9RU56_SPIOL against nr**.

Search parameters

**MS data file:**	\\128.97.66.218\tank\windowsVM\Bill Cramer\MGF\wc_QE_021417_Cramer_Gel_1S_4a.mgf
**Enzyme:**	Trypsin/P: cuts C-term side of KR.
**Fixed modifications:**	Carbamidomethyl (C)
**Variable modifications:**	Oxidation (M)

Protein sequence coverage: 27%

Matched peptides shown in ***bold red***.

```
  1 MSSVCASSAT  TAVCASSASS  QKKGNVLGAT  RASFLGGKKL  RDSSKYRTPV
 51 SSNSFSVSAA  AAEPERPIWF  PGSTPPSWLD  GSLPADFGFD  PLGFGSDPET
101 LRWMVQSEIV  HCRWAMLGAA  GIFIPEFLTK  LGILNTPSWY  TAGELDYFTD
151 TTTLFVVELV  LIGWAEGRRW  ADILKPGCVN  TDPIFPNNKL  TGTDVGYPGG
201 LWFDPLGWGS  GSPQKVKELR  TKEIKNGRLA  MLAVMGAWFQ  HIYTGTGPID
251 NLFAHLADPG  HATIFSAFTP  K
```

Unformatted sequence string: **271 residues** (for pasting into other applications).

Sort peptides by ◉ Residue Number ○ Increasing Mass ○ Decreasing Mass

Show predicted peptides also

Query	Start – End	Observed	Mr(expt)	Mr(calc)	ppm	M	Score	Expect	Rank	U	Peptide
17328	103 – 113	722.8447	1443.6749	1443.6751	-0.15	0	54	8.8e-06	1	U	R.WMVQSEIVHCR.W
17329	103 – 113	482.2323	1443.6749	1443.6751	-0.14	0	3	0.51	1	U	R.WMVQSEIVHCR.W
17331	103 – 113	482.2330	1443.6773	1443.6751	1.47	0	29	0.0017	1	U	R.WMVQSEIVHCR.W
17706	103 – 113	730.8418	1459.6690	1459.6700	-0.68	0	63	2e-06	1	U	R.WMVQSEIVHCR.W + Oxidation (M)
17709	103 – 113	487.5646	1459.6720	1459.6700	1.36	0	16	0.11	1	U	R.WMVQSEIVHCR.W + Oxidation (M)
17711	103 – 113	487.5646	1459.6721	1459.6700	1.38	0	11	0.35	1	U	R.WMVQSEIVHCR.W + Oxidation (M)
17712	103 – 113	730.8433	1459.6721	1459.6700	1.39	0	64	2e-06	1	U	R.WMVQSEIVHCR.W + Oxidation (M)
23864	114 – 130	933.0048	1863.9950	1863.9957	-0.35	0	56	5.4e-06	1	U	R.WAMLGAAGIFIPEFLTK.L
24009	114 – 130	941.0038	1879.9930	1879.9906	1.24	0	91	2.9e-09	1	U	R.WAMLGAAGIFIPEFLTK.L + Oxidation (M)
24010	114 – 130	627.6720	1879.9940	1879.9906	1.82	0	39	0.0002	1	U	R.WAMLGAAGIFIPEFLTK.L + Oxidation (M)
27797	169 – 189	614.5682	2454.2437	2454.2478	-1.67	2	9	0.16	1	U	R.RWADILKPGCVNTDPIFPNNK.L
27798	169 – 189	819.0907	2454.2504	2454.2478	1.05	2	26	0.0038	1	U	R.RWADILKPGCVNTDPIFPNNK.L
27799	169 – 189	819.0913	2454.2521	2454.2478	1.77	2	14	0.049	1	U	R.RWADILKPGCVNTDPIFPNNK.L
27062	170 – 189	1150.0791	2298.1437	2298.1467	-1.28	1	25	0.0044	1	U	R.WADILKPGCVNTDPIFPNNK.L
27063	170 – 189	1150.0800	2298.1454	2298.1467	-0.54	1	15	0.039	1	U	R.WADILKPGCVNTDPIFPNNK.L
27064	170 – 189	767.0561	2298.1466	2298.1467	-0.034	1	20	0.012	1	U	R.WADILKPGCVNTDPIFPNNK.L
27065	170 – 189	767.0570	2298.1492	2298.1467	1.09	1	26	0.0035	1	U	R.WADILKPGCVNTDPIFPNNK.L
28764	190 – 215	898.1086	2691.3040	2691.2970	2.60	0	76	7.9e-08	1	U	K.LTGTDVGYPGGLWFDPLGWGSGSPQK.V
28765	190 – 215	1346.6604	2691.3062	2691.2970	3.45	0	67	5.7e-07	1	U	K.LTGTDVGYPGGLWFDPLGWGSGSPQK.V

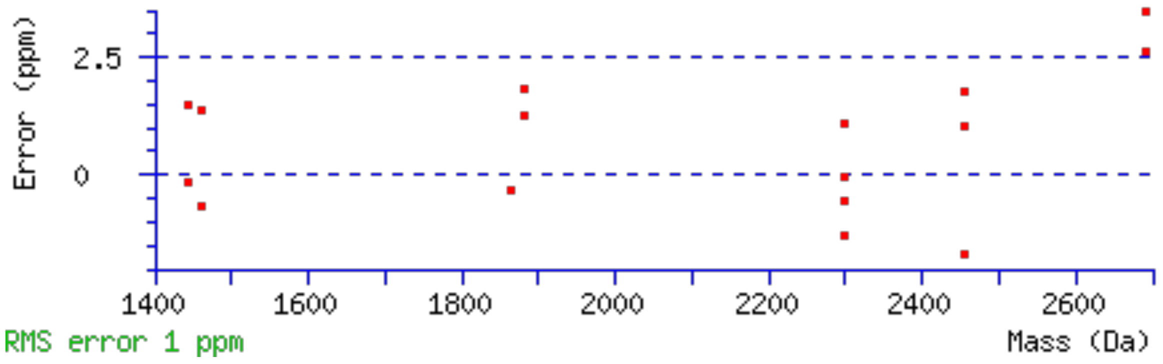

Mascot: http://www.matrixscience.com/

MASCOT Search Results

Protein View: tr|A0A0K9RXF7|A0A0K9RXF7_SPIOL

>tr|A0A0K9RXF7|A0A0K9RXF7_SPIOL Uncharacterized protein OS=Spinacia oleracea OX=3562 GN=SOVF_017720 PE=3 SV=1

**Database:**	Uni-Spinach
**Score:**	444
**Nominal mass (M_r):**	27664
**Calculated pI:**	5.80

Sequence similarity is available as an NCBI BLAST search of tr|A0A0K9RXF7|A0A0K9RXF7_SPIOL against nr.

Search parameters

**MS data file:**	\\128.97.66.218\tank\windowsVM\Bill Cramer\MGF\wc_QE_021417_Cramer_Gel_1S_4a.mgf
**Enzyme:**	Trypsin/P: cuts C-term side of KR.
**Fixed modifications:**	Carbamidomethyl (C)
**Variable modifications:**	Oxidation (M)

Protein sequence coverage: 47%

Matched peptides shown in ***bold red***.

```
  1 MAALQNPVAL QSRTTTAVAA LSTSSTTSTP KPFSLSFSSS TATFNPLRLK
 51 ILTASKLTAK PRGGALGTRM VDSTASRYAS ALADVADVTG TLEATNSDVE
101 KLIRIFSEEP VYYFFANPVI SIDNKRSVLD EIITTSGLQP HTANFINILI
151 DSERINLVKE ILNEFEDVFN KITGTEVAVV TSVVKLENDH LAQIAKGVQK
201 ITGAKNVRIK TVIDPSLVAG FTIRYGNEGS KLVDMSVKKQ LEEIAAQLEM
251 DDVTLAV
```

Unformatted sequence string: 257 residues (for pasting into other applications).

Sort peptides by ◉ Residue Number ○ Increasing Mass ○ Decreasing Mass

Show predicted peptides also

Query	Start – End	Observed	Mr(expt)	Mr(calc)	ppm	M	Score	Expect	Rank	U	Peptide
23812	32 – 48	929.9789	1857.9432	1857.9261	9.22	0	1	1.1	3	U	K.PFSLSFSSSTATFNPLR.L
27753	78 – 101	814.0636	2439.1690	2439.1653	1.52	0	94	1.5e-09	1	U	R.YASALADVADVTGTLEATNSDVEK.L
27754	78 – 101	1220.5930	2439.1714	2439.1653	2.49	0	120	6.1e-12	1	U	R.YASALADVADVTGTLEATNSDVEK.L
28565	105 – 126	883.4533	2647.3382	2647.3322	2.25	1	58	3.3e-06	1	U	R.IFSEEPVYYFFANPVISIDNKR.S
18462	160 – 171	748.8675	1495.7204	1495.7195	0.62	0	58	3.7e-06	1	U	K.EILNEFEDVFNK.I
18463	160 – 171	499.5810	1495.7212	1495.7195	1.17	0	18	0.023	1	U	K.EILNEFEDVFNK.I
16313	172 – 185	468.2772	1401.8098	1401.8079	1.33	0	30	0.004	1	U	K.ITGTEVAVVTSVVK.L
16314	172 – 185	701.9136	1401.8127	1401.8079	3.39	0	39	0.00037	1	U	K.ITGTEVAVVTSVVK.L
18323	211 – 224	744.9261	1487.8377	1487.8348	1.94	0	65	8.3e-07	1	U	K.TVIDPSLVAGFTIR.Y
18324	211 – 224	496.9532	1487.8379	1487.8348	2.08	0	55	7.5e-06	1	U	K.TVIDPSLVAGFTIR.Y
25937	239 – 257	706.0334	2115.0785	2115.0769	0.74	1	14	0.044	1	U	K.KQLEEIAAQLEMDDVTLAV.-
25058	240 – 257	994.4997	1986.9849	1986.9819	1.47	0	16	0.029	1	U	K.QLEEIAAQLEMDDVTLAV.-
25178	240 – 257	1002.4962	2002.9778	2002.9769	0.48	0	41	0.00015	1	U	K.QLEEIAAQLEMDDVTLAV.- + Oxidation (M)
25179	240 – 257	668.6673	2002.9800	2002.9769	1.58	0	29	0.002	1	U	K.QLEEIAAQLEMDDVTLAV.- + Oxidation (M)

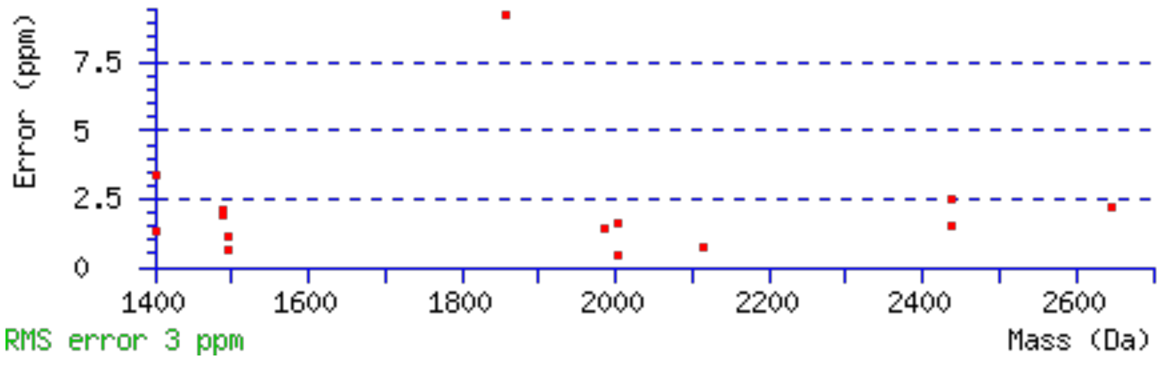

Mascot: http://www.matrixscience.com/

MASCOT Search Results

Protein View: tr|A0A0K9RVH9|A0A0K9RVH9_SPIOL

>tr|A0A0K9RVH9|A0A0K9RVH9_SPIOL Uncharacterized protein OS=Spinacia oleracea OX=3562 GN=SOVF_023270 PE=4 SV=1

**Database:**	Uni-Spinach
**Score:**	442
**Nominal mass (M_r):**	25568
**Calculated pI:**	9.40

Sequence similarity is available as **an NCBI BLAST search of tr|A0A0K9RVH9|A0A0K9RVH9_SPIOL against nr**.

Search parameters

**MS data file:**	\\128.97.66.218\tank\windowsVM\Bill Cramer\MGF\wc_QE_021417_Cramer_Gel_1S_4a.mgf
**Enzyme:**	Trypsin/P: cuts C-term side of KR.
**Fixed modifications:**	**Carbamidomethyl (C)**
**Variable modifications:**	**Oxidation (M)**

Protein sequence coverage: 43%

Matched peptides shown in ***bold red***.

```
  1 MSFTIPTNLY KPLATKPKHL SSSSFAPRSK IVCQQENDQQ QPKKLELAKV
 51 GANAAAALAL SSVLLSSWSV APDAAMADIA GLTPCKESKQ FAKREKQALK
101 KLQASLKLYA DDSAPALAIK ATMEKTKKRF DNYGKYGLLC GSDGLPHLIV
151 SGDQRHWGEF ITPGILFLYI AGWIGWVGRS YLIAIRDEKK PTQKEIIIDV
201 PLASSLLFRG FSWPVAAYRE LLNGELVDNN F
```

Unformatted sequence string: **231 residues** (for pasting into other applications).

Sort peptides by ◉ Residue Number ○ Increasing Mass ○ Decreasing Mass

Show predicted peptides also

Query	Start – End	Observed	Mr(expt)	Mr(calc)	ppm	M	Score	Expect	Rank	U	Peptide
25938	101 – 120	706.0726	2115.1960	2115.1939	1.01	2	38	0.00034	1	U	K.KLQASLKLYADDSAPALAIK.A
25063	102 – 120	663.3740	1987.1003	1987.0989	0.67	1	33	0.0024	1	U	K.LQASLKLYADDSAPALAIK.A
14965	108 – 120	674.3610	1346.7074	1346.7081	-0.57	0	11	0.096	1	U	K.LYADDSAPALAIK.A
14966	108 – 120	674.3616	1346.7087	1346.7081	0.40	0	15	0.38	1	U	K.LYADDSAPALAIK.A
14967	108 – 120	674.3618	1346.7090	1346.7081	0.66	0	10	1	2	U	K.LYADDSAPALAIK.A
14968	108 – 120	449.9103	1346.7090	1346.7081	0.66	0	19	0.015	1	U	K.LYADDSAPALAIK.A
14970	108 – 120	449.9104	1346.7093	1346.7081	0.88	0	10	0.12	1	U	K.LYADDSAPALAIK.A
14971	108 – 120	674.3620	1346.7095	1346.7081	1.00	0	31	0.0012	1	U	K.LYADDSAPALAIK.A
14972	108 – 120	674.3629	1346.7112	1346.7081	2.30	0	43	0.00054	1	U	K.LYADDSAPALAIK.A
6483	128 – 135	514.2700	1026.5255	1026.5247	0.84	2	16	0.035	1	U	K.KRFDNYGK.Y
3740	129 – 135	450.2216	898.4286	898.4297	-1.22	1	2	2.3	1	U	K.RFDNYGK.Y
610	130 – 135	372.1712	742.3278	742.3286	-1.07	0	1	1.4	1	U	R.FDNYGK.Y
611	130 – 135	372.1717	742.3288	742.3286	0.25	0	0	2	1	U	R.FDNYGK.Y
26210	136 – 155	1079.0404	2156.0663	2156.0685	-1.00	0	77	1.4e-07	1	U	K.YGLLCGSDGLPHLIVSGDQR.H
26211	136 – 155	719.6972	2156.0697	2156.0685	0.58	0	34	0.00067	1	U	K.YGLLCGSDGLPHLIVSGDQR.H
26212	136 – 155	719.6978	2156.0715	2156.0685	1.40	0	47	4e-05	1	U	K.YGLLCGSDGLPHLIVSGDQR.H
26213	136 – 155	719.7001	2156.0785	2156.0685	4.67	0	57	4.5e-06	1	U	K.YGLLCGSDGLPHLIVSGDQR.H
2382	180 – 186	418.2551	834.4957	834.4963	-0.76	0	27	0.006	1	U	R.SYLIAIR.D
2384	180 – 186	418.2557	834.4969	834.4963	0.71	0	27	0.0061	1	U	R.SYLIAIR.D
11423	180 – 189	604.3373	1206.6600	1206.6608	-0.65	1	22	0.0095	1	U	R.SYLIAIRDEK.K
11425	180 – 189	604.3378	1206.6611	1206.6608	0.28	1	26	0.0036	1	U	R.SYLIAIRDEK.K
11426	180 – 189	403.2283	1206.6632	1206.6608	1.97	1	10	0.12	1	U	R.SYLIAIRDEK.K
26878	190 – 209	756.7824	2267.3255	2267.3253	0.090	2	11	0.087	1	U	K.KPTQKEIIIDVPLASSLLFR.G
26879	190 – 209	567.8395	2267.3288	2267.3253	1.57	2	48	1.5e-05	1	U	K.KPTQKEIIIDVPLASSLLFR.G
21646	195 – 209	843.4966	1684.9786	1684.9763	1.33	0	67	5.9e-07	1	U	K.EIIIDVPLASSLLFR.G
21647	195 – 209	562.6677	1684.9813	1684.9763	2.96	0	48	3.2e-05	1	U	K.EIIIDVPLASSLLFR.G
9898	210 – 219	385.1977	1152.5713	1152.5716	-0.27	0	29	0.002	1	U	R.GFSWPVAAYR.E
9900	210 – 219	577.2934	1152.5722	1152.5716	0.52	0	19	0.017	1	U	R.GFSWPVAAYR.E
9902	210 – 219	577.2942	1152.5738	1152.5716	1.85	0	36	0.00047	1	U	R.GFSWPVAAYR.E
9904	210 – 219	577.2964	1152.5783	1152.5716	5.77	0	7	0.23	1	U	R.GFSWPVAAYR.E

15647	220 - 231	688.8366	1375.6587	1375.6619	-2.39	0	2	0.6	1	U	R.ELLNGELVDNNF.-

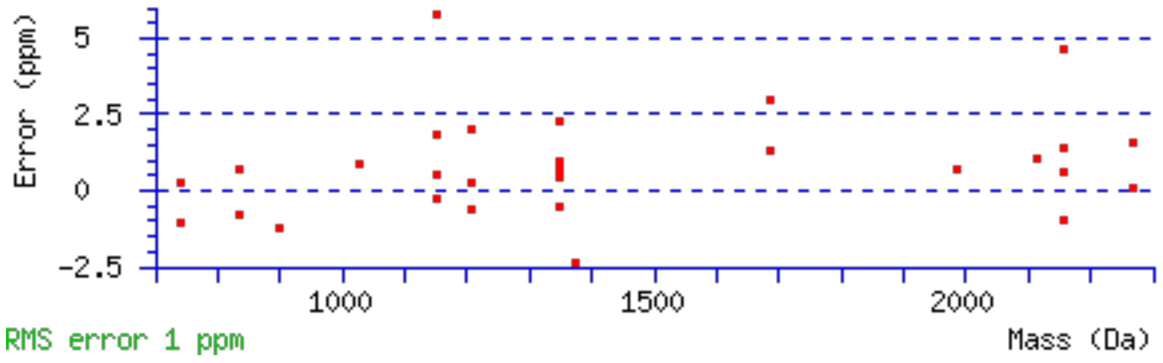

Mascot: http://www.matrixscience.com/

MASCOT Search Results

Protein View: tr|A0A0K9R5V6|A0A0K9R5V6_SPIOL

>tr|A0A0K9R5V6|A0A0K9R5V6_SPIOL Rhodanese domain-containing protein OS=Spinacia oleracea OX=3562 GN=SOVF_109900 PE=4 SV=1

**Database:**	Uni-Spinach
**Score:**	422
**Nominal mass (M_r):**	43226
**Calculated pI:**	9.44

Sequence similarity is available as an NCBI BLAST search of tr|A0A0K9R5V6|A0A0K9R5V6_SPIOL against nr.

Search parameters

**MS data file:**	\\128.97.66.218\tank\windowsVM\Bill Cramer\MGF\wc_QE_021417_Cramer_Gel_1S_4a.mgf
**Enzyme:**	Trypsin/P: cuts C-term side of KR.
**Fixed modifications:**	Carbamidomethyl (C)
**Variable modifications:**	Oxidation (M)

Protein sequence coverage: 36%

Matched peptides shown in ***bold red***.

```
  1 MAMEMMALRV SASAKPTISP SSSSSSSQKS QLLKQSSKLQ LKQAQNLFFP
 51 TSTSLSLLTL FTTPFDAKAV SLPKEQLVTS LTQVEQTIDQ VQEVGSSVFD
101 SAQKVFQVVA EALKPGIDAA TPIVQQAGQE AFKAASPLIS EASKKAQEAM
151 QNSGISSESM TTATQTVTSA AEQTTKAFED AKPLASSTFE TISNSDPALL
201 AEGAAALFLA YLLFPRVWSI VSFNLRGYKG GLTPAQTLEM LCTQNYYLID
251 MRSEKDKNKA GIPQLPSSAK SRMIAIPLEE LPSKVRNLVR NSKKVEAEIV
301 ALKVSYLKKL SKSTNIVIMD SYSDSAKTVA KSLTGLGFKN SWILTDGFSG
351 GKGWLQSRLG TESYNLSFGE IFSPSRIISG GTGRFGTTSS TVQIGRKMLP
401 GSN
```

Unformatted sequence string: 403 residues (for pasting into other applications).

Sort peptides by ◉ Residue Number ○ Increasing Mass ○ Decreasing Mass

Show predicted peptides also

Query	Start – End	Observed	Mr(expt)	Mr(calc)	ppm	M	Score	Expect	Rank	U	Peptide
29429	105 – 133	1009.2191	3024.6353	3024.6284	2.28	1	79	4.4e-08	1	U	K.VFQVVAEALKPGIDAATPIVQQAGQEAFK.A
29430	105 – 133	757.1665	3024.6369	3024.6284	2.80	1	50	3.6e-05	1	U	K.VFQVVAEALKPGIDAATPIVQQAGQEAFK.A
7586	134 – 144	537.2957	1072.5768	1072.5764	0.37	0	29	0.0019	1	U	K.AASPLISEASK.K
7587	134 – 144	537.2974	1072.5803	1072.5764	3.62	0	21	0.012	1	U	K.AASPLISEASK.K
11234	134 – 145	401.2306	1200.6700	1200.6714	-1.11	1	10	0.13	1	U	K.AASPLISEASKK.A
11798	217 – 226	610.8430	1219.6715	1219.6713	0.15	0	54	8e-06	1	U	R.VWSIVSFNLR.G
7457	260 – 270	534.8062	1067.5978	1067.5975	0.28	0	21	0.039	1	U	K.AGIPQLPSSAK.S
7458	260 – 270	534.8069	1067.5993	1067.5975	1.71	0	22	0.033	1	U	K.AGIPQLPSSAK.S
14801	273 – 284	670.8785	1339.7425	1339.7421	0.33	0	37	0.00096	1	U	R.MIAIPLEELPSK.V
15154	273 – 284	678.8756	1355.7367	1355.7370	-0.23	0	42	0.00011	1	U	R.MIAIPLEELPSK.V + Oxidation (M)
8394	294 – 303	367.2291	1098.6653	1098.6648	0.47	1	21	0.047	1	U	K.KVEAEIVALK.V
20817	313 – 327	815.8870	1629.7595	1629.7556	2.39	0	63	1.3e-06	1	U	K.STNIVIMDSYSDSAK.T
21074	313 – 327	823.8813	1645.7480	1645.7505	-1.49	0	6	1.1	1	U	K.STNIVIMDSYSDSAK.T + Oxidation (M)
15797	340 – 352	691.3403	1380.6660	1380.6674	-0.97	0	45	7.2e-05	1	U	K.NSWILTDGFSGGK.G
648	353 – 358	373.7013	745.3880	745.3871	1.23	0	4	0.41	1	U	K.GWLQSR.L
25176	359 – 376	1002.4887	2002.9629	2002.9636	-0.37	0	124	2.1e-12	1	U	R.LGTESYNLSFGEIFSPSR.I
25177	359 – 376	668.6625	2002.9658	2002.9636	1.08	0	3	0.56	1	U	R.LGTESYNLSFGEIFSPSR.I
12680	385 – 396	627.3265	1252.6384	1252.6412	-2.19	0	37	0.00033	1	U	R.FGTTSSTVQIGR.K

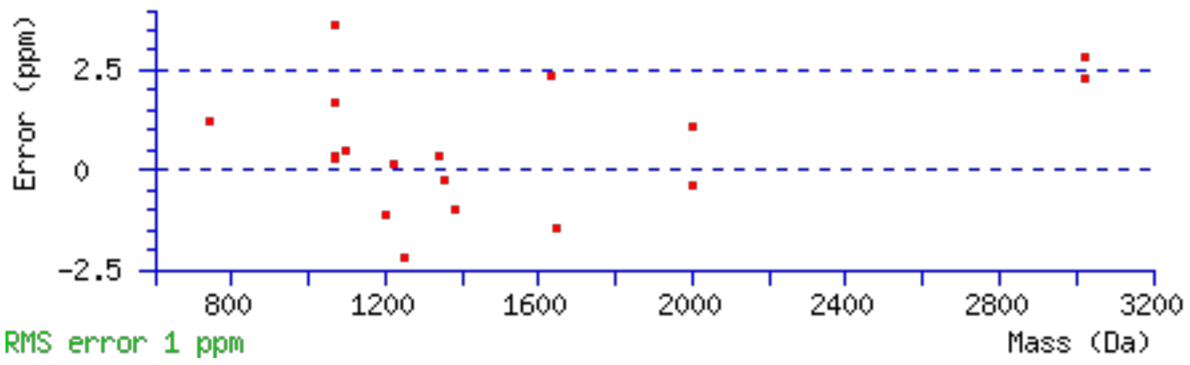

MASCOT Search Results

Protein View: sp|P00875|RBL_SPIOL

>sp|P00875|RBL_SPIOL Ribulose bisphosphate carboxylase large chain OS=Spinacia oleracea OX=3562 GN=rbcL PE=1 SV=1

**Database:**	Uni-Spinach
**Score:**	409
**Nominal mass (M_r):**	53220
**Calculated pI:**	6.13

Sequence similarity is available as **an NCBI BLAST search of sp|P00875|RBL_SPIOL against nr**.

Search parameters

**MS data file:**	\\128.97.66.218\tank\windowsVM\Bill Cramer\MGF\wc_QE_021417_Cramer_Gel_1S_4a.mgf
**Enzyme:**	Trypsin/P: cuts C-term side of KR.
**Fixed modifications:**	Carbamidomethyl (C)
**Variable modifications:**	Oxidation (M)

Protein sequence coverage: 39%

Matched peptides shown in ***bold red***.

1 MSPQTETKAS VEFKAGVKDY K**LTYYTPEYE** **TLDTDILAAF** **R**VSPQPGVPP
51 EEAGAAVAAE SSTGTWTTVW TDGLTNLDRY KGRCYHIEPV AGEENQYICY
101 VAYPLDLFEE GSVTNMFTSI VGNVFGFKAL R**ALRLEDLR**I PVAYVK**TFQG**
151 **PPHGIQVER**D KLNK**YGRPLL** **GCTIKPK**LGL SAKNYGRAVY ECLR**GGLDFT**
201 **KDDENVNSQP** **FMR**WRDR**FLF** **CAEALYK**AQA ETGEIK**GHYL** **NATAGTCEDM**
251 **MK**RAVFAREL GVPIVMHDYL TGGFTANTTL SHYCR**DNGLL** **LHIHRAMHAV**
301 **IDRQKNHGMH** **FR**VLAKALR**L** **SGGDHIHSGT** **VVGK**LEGERD **ITLGFVDLLR**
351 **DDYTEKDR**SR GIYFTQSWVS TPGVLPVASG GIHVWHMPAL TEIFGDDSVL
401 QFGGGTLGHP WGNAPGAVAN R**VALEACVQA** **R**NEGRDLAR**E** **GNTIIR**EATK
451 WSPELAAACE VWK**EIKFEFP** **AMDTV**

Unformatted sequence string: **475 residues** (for pasting into other applications).

Sort peptides by ◉ Residue Number ○ Increasing Mass ○ Decreasing Mass

Show predicted peptides also

Query	Start – End	Observed	Mr(expt)	Mr(calc)	ppm	M	Score	Expect	Rank	U	Peptide
27586	22 – 41	799.0647	2394.1724	2394.1631	3.88	0	72	1.7e-07	1	U	K.LTYYTPEYETLDTDILAAFR.V
5614	132 – 139	493.2930	984.5714	984.5716	-0.22	1	4	0.53	1	U	R.ALRLEDLR.I
17824	147 – 159	489.2559	1464.7460	1464.7474	-0.93	0	17	0.025	1	U	K.TFQGPPHGIQVER.D
17825	147 – 159	733.3804	1464.7462	1464.7474	-0.77	0	45	6.6e-05	1	U	K.TFQGPPHGIQVER.D
17826	147 – 159	733.3808	1464.7470	1464.7474	-0.24	0	67	5.1e-07	1	U	K.TFQGPPHGIQVER.D
18612	165 – 177	501.6212	1501.8417	1501.8439	-1.49	2	6	0.26	1	U	K.YGRPLLGCTIKPK.L
18613	165 – 177	376.4679	1501.8426	1501.8439	-0.87	2	4	0.4	1	U	K.YGRPLLGCTIKPK.L
26274	195 – 213	723.9985	2168.9736	2168.9797	-2.83	1	15	0.11	1	U	R.GGLDFTKDDENVNSQPFMR.W
26275	195 – 213	1085.4999	2168.9853	2168.9797	2.59	1	54	1.9e-05	1	U	R.GGLDFTKDDENVNSQPFMR.W
26369	195 – 213	729.3320	2184.9742	2184.9746	-0.18	1	33	0.0024	1	U	R.GGLDFTKDDENVNSQPFMR.W + Oxidation (M)
17507	202 – 213	726.3138	1450.6130	1450.6147	-1.13	0	28	0.0028	1	U	K.DDENVNSQPFMR.W
17859	202 – 213	734.3097	1466.6048	1466.6096	-3.24	0	18	0.021	1	U	K.DDENVNSQPFMR.W + Oxidation (M)
17861	202 – 213	734.3132	1466.6118	1466.6096	1.47	0	18	0.021	1	U	K.DDENVNSQPFMR.W + Oxidation (M)
12899	218 – 227	631.3190	1260.6235	1260.6213	1.81	0	37	0.00033	1	U	R.FLFCAEALYK.A
23101	237 – 252	600.2560	1797.7462	1797.7484	-1.25	0	37	0.00018	1	U	K.GHYLNATAGTCEDMMK.R
23103	237 – 252	899.8811	1797.7476	1797.7484	-0.43	0	1	0.76	1	U	K.GHYLNATAGTCEDMMK.R
23306	237 – 252	605.5894	1813.7463	1813.7433	1.62	0	17	0.021	1	U	K.GHYLNATAGTCEDMMK.R + Oxidation (M)
10841	286 – 295	594.3356	1186.6567	1186.6571	-0.35	0	47	4e-05	1	U	R.DNGLLLHIHR.A
10842	286 – 295	396.5601	1186.6585	1186.6571	1.18	0	23	0.0065	1	U	R.DNGLLLHIHR.A
4383	296 – 303	464.7364	927.4583	927.4596	-1.42	0	32	0.0032	1	U	R.AMHAVIDR.Q +

											Oxidation (M)
9932	304 - 312	385.5258	1153.5555	1153.5563	-0.68	1	9	0.15	1	U	R.QKNHGMHFR.V
17777	320 - 334	732.3827	1462.7508	1462.7529	-1.43	0	4	0.44	1	U	R.LSGGDHIHSGTVVGK.L
17779	320 - 334	488.5911	1462.7514	1462.7529	-1.02	0	7	0.23	1	U	R.LSGGDHIHSGTVVGK.L
17780	320 - 334	488.5913	1462.7521	1462.7529	-0.54	0	4	0.46	1	U	R.LSGGDHIHSGTVVGK.L
12908	340 - 350	631.3622	1260.7099	1260.7078	1.65	0	56	6.2e-06	1	U	R.DITLGFVDLLR.D
6735	351 - 358	521.2275	1040.4405	1040.4411	-0.50	1	27	0.005	1	U	R.DDYTEKDR.S
8796	422 - 431	558.7951	1115.5756	1115.5757	-0.066	0	43	9.9e-05	1	U	R.VALEACVQAR.N
8797	422 - 431	558.7959	1115.5772	1115.5757	1.37	0	5	0.34	1	U	R.VALEACVQAR.N
1679	440 - 446	401.7240	801.4334	801.4344	-1.25	0	18	0.038	1	U	R.EGNTIIR.E
1687	440 - 446	401.7248	801.4350	801.4344	0.75	0	16	0.038	1	U	R.EGNTIIR.E
16875	464 - 475	713.8493	1425.6840	1425.6850	-0.68	1	8	0.18	1	U	K.EIKFEFPAMDTV.-
17278	464 - 475	721.8497	1441.6849	1441.6799	3.44	1	9	0.14	1	U	K.EIKFEFPAMDTV.- + Oxidation (M)

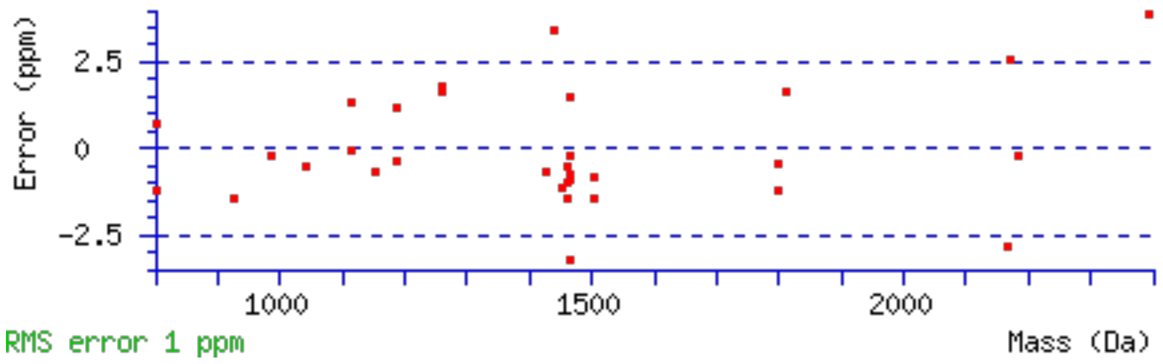

MATRIX SCIENCE MASCOT Search Results

Protein View: tr|A0A0K9RHQ2|A0A0K9RHQ2_SPIOL

>tr|A0A0K9RHQ2|A0A0K9RHQ2_SPIOL Uncharacterized protein OS=Spinacia oleracea OX=3562 GN=SOVF_071290 PE=4 SV=1

**Database:**	Uni-Spinach
**Score:**	371
**Nominal mass (M_r):**	18374
**Calculated pI:**	9.33

Sequence similarity is available as an NCBI BLAST search of tr|A0A0K9RHQ2|A0A0K9RHQ2_SPIOL against nr.

Search parameters

**MS data file:**	\\128.97.66.218\tank\windowsVM\Bill Cramer\MGF\wc_QE_021417_Cramer_Gel_1S_4a.mgf
**Enzyme:**	Trypsin/P: cuts C-term side of KR.
**Fixed modifications:**	Carbamidomethyl (C)
**Variable modifications:**	Oxidation (M)

Protein sequence coverage: 23%

Matched peptides shown in ***bold red***.

```
  1 MAAATASLSS TLLAPCSSKQ PQPQQQHQHQ QLKCKSFSGL RPLKLNISSN
 51 NSSSSLSMSS ARRSMTCRAE LSPSLVISLS TGLSLFLGRF VFFNFQRENM
101 AKQVPEQNGM SHFEAGDTRA KEYVSLLKSN DPVGFNIVDV LAWGSIGHIV
151 AYYILATASN GYDPSFF
```

Unformatted sequence string: 167 residues (for pasting into other applications).

Sort peptides by ◉ Residue Number ○ Increasing Mass ○ Decreasing Mass

Show predicted peptides also

Query	Start – End	Observed	Mr(expt)	Mr(calc)	ppm	M	Score	Expect	Rank	U	Peptide
8526	90 – 97	552.7851	1103.5556	1103.5553	0.27	0	43	9.4e-05	1	U	R.FVFFNFQR.E
8527	90 – 97	552.7858	1103.5570	1103.5553	1.57	0	28	0.0025	1	U	R.FVFFNFQR.E
21536	90 – 102	839.4149	1676.8151	1676.8133	1.09	1	43	8.4e-05	1	U	R.FVFFNFQRENMAK.Q
21538	90 – 102	559.9474	1676.8204	1676.8133	4.21	1	19	0.015	1	U	R.FVFFNFQRENMAK.Q
21735	90 – 102	565.2760	1692.8060	1692.8082	-1.30	1	12	0.082	1	U	R.FVFFNFQRENMAK.Q + Oxidation (M)
21736	90 – 102	847.4108	1692.8070	1692.8082	-0.74	1	32	0.001	1	U	R.FVFFNFQRENMAK.Q + Oxidation (M)
24229	103 – 119	634.9508	1901.8305	1901.8326	-1.12	0	32	0.0018	1	U	K.QVPEQNGMSHFEAGDTR.A
24230	103 – 119	951.9229	1901.8312	1901.8326	-0.77	0	61	2.4e-06	1	U	K.QVPEQNGMSHFEAGDTR.A
24231	103 – 119	634.9513	1901.8320	1901.8326	-0.33	0	33	0.0017	1	U	K.QVPEQNGMSHFEAGDTR.A
24232	103 – 119	951.9249	1901.8353	1901.8326	1.39	0	36	0.00077	1	U	K.QVPEQNGMSHFEAGDTR.A
24233	103 – 119	634.9525	1901.8356	1901.8326	1.55	0	44	0.00013	1	U	K.QVPEQNGMSHFEAGDTR.A
24234	103 – 119	634.9528	1901.8365	1901.8326	2.04	0	26	0.008	1	U	K.QVPEQNGMSHFEAGDTR.A
24235	103 – 119	634.9530	1901.8373	1901.8326	2.44	0	9	0.37	1	U	K.QVPEQNGMSHFEAGDTR.A
24349	103 – 119	640.2824	1917.8253	1917.8275	-1.16	0	14	0.083	1	U	K.QVPEQNGMSHFEAGDTR.A + Oxidation (M)
24350	103 – 119	959.9208	1917.8270	1917.8275	-0.27	0	0	2	1	U	K.QVPEQNGMSHFEAGDTR.A + Oxidation (M)
24351	103 – 119	640.2830	1917.8271	1917.8275	-0.23	0	44	8e-05	1	U	K.QVPEQNGMSHFEAGDTR.A + Oxidation (M)
24352	103 – 119	640.2830	1917.8272	1917.8275	-0.19	0	3	1	1	U	K.QVPEQNGMSHFEAGDTR.A + Oxidation (M)
24353	103 – 119	640.2832	1917.8278	1917.8275	0.12	0	66	5.5e-07	1	U	K.QVPEQNGMSHFEAGDTR.A + Oxidation (M)
24354	103 – 119	959.9212	1917.8278	1917.8275	0.16	0	3	1.2	1	U	K.QVPEQNGMSHFEAGDTR.A + Oxidation (M)
24355	103 – 119	640.2832	1917.8279	1917.8275	0.19	0	37	0.00039	1	U	K.QVPEQNGMSHFEAGDTR.A + Oxidation (M)
24356	103 – 119	640.2836	1917.8290	1917.8275	0.77	0	47	4.4e-05	1	U	K.QVPEQNGMSHFEAGDTR.A

											+ Oxidation (M)
6974	120 - 128	350.8781	1049.6123	1049.6121	0.25	1	1	2.8	1	U	R.AKEYVSLLK.S
6975	120 - 128	525.8135	1049.6125	1049.6121	0.41	1	5	1.1	1	U	R.AKEYVSLLK.S
2757	122 - 128	426.2474	850.4802	850.4800	0.26	0	22	0.024	1	U	K.EYVSLLK.S

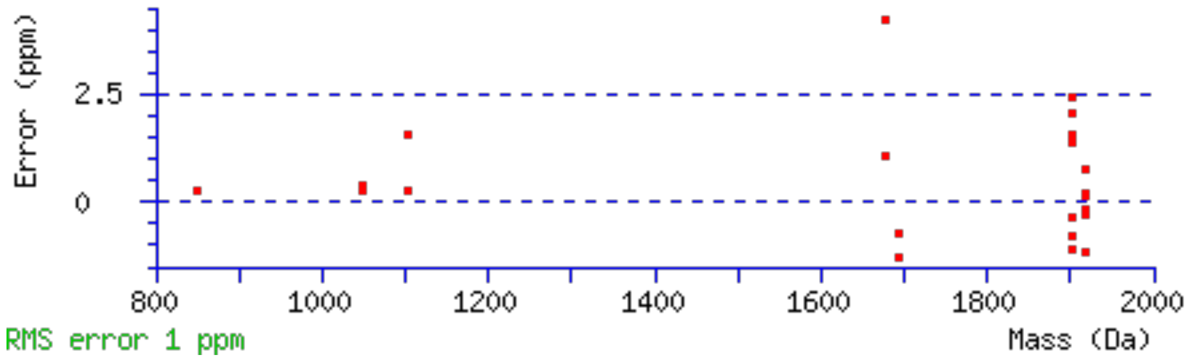

MASCOT Search Results

Protein View: sp|P00825|ATPB_SPIOL

>sp|P00825|ATPB_SPIOL ATP synthase subunit beta, chloroplastic OS=Spinacia oleracea OX=3562 GN=atpB PE=1 SV=2

**Database:**	Uni-Spinach
**Score:**	2106
**Nominal mass (M_r):**	53768
**Calculated pI:**	5.22

Sequence similarity is available as an NCBI BLAST search of sp|P00825|ATPB_SPIOL against nr.

Search parameters

**MS data file:**	\\128.97.66.218\tank\windowsVM\Bill Cramer\MGF\wc_QE_021417_Cramer_Gel_3S_4a.mgf
**Enzyme:**	Trypsin: cuts C-term side of KR unless next residue is P.
**Fixed modifications:**	Carbamidomethyl (C)
**Variable modifications:**	Oxidation (M)

Protein sequence coverage: 71%

Matched peptides shown in ***bold red***.

```
  1 MRINPTTSDP GVSTLEKKNL GRIAQIIGPV LDVAFPPGKM PNIYNALIVK
 51 GRDTAGQPMN VTCEVQQLLG NNRVRAVAMS ATDGLTRGME VIDTGAPLSV
101 PVGGATLGRI FNVLGEPVDN LGPVDTRTTS PIHRSAPAFT QLDTKLSIFE
151 TGIKVVDLLA PYRRGGKIGL FGGAGVGKTV LIMELINNIA KAHGGVSVFG
201 GVGERTREGN DLYMEMKESG VINEQNIAES KVALVYGQMN EPPGARMRVG
251 LTALTMAEYF RDVNEQDVLL FIDNIFRFVQ AGSEVSALLG RMPSAVGYQP
301 TLSTEMGSLQ ERITSTKEGS ITSIQAVYVP ADDLTDPAPA TTFAHLDATT
351 VLSRGLAAKG IYPAVDPLDS TSTMLQPRIV GEEHYEIAQR VKETLQRYKE
401 LQDIIAILGL DELSEEDRLT VARARKIERF LSQPFFVAEV FTGSPGKYVG
451 LAETIRGFQL ILSGELDSLP EQAFYLVGNI DEATAKAMNL EMESKLKK
```

Unformatted sequence string: 498 residues (for pasting into other applications).

Sort peptides by ◉ Residue Number ○ Increasing Mass ○ Decreasing Mass

Show predicted peptides also

Query	Start – End	Observed	Mr(expt)	Mr(calc)	ppm	M	Score	Expect	Rank	U	Peptide
13639	3 – 17	779.9015	1557.7884	1557.7886	-0.13	0	83	1.6e-08	1	U	R.INPTTSDPGVSTLEK.K
15264	23 – 39	579.0107	1734.0103	1734.0080	1.35	0	30	0.0019	1	U	R.IAQIIGPVLDVAFPPGK.M
15265	23 – 39	868.0128	1734.0110	1734.0080	1.74	0	55	5.3e-06	1	U	R.IAQIIGPVLDVAFPPGK.M
15266	23 – 39	868.0147	1734.0148	1734.0080	3.90	0	33	0.00091	1	U	R.IAQIIGPVLDVAFPPGK.M
15267	23 – 39	579.0128	1734.0166	1734.0080	4.99	0	34	0.00059	1	U	R.IAQIIGPVLDVAFPPGK.M
9251	40 – 50	638.3617	1274.7088	1274.7056	2.50	0	22	0.0082	1	U	K.MPNIYNALIVK.G
9480	40 – 50	646.3587	1290.7029	1290.7006	1.84	0	40	0.00018	1	U	K.MPNIYNALIVK.G + Oxidation (M)
9481	40 – 50	646.3598	1290.7050	1290.7006	3.42	0	0	0.95	1	U	K.MPNIYNALIVK.G + Oxidation (M)
19170	53 – 73	782.3708	2344.0906	2344.0900	0.28	0	87	7.6e-09	1	U	R.DTAGQPMNVTCEVQQLLGNNR.V
7473	76 – 87	596.8038	1191.5929	1191.5918	0.99	0	46	5.1e-05	1	U	R.AVAMSATDGLTR.G
7474	76 – 87	596.8039	1191.5933	1191.5918	1.28	0	13	0.061	1	U	R.AVAMSATDGLTR.G
7475	76 – 87	596.8045	1191.5944	1191.5918	2.20	0	44	6.9e-05	1	U	R.AVAMSATDGLTR.G
7858	76 – 87	604.8015	1207.5884	1207.5867	1.45	0	23	0.0067	1	U	R.AVAMSATDGLTR.G + Oxidation (M)
7859	76 – 87	604.8016	1207.5887	1207.5867	1.67	0	51	1.5e-05	1	U	R.AVAMSATDGLTR.G + Oxidation (M)
7860	76 – 87	604.8017	1207.5889	1207.5867	1.86	0	2	0.6	1	U	R.AVAMSATDGLTR.G + Oxidation (M)
7861	76 – 87	604.8019	1207.5892	1207.5867	2.10	0	55	6.8e-06	1	U	R.AVAMSATDGLTR.G + Oxidation (M)
17689	88 – 109	1049.0575	2096.1005	2096.0936	3.26	0	68	4.4e-07	1	U	R.GMEVIDTGAPLSVPVGGATLGR.I
17690	88 – 109	699.7075	2096.1006	2096.0936	3.34	0	15	0.04	1	U	R.GMEVIDTGAPLSVPVGGATLGR.I
17756	88 – 109	705.0382	2112.0927	2112.0885	1.96	0	51	1.7e-05	1	U	R.GMEVIDTGAPLSVPVGGATLGR.I + Oxidation (M)

17757	88 – 109	1057.0540	2112.0934	2112.0885	2.33	0	26	0.0034	1	U	R.GMEVIDTGAPLSVPVGGATLGR.I + Oxidation (M)
17758	88 – 109	705.0387	2112.0943	2112.0885	2.71	0	3	0.49	1	U	R.GMEVIDTGAPLSVPVGGATLGR.I + Oxidation (M)
16953	110 – 127	978.0163	1954.0179	1954.0160	0.99	0	113	2.4e-11	1	U	R.IFNVLGEPVDNLGPVDTR.T
16954	110 – 127	652.3476	1954.0209	1954.0160	2.52	0	47	4.2e-05	1	U	R.IFNVLGEPVDNLGPVDTR.T
7140	135 – 145	589.8074	1177.6003	1177.5979	2.00	0	33	0.00075	1	U	R.SAPAFTQLDTK.L
3933	146 – 154	504.2931	1006.5716	1006.5699	1.71	0	16	0.17	1	U	K.LSIFETGIK.V
3934	146 – 154	504.2939	1006.5732	1006.5699	3.32	0	34	0.0024	1	U	K.LSIFETGIK.V
4554	155 – 163	523.3057	1044.5968	1044.5968	0.074	0	55	2e-05	1	U	K.VVDLLAPYR.R
4557	155 – 163	523.3058	1044.5971	1044.5968	0.35	0	41	0.00055	1	U	K.VVDLLAPYR.R
4559	155 – 163	523.3062	1044.5978	1044.5968	0.95	0	44	0.00029	1	U	K.VVDLLAPYR.R
7699	155 – 164	401.2408	1200.7006	1200.6979	2.25	1	26	0.0038	1	U	K.VVDLLAPYRR.G
3514	168 – 178	488.2849	974.5552	974.5549	0.32	0	8	0.16	1		K.IGLFGGAGVGK.T
3515	168 – 178	488.2854	974.5562	974.5549	1.33	0	19	0.016	1		K.IGLFGGAGVGK.T
12510	179 – 191	736.4328	1470.8511	1470.8479	2.11	0	64	1.6e-06	1	U	K.TVLIMELINNIAK.A
12511	179 – 191	491.2914	1470.8524	1470.8479	2.99	0	44	0.00018	1	U	K.TVLIMELINNIAK.A
12751	179 – 191	496.6229	1486.8469	1486.8429	2.74	0	19	0.048	1	U	K.TVLIMELINNIAK.A + Oxidation (M)
12752	179 – 191	744.4320	1486.8494	1486.8429	4.37	0	57	8.8e-06	1	U	K.TVLIMELINNIAK.A + Oxidation (M)
10137	192 – 205	443.5611	1327.6615	1327.6633	-1.41	0	45	6.6e-05	1	U	K.AHGGVSVFGGVGER.T
10138	192 – 205	664.8403	1327.6660	1327.6633	2.05	0	18	0.019	1	U	K.AHGGVSVFGGVGER.T
10139	192 – 205	664.8414	1327.6683	1327.6633	3.72	0	3	0.5	1	U	K.AHGGVSVFGGVGER.T
12729	206 – 217	496.2274	1485.6605	1485.6592	0.89	1	19	0.049	1	U	R.TREGNDLYMEMK.E
12963	206 – 217	751.8354	1501.6562	1501.6541	1.44	1	2	1.6	2	U	R.TREGNDLYMEMK.E + Oxidation (M)
8301	208 – 217	615.2625	1228.5105	1228.5104	0.080	0	10	0.13	1	U	R.EGNDLYMEMK.E
8302	208 – 217	615.2637	1228.5128	1228.5104	1.97	0	8	0.21	1	U	R.EGNDLYMEMK.E
8648	208 – 217	623.2602	1244.5059	1244.5053	0.52	0	11	0.093	1	U	R.EGNDLYMEMK.E + Oxidation (M)
8968	208 – 217	631.2577	1260.5009	1260.5002	0.57	0	7	0.22	1	U	R.EGNDLYMEMK.E + 2 Oxidation (M)
13170	218 – 231	759.3763	1516.7381	1516.7369	0.82	0	56	5.9e-06	1	U	K.ESGVINEQNIAESK.V
13171	218 – 231	759.3770	1516.7394	1516.7369	1.66	0	51	1.7e-05	1	U	K.ESGVINEQNIAESK.V
14036	232 – 246	801.4074	1600.8003	1600.8031	-1.77	0	29	0.0021	1	U	K.VALVYGQMNEPPGAR.M
14037	232 – 246	534.6077	1600.8012	1600.8031	-1.24	0	23	0.0077	1	U	K.VALVYGQMNEPPGAR.M
14038	232 – 246	801.4085	1600.8024	1600.8031	-0.44	0	13	0.058	1	U	K.VALVYGQMNEPPGAR.M
14040	232 – 246	801.4090	1600.8035	1600.8031	0.22	0	58	3.5e-06	1	U	K.VALVYGQMNEPPGAR.M
14041	232 – 246	534.6087	1600.8044	1600.8031	0.77	0	37	0.00036	1	U	K.VALVYGQMNEPPGAR.M
14042	232 – 246	801.4095	1600.8045	1600.8031	0.82	0	77	6.6e-08	1	U	K.VALVYGQMNEPPGAR.M
14043	232 – 246	801.4098	1600.8051	1600.8031	1.22	0	77	6.6e-08	1	U	K.VALVYGQMNEPPGAR.M
14044	232 – 246	801.4099	1600.8052	1600.8031	1.31	0	76	6.7e-08	1	U	K.VALVYGQMNEPPGAR.M
14045	232 – 246	534.6094	1600.8065	1600.8031	2.10	0	10	0.13	1	U	K.VALVYGQMNEPPGAR.M
14046	232 – 246	801.4108	1600.8071	1600.8031	2.49	0	32	0.00092	1	U	K.VALVYGQMNEPPGAR.M
14047	232 – 246	801.4113	1600.8081	1600.8031	3.12	0	26	0.0037	1	U	K.VALVYGQMNEPPGAR.M
14048	232 – 246	801.4126	1600.8106	1600.8031	4.69	0	20	0.012	1	U	K.VALVYGQMNEPPGAR.M
14153	232 – 246	809.4047	1616.7948	1616.7981	-1.99	0	10	0.12	1	U	K.VALVYGQMNEPPGAR.M + Oxidation (M)
14154	232 – 246	809.4056	1616.7966	1616.7981	-0.88	0	7	0.46	1	U	K.VALVYGQMNEPPGAR.M + Oxidation (M)
14155	232 – 246	539.9395	1616.7967	1616.7981	-0.86	0	8	0.19	1	U	K.VALVYGQMNEPPGAR.M + Oxidation (M)
14156	232 – 246	809.4063	1616.7981	1616.7981	0.034	0	11	0.1	1	U	K.VALVYGQMNEPPGAR.M + Oxidation (M)
14157	232 – 246	539.9403	1616.7989	1616.7981	0.54	0	5	0.31	1	U	K.VALVYGQMNEPPGAR.M + Oxidation (M)
14158	232 – 246	539.9408	1616.8007	1616.7981	1.64	0	9	0.15	1	U	K.VALVYGQMNEPPGAR.M + Oxidation (M)
14159	232 – 246	809.4077	1616.8008	1616.7981	1.72	0	4	0.6	1	U	K.VALVYGQMNEPPGAR.M + Oxidation (M)
14160	232 – 246	809.4077	1616.8009	1616.7981	1.75	0	29	0.0027	1	U	K.VALVYGQMNEPPGAR.M + Oxidation (M)
14161	232 – 246	809.4086	1616.8027	1616.7981	2.84	0	12	0.085	1	U	K.VALVYGQMNEPPGAR.M + Oxidation (M)
14162	232 – 246	809.4088	1616.8031	1616.7981	3.12	0	29	0.0019	1	U	K.VALVYGQMNEPPGAR.M + Oxidation (M)
14163	232 – 246	539.9418	1616.8036	1616.7981	3.41	0	12	0.075	1	U	K.VALVYGQMNEPPGAR.M + Oxidation (M)
14164	232 – 246	809.4109	1616.8073	1616.7981	5.70	0	4	0.45	1	U	K.VALVYGQMNEPPGAR.M + Oxidation (M)
12509	249 – 261	736.3861	1470.7576	1470.7541	2.38	0	68	4.6e-07	1	U	R.VGLTALTMAEYFR.D
12748	249 – 261	496.5908	1486.7506	1486.7490	1.06	0	20	0.014	1	U	R.VGLTALTMAEYFR.D + Oxidation (M)
12749	249 – 261	744.3840	1486.7534	1486.7490	2.94	0	61	1.7e-06	1	U	R.VGLTALTMAEYFR.D +

											Oxidation (M)
16929	262 – 277	975.4967	1948.9788	1948.9894	-5.44	0	76	7.8e-08	1	U	R.DVNEQDVLLFIDNIFR.F
11875	278 – 291	478.5971	1432.7695	1432.7674	1.47	0	45	6e-05	1	U	R.FVQAGSEVSALLGR.M
11876	278 – 291	717.3924	1432.7703	1432.7674	2.00	0	49	2.6e-05	1	U	R.FVQAGSEVSALLGR.M
11877	278 – 291	717.3928	1432.7711	1432.7674	2.56	0	94	1.7e-09	1	U	R.FVQAGSEVSALLGR.M
18733	292 – 312	761.3645	2281.0716	2281.0719	-0.12	0	64	1.1e-06	1	U	R.MPSAVGYQPTLSTEMGSLQER.I
18734	292 – 312	1141.5458	2281.0770	2281.0719	2.26	0	14	0.047	1	U	R.MPSAVGYQPTLSTEMGSLQER.I
18735	292 – 312	761.3665	2281.0776	2281.0719	2.53	0	88	6.1e-09	1	U	R.MPSAVGYQPTLSTEMGSLQER.I
18855	292 – 312	1149.5394	2297.0642	2297.0668	-1.10	0	23	0.023	1	U	R.MPSAVGYQPTLSTEMGSLQER.I + Oxidation (M)
18856	292 – 312	766.6980	2297.0722	2297.0668	2.37	0	99	5.2e-10	1	U	R.MPSAVGYQPTLSTEMGSLQER.I + Oxidation (M)
18857	292 – 312	766.6981	2297.0725	2297.0668	2.48	0	46	4.5e-05	1	U	R.MPSAVGYQPTLSTEMGSLQER.I + Oxidation (M)
18858	292 – 312	766.6991	2297.0754	2297.0668	3.74	0	50	2.1e-05	1	U	R.MPSAVGYQPTLSTEMGSLQER.I + Oxidation (M)
18974	292 – 312	772.0284	2313.0634	2313.0617	0.76	0	69	3.7e-07	1	U	R.MPSAVGYQPTLSTEMGSLQER.I + 2 Oxidation (M)
17534	360 – 378	1031.0210	2060.0275	2060.0248	1.29	0	76	7e-08	1	U	K.GIYPAVDPLDSTSTMLQPR.I
17536	360 – 378	1031.0254	2060.0363	2060.0248	5.56	0	2	0.59	1	U	K.GIYPAVDPLDSTSTMLQPR.I
17592	360 – 378	1039.0170	2076.0194	2076.0198	-0.15	0	44	7.7e-05	1	U	K.GIYPAVDPLDSTSTMLQPR.I + Oxidation (M)
17593	360 – 378	693.0158	2076.0255	2076.0198	2.74	0	11	0.1	1	U	K.GIYPAVDPLDSTSTMLQPR.I + Oxidation (M)
12039	379 – 390	722.3656	1442.7166	1442.7154	0.85	0	46	4.4e-05	1	U	R.IVGEEHYEIAQR.V
12042	379 – 390	481.9135	1442.7188	1442.7154	2.38	0	34	0.00066	1	U	R.IVGEEHYEIAQR.V
12043	379 – 390	722.3669	1442.7192	1442.7154	2.69	0	1	0.8	1	U	R.IVGEEHYEIAQR.V
16992	430 – 447	653.3415	1957.0028	1956.9986	2.14	0	41	0.00015	1	U	R.FLSQPFFVAEVFTGSPGK.Y
4153	448 – 456	511.2883	1020.5620	1020.5604	1.55	0	4	0.39	1	U	K.YVGLAETIR.G
4696	487 – 495	526.7415	1051.4684	1051.4678	0.61	0	24	0.016	1	U	K.AMNLEMESK.L

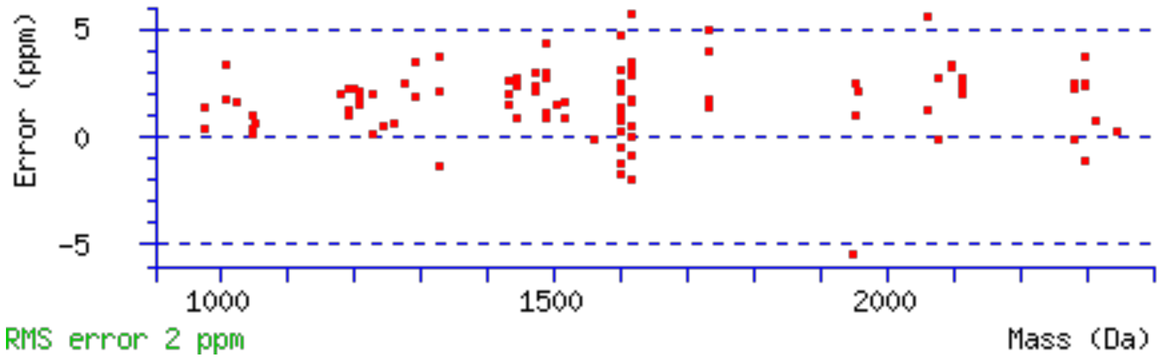

MASCOT Search Results

Protein View: sp|P06512|PSAB_SPIOL

>sp|P06512|PSAB_SPIOL Photosystem I P700 chlorophyll a apoprotein A2 OS=Spinacia oleracea OX=3562 GN=psaB PE=3 SV=1

**Database:**	Uni-Spinach
**Score:**	2006
**Nominal mass (M_r):**	82491
**Calculated pI:**	6.72

Sequence similarity is available as an NCBI BLAST search of sp|P06512|PSAB_SPIOL against nr.

Search parameters

**MS data file:**	\\128.97.66.218\tank\windowsVM\Bill Cramer\MGF\wc_QE_021417_Cramer_Gel_3S_4a.mgf
**Enzyme:**	Trypsin: cuts C-term side of KR unless next residue is P.
**Fixed modifications:**	Carbamidomethyl (C)
**Variable modifications:**	Oxidation (M)

Protein sequence coverage: 24%

Matched peptides shown in ***bold red***.

1 MALRFPR**FSQ** **GLAQDPTTRR** **IWFGIATAHD** **FESHDDITEE** **R**LYQNIFASH
51 FGQLAIIFLW TSGNLFHVAW QGNFESWVQD PLHVRPIAHA IWDPHFGQPA
101 VEAFTRGGAL GPVNIAYSGV YQWWYTIGLR TNEDLYTGAL FLLFLSVISL
151 LGGWLHLQPK **WKPSVSWFKN** **AESR**LNHHLS GLFGVSSLAW TGHLVHVAIP
201 GSRGEYVRWN NFLDVLPHPQ GLGPLFTGQW NLYAQNPDSS SHLFGTSQGA
251 GTAILTLLGG FHPQTQSLWL TDMAHHHLAI AFVFLVAGHM YR**TNFGIGHS**
301 **MKDLLEAHIP** **PGGR**LGRGHK GLYDTINNSL HFQLGLALAS LGVITSLVAQ
351 HMYSLPAYAF IAQDFTTQAA LYTHHQYIAG FIMTGAFAHG AIFFIR**DYNP**
401 **EQNEDNVLAR** MLDHKEAIIS HLSWASLFLG FHTLGLYVHN DVMLAFGTPE
451 K**QILIEPIFA** **QWIQSAHGKT** **SYGFDVLLSS** **TSGPAFNAGR** SIWLPGWLNA
501 VNENSNSLFL TIGPGDFLVH HAIALGLHTT TLILVKGALD ARGSK**LMPDK**
551 **KDFGYSFPCD** **GPGR**GGTCDI SAWDAFYLAV FWMLNTIGWV TFYWHWKHIT
601 LWQGNVSQFN ESSTYLMGWL RDYLWLNSSQ LINGYNPFGM NSLSVWAWMF
651 LFGHLVWATG FMFLISWR**GY** **WQELIETLAW** **AHERTPLANL** **IR**WR**DKPVAL**
701 **SIVQAR**LVGL AHFSVGYIFT YAAFLIASTS GKFG

Unformatted sequence string: 734 residues (for pasting into other applications).

Sort peptides by ◉ Residue Number ○ Increasing Mass ○ Decreasing Mass

Show predicted peptides also

Query	Start – End	Observed	Mr(expt)	Mr(calc)	ppm	M	Score	Expect	Rank	U	Peptide
10017	8 – 19	660.8318	1319.6491	1319.6470	1.60	0	38	0.0003	1	U	R.FSQGLAQDPTTR.R
10018	8 – 19	660.8320	1319.6494	1319.6470	1.82	0	34	0.00059	1	U	R.FSQGLAQDPTTR.R
10019	8 – 19	660.8322	1319.6498	1319.6470	2.14	0	25	0.0042	1	U	R.FSQGLAQDPTTR.R
10020	8 – 19	660.8322	1319.6499	1319.6470	2.20	0	38	0.00067	1	U	R.FSQGLAQDPTTR.R
10021	8 – 19	440.8907	1319.6503	1319.6470	2.50	0	7	0.22	1	U	R.FSQGLAQDPTTR.R
12573	8 – 20	492.9237	1475.7493	1475.7481	0.79	1	20	0.048	1	U	R.FSQGLAQDPTTRR.I
12574	8 – 20	738.8825	1475.7504	1475.7481	1.60	1	11	0.16	1	U	R.FSQGLAQDPTTRR.I
20340	20 – 41	882.4166	2644.2280	2644.2306	-0.98	1	9	0.16	1	U	R.RIWFGIATAHDFESHDDITEER.L
20342	20 – 41	662.0653	2644.2322	2644.2306	0.60	1	72	1.9e-07	1	U	R.RIWFGIATAHDFESHDDITEER.L
20343	20 – 41	662.0655	2644.2328	2644.2306	0.81	1	38	0.00025	1	U	R.RIWFGIATAHDFESHDDITEER.L
20344	20 – 41	662.0656	2644.2333	2644.2306	1.01	1	19	0.016	1	U	R.RIWFGIATAHDFESHDDITEER.L
20347	20 – 41	529.8547	2644.2371	2644.2306	2.46	1	2	0.58	1	U	R.RIWFGIATAHDFESHDDITEER.L
20348	20 – 41	662.0667	2644.2377	2644.2306	2.66	1	57	4.9e-06	1	U	R.RIWFGIATAHDFESHDDITEER.L
20349	20 – 41	529.8549	2644.2380	2644.2306	2.80	1	14	0.044	1	U	R.RIWFGIATAHDFESHDDITEER.L
20350	20 – 41	662.0668	2644.2383	2644.2306	2.90	1	11	0.084	1	U	R.RIWFGIATAHDFESHDDITEER.L
20351	20 – 41	662.0670	2644.2390	2644.2306	3.18	1	50	2.1e-05	1	U	R.RIWFGIATAHDFESHDDITEER.L
19767	21 – 41	830.3845	2488.1316	2488.1295	0.86	0	66	1.2e-06	1	U	R.IWFGIATAHDFESHDDITEER.L
19768	21 – 41	623.0405	2488.1330	2488.1295	1.39	0	32	0.0031	1	U	R.IWFGIATAHDFESHDDITEER.L
19769	21 – 41	830.3851	2488.1335	2488.1295	1.61	0	69	6.2e-07	1	U	R.IWFGIATAHDFESHDDITEER.L
19770	21 – 41	830.3851	2488.1336	2488.1295	1.65	0	44	0.00019	1	U	R.IWFGIATAHDFESHDDITEER.L

19771	21 – 41	830.3856	2488.1350	2488.1295	2.19	0	20	0.046	1	U	R.IWFGIATAHDFESHDDITEER.L
19772	21 – 41	623.0411	2488.1353	2488.1295	2.31	0	95	1.4e-09	1	U	R.IWFGIATAHDFESHDDITEER.L
19773	21 – 41	623.0412	2488.1356	2488.1295	2.43	0	51	3.9e-05	1	U	R.IWFGIATAHDFESHDDITEER.L
19774	21 – 41	623.0412	2488.1359	2488.1295	2.56	0	78	7e-08	1	U	R.IWFGIATAHDFESHDDITEER.L
19775	21 – 41	623.0413	2488.1361	2488.1295	2.65	0	38	0.00074	1	U	R.IWFGIATAHDFESHDDITEER.L
19776	21 – 41	623.0414	2488.1363	2488.1295	2.74	0	67	9.1e-07	1	U	R.IWFGIATAHDFESHDDITEER.L
19777	21 – 41	623.0414	2488.1366	2488.1295	2.85	0	79	6.9e-08	1	U	R.IWFGIATAHDFESHDDITEER.L
19778	21 – 41	623.0417	2488.1376	2488.1295	3.26	0	40	0.00055	1	U	R.IWFGIATAHDFESHDDITEER.L
6879	161 – 169	388.8787	1163.6142	1163.6128	1.19	0	11	0.097	1	U	K.WKPSVSWFK.N
6881	161 – 169	582.8146	1163.6146	1163.6128	1.60	0	32	0.0011	1	U	K.WKPSVSWFK.N
6882	161 – 169	388.8789	1163.6149	1163.6128	1.82	0	0	0.97	1	U	K.WKPSVSWFK.N
6883	161 – 169	582.8148	1163.6151	1163.6128	2.01	0	32	0.0011	1	U	K.WKPSVSWFK.N
6884	161 – 169	388.8790	1163.6153	1163.6128	2.15	0	8	0.2	1	U	K.WKPSVSWFK.N
6885	161 – 169	388.8791	1163.6155	1163.6128	2.40	0	12	0.079	1	U	K.WKPSVSWFK.N
15172	161 – 174	574.6322	1720.8747	1720.8685	3.59	1	10	0.12	1	U	K.WKPSVSWFKNAESR.L
5469	293 – 302	546.2695	1090.5244	1090.5230	1.33	0	44	7.3e-05	1	U	R.TNFGIGHSMK.D
5471	293 – 302	364.5157	1090.5253	1090.5230	2.14	0	9	0.16	1	U	R.TNFGIGHSMK.D
5805	293 – 302	554.2661	1106.5177	1106.5179	-0.15	0	31	0.0014	1	U	R.TNFGIGHSMK.D + Oxidation (M)
19183	293 – 314	587.5558	2346.1941	2346.1903	1.63	1	5	0.37	1	U	R.TNFGIGHSMKDLLEAHIPPGGR.L
19185	293 – 314	587.5571	2346.1995	2346.1903	3.91	1	1	0.88	1	U	R.TNFGIGHSMKDLLEAHIPPGGR.L
19270	293 – 314	591.5536	2362.1853	2362.1852	0.042	1	17	0.037	1	U	R.TNFGIGHSMKDLLEAHIPPGGR.L + Oxidation (M)
9179	303 – 314	637.8461	1273.6776	1273.6779	-0.19	0	35	0.00053	1	U	K.DLLEAHIPPGGR.L
9180	303 – 314	637.8461	1273.6777	1273.6779	-0.15	0	32	0.001	1	U	K.DLLEAHIPPGGR.L
9181	303 – 314	425.5665	1273.6778	1273.6779	-0.079	0	6	0.29	1	U	K.DLLEAHIPPGGR.L
9182	303 – 314	425.5666	1273.6778	1273.6779	-0.027	0	10	0.11	1	U	K.DLLEAHIPPGGR.L
9183	303 – 314	425.5666	1273.6780	1273.6779	0.11	0	24	0.0059	1	U	K.DLLEAHIPPGGR.L
9184	303 – 314	637.8464	1273.6782	1273.6779	0.25	0	35	0.00056	1	U	K.DLLEAHIPPGGR.L
9185	303 – 314	637.8464	1273.6782	1273.6779	0.29	0	30	0.0015	1	U	K.DLLEAHIPPGGR.L
9186	303 – 314	425.5667	1273.6784	1273.6779	0.42	0	21	0.01	1	U	K.DLLEAHIPPGGR.L
9187	303 – 314	637.8466	1273.6786	1273.6779	0.59	0	34	0.00061	1	U	K.DLLEAHIPPGGR.L
9188	303 – 314	425.5668	1273.6786	1273.6779	0.59	0	9	0.15	1	U	K.DLLEAHIPPGGR.L
9189	303 – 314	425.5669	1273.6788	1273.6779	0.70	0	10	0.11	1	U	K.DLLEAHIPPGGR.L
9190	303 – 314	425.5669	1273.6788	1273.6779	0.73	0	9	0.16	1	U	K.DLLEAHIPPGGR.L
9191	303 – 314	425.5669	1273.6788	1273.6779	0.74	0	0	1	2	U	K.DLLEAHIPPGGR.L
9192	303 – 314	637.8467	1273.6788	1273.6779	0.76	0	15	0.037	1	U	K.DLLEAHIPPGGR.L
9193	303 – 314	637.8468	1273.6790	1273.6779	0.89	0	39	0.00021	1	U	K.DLLEAHIPPGGR.L
9194	303 – 314	637.8468	1273.6790	1273.6779	0.89	0	40	0.00018	1	U	K.DLLEAHIPPGGR.L
9195	303 – 314	637.8468	1273.6790	1273.6779	0.91	0	33	0.00084	1	U	K.DLLEAHIPPGGR.L
9196	303 – 314	425.5670	1273.6791	1273.6779	0.94	0	12	0.069	1	U	K.DLLEAHIPPGGR.L
9197	303 – 314	425.5670	1273.6791	1273.6779	0.96	0	11	0.1	1	U	K.DLLEAHIPPGGR.L
9198	303 – 314	637.8468	1273.6791	1273.6779	1.00	0	28	0.0023	1	U	K.DLLEAHIPPGGR.L
9199	303 – 314	425.5670	1273.6792	1273.6779	1.02	0	24	0.0055	1	U	K.DLLEAHIPPGGR.L
9200	303 – 314	425.5670	1273.6792	1273.6779	1.07	0	10	0.13	1	U	K.DLLEAHIPPGGR.L
9201	303 – 314	425.5670	1273.6793	1273.6779	1.13	0	20	0.012	1	U	K.DLLEAHIPPGGR.L
9202	303 – 314	425.5671	1273.6793	1273.6779	1.14	0	6	0.29	1	U	K.DLLEAHIPPGGR.L
9203	303 – 314	637.8470	1273.6795	1273.6779	1.25	0	1	0.77	1	U	K.DLLEAHIPPGGR.L
9204	303 – 314	637.8470	1273.6795	1273.6779	1.27	0	38	0.00028	1	U	K.DLLEAHIPPGGR.L
9205	303 – 314	637.8470	1273.6795	1273.6779	1.28	0	39	0.00024	1	U	K.DLLEAHIPPGGR.L
9206	303 – 314	637.8470	1273.6795	1273.6779	1.28	0	52	1.5e-05	1	U	K.DLLEAHIPPGGR.L
9207	303 – 314	637.8471	1273.6796	1273.6779	1.33	0	48	2.9e-05	1	U	K.DLLEAHIPPGGR.L
9208	303 – 314	637.8471	1273.6796	1273.6779	1.36	0	61	1.7e-06	1	U	K.DLLEAHIPPGGR.L
9209	303 – 314	425.5672	1273.6797	1273.6779	1.45	0	19	0.017	1	U	K.DLLEAHIPPGGR.L
9210	303 – 314	637.8472	1273.6798	1273.6779	1.49	0	28	0.0021	1	U	K.DLLEAHIPPGGR.L
9211	303 – 314	425.5672	1273.6798	1273.6779	1.51	0	12	0.071	1	U	K.DLLEAHIPPGGR.L
9212	303 – 314	425.5672	1273.6798	1273.6779	1.53	0	6	0.28	1	U	K.DLLEAHIPPGGR.L
9213	303 – 314	425.5672	1273.6798	1273.6779	1.54	0	11	0.098	1	U	K.DLLEAHIPPGGR.L
9214	303 – 314	425.5672	1273.6799	1273.6779	1.60	0	25	0.0045	1	U	K.DLLEAHIPPGGR.L
9216	303 – 314	637.8473	1273.6800	1273.6779	1.64	0	17	0.025	1	U	K.DLLEAHIPPGGR.L
9217	303 – 314	637.8473	1273.6801	1273.6779	1.75	0	28	0.0025	1	U	K.DLLEAHIPPGGR.L
9218	303 – 314	425.5673	1273.6801	1273.6779	1.76	0	13	0.066	1	U	K.DLLEAHIPPGGR.L
9219	303 – 314	637.8473	1273.6801	1273.6779	1.78	0	19	0.016	1	U	K.DLLEAHIPPGGR.L
9220	303 – 314	637.8473	1273.6801	1273.6779	1.78	0	2	0.68	1	U	K.DLLEAHIPPGGR.L
9221	303 – 314	425.5674	1273.6803	1273.6779	1.88	0	31	0.0011	1	U	K.DLLEAHIPPGGR.L
9222	303 – 314	425.5674	1273.6804	1273.6779	1.97	0	14	0.051	1	U	K.DLLEAHIPPGGR.L
9223	303 – 314	637.8475	1273.6804	1273.6779	1.97	0	35	0.00055	1	U	K.DLLEAHIPPGGR.L
9224	303 – 314	425.5675	1273.6806	1273.6779	2.12	0	12	0.079	1	U	K.DLLEAHIPPGGR.L
9225	303 – 314	637.8476	1273.6806	1273.6779	2.14	0	44	7.7e-05	1	U	K.DLLEAHIPPGGR.L
9226	303 – 314	637.8476	1273.6806	1273.6779	2.18	0	38	0.00033	1	U	K.DLLEAHIPPGGR.L
9227	303 – 314	637.8476	1273.6807	1273.6779	2.25	0	0	0.98	1	U	K.DLLEAHIPPGGR.L
9228	303 – 314	637.8477	1273.6808	1273.6779	2.30	0	52	1.3e-05	1	U	K.DLLEAHIPPGGR.L
9229	303 – 314	425.5675	1273.6808	1273.6779	2.31	0	19	0.018	1	U	K.DLLEAHIPPGGR.L

9230	303 - 314	637.8477	1273.6808	1273.6779	2.34	0	24	0.0061	1	U	K.DLLEAHIPPGGR.L
9231	303 - 314	637.8477	1273.6808	1273.6779	2.34	0	35	0.00057	1	U	K.DLLEAHIPPGGR.L
9232	303 - 314	425.5676	1273.6809	1273.6779	2.36	0	21	0.011	1	U	K.DLLEAHIPPGGR.L
9233	303 - 314	425.5676	1273.6809	1273.6779	2.37	0	27	0.0032	1	U	K.DLLEAHIPPGGR.L
9234	303 - 314	425.5676	1273.6810	1273.6779	2.49	0	10	0.12	1	U	K.DLLEAHIPPGGR.L
9235	303 - 314	425.5676	1273.6811	1273.6779	2.53	0	32	0.0011	1	U	K.DLLEAHIPPGGR.L
9237	303 - 314	637.8480	1273.6815	1273.6779	2.83	0	38	0.00028	1	U	K.DLLEAHIPPGGR.L
9238	303 - 314	637.8481	1273.6816	1273.6779	2.92	0	39	0.00024	1	U	K.DLLEAHIPPGGR.L
9239	303 - 314	425.5679	1273.6820	1273.6779	3.22	0	35	0.00057	1	U	K.DLLEAHIPPGGR.L
9240	303 - 314	425.5680	1273.6821	1273.6779	3.29	0	16	0.032	1	U	K.DLLEAHIPPGGR.L
9241	303 - 314	425.5680	1273.6821	1273.6779	3.35	0	14	0.053	1	U	K.DLLEAHIPPGGR.L
14734	397 - 410	838.8795	1675.7445	1675.7438	0.46	0	54	1.6e-05	1	U	R.DYNPEQNEDNVLAR.M
14735	397 - 410	838.8812	1675.7479	1675.7438	2.49	0	75	1.3e-07	1	U	R.DYNPEQNEDNVLAR.M
14736	397 - 410	838.8815	1675.7484	1675.7438	2.74	0	8	0.61	1	U	R.DYNPEQNEDNVLAR.M
14737	397 - 410	559.5903	1675.7492	1675.7438	3.23	0	57	7.6e-06	1	U	R.DYNPEQNEDNVLAR.M
14738	397 - 410	559.5906	1675.7500	1675.7438	3.75	0	61	3.6e-06	1	U	R.DYNPEQNEDNVLAR.M
17598	452 - 469	693.7197	2078.1374	2078.1313	2.95	0	26	0.013	1	U	K.QILIEPIFAQWIQSAHGK.T
17599	452 - 469	693.7199	2078.1379	2078.1313	3.17	0	55	1.3e-05	1	U	K.QILIEPIFAQWIQSAHGK.T
17600	452 - 469	1040.0776	2078.1407	2078.1313	4.53	0	64	1.7e-06	1	U	K.QILIEPIFAQWIQSAHGK.T
17929	470 - 490	1074.0240	2146.0335	2146.0331	0.18	0	100	4.3e-10	1	U	K.TSYGFDVLLSSTSGPAFNAGR.S
17930	470 - 490	716.3537	2146.0394	2146.0331	2.91	0	68	4.3e-07	1	U	K.TSYGFDVLLSSTSGPAFNAGR.S
289	546 - 551	366.2101	730.4056	730.4047	1.24	1	0	0.96	1	U	K.LMPDKK.D
14050	551 - 564	534.9041	1601.6904	1601.6933	-1.81	1	14	0.09	1	U	K.KDFGYSFPCDGPGR.G
12530	552 - 564	737.8073	1473.6001	1473.5983	1.16	0	62	6.5e-07	1	U	K.DFGYSFPCDGPGR.G
12532	552 - 564	737.8083	1473.6020	1473.5983	2.51	0	17	0.02	1	U	K.DFGYSFPCDGPGR.G
17263	669 - 684	668.0006	2000.9800	2000.9744	2.79	0	18	0.02	1	U	R.GYWQELIETLAWAHER.T
2381	685 - 692	449.2796	896.5446	896.5443	0.32	0	11	0.13	1	U	R.TPLANLIR.W
2383	685 - 692	449.2800	896.5454	896.5443	1.23	0	2	1.2	1	U	R.TPLANLIR.W
2384	685 - 692	449.2804	896.5462	896.5443	2.09	0	12	0.12	1	U	R.TPLANLIR.W
9561	695 - 706	648.8856	1295.7566	1295.7561	0.37	0	50	3.7e-05	1	U	R.DKPVALSIVQAR.L
9562	695 - 706	648.8861	1295.7576	1295.7561	1.15	0	56	9.5e-06	1	U	R.DKPVALSIVQAR.L
9563	695 - 706	432.9266	1295.7580	1295.7561	1.43	0	28	0.0052	1	U	R.DKPVALSIVQAR.L
9564	695 - 706	432.9268	1295.7586	1295.7561	1.91	0	25	0.012	1	U	R.DKPVALSIVQAR.L

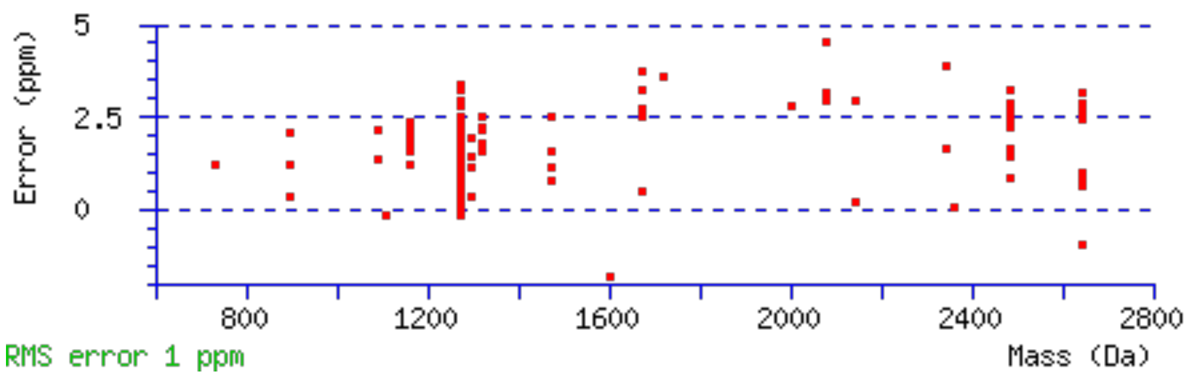

Mascot: http://www.matrixscience.com/

MASCOT Search Results

Protein View: tr|A0A0K9R886|A0A0K9R886_SPIOL

>tr|A0A0K9R886|A0A0K9R886_SPIOL Uncharacterized protein OS=Spinacia oleracea OX=3562 GN=SOVF_102250 PE=4 SV=1

**Database:**	Uni-Spinach
**Score:**	1343
**Nominal mass (M_r):**	23145
**Calculated pI:**	9.76

Sequence similarity is available as an NCBI BLAST search of tr|A0A0K9R886|A0A0K9R886_SPIOL against nr.

Search parameters

**MS data file:**	\\128.97.66.218\tank\windowsVM\Bill Cramer\MGF\wc_QE_021417_Cramer_Gel_3S_4a.mgf
**Enzyme:**	Trypsin: cuts C-term side of KR unless next residue is P.
**Fixed modifications:**	Carbamidomethyl (C)
**Variable modifications:**	Oxidation (M)

Protein sequence coverage: 58%

Matched peptides shown in ***bold red***.

```
  1 MAMATQATLF SPSSLSSAKP IDTRLTTSFK QPSAVTFASK PASRHHSIRA
 51 AAAAEGKAAA ATETKEAPKG FTPPELDPNT PSPIFAGSTG GLLRKAQVEE
101 FYVITWESPK EQIFEMPTGG AAIMREGPNL LKLARKEQCL ALGTRLRSKY
151 KIKYQFYRVF PSGEVQYLHP KDGVYPEKVN PGRQGVGLNM RSIGKNVSPI
201 EVKFTGKQPY DL
```

Unformatted sequence string: 212 residues (for pasting into other applications).

Sort peptides by ◉ Residue Number ○ Increasing Mass ○ Decreasing Mass

Show predicted peptides also

Query	Start – End	Observed	Mr(expt)	Mr(calc)	ppm	M	Score	Expect	Rank	U	Peptide
19969	70 – 94	847.7727	2540.2962	2540.2911	1.98	0	80	2.9e-08	1	U	K.GFTPPELDPNTPSPIFAGSTGGLLR.K
19970	70 – 94	1271.1559	2540.2972	2540.2911	2.40	0	67	4.8e-07	1	U	K.GFTPPELDPNTPSPIFAGSTGGLLR.K
19971	70 – 94	636.0819	2540.2983	2540.2911	2.84	0	66	6.5e-07	1	U	K.GFTPPELDPNTPSPIFAGSTGGLLR.K
20490	70 – 95	890.4710	2668.3911	2668.3861	1.88	1	28	0.0022	1	U	K.GFTPPELDPNTPSPIFAGSTGGLLRK.A
20491	70 – 95	890.4721	2668.3946	2668.3861	3.19	1	11	0.087	1	U	K.GFTPPELDPNTPSPIFAGSTGGLLRK.A
16938	95 – 110	652.0029	1952.9868	1952.9884	-0.80	1	4	0.43	1	U	R.KAQVEEFYVITWESPK.E
16941	95 – 110	977.5028	1952.9911	1952.9884	1.39	1	70	2.7e-07	1	U	R.KAQVEEFYVITWESPK.E
16942	95 – 110	652.0047	1952.9924	1952.9884	2.07	1	26	0.0037	1	U	R.KAQVEEFYVITWESPK.E
16943	95 – 110	652.0049	1952.9929	1952.9884	2.31	1	52	1.2e-05	1	U	R.KAQVEEFYVITWESPK.E
16944	95 – 110	977.5038	1952.9931	1952.9884	2.45	1	59	2.8e-06	1	U	R.KAQVEEFYVITWESPK.E
16945	95 – 110	652.0050	1952.9931	1952.9884	2.45	1	42	0.00011	1	U	R.KAQVEEFYVITWESPK.E
16946	95 – 110	652.0054	1952.9944	1952.9884	3.08	1	11	0.087	1	U	R.KAQVEEFYVITWESPK.E
16947	95 – 110	652.0062	1952.9966	1952.9884	4.23	1	54	7.9e-06	1	U	R.KAQVEEFYVITWESPK.E
15979	96 – 110	609.3055	1824.8947	1824.8934	0.72	0	67	5.5e-07	1	U	K.AQVEEFYVITWESPK.E
15980	96 – 110	913.4551	1824.8956	1824.8934	1.20	0	107	1e-10	1	U	K.AQVEEFYVITWESPK.E
14488	111 – 125	825.9038	1649.7930	1649.7905	1.50	0	18	0.019	1	U	K.EQIFEMPTGGAAIMR.E
14489	111 – 125	550.9390	1649.7951	1649.7905	2.77	0	51	1.7e-05	1	U	K.EQIFEMPTGGAAIMR.E
14490	111 – 125	825.9056	1649.7966	1649.7905	3.67	0	82	3.4e-08	1	U	K.EQIFEMPTGGAAIMR.E
14639	111 – 125	833.8998	1665.7850	1665.7854	-0.29	0	18	0.018	1	U	K.EQIFEMPTGGAAIMR.E + Oxidation (M)
14640	111 – 125	833.8999	1665.7853	1665.7854	-0.097	0	57	4.1e-06	1	U	K.EQIFEMPTGGAAIMR.E + Oxidation (M)
14642	111 – 125	833.9003	1665.7860	1665.7854	0.35	0	81	2.6e-08	1	U	K.EQIFEMPTGGAAIMR.E + Oxidation (M)
14644	111 – 125	833.9010	1665.7875	1665.7854	1.26	0	43	0.0001	1	U	K.EQIFEMPTGGAAIMR.E + Oxidation (M)
14645	111 – 125	556.2700	1665.7882	1665.7854	1.66	0	40	0.00018	1	U	K.EQIFEMPTGGAAIMR.E + Oxidation (M)
14646	111 – 125	556.2701	1665.7884	1665.7854	1.77	0	28	0.0026	1	U	K.EQIFEMPTGGAAIMR.E + Oxidation (M)
14649	111 – 125	556.2704	1665.7895	1665.7854	2.44	0	8	0.2	1	U	K.EQIFEMPTGGAAIMR.E + Oxidation (M)
14793	111 – 125	841.8975	1681.7804	1681.7804	0.049	0	9	0.14	1	U	K.EQIFEMPTGGAAIMR.E + 2 Oxidation (M)

14794	111 – 125	841.8975	1681.7804	1681.7804	0.051	0	47	3.9e-05	1	U	K.EQIFEMPTGGAAIMR.E + 2 Oxidation (M)
14795	111 – 125	561.6009	1681.7809	1681.7804	0.34	0	37	0.00037	1	U	K.EQIFEMPTGGAAIMR.E + 2 Oxidation (M)
14796	111 – 125	841.8986	1681.7826	1681.7804	1.36	0	0	0.99	1	U	K.EQIFEMPTGGAAIMR.E + 2 Oxidation (M)
14797	111 – 125	561.6016	1681.7831	1681.7804	1.63	0	13	0.067	1	U	K.EQIFEMPTGGAAIMR.E + 2 Oxidation (M)
14798	111 – 125	841.8994	1681.7842	1681.7804	2.31	0	19	0.017	1	U	K.EQIFEMPTGGAAIMR.E + 2 Oxidation (M)
663	126 – 132	385.7243	769.4341	769.4334	0.96	0	10	0.52	1	U	R.EGPNLLK.L
665	126 – 132	385.7246	769.4346	769.4334	1.61	0	9	0.5	1	U	R.EGPNLLK.L
7098	136 – 145	392.5447	1174.6121	1174.6128	-0.58	1	27	0.0047	1	U	R.KEQCLALGTR.L
7099	136 – 145	392.5453	1174.6139	1174.6128	0.95	1	31	0.0013	1	U	R.KEQCLALGTR.L
7100	136 – 145	588.3143	1174.6140	1174.6128	1.01	1	58	1.2e-05	1	U	R.KEQCLALGTR.L
4607	137 – 145	524.2667	1046.5189	1046.5179	1.03	0	13	0.063	1	U	K.EQCLALGTR.L
4608	137 – 145	524.2674	1046.5202	1046.5179	2.26	0	20	0.012	1	U	K.EQCLALGTR.L
4086	152 – 158	509.2784	1016.5423	1016.5443	-1.99	1	13	0.42	1	U	K.IKYQFYR.V
719	154 – 158	388.6893	775.3640	775.3653	-1.63	0	14	0.072	1	U	K.YQFYR.V
720	154 – 158	388.6900	775.3654	775.3653	0.079	0	10	0.45	1	U	K.YQFYR.V
721	154 – 158	388.6900	775.3655	775.3653	0.28	0	18	0.053	1	U	K.YQFYR.V
12927	159 – 171	750.8961	1499.7777	1499.7773	0.29	0	40	0.00016	1	U	R.VFPSGEVQYLHPK.D
12928	159 – 171	750.8963	1499.7779	1499.7773	0.46	0	44	8.2e-05	1	U	R.VFPSGEVQYLHPK.D
12929	159 – 171	750.8963	1499.7780	1499.7773	0.52	0	40	0.00017	1	U	R.VFPSGEVQYLHPK.D
12930	159 – 171	750.8963	1499.7781	1499.7773	0.52	0	49	2.6e-05	1	U	R.VFPSGEVQYLHPK.D
12931	159 – 171	750.8965	1499.7784	1499.7773	0.74	0	39	0.00023	1	U	R.VFPSGEVQYLHPK.D
12932	159 – 171	750.8965	1499.7784	1499.7773	0.78	0	46	4.5e-05	1	U	R.VFPSGEVQYLHPK.D
12933	159 – 171	750.8965	1499.7785	1499.7773	0.83	0	29	0.0019	1	U	R.VFPSGEVQYLHPK.D
12934	159 – 171	750.8966	1499.7787	1499.7773	0.94	0	45	5.7e-05	1	U	R.VFPSGEVQYLHPK.D
12935	159 – 171	500.9336	1499.7790	1499.7773	1.18	0	25	0.005	1	U	R.VFPSGEVQYLHPK.D
12936	159 – 171	750.8971	1499.7797	1499.7773	1.62	0	40	0.00018	1	U	R.VFPSGEVQYLHPK.D
12937	159 – 171	500.9339	1499.7799	1499.7773	1.78	0	26	0.0034	1	U	R.VFPSGEVQYLHPK.D
12938	159 – 171	500.9340	1499.7800	1499.7773	1.84	0	28	0.0023	1	U	R.VFPSGEVQYLHPK.D
12939	159 – 171	500.9340	1499.7803	1499.7773	2.00	0	27	0.0027	1	U	R.VFPSGEVQYLHPK.D
12941	159 – 171	500.9343	1499.7812	1499.7773	2.63	0	24	0.0058	1	U	R.VFPSGEVQYLHPK.D
12942	159 – 171	500.9345	1499.7817	1499.7773	2.97	0	43	8.5e-05	1	U	R.VFPSGEVQYLHPK.D
12943	159 – 171	500.9345	1499.7818	1499.7773	3.00	0	34	0.00068	1	U	R.VFPSGEVQYLHPK.D
12944	159 – 171	500.9346	1499.7819	1499.7773	3.07	0	23	0.0076	1	U	R.VFPSGEVQYLHPK.D
12945	159 – 171	500.9346	1499.7820	1499.7773	3.16	0	46	4.7e-05	1	U	R.VFPSGEVQYLHPK.D
1053	172 – 178	404.1976	806.3807	806.3810	-0.40	0	9	0.14	1	U	K.DGVYPEK.V
1056	172 – 178	404.1980	806.3815	806.3810	0.54	0	5	0.31	1	U	K.DGVYPEK.V
1057	172 – 178	404.1980	806.3815	806.3810	0.54	0	11	0.089	1	U	K.DGVYPEK.V
1059	172 – 178	404.1982	806.3819	806.3810	1.08	0	7	0.22	1	U	K.DGVYPEK.V
10166	172 – 183	444.2304	1329.6694	1329.6677	1.25	1	25	0.0046	1	U	K.DGVYPEKVNPGR.Q
2129	184 – 191	437.7324	873.4503	873.4491	1.43	0	8	0.2	1	U	R.QGVGLNMR.S
9124	192 – 203	424.2501	1269.7285	1269.7292	-0.60	1	15	0.039	1	U	R.SIGKNVSPIEVK.F
9126	192 – 203	424.2512	1269.7317	1269.7292	1.94	1	12	0.11	1	U	R.SIGKNVSPIEVK.F

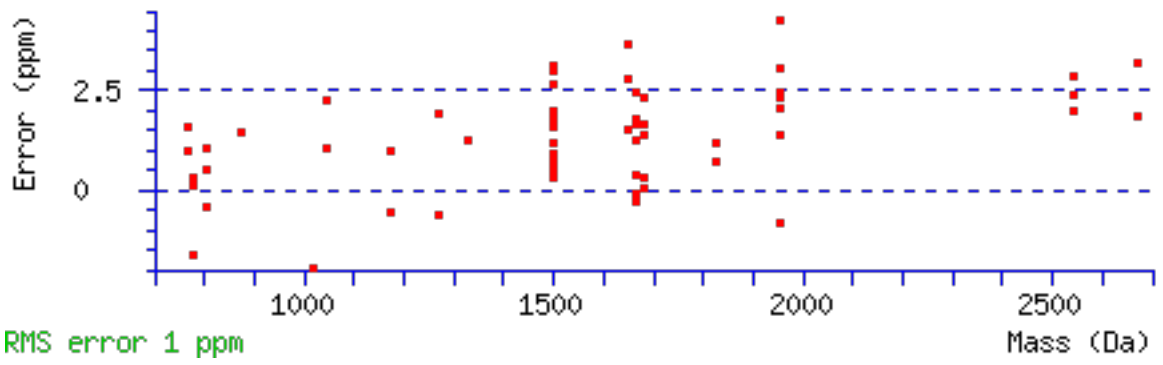

MASCOT Search Results

Protein View: sp|P06450|ATPA_SPIOL

>sp|P06450|ATPA_SPIOL ATP synthase subunit alpha, chloroplastic OS=Spinacia oleracea OX=3562 GN=atpA PE=1 SV=1

**Database:**	Uni-Spinach
**Score:**	861
**Nominal mass (M_r):**	55474
**Calculated pI:**	5.16

Sequence similarity is available as **an NCBI BLAST search of sp|P06450|ATPA_SPIOL against nr**.

Search parameters

**MS data file:**	\\128.97.66.218\tank\windowsVM\Bill Cramer\MGF\wc_QE_021417_Cramer_Gel_3S_4a.mgf
**Enzyme:**	Trypsin: cuts C-term side of KR unless next residue is P.
**Fixed modifications:**	**Carbamidomethyl (C)**
**Variable modifications:**	**Oxidation (M)**

Protein sequence coverage: 42%

Matched peptides shown in ***bold red***.

1 MATIRADEIS KIIR**ERIEGY** **NR**EVK**VVNTG** **TVLQVGDGIA** **R**IHGLDEVMA
51 GELVEFEEGT IGIALNLESN NVGVVLMGDG LMIQEGSSVK ATGR**IAQIPV**
101 **SEAYLGRVIN** **ALAKPIDGRG** **EITASESR**LI ESPAPGIMSR R**SVYEPLQTG**
151 **LIAIDAMIPV** **GR**GQR**ELIIG** **DR**QTGK**TAVA** **TDTILNQQGQ** **NVICVYVAIG**
201 **QKASSVAQVV** **TNFQER**GAME YTIVVAETAD SPATLQYLAP YTGAALAEYF
251 MYR**ERHTLII** **YDDLSK**QAQA YR**QMSLLLR**R PPGR**EAYPGD** **VFYLHSR**LLE
301 RAAKLSSLLG EGSMTALPIV ETQAGDVSAY IPTNVISITD GQIFLSADLF
351 NAGIRPAINV GISVSR**VGSA** **AQIK**AMKKVA GKLKLELAQF AELEAFAQFA
401 SDLDK**ATQNQ** **LAR**GQRLREL LKQPQSAPLT VEEQVMTIYT GTNGYLDSLE
451 LDQVR**KYLVE** **LR**TYVK**TNKP** **EFQEIISSTK** **TFTEEAEALL** **KEAIQEQMER**
501 FLLQEQA

Unformatted sequence string: **507 residues** (for pasting into other applications).

Sort peptides by ◉ Residue Number ○ Increasing Mass ○ Decreasing Mass

Show predicted peptides also

Query	Start – End	Observed	Mr(expt)	Mr(calc)	ppm	M	Score	Expect	Rank	U	Peptide
4394	15 – 22	518.7631	1035.5117	1035.5097	1.89	1	0	0.96	1		R.ERIEGYNR.E
389	17 – 22	376.1900	750.3655	750.3660	-0.69	0	3	0.53	1		R.IEGYNR.E
390	17 – 22	376.1903	750.3661	750.3660	0.048	0	10	0.65	1		R.IEGYNR.E
392	17 – 22	376.1909	750.3673	750.3660	1.63	0	1	0.73	1		R.IEGYNR.E
14001	26 – 41	799.9483	1597.8820	1597.8788	2.02	0	82	2.3e-08	1		K.VVNTGTVLQVGDGIAR.I
14002	26 – 41	533.6346	1597.8821	1597.8788	2.05	0	38	0.00025	1		K.VVNTGTVLQVGDGIAR.I
14003	26 – 41	533.6355	1597.8847	1597.8788	3.68	0	3	0.5	1		K.VVNTGTVLQVGDGIAR.I
11582	95 – 107	708.8968	1415.7791	1415.7772	1.30	0	39	0.00022	1		R.IAQIPVSEAYLGR.V
11583	95 – 107	708.8972	1415.7798	1415.7772	1.79	0	50	2.2e-05	1		R.IAQIPVSEAYLGR.V
9075	108 – 119	422.9224	1265.7454	1265.7455	-0.098	0	27	0.0067	1		R.VINALAKPIDGR.G
3132	120 – 128	475.2327	948.4509	948.4512	-0.33	0	14	0.053	1		R.GEITASESR.L
3133	120 – 128	475.2330	948.4514	948.4512	0.19	0	27	0.0032	1		R.GEITASESR.L
3135	120 – 128	475.2331	948.4516	948.4512	0.39	0	17	0.027	1		R.GEITASESR.L
3136	120 – 128	475.2331	948.4516	948.4512	0.43	0	21	0.011	1		R.GEITASESR.L
3137	120 – 128	475.2331	948.4516	948.4512	0.45	0	16	0.029	1		R.GEITASESR.L
3138	120 – 128	475.2332	948.4519	948.4512	0.74	0	13	0.056	1		R.GEITASESR.L
18439	142 – 162	1122.1104	2242.2063	2242.2031	1.43	0	55	7.2e-06	1		R.SVYEPLQTGLIAIDAMIPVGR.G
18440	142 – 162	748.4098	2242.2075	2242.2031	1.93	0	54	9.6e-06	1		R.SVYEPLQTGLIAIDAMIPVGR.G
18564	142 – 162	1130.1084	2258.2022	2258.1980	1.83	0	63	1.3e-06	1		R.SVYEPLQTGLIAIDAMIPVGR.G + Oxidation (M)
18565	142 – 162	753.7418	2258.2034	2258.1980	2.39	0	50	2e-05	1		R.SVYEPLQTGLIAIDAMIPVGR.G + Oxidation (M)
1186	166 – 172	408.2353	814.4560	814.4548	1.39	0	17	0.059	1		R.ELIIGDR.Q
1187	166 – 172	408.2357	814.4569	814.4548	2.51	0	17	0.027	1		R.ELIIGDR.Q
20969	177 – 202	935.4922	2803.4549	2803.4538	0.38	0	99	5.2e-10	1		K.TAVATDTILNQQGQNVICVYVAIGQK.A
13421	203 – 216	768.3944	1534.7743	1534.7740	0.22	0	55	6.5e-06	1		K.ASSVAQVVTNFQER.G
13422	203 – 216	768.3953	1534.7760	1534.7740	1.31	0	66	6e-07	1		K.ASSVAQVVTNFQER.G

13423	203 - 216	512.5994	1534.7764	1534.7740	1.55	0	14	0.045	1		K.ASSVAQVVTNFQER.G
14054	254 - 266	534.9554	1601.8445	1601.8413	1.99	1	22	0.0092	1		R.ERHTLIIYDDLSK.Q
9953	256 - 266	439.9060	1316.6962	1316.6976	-1.09	0	29	0.002	1		R.HTLIIYDDLSK.Q
9955	256 - 266	659.3578	1316.7010	1316.6976	2.57	0	53	2.5e-05	1		R.HTLIIYDDLSK.Q
9956	256 - 266	439.9077	1316.7014	1316.6976	2.87	0	32	0.00092	1		R.HTLIIYDDLSK.Q
1920	273 - 279	430.7548	859.4951	859.4949	0.20	0	20	0.11	1		R.QMSLLLR.R
1921	273 - 279	430.7551	859.4957	859.4949	0.88	0	14	0.16	1		R.QMSLLLR.R
13599	285 - 297	518.5851	1552.7336	1552.7310	1.66	0	39	0.00021	1		R.EAYPGDVFYLHSR.L
13600	285 - 297	777.3746	1552.7346	1552.7310	2.30	0	41	0.00014	1		R.EAYPGDVFYLHSR.L
13601	285 - 297	518.5855	1552.7348	1552.7310	2.42	0	13	0.057	1		R.EAYPGDVFYLHSR.L
13602	285 - 297	518.5856	1552.7350	1552.7310	2.55	0	49	2.5e-05	1		R.EAYPGDVFYLHSR.L
694	367 - 374	387.2295	772.4445	772.4443	0.27	0	8	0.19	1	U	R.VGSAAQIK.A
2458	406 - 413	451.2461	900.4776	900.4777	-0.14	0	10	0.12	1	U	K.ATQNQLAR.G
2459	406 - 413	451.2461	900.4777	900.4777	-0.041	0	13	0.067	1	U	K.ATQNQLAR.G
2460	406 - 413	451.2461	900.4777	900.4777	0.041	0	15	0.035	1	U	K.ATQNQLAR.G
2461	406 - 413	451.2464	900.4782	900.4777	0.56	0	2	0.71	1	U	K.ATQNQLAR.G
2463	406 - 413	451.2464	900.4783	900.4777	0.70	0	21	0.01	1	U	K.ATQNQLAR.G
2464	406 - 413	451.2465	900.4784	900.4777	0.72	0	36	0.00041	1	U	K.ATQNQLAR.G
2773	456 - 462	460.7822	919.5498	919.5491	0.75	1	28	0.0052	1	U	R.KYLVELR.T
872	457 - 462	396.7347	791.4548	791.4541	0.92	0	35	0.0025	1	U	K.YLVELR.T
873	457 - 462	396.7349	791.4553	791.4541	1.51	0	24	0.03	1	U	K.YLVELR.T
14222	467 - 480	811.4251	1620.8356	1620.8359	-0.19	0	30	0.0037	1	U	K.TNKPEFQEIISSTK.T
14224	467 - 480	541.2863	1620.8370	1620.8359	0.67	0	37	0.00034	1	U	K.TNKPEFQEIISSTK.T
14225	467 - 480	541.2869	1620.8388	1620.8359	1.82	0	27	0.0028	1	U	K.TNKPEFQEIISSTK.T
14227	467 - 480	541.2878	1620.8416	1620.8359	3.51	0	5	0.37	1	U	K.TNKPEFQEIISSTK.T
8756	481 - 491	417.8876	1250.6410	1250.6394	1.28	0	12	0.07	1	U	K.TFTEEAEALLK.E
8759	481 - 491	626.3308	1250.6470	1250.6394	6.07	0	49	2.7e-05	1	U	K.TFTEEAEALLK.E
19285	481 - 500	789.3907	2365.1502	2365.1471	1.31	1	0	0.95	1	U	K.TFTEEAEALLKEAIQEQMER.F
6255	492 - 500	567.2665	1132.5185	1132.5182	0.22	0	20	0.043	1	U	K.EAIQEQMER.F
6256	492 - 500	567.2675	1132.5204	1132.5182	1.90	0	59	5.9e-06	1	U	K.EAIQEQMER.F

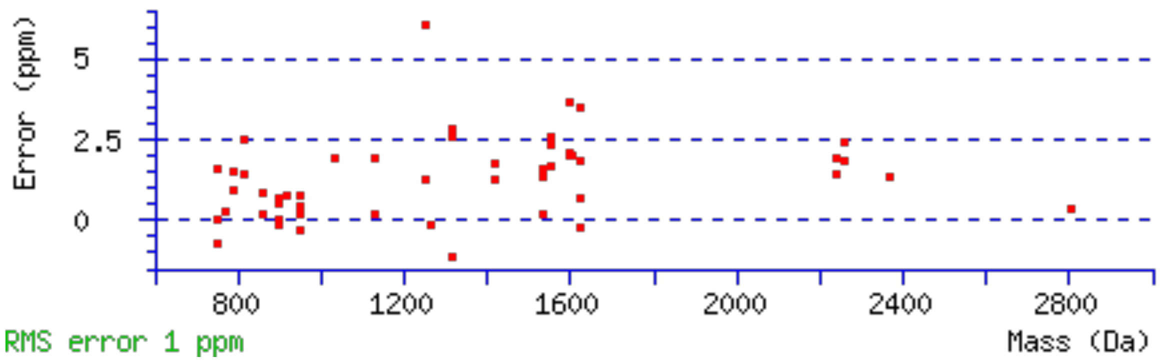

Mascot: http://www.matrixscience.com/

MASCOT Search Results

Protein View: tr|A0A0K9R1K7|A0A0K9R1K7_SPIOL

>tr|A0A0K9R1K7|A0A0K9R1K7_SPIOL Chlorophyll a-b binding protein, chloroplastic OS=Spinacia oleracea OX=3562 GN=SOVF_123890 PE=3 SV=1

**Database:**	Uni-Spinach
**Score:**	694
**Nominal mass (M_r):**	29569
**Calculated pI:**	7.85

Sequence similarity is available as **an NCBI BLAST search of tr|A0A0K9R1K7|A0A0K9R1K7_SPIOL against nr**.

Search parameters

**MS data file:**	\\128.97.66.218\tank\windowsVM\Bill Cramer\MGF\wc_QE_021417_Cramer_Gel_3S_4a.mgf
**Enzyme:**	Trypsin: cuts C-term side of KR unless next residue is P.
**Fixed modifications:**	Carbamidomethyl (C)
**Variable modifications:**	Oxidation (M)

Protein sequence coverage: 33%

Matched peptides shown in ***bold red***.

1 MATQALVSSS LTSSVETARQ ILGARSGLAP SSLRKNSFVV RAASTPPVK**Q**
51 **NANRPLWFAS** **K**QSLSYLDGS LPGDFGFDPL GLSDPEGTGG FIEPR**WLAYG**
101 **EIINGRYAML** **GAVGAIAPEI** **LGK**AGLIPQE TALPWFQTGV IPPAGTYNYW
151 ADPFTLFVFE MALMGFAEHR **RLQDWYNPGS** **MGKQYFLGLE** **KGFAGSGEPA**
201 **YPGGPIFNPL** **GFGKDEKSLK** **ELK**LKEVKNG RLAMLAILGY FIQGLVTGVG
251 PYQNLLDHLA DPVNNNILTS LKFH

Unformatted sequence string: **274 residues** (for pasting into other applications).

Sort peptides by ◉ Residue Number ○ Increasing Mass ○ Decreasing Mass

Show predicted peptides also

Query	Start – End	Observed	Mr(expt)	Mr(calc)	ppm	M	Score	Expect	Rank	U	Peptide
11829	50 – 61	716.3786	1430.7426	1430.7419	0.56	0	9	0.16	1	U	K.QNANRPLWFASK.Q
11830	50 – 61	716.3788	1430.7431	1430.7419	0.86	0	0	0.96	1	U	K.QNANRPLWFASK.Q
11831	50 – 61	716.3790	1430.7435	1430.7419	1.15	0	27	0.0031	1	U	K.QNANRPLWFASK.Q
11832	50 – 61	477.9219	1430.7440	1430.7419	1.51	0	9	0.13	1	U	K.QNANRPLWFASK.Q
11833	50 – 61	477.9225	1430.7457	1430.7419	2.67	0	8	0.19	1	U	K.QNANRPLWFASK.Q
11834	50 – 61	477.9233	1430.7480	1430.7419	4.31	0	18	0.022	1	U	K.QNANRPLWFASK.Q
9475	96 – 106	646.3438	1290.6731	1290.6720	0.79	0	59	2.7e-06	1	U	R.WLAYGEIINGR.Y
9476	96 – 106	431.2322	1290.6747	1290.6720	2.04	0	13	0.066	1	U	R.WLAYGEIINGR.Y
14712	107 – 123	837.4692	1672.9238	1672.9222	0.99	0	71	2e-07	1	U	R.YAMLGAVGAIAPEILGK.A
14713	107 – 123	558.6489	1672.9249	1672.9222	1.61	0	44	7.2e-05	1	U	R.YAMLGAVGAIAPEILGK.A
14863	107 – 123	845.4672	1688.9199	1688.9171	1.67	0	105	1.6e-10	1	U	R.YAMLGAVGAIAPEILGK.A + Oxidation (M)
14864	107 – 123	563.9813	1688.9220	1688.9171	2.91	0	52	1.3e-05	1	U	R.YAMLGAVGAIAPEILGK.A + Oxidation (M)
13574	171 – 183	517.9176	1550.7310	1550.7300	0.65	1	2	0.72	1	U	R.RLQDWYNPGSMGK.Q
13575	171 – 183	776.3729	1550.7313	1550.7300	0.86	1	57	4.5e-06	1	U	R.RLQDWYNPGSMGK.Q
13576	171 – 183	776.3730	1550.7314	1550.7300	0.95	1	18	0.022	1	U	R.RLQDWYNPGSMGK.Q
13577	171 – 183	517.9181	1550.7324	1550.7300	1.55	1	37	0.00034	1	U	R.RLQDWYNPGSMGK.Q
13578	171 – 183	517.9182	1550.7328	1550.7300	1.80	1	12	0.078	1	U	R.RLQDWYNPGSMGK.Q
13712	171 – 183	784.3709	1566.7273	1566.7249	1.51	1	59	3.2e-06	1	U	R.RLQDWYNPGSMGK.Q + Oxidation (M)
13713	171 – 183	523.2498	1566.7277	1566.7249	1.79	1	12	0.08	1	U	R.RLQDWYNPGSMGK.Q + Oxidation (M)
13714	171 – 183	523.2502	1566.7289	1566.7249	2.57	1	33	0.00074	1	U	R.RLQDWYNPGSMGK.Q + Oxidation (M)
13717	171 – 183	784.3743	1566.7340	1566.7249	5.84	1	24	0.0057	1	U	R.RLQDWYNPGSMGK.Q + Oxidation (M)
11218	172 – 183	698.3219	1394.6293	1394.6289	0.30	0	61	3.2e-06	1	U	R.LQDWYNPGSMGK.Q
11219	172 – 183	698.3221	1394.6296	1394.6289	0.52	0	33	0.0023	1	U	R.LQDWYNPGSMGK.Q
11220	172 – 183	698.3228	1394.6310	1394.6289	1.56	0	19	0.059	1	U	R.LQDWYNPGSMGK.Q
11223	172 – 183	698.3233	1394.6321	1394.6289	2.29	0	11	0.41	1	U	R.LQDWYNPGSMGK.Q
11493	172 – 183	706.3198	1410.6250	1410.6238	0.83	0	56	1.1e-05	1	U	R.LQDWYNPGSMGK.Q + Oxidation (M)
11495	172 – 183	706.3202	1410.6258	1410.6238	1.39	0	33	0.0021	1	U	R.LQDWYNPGSMGK.Q + Oxidation

											(M)
11496	172 – 183	706.3203	1410.6260	1410.6238	1.56	0	7	0.8	1	U	R.LQDWYNPGSMGK.Q + Oxidation (M)
3795	184 – 191	499.2719	996.5291	996.5280	1.14	0	10	0.13	1	U	K.QYFLGLEK.G
18386	192 – 214	1119.0548	2236.0951	2236.0953	-0.12	0	66	7e-07	1	U	K.GFAGSGEPAYPGGPIFNPLGFGK.D
18387	192 – 214	746.3723	2236.0951	2236.0953	-0.10	0	67	4.7e-07	1	U	K.GFAGSGEPAYPGGPIFNPLGFGK.D
20233	192 – 217	870.4274	2608.2605	2608.2598	0.26	1	61	1.8e-06	1	U	K.GFAGSGEPAYPGGPIFNPLGFGKDEK.S
20234	192 – 217	870.4306	2608.2701	2608.2598	3.94	1	38	0.00027	1	U	K.GFAGSGEPAYPGGPIFNPLGFGKDEK.S
116	218 – 223	359.2290	716.4435	716.4432	0.36	1	2	1.9	5	U	K.SLKELK.L

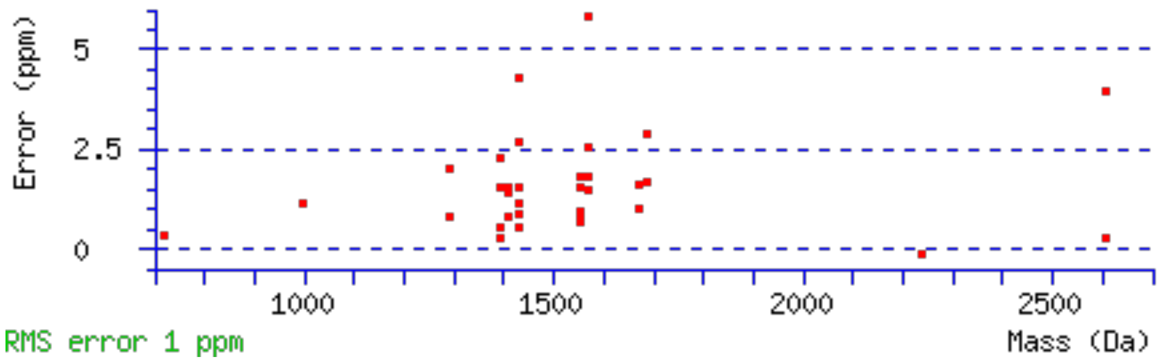

Mascot: http://www.matrixscience.com/

MASCOT Search Results

Protein View: tr|A0A0K9QGT7|A0A0K9QGT7_SPIOL

>tr|A0A0K9QGT7|A0A0K9QGT7_SPIOL Ferredoxin--NADP reductase, chloroplastic OS=Spinacia oleracea OX=3562 GN=SOVF_180280 PE=3 SV=1

**Database:**	Uni-Spinach
**Score:**	604
**Nominal mass (M_r):**	41399
**Calculated pI:**	8.67

Sequence similarity is available as an NCBI BLAST search of tr|A0A0K9QGT7|A0A0K9QGT7_SPIOL against nr.

Search parameters

**MS data file:**	\\128.97.66.218\tank\windowsVM\Bill Cramer\MGF\wc_QE_021417_Cramer_Gel_3S_4a.mgf
**Enzyme:**	Trypsin: cuts C-term side of KR unless next residue is P.
**Fixed modifications:**	Carbamidomethyl (C)
**Variable modifications:**	Oxidation (M)

Protein sequence coverage: 48%

Matched peptides shown in ***bold red***.

```
  1 MTTAVTAAVS  FPSTKTTSLS  ARSSSVISPD  KISYKKVPLY  YRNVSATGKM
 51 GPIRAQIASD  VEAPPPAPAK  VEKHSKKMEE  GITVNKFKPK  TPYVGRCLLN
101 TKITGDDAPG  ETWHMVFSHE  GEIPYREGQS  VGVIPDGEDK  NGKPHKLRLY
151 SIASSALGDF  GDAKSVSLCV  KRLIYTNDAG  ETIKGVCSNF  LCDLKPGAEV
201 KLTGPVGKEM  LMPKDPNATI  IMLGTGTGIA  PFRSFLWKMF  FEKHDDYKFN
251 GLAWLFLGVP  TSSSLLYKEE  FEKMKEKAPD  NFRLDFAVSR  EQTNEKGEKM
301 YIQTRMAQYA  VELWEMLKKD  NTYVYMCGLK  GMEKGIDDIM  VSLAAAEGID
351 WIEYKRQLKK  AEQWNVEVY
```

Unformatted sequence string: 369 residues (for pasting into other applications).

Sort peptides by ◉ Residue Number ○ Increasing Mass ○ Decreasing Mass

Show predicted peptides also

Query	Start – End	Observed	Mr(expt)	Mr(calc)	ppm	M	Score	Expect	Rank	U	Peptide
6564	77 – 86	574.8030	1147.5914	1147.5907	0.64	1	13	0.063	1	U	K.KMEEGITVNK.F
6565	77 – 86	383.5379	1147.5918	1147.5907	1.00	1	7	0.21	1	U	K.KMEEGITVNK.F
6566	77 – 86	574.8045	1147.5945	1147.5907	3.34	1	0	0.98	1	U	K.KMEEGITVNK.F
6874	77 – 86	388.8693	1163.5860	1163.5856	0.36	1	4	0.4	1	U	K.KMEEGITVNK.F + Oxidation (M)
6875	77 – 86	388.8694	1163.5864	1163.5856	0.66	1	10	0.1	1	U	K.KMEEGITVNK.F + Oxidation (M)
6876	77 – 86	582.8006	1163.5866	1163.5856	0.82	1	48	0.00018	1	U	K.KMEEGITVNK.F + Oxidation (M)
4126	78 – 86	510.7550	1019.4955	1019.4957	-0.25	0	6	0.29	1	U	K.MEEGITVNK.F
4393	78 – 86	518.7534	1035.4922	1035.4906	1.55	0	7	0.22	1	U	K.MEEGITVNK.F + Oxidation (M)
20800	103 – 126	686.8161	2743.2353	2743.2337	0.59	0	45	0.00014	1	U	K.ITGDDAPGETWHMVFSHEGEIPYR.E
11793	127 – 140	715.3440	1428.6735	1428.6733	0.15	0	56	5.9e-06	1	U	R.EGQSVGVIPDGEDK.N
11797	127 – 140	715.3477	1428.6808	1428.6733	5.31	0	8	0.18	1	U	R.EGQSVGVIPDGEDK.N
14131	149 – 164	538.9400	1613.7982	1613.7937	2.82	0	43	8.9e-05	1	U	R.LYSIASSALGDFGDAK.S
14132	149 – 164	807.9107	1613.8068	1613.7937	8.10	0	47	3.8e-05	1	U	R.LYSIASSALGDFGDAK.S
870	165 – 171	396.7185	791.4225	791.4211	1.74	0	3	0.49	1	U	K.SVSLCVK.R
12838	172 – 184	498.6040	1492.7901	1492.7885	1.03	1	13	0.061	1	U	K.RLIYTNDAGETIK.G
10249	173 – 184	669.3514	1336.6882	1336.6874	0.62	0	39	0.00022	1	U	R.LIYTNDAGETIK.G
16555	185 – 201	631.9790	1892.9151	1892.9125	1.39	0	15	0.038	1	U	K.GVCSNFLCDLKPGAEVK.L
16897	215 – 233	649.0133	1944.0181	1944.0139	2.19	0	73	1.5e-07	1	U	K.DPNATIIMLGTGTGIAPFR.S
16898	215 – 233	973.0165	1944.0184	1944.0139	2.30	0	93	1.8e-09	1	U	K.DPNATIIMLGTGTGIAPFR.S
17007	215 – 233	654.3438	1960.0096	1960.0088	0.38	0	64	9.6e-07	1	U	K.DPNATIIMLGTGTGIAPFR.S + Oxidation (M)
17008	215 – 233	981.0121	1960.0096	1960.0088	0.42	0	96	1e-09	1	U	K.DPNATIIMLGTGTGIAPFR.S + Oxidation (M)
22	239 – 243	351.1705	700.3264	700.3254	1.34	0	2	1.2	1	U	K.MFFEK.H
3529	276 – 283	488.7468	975.4791	975.4774	1.74	1	7	0.21	1	U	K.EKAPDNFR.L
134	278 – 283	360.1773	718.3401	718.3398	0.41	0	18	0.064	1	U	K.APDNFR.L

135	278 - 283	360.1777	718.3408	718.3398	1.29	0	3	0.5	1	U	K.APDNFR.L
13022	278 - 290	503.2611	1506.7615	1506.7579	2.34	1	18	0.021	1	U	K.APDNFRLDFAVSR.E
1071	284 - 290	404.2220	806.4294	806.4286	0.92	0	30	0.0078	1	U	R.LDFAVSR.E
1129	300 - 305	406.2102	810.4058	810.4058	0.0074	0	22	0.022	1	U	K.MYIQTR.M
1130	300 - 305	406.2104	810.4062	810.4058	0.48	0	13	0.071	1	U	K.MYIQTR.M
14104	306 - 318	537.9364	1610.7874	1610.7836	2.33	0	12	0.082	1	U	R.MAQYAVELWEMLK.K
14105	306 - 318	806.4016	1610.7887	1610.7836	3.14	0	63	1.3e-06	1	U	R.MAQYAVELWEMLK.K
14277	306 - 318	814.3947	1626.7748	1626.7785	-2.28	0	43	9.9e-05	1	U	R.MAQYAVELWEMLK.K + Oxidation (M)
14278	306 - 318	543.2670	1626.7792	1626.7785	0.38	0	4	0.46	1	U	R.MAQYAVELWEMLK.K + Oxidation (M)
14280	306 - 318	814.3992	1626.7839	1626.7785	3.26	0	56	5.1e-06	1	U	R.MAQYAVELWEMLK.K + Oxidation (M)
14428	306 - 318	822.3971	1642.7796	1642.7735	3.71	0	20	0.015	1	U	R.MAQYAVELWEMLK.K + 2 Oxidation (M)
10699	320 - 330	682.3061	1362.5977	1362.5948	2.10	0	30	0.0023	1	U	K.DNTYVYMCGLK.G
10973	320 - 330	690.3043	1378.5940	1378.5897	3.14	0	19	0.024	1	U	K.DNTYVYMCGLK.G + Oxidation (M)
9042	360 - 369	633.3119	1264.6093	1264.6088	0.43	1	2	0.59	1	U	K.KAEQWNVEVY.-

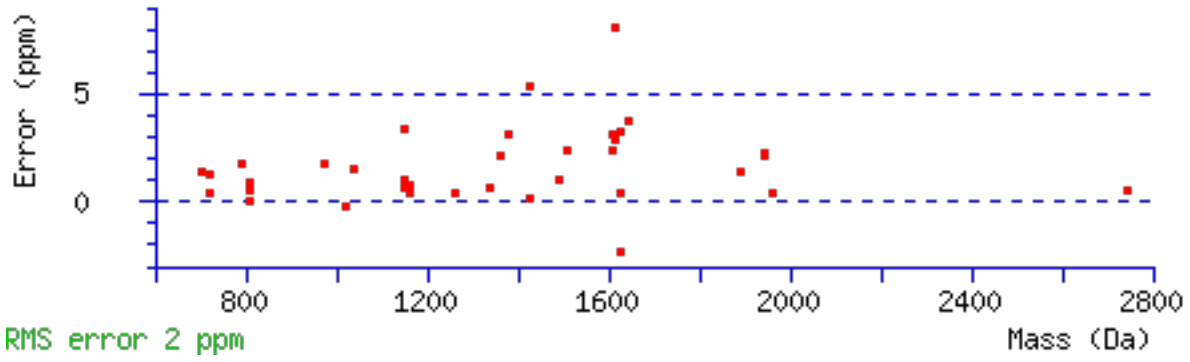

RMS error 2 ppm

Mascot: http://www.matrixscience.com/

MASCOT Search Results

Protein View: sp|P04160|PSBB_SPIOL

>sp|P04160|PSBB_SPIOL Photosystem II CP47 reaction center protein OS=Spinacia oleracea OX=3562 GN=psbB PE=1 SV=2

**Database:**	Uni-Spinach
**Score:**	517
**Nominal mass (M_r):**	56231
**Calculated pI:**	6.16

Sequence similarity is available as **an NCBI BLAST search of sp|P04160|PSBB_SPIOL against nr**.

Search parameters

**MS data file:**	\\128.97.66.218\tank\windowsVM\Bill Cramer\MGF\wc_QE_021417_Cramer_Gel_3S_4a.mgf
**Enzyme:**	Trypsin: cuts C-term side of KR unless next residue is P.
**Fixed modifications:**	**Carbamidomethyl (C)**
**Variable modifications:**	**Oxidation (M)**

Protein sequence coverage: 36%

Matched peptides shown in ***bold red***.

1 M**GLPWYRVHT** **VVLNDPGR**LI SVHIMHTALV AGWAGSMALY ELAVFDPSDP
51 VLDPMWR**QGM** **FVIPFMTR**LG ITNSWGGWSI TGGTITDPGI WSYEGVAGAH
101 IMFSGLCFLA AIWHWVYWDL EIFSDERTGK PSLDLPKIFG IHLFLSGVAC
151 FGFGAFHVTG LYGPGIWVSD PYGLTGKVQP VSPAWGVEGF DPFVPGGIAS
201 HHIAAGTLGI LAGLFHLSVR PPQRLYKGLR MGNIETVLSS SIAAVFFAAF
251 VVAGTMWYGS ATTPIELFGP TR**YQWDQGYF** **QQEIYRRVSA** **GLAENQSFSE**
301 **AWSK**IPEK**LA** **FYDYIGNNPA** KGGLFR**AGSM** **DNGDGIAVGW** **LGHPIFR**DKE
351 GRELFVR**RMP** **TFFETFPVVL** **IDGDGIVRAD** **VPFR**RAESK**Y** **SVEQVGVTVE**
401 **FYGGELNGVS** **YSDPATVK**KY AR**RAQLGEIF** **ELDR**ATLKSD GVFRSSPRGW
451 FTFGHASFAL LFFFGHIWHG SRTLFR**DVFA** **GIDPDLDVQV** **EFGAFQK**IGD
501 PTTRRQGV

Unformatted sequence string: **508 residues** (for pasting into other applications).

Sort peptides by ◉ Residue Number ○ Increasing Mass ○ Decreasing Mass

Show predicted peptides also

Query	Start – End	Observed	Mr(expt)	Mr(calc)	ppm	M	Score	Expect	Rank	U	Peptide
850	2 – 7	396.2138	790.4131	790.4126	0.61	0	6	0.29	1	U	M.GLPWYR.V
7822	8 – 18	603.8342	1205.6538	1205.6517	1.73	0	32	0.00099	1	U	R.VHTVVLNDPGR.L
7823	8 – 18	402.8920	1205.6541	1205.6517	2.02	0	43	9.5e-05	1	U	R.VHTVVLNDPGR.L
10112	58 – 68	663.8403	1325.6661	1325.6624	2.76	0	32	0.0011	1	U	R.QGMFVIPFMTR.L
16733	273 – 286	962.4398	1922.8651	1922.8587	3.30	0	91	2.3e-09	1	U	R.YQWDQGYFQQEIYR.R
16734	273 – 286	641.9635	1922.8688	1922.8587	5.22	0	40	0.00032	1	U	R.YQWDQGYFQQEIYR.R
17054	287 – 304	656.3263	1965.9571	1965.9544	1.35	1	4	0.44	1	U	R.RVSAGLAENQSFSEAWSK.I
15875	288 – 304	604.2916	1809.8530	1809.8533	-0.15	0	35	0.00053	1	U	R.VSAGLAENQSFSEAWSK.I
15876	288 – 304	905.9356	1809.8566	1809.8533	1.84	0	87	6.7e-09	1	U	R.VSAGLAENQSFSEAWSK.I
15877	288 – 304	905.9367	1809.8588	1809.8533	3.03	0	2	0.63	1	U	R.VSAGLAENQSFSEAWSK.I
12717	309 – 321	743.3736	1484.7327	1484.7299	1.85	0	38	0.00026	1	U	K.LAFYDYIGNNPAK.G
18047	327 – 347	724.0243	2169.0509	2169.0426	3.86	0	43	9.8e-05	1	U	R.AGSMDNGDGIAVGWLGHPIFR.D
19493	358 – 378	803.7617	2408.2633	2408.2563	2.93	1	52	1.3e-05	1	U	R.RMPTFFETFPVVLIDGDGIVR.A
19562	358 – 378	809.0920	2424.2543	2424.2512	1.27	1	46	4.8e-05	1	U	R.RMPTFFETFPVVLIDGDGIVR.A + Oxidation (M)
59	379 – 384	352.6900	703.3655	703.3653	0.24	0	16	0.095	1	U	R.ADVPFR.R
60	379 – 384	352.6905	703.3664	703.3653	1.59	0	5	0.34	1	U	R.ADVPFR.R
21451	390 – 418	1032.1701	3093.4885	3093.4819	2.15	0	27	0.0029	1	U	K.YSVEQVGVTVEFYGGELNGVSYSDPATVK.K
12087	423 – 434	723.8879	1445.7612	1445.7626	-0.97	1	5	0.47	1	U	R.RAQLGEIFELDR.A
12088	423 – 434	482.9285	1445.7637	1445.7626	0.76	1	20	0.013	1	U	R.RAQLGEIFELDR.A
9460	424 – 434	645.8398	1289.6650	1289.6615	2.67	0	51	1.7e-05	1	U	R.AQLGEIFELDR.A
18953	477 – 497	1155.5721	2309.1296	2309.1216	3.46	0	48	2.9e-05	1	U	R.DVFAGIDPDLDVQVEFGAFQK.I
18954	477 – 497	770.7177	2309.1312	2309.1216	4.13	0	65	8.9e-07	1	U	R.DVFAGIDPDLDVQVEFGAFQK.I

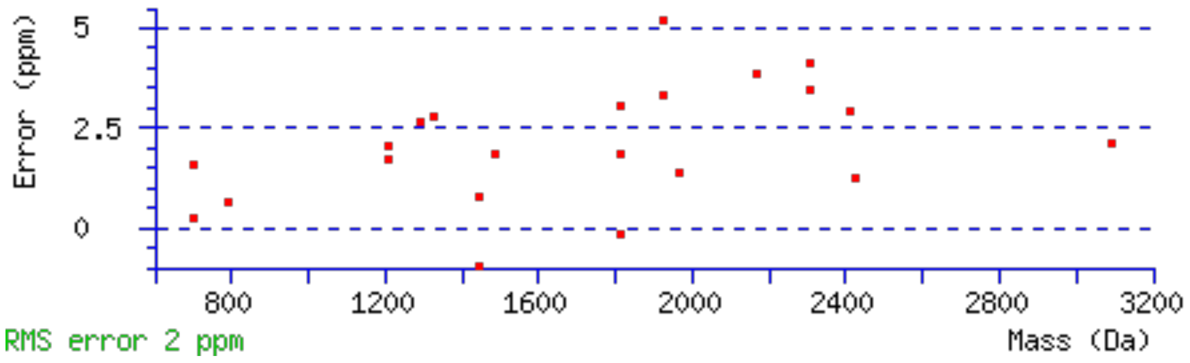

Mascot: http://www.matrixscience.com/

MASCOT Search Results

Protein View: tr|A0A0K9RVH9|A0A0K9RVH9_SPIOL

>tr|A0A0K9RVH9|A0A0K9RVH9_SPIOL Uncharacterized protein OS=Spinacia oleracea OX=3562 GN=SOVF_023270 PE=4 SV=1

**Database:**	Uni-Spinach
**Score:**	492
**Nominal mass (M_r):**	25568
**Calculated pI:**	9.40

Sequence similarity is available as **an NCBI BLAST search of tr|A0A0K9RVH9|A0A0K9RVH9_SPIOL against nr**.

Search parameters

**MS data file:**	\\128.97.66.218\tank\windowsVM\Bill Cramer\MGF\wc_QE_021417_Cramer_Gel_3S_4a.mgf
**Enzyme:**	Trypsin: cuts C-term side of KR unless next residue is P.
**Fixed modifications:**	**Carbamidomethyl (C)**
**Variable modifications:**	**Oxidation (M)**

Protein sequence coverage: 42%

Matched peptides shown in ***bold red***.

```
  1 MSFTIPTNLY KPLATKPKHL SSSSFAPRSK IVCQQENDQQ QPKKLELAKV
 51 GANAAAALAL SSVLLSSWSV APDAAMADIA GLTPCKESKQ FAKREKQALK
101 KLQASLKLYA DDSAPALAIK ATMEKTKKRF DNYGKYGLLC GSDGLPHLIV
151 SGDQRHWGEF ITPGILFLYI AGWIGWVGRS YLIAIRDEKK PTQKEIIIDV
201 PLASSLLFRG FSWPVAAYRE LLNGELVDNN F
```

Unformatted sequence string: **231 residues** (for pasting into other applications).

Sort peptides by ◉ Residue Number ○ Increasing Mass ○ Decreasing Mass

Show predicted peptides also

Query	Start – End	Observed	Mr(expt)	Mr(calc)	ppm	M	Score	Expect	Rank	U	Peptide
814	101 – 107	394.2557	786.4968	786.4963	0.60	1	8	0.49	1	U	K.KLQASLK.L
815	101 – 107	394.2557	786.4969	786.4963	0.77	1	16	0.097	1	U	K.KLQASLK.L
17773	101 – 120	529.8064	2115.1965	2115.1939	1.23	2	10	0.22	1	U	K.KLQASLKLYADDSAPALAIK.A
17189	102 – 120	663.3758	1987.1054	1987.0989	3.28	1	2	2.5	1	U	K.LQASLKLYADDSAPALAIK.A
10403	108 – 120	449.9104	1346.7095	1346.7081	1.01	0	25	0.011	1	U	K.LYADDSAPALAIK.A
10404	108 – 120	449.9107	1346.7103	1346.7081	1.58	0	2	0.58	1	U	K.LYADDSAPALAIK.A
10405	108 – 120	674.3628	1346.7110	1346.7081	2.10	0	41	0.00066	1	U	K.LYADDSAPALAIK.A
10407	108 – 120	674.3637	1346.7128	1346.7081	3.46	0	44	0.00044	1	U	K.LYADDSAPALAIK.A
10408	108 – 120	674.3647	1346.7149	1346.7081	4.98	0	29	0.0096	1	U	K.LYADDSAPALAIK.A
2407	129 – 135	450.2226	898.4307	898.4297	1.14	1	6	1.1	1	U	K.RFDNYGK.Y
2410	129 – 135	450.2229	898.4312	898.4297	1.66	1	9	0.37	1	U	K.RFDNYGK.Y
17990	136 – 155	719.6971	2156.0694	2156.0685	0.46	0	45	5.6e-05	1	U	K.YGLLCGSDGLPHLIVSGDQR.H
17991	136 – 155	719.6973	2156.0701	2156.0685	0.76	0	40	0.00017	1	U	K.YGLLCGSDGLPHLIVSGDQR.H
17992	136 – 155	719.6975	2156.0706	2156.0685	1.01	0	44	7.9e-05	1	U	K.YGLLCGSDGLPHLIVSGDQR.H
17993	136 – 155	719.6982	2156.0729	2156.0685	2.06	0	54	8.6e-06	1	U	K.YGLLCGSDGLPHLIVSGDQR.H
17994	136 – 155	1079.0445	2156.0744	2156.0685	2.76	0	80	3.3e-08	1	U	K.YGLLCGSDGLPHLIVSGDQR.H
17995	136 – 155	719.6988	2156.0747	2156.0685	2.90	0	1	0.8	1	U	K.YGLLCGSDGLPHLIVSGDQR.H
17996	136 – 155	719.6989	2156.0750	2156.0685	3.04	0	37	0.00037	1	U	K.YGLLCGSDGLPHLIVSGDQR.H
1527	180 – 186	418.2556	834.4966	834.4963	0.38	0	14	0.098	1	U	R.SYLIAIR.D
1530	180 – 186	418.2558	834.4971	834.4963	0.90	0	21	0.02	1	U	R.SYLIAIR.D
1533	180 – 186	418.2567	834.4987	834.4963	2.93	0	26	0.0055	1	U	R.SYLIAIR.D
7845	180 – 189	604.3378	1206.6611	1206.6608	0.21	1	24	0.0051	1	U	R.SYLIAIRDEK.K
7847	180 – 189	604.3388	1206.6630	1206.6608	1.83	1	27	0.0028	1	U	R.SYLIAIRDEK.K
7848	180 – 189	403.2285	1206.6636	1206.6608	2.28	1	13	0.055	1	U	R.SYLIAIRDEK.K
7850	180 – 189	604.3407	1206.6668	1206.6608	5.00	1	3	0.57	1	U	R.SYLIAIRDEK.K
18634	190 – 209	567.8398	2267.3301	2267.3253	2.12	1	28	0.0016	1	U	K.KPTQKEIIIDVPLASSLLFR.G
14829	195 – 209	562.6674	1684.9803	1684.9763	2.33	0	81	1.7e-08	1	U	K.EIIIDVPLASSLLFR.G
14830	195 – 209	843.4983	1684.9820	1684.9763	3.36	0	63	1.2e-06	1	U	K.EIIIDVPLASSLLFR.G
6689	210 – 219	385.1979	1152.5718	1152.5716	0.15	0	17	0.025	1	U	R.GFSWPVAAYR.E
6691	210 – 219	577.2938	1152.5730	1152.5716	1.18	0	47	3.8e-05	1	U	R.GFSWPVAAYR.E

6693	210 - 219	577.2946	1152.5747	1152.5716	2.66	0	27	0.0032	1	U	R.GFSWPVAAYR.E
10916	220 - 231	688.8406	1375.6667	1375.6619	3.49	0	4	0.39	1	U	R.ELLNGELVDNNF.-

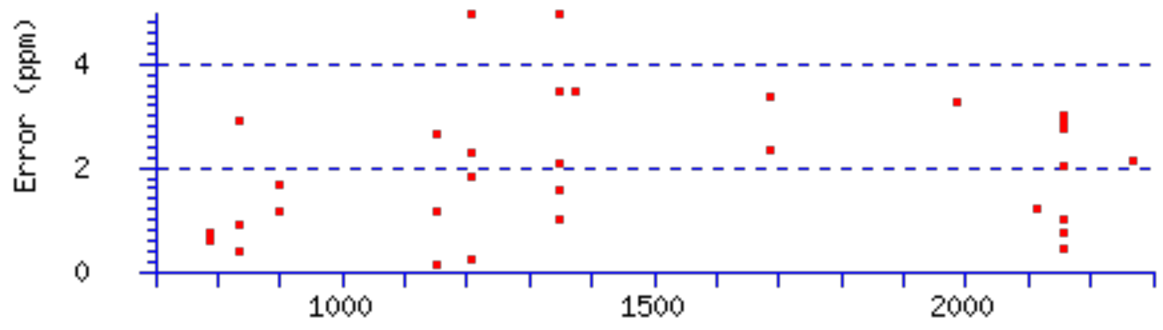

RMS error 2 ppm

Mascot: http://www.matrixscience.com/

MASCOT Search Results

Protein View: sp|P06511|PSAA_SPIOL

>sp|P06511|PSAA_SPIOL Photosystem I P700 chlorophyll a apoprotein A1 OS=Spinacia oleracea OX=3562 GN=psaA PE=3 SV=1

**Database:**	Uni-Spinach
**Score:**	471
**Nominal mass (M_r):**	83161
**Calculated pI:**	6.74

Sequence similarity is available as an NCBI BLAST search of sp|P06511|PSAA_SPIOL against nr.

Search parameters

**MS data file:**	\\128.97.66.218\tank\windowsVM\Bill Cramer\MGF\wc_QE_021417_Cramer_Gel_3S_4a.mgf
**Enzyme:**	Trypsin: cuts C-term side of KR unless next residue is P.
**Fixed modifications:**	Carbamidomethyl (C)
**Variable modifications:**	Oxidation (M)

Protein sequence coverage: 19%

Matched peptides shown in ***bold red***.

1 MIIR**SPEPEV** **KILVDRDPVK** **TSFEAWAKPG** **HFSR**TIAKGP ETTTWIWNLH
51 ADAHDFDSHT SDLEEISRKI FSAHFGQLSI IFLWLSGMYF HGARFSNYEA
101 WLSDPTHIGP SAQVVWPIVG QEILNGDVGG GFR**GIQITSG** **FFQIWR**ASGI
151 TSELQLYCTA IGALVFAALM LFAGWFHYHK AAPKLAWFQD VESMLNHHLA
201 GLLGLGSLSW AGHQIHVSLP INQFLNAGVD PKEIPLPHEL ILNRDLLAQL
251 YPSFAEGATP FFTLNWSK**YA** **DFLTFR**GGLD PVTGGLWLTD TAHHHLAIAI
301 LFLIAGHMYR **TNWGIGHGLK** **DILEAHKGPF** **TGQGHK**GLYE ILTTSWHAQL
351 ALNLAMLGSL TIVVAHHMYA MPPYPYLATD YGTQLSLFTH HMWIGGFLIV
401 GAAAHAAIFM VR**DYDPTTRY** **NDLLDRVLR**H RDAIISHLNW ACIFLGFHSF
451 GLYIHNDTMS ALGRPQDMFS DTAIQLQPVF AQWIQNTHAL APSATAPGAT
501 ASTSLTWGGS DLVAVGGKVA LLPIPLGTAD FLVHHIHAFT IHVTVLILLK
551 GVLFARSSR**L** **IPDKANLGFR** **FPCDGPGR**GG TCQVSAWDHV FLGLFWMYNS
601 ISVVIFHFSW KMQSDVWGSI SDQGVVTHIT GGNFAQSSIT INGWLRDFLW
651 AQASQVIQSY GSSLSAYGLF FLGAHFVWAF SLMFLFSGR**G** **YWQELIESIV**
701 **WAHNK**LKVAP **ATQPRALSIV** **QGR**AVGVTHY LLGGIATTWA FFLARIIAVG

Unformatted sequence string: 750 residues (for pasting into other applications).

Sort peptides by ⦿ Residue Number ○ Increasing Mass ○ Decreasing Mass

Show predicted peptides also

Query	Start – End	Observed	Mr(expt)	Mr(calc)	ppm	M	Score	Expect	Rank	U	Peptide
783	5 – 11	393.2054	784.3962	784.3967	-0.64	0	3	1.3	1	U	R.SPEPEVK.I
784	5 – 11	393.2054	784.3963	784.3967	-0.44	0	4	0.9	1	U	R.SPEPEVK.I
785	5 – 11	393.2055	784.3965	784.3967	-0.19	0	6	0.6	1	U	R.SPEPEVK.I
786	5 – 11	393.2056	784.3966	784.3967	-0.070	0	18	0.038	1	U	R.SPEPEVK.I
787	5 – 11	393.2056	784.3967	784.3967	-0.0064	0	7	0.46	1	U	R.SPEPEVK.I
788	5 – 11	393.2057	784.3968	784.3967	0.11	0	5	0.69	1	U	R.SPEPEVK.I
789	5 – 11	393.2057	784.3969	784.3967	0.25	0	6	0.54	1	U	R.SPEPEVK.I
790	5 – 11	393.2057	784.3969	784.3967	0.29	0	7	0.45	1	U	R.SPEPEVK.I
791	5 – 11	393.2058	784.3970	784.3967	0.40	0	9	0.28	1	U	R.SPEPEVK.I
792	5 – 11	393.2058	784.3971	784.3967	0.52	0	6	0.66	1	U	R.SPEPEVK.I
793	5 – 11	393.2058	784.3971	784.3967	0.53	0	5	0.82	1	U	R.SPEPEVK.I
794	5 – 11	393.2058	784.3971	784.3967	0.54	0	7	0.52	1	U	R.SPEPEVK.I
795	5 – 11	393.2058	784.3971	784.3967	0.55	0	6	0.65	1	U	R.SPEPEVK.I
796	5 – 11	393.2058	784.3971	784.3967	0.58	0	4	0.93	1	U	R.SPEPEVK.I
797	5 – 11	393.2059	784.3972	784.3967	0.65	0	4	1	1	U	R.SPEPEVK.I
798	5 – 11	393.2059	784.3973	784.3967	0.80	0	6	0.58	1	U	R.SPEPEVK.I
799	5 – 11	393.2060	784.3974	784.3967	0.93	0	5	0.69	1	U	R.SPEPEVK.I
4731	12 – 20	527.8162	1053.6179	1053.6182	-0.37	1	12	0.14	1	U	K.ILVDRDPVK.T
4732	12 – 20	527.8165	1053.6184	1053.6182	0.17	1	33	0.0012	1	U	K.ILVDRDPVK.T
20398	12 – 34	664.8554	2655.3924	2655.3922	0.100	2	42	0.00011	1	U	K.ILVDRDPVKTSFEAWAKPGHFSR.T
20399	12 – 34	443.5727	2655.3926	2655.3922	0.16	2	16	0.031	1	U	K.ILVDRDPVKTSFEAWAKPGHFSR.T

17529	17 – 34	687.3509	2059.0309	2059.0276	1.61	1	8	0.17	1	U	R.DPVKTSFEAWAKPGHFSR.T
14193	21 – 34	810.9003	1619.7861	1619.7845	0.98	0	12	0.074	1	U	K.TSFEAWAKPGHFSR.T
14194	21 – 34	540.9361	1619.7865	1619.7845	1.24	0	33	0.00089	1	U	K.TSFEAWAKPGHFSR.T
14195	21 – 34	405.9539	1619.7866	1619.7845	1.32	0	32	0.001	1	U	K.TSFEAWAKPGHFSR.T
14197	21 – 34	405.9540	1619.7870	1619.7845	1.56	0	20	0.012	1	U	K.TSFEAWAKPGHFSR.T
14198	21 – 34	540.9365	1619.7876	1619.7845	1.92	0	32	0.00095	1	U	K.TSFEAWAKPGHFSR.T
14201	21 – 34	405.9544	1619.7885	1619.7845	2.51	0	20	0.012	1	U	K.TSFEAWAKPGHFSR.T
13587	134 – 146	776.9188	1551.8230	1551.8198	2.06	0	85	1.1e-08	1	U	R.GIQITSGFFQIWR.A
13588	134 – 146	518.2820	1551.8242	1551.8198	2.80	0	64	1e-06	1	U	R.GIQITSGFFQIWR.A
4345	269 – 276	516.7622	1031.5098	1031.5076	2.11	0	45	6.7e-05	1	U	K.YADFLTFR.G
4347	269 – 276	516.7629	1031.5113	1031.5076	3.59	0	4	0.41	1	U	K.YADFLTFR.G
4348	269 – 276	516.7633	1031.5120	1031.5076	4.28	0	17	0.025	1	U	K.YADFLTFR.G
5255	311 – 320	541.7908	1081.5671	1081.5669	0.19	0	31	0.0012	1	U	R.TNWGIGHGLK.D
5257	311 – 320	541.7914	1081.5683	1081.5669	1.33	0	29	0.0018	1	U	R.TNWGIGHGLK.D
16509	311 – 327	630.3388	1887.9944	1887.9955	-0.58	1	1	0.84	1	U	R.TNWGIGHGLKDILEAHK.G
16510	311 – 327	473.0062	1887.9959	1887.9955	0.19	1	1	0.73	1	U	R.TNWGIGHGLKDILEAHK.G
16512	311 – 327	630.3399	1887.9979	1887.9955	1.25	1	12	0.07	1	U	R.TNWGIGHGLKDILEAHK.G
16514	311 – 327	473.0070	1887.9989	1887.9955	1.79	1	7	0.23	1	U	R.TNWGIGHGLKDILEAHK.G
1336	321 – 327	413.2271	824.4397	824.4392	0.67	0	6	1.3	1	U	K.DILEAHK.G
15255	321 – 336	434.4786	1733.8854	1733.8849	0.28	1	34	0.00075	1	U	K.DILEAHKGPFTGQGHK.G
15256	321 – 336	578.9698	1733.8876	1733.8849	1.56	1	30	0.0018	1	U	K.DILEAHKGPFTGQGHK.G
15257	321 – 336	578.9699	1733.8879	1733.8849	1.72	1	12	0.079	1	U	K.DILEAHKGPFTGQGHK.G
15258	321 – 336	578.9702	1733.8886	1733.8849	2.15	1	29	0.002	1	U	K.DILEAHKGPFTGQGHK.G
15259	321 – 336	434.4796	1733.8893	1733.8849	2.51	1	35	0.00053	1	U	K.DILEAHKGPFTGQGHK.G
15260	321 – 336	434.4797	1733.8897	1733.8849	2.76	1	7	0.24	1	U	K.DILEAHKGPFTGQGHK.G
2835	328 – 336	464.7359	927.4572	927.4563	1.00	0	7	0.78	1	U	K.GPFTGQGHK.G
1981	413 – 419	434.1962	866.3778	866.3770	0.87	0	13	0.12	1	U	R.DYDPTTR.Y
1982	413 – 419	434.1962	866.3779	866.3770	0.97	0	8	0.28	1	U	R.DYDPTTR.Y
1983	413 – 419	434.1964	866.3782	866.3770	1.33	0	13	0.11	1	U	R.DYDPTTR.Y
15457	413 – 426	586.2782	1755.8127	1755.8064	3.62	1	4	0.39	1	U	R.DYDPTTRYNDLLDR.V
2597	420 – 426	454.7276	907.4406	907.4399	0.79	0	21	0.029	1	U	R.YNDLLDR.V
2598	420 – 426	454.7280	907.4415	907.4399	1.69	0	4	0.46	1	U	R.YNDLLDR.V
9266	420 – 429	426.2401	1275.6983	1275.6935	3.78	1	16	0.029	1	U	R.YNDLLDRVLR.H
8615	560 – 570	415.2422	1242.7047	1242.7084	-3.02	1	19	0.016	1	U	R.LIPDKANLGFR.F
8616	560 – 570	415.2429	1242.7070	1242.7084	-1.15	1	3	0.53	1	U	R.LIPDKANLGFR.F
8617	560 – 570	622.3627	1242.7109	1242.7084	1.98	1	40	0.00016	1	U	R.LIPDKANLGFR.F
8618	560 – 570	415.2443	1242.7110	1242.7084	2.04	1	38	0.00029	1	U	R.LIPDKANLGFR.F
2529	571 – 578	453.2002	904.3859	904.3862	-0.23	0	15	0.074	1	U	R.FPCDGPGR.G
2530	571 – 578	453.2003	904.3861	904.3862	-0.077	0	7	0.39	1	U	R.FPCDGPGR.G
2531	571 – 578	453.2003	904.3861	904.3862	-0.020	0	10	0.2	1	U	R.FPCDGPGR.G
2532	571 – 578	453.2004	904.3863	904.3862	0.11	0	17	0.046	1	U	R.FPCDGPGR.G
2533	571 – 578	453.2004	904.3863	904.3862	0.17	0	15	0.077	1	U	R.FPCDGPGR.G
2535	571 – 578	453.2009	904.3873	904.3862	1.32	0	18	0.029	1	U	R.FPCDGPGR.G
2536	571 – 578	453.2010	904.3874	904.3862	1.41	0	5	0.63	1	U	R.FPCDGPGR.G
2537	571 – 578	453.2010	904.3875	904.3862	1.53	0	9	0.26	1	U	R.FPCDGPGR.G
2538	571 – 578	453.2012	904.3877	904.3862	1.76	0	20	0.018	1	U	R.FPCDGPGR.G
2539	571 – 578	453.2012	904.3878	904.3862	1.81	0	10	0.18	1	U	R.FPCDGPGR.G
17095	690 – 705	658.3370	1971.9893	1971.9843	2.55	0	36	0.00046	1	U	R.GYWQELIESIVWAHNK.L
1576	708 – 715	420.2441	838.4736	838.4661	9.01	0	3	1.2	2	U	K.VAPATQPR.A
1577	708 – 715	420.2444	838.4742	838.4661	9.72	0	2	1.1	2	U	K.VAPATQPR.A
1578	708 – 715	420.2444	838.4742	838.4661	9.72	0	3	1.2	2	U	K.VAPATQPR.A
1669	716 – 723	422.2567	842.4988	842.4974	1.67	0	7	0.23	1	U	R.ALSIVQGR.A
1670	716 – 723	422.2569	842.4993	842.4974	2.27	0	22	0.0095	1	U	R.ALSIVQGR.A

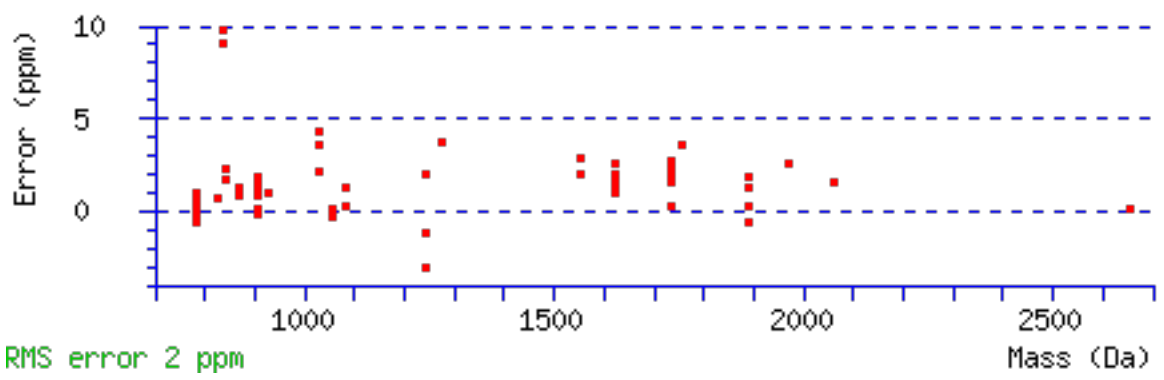

Mascot: http://www.matrixscience.com/

MASCOT Search Results

Protein View: sp|P10098|PSAC_SPIOL

>sp|P10098|PSAC_SPIOL Photosystem I iron-sulfur center OS=Spinacia oleracea OX=3562 GN=psaC PE=1 SV=3

**Database:**	Uni-Spinach
**Score:**	450
**Nominal mass (M_r):**	9531
**Calculated pI:**	6.67

Sequence similarity is available as an NCBI BLAST search of sp|P10098|PSAC_SPIOL against nr.

Search parameters

**MS data file:**	\\128.97.66.218\tank\windowsVM\Bill Cramer\MGF\wc_QE_021417_Cramer_Gel_3S_4a.mgf
**Enzyme:**	Trypsin: cuts C-term side of KR unless next residue is P.
**Fixed modifications:**	Carbamidomethyl (C)
**Variable modifications:**	Oxidation (M)

Protein sequence coverage: 80%

Matched peptides shown in ***bold red***.

```
 1 MSHSVKIYDT CIGCTQCVRA CPTDVLEMIP WDGCKAKQIA SAPRTEDCVG
51 CKRCESACPT DFLSVRVYLW HETTRSMGLG Y
```

Unformatted sequence string: 81 residues (for pasting into other applications).

Sort peptides by ◉ Residue Number ○ Increasing Mass ○ Decreasing Mass

Show predicted peptides also

Query	Start – End	Observed	Mr(expt)	Mr(calc)	ppm	M	Score	Expect	Rank	U	Peptide
14442	7 – 19	823.3574	1644.7002	1644.7059	-3.45	0	33	0.00072	1	U	K.IYDTCIGCTQCVR.A
14443	7 – 19	823.3601	1644.7056	1644.7059	-0.15	0	69	2.2e-07	1	U	K.IYDTCIGCTQCVR.A
14445	7 – 19	549.2455	1644.7147	1644.7059	5.36	0	28	0.003	1	U	K.IYDTCIGCTQCVR.A
16534	20 – 35	946.4237	1890.8328	1890.8314	0.72	0	78	4.5e-08	1	U	R.ACPTDVLEMIPWDGCK.A
16535	20 – 35	631.2851	1890.8333	1890.8314	1.00	0	54	1.1e-05	1	U	R.ACPTDVLEMIPWDGCK.A
16644	20 – 35	954.4209	1906.8272	1906.8264	0.46	0	58	3.9e-06	1	U	R.ACPTDVLEMIPWDGCK.A + Oxidation (M)
16645	20 – 35	636.6181	1906.8324	1906.8264	3.16	0	23	0.013	1	U	R.ACPTDVLEMIPWDGCK.A + Oxidation (M)
16646	20 – 35	954.4239	1906.8333	1906.8264	3.62	0	31	0.0025	1	U	R.ACPTDVLEMIPWDGCK.A + Oxidation (M)
358	38 – 44	371.7140	741.4135	741.4133	0.22	0	1	2.7	1	U	K.QIASAPR.T
359	38 – 44	371.7141	741.4137	741.4133	0.48	0	16	0.081	1	U	K.QIASAPR.T
360	38 – 44	371.7141	741.4137	741.4133	0.50	0	14	0.12	1	U	K.QIASAPR.T
361	38 – 44	371.7142	741.4139	741.4133	0.74	0	6	0.79	1	U	K.QIASAPR.T
14916	53 – 66	849.3875	1696.7604	1696.7661	-3.35	1	21	0.025	1	U	K.RCESACPTDFLSVR.V
14917	53 – 66	566.5973	1696.7700	1696.7661	2.28	1	44	0.00013	1	U	K.RCESACPTDFLSVR.V
13500	54 – 66	514.5622	1540.6647	1540.6650	-0.22	0	23	0.014	1	U	R.CESACPTDFLSVR.V
13501	54 – 66	771.3405	1540.6665	1540.6650	0.93	0	41	0.00022	1	U	R.CESACPTDFLSVR.V
13502	54 – 66	771.3409	1540.6673	1540.6650	1.50	0	68	4.4e-07	1	U	R.CESACPTDFLSVR.V
13503	54 – 66	771.3417	1540.6688	1540.6650	2.48	0	62	2.1e-06	1	U	R.CESACPTDFLSVR.V
7771	67 – 75	402.2085	1203.6038	1203.6037	0.091	0	13	0.065	1	U	R.VYLWHETTR.S
7772	67 – 75	602.8093	1203.6040	1203.6037	0.25	0	29	0.0021	1	U	R.VYLWHETTR.S
7773	67 – 75	602.8094	1203.6042	1203.6037	0.48	0	28	0.0026	1	U	R.VYLWHETTR.S
7774	67 – 75	402.2090	1203.6051	1203.6037	1.22	0	15	0.037	1	U	R.VYLWHETTR.S
7775	67 – 75	402.2096	1203.6069	1203.6037	2.67	0	2	0.59	1	U	R.VYLWHETTR.S
7777	67 – 75	402.2097	1203.6074	1203.6037	3.10	0	15	0.039	1	U	R.VYLWHETTR.S
16011	67 – 81	610.2970	1827.8690	1827.8614	4.18	1	2	0.64	1	U	R.VYLWHETTRSMGLGY.- + Oxidation (M)

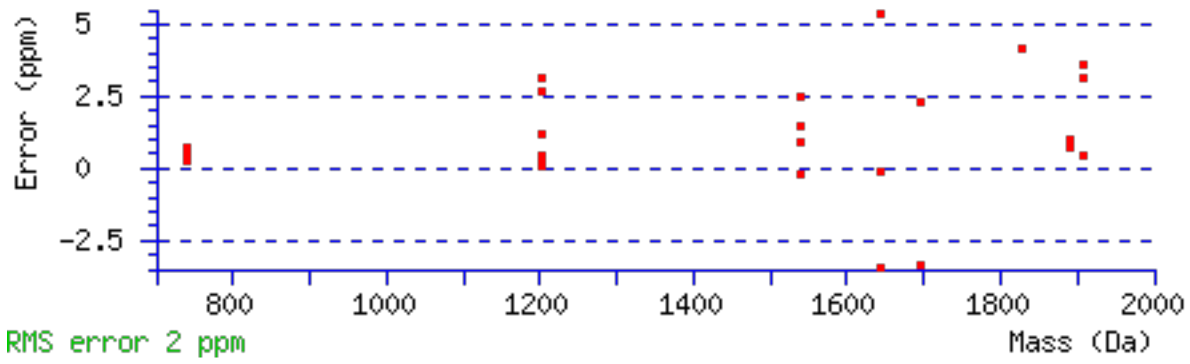

**Mascot: http://www.matrixscience.com/**

MASCOT Search Results

Protein View: tr|A0A0K9RXF7|A0A0K9RXF7_SPIOL

>tr|A0A0K9RXF7|A0A0K9RXF7_SPIOL Uncharacterized protein OS=Spinacia oleracea OX=3562 GN=SOVF_017720 PE=3 SV=1

**Database:**	Uni-Spinach
**Score:**	449
**Nominal mass (M_r):**	27664
**Calculated pI:**	5.80

Sequence similarity is available as **an NCBI BLAST search of tr|A0A0K9RXF7|A0A0K9RXF7_SPIOL against nr**.

Search parameters

**MS data file:**	\\128.97.66.218\tank\windowsVM\Bill Cramer\MGF\wc_QE_021417_Cramer_Gel_3S_4a.mgf
**Enzyme:**	Trypsin: cuts C-term side of KR unless next residue is P.
**Fixed modifications:**	Carbamidomethyl (C)
**Variable modifications:**	Oxidation (M)

Protein sequence coverage: 58%

Matched peptides shown in ***bold red***.

```
  1 MAALQNPVAL QSRTTTAVAA LSTSSTTSTP KPFSLSFSSS TATFNPLRLK
 51 ILTASKLTAK PRGGALGTRM VDSTASRYAS ALADVADVTG TLEATNSDVE
101 KLIRIFSEEP VYYFFANPVI SIDNKRSVLD EIITTSGLQP HTANFINILI
151 DSERINLVKE ILNEFEDVFN KITGTEVAVV TSVVKLENDH LAQIAKGVQK
201 ITGAKNVRIK TVIDPSLVAG FTIRYGNEGS KLVDMSVKKQ LEEIAAQLEM
251 DDVTLAV
```

Unformatted sequence string: **257 residues** (for pasting into other applications).

Sort peptides by ◉ Residue Number ○ Increasing Mass ○ Decreasing Mass

Show predicted peptides also

Query	Start – End	Observed	Mr(expt)	Mr(calc)	ppm	M	Score	Expect	Rank	U	Peptide
19601	78 – 101	814.0626	2439.1660	2439.1653	0.28	0	74	1.1e-07	1	U	R.YASALADVADVTGTLEATNSDVEK.L
19602	78 – 101	1220.5911	2439.1677	2439.1653	1.00	0	90	3.6e-09	1	U	R.YASALADVADVTGTLEATNSDVEK.L
19799	105 – 125	831.4211	2491.2416	2491.2311	4.22	0	79	3.7e-08	1	U	R.IFSEEPVYYFFANPVISIDNK.R
20372	105 – 126	883.4540	2647.3403	2647.3322	3.04	1	59	2.9e-06	1	U	R.IFSEEPVYYFFANPVISIDNKR.S
21456	127 – 154	774.9137	3095.6257	3095.6139	3.81	0	22	0.033	1	U	R.SVLDEIITTSGLQPHTANFINILIDSER.I
12869	160 – 171	748.8679	1495.7212	1495.7195	1.14	0	60	2.6e-06	1	U	K.EILNEFEDVFNK.I
12870	160 – 171	499.5814	1495.7225	1495.7195	2.01	0	27	0.0028	1	U	K.EILNEFEDVFNK.I
11352	172 – 185	468.2764	1401.8073	1401.8079	-0.43	0	18	0.078	1	U	K.ITGTEVAVVTSVVK.L
11353	172 – 185	701.9116	1401.8087	1401.8079	0.58	0	66	1.1e-06	1	U	K.ITGTEVAVVTSVVK.L
8761	186 – 196	417.8953	1250.6640	1250.6619	1.68	0	27	0.0037	1	U	K.LENDHLAQIAK.G
12768	211 – 224	744.9255	1487.8364	1487.8348	1.10	0	64	9.2e-07	1	U	K.TVIDPSLVAGFTIR.Y
12769	211 – 224	496.9530	1487.8373	1487.8348	1.67	0	47	4.1e-05	1	U	K.TVIDPSLVAGFTIR.Y
855	232 – 238	396.2209	790.4272	790.4259	1.73	0	14	0.15	1	U	K.LVDMSVK.K
17278	240 – 257	668.6697	2002.9873	2002.9769	5.20	0	0	0.95	1	U	K.QLEEIAAQLEMDDVTLAV.- + Oxidation (M)

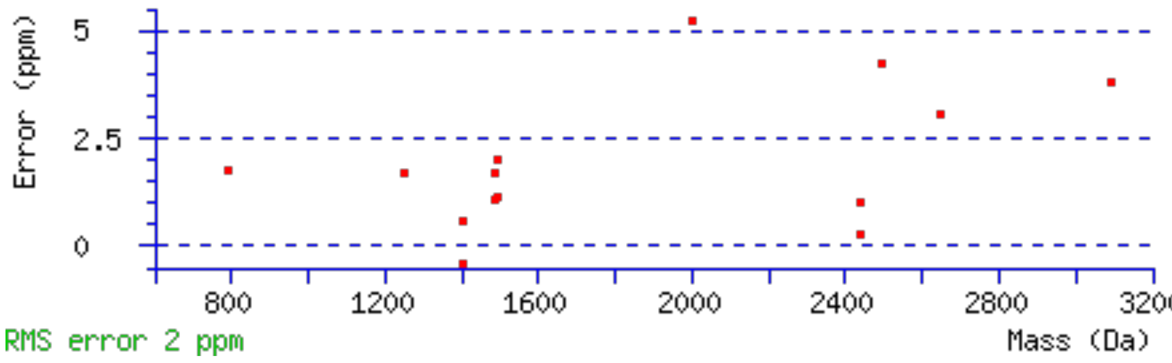

Mascot: http://www.matrixscience.com/

MASCOT Search Results

Protein View: tr|A0A0K9RD58|A0A0K9RD58_SPIOL

>tr|A0A0K9RD58|A0A0K9RD58_SPIOL AAA domain-containing protein OS=Spinacia oleracea OX=3562 GN=SOVF_080200 PE=3 SV=1

**Database:**	Uni-Spinach
**Score:**	439
**Nominal mass (M_r):**	73817
**Calculated pI:**	5.70

Sequence similarity is available as **an NCBI BLAST search of tr|A0A0K9RD58|A0A0K9RD58_SPIOL against nr**.

Search parameters

**MS data file:**	\\128.97.66.218\tank\windowsVM\Bill Cramer\MGF\wc_QE_021417_Cramer_Gel_3S_4a.mgf
**Enzyme:**	Trypsin: cuts C-term side of KR unless next residue is P.
**Fixed modifications:**	**Carbamidomethyl (C)**
**Variable modifications:**	**Oxidation (M)**

Protein sequence coverage: 30%

Matched peptides shown in ***bold red***.

```
  1 MAASSACLLG HGSSTSSSKT ILKERLFHGH LFAGRSVLLK SQKAFIVKAS
 51 SDKAQSDGRR GFLKLLLGNA GVASTLVASG NANADEQGVS SSRMSYSRFL
101 EYLDKDRVQK VDLFENGTIA IVEAISPELG NRVQRVRVQL PGLSQELLQK
151 LREKNIDFAA HNAQEDSGSV LFNLIGNLAF PLILIGGLFL LSRRSGGGMG
201 GPGGPGNPLA FGQSKAKFQM EPNTGVTFDD VAGVDEAKQD FMEVVEFLKK
251 PERFTAVGAR IPKGVLLVGP PGTGKTLLAK AIAGEAGVPF FSISGSEFVE
301 MFVGVGASRV RDLFKKAKEN APCIVFVDEI DAVGRSRGTG IGGGNDEREQ
351 TLNQLLTEMD GFEGNTGIIV VAATNRADIL DSALLRPGRF DRQVSVDVPD
401 VRGRTDILKV HASNKKFDGD VSLEVIAMRT PGFSGADLAN LLNEAAILAG
451 RRGKTAICSK EIDDSIDRIV AGMEGTVMTD SKSKSLVAYH EVGHAICGTL
501 TPGHDAVQKV TLVPRGQARG LTWFIPSDDP TLISKQQLFA RIVGGLGGRA
551 AEEIIFGESE VTTGAAGDLQ QITGLAKQMV TTFGMSDIGP WSLMDASAQS
601 GDVIMRMMAR NSMSERLAED IDNAVKKITD EAYQIALTHI RNNREAIDKI
651 VDVLLETETV SGDEFRTLLA EFTEIPVENR VPAAAASPVT V
```

Unformatted sequence string: **691 residues** (for pasting into other applications).

Sort peptides by ◉ Residue Number ○ Increasing Mass ○ Decreasing Mass

Show predicted peptides also

Query	Start – End	Observed	Mr(expt)	Mr(calc)	ppm	M	Score	Expect	Rank	U	Peptide
7628	99 – 107	599.8087	1197.6028	1197.6030	-0.13	1	20	0.015	1	U	R.FLEYLDKDR.V
7629	99 – 107	400.2091	1197.6054	1197.6030	2.07	1	27	0.0031	1	U	R.FLEYLDKDR.V
12197	138 – 150	726.9269	1451.8392	1451.8348	3.08	0	15	0.1	1	U	R.VQLPGLSQELLQK.L
16035	195 – 215	610.6262	1828.8568	1828.8527	2.26	0	8	0.17	1	U	R.SGGGMGGPGGPGNPLAFGQSK.A
16036	195 – 215	915.4370	1828.8594	1828.8527	3.71	0	33	0.00075	1	U	R.SGGGMGGPGGPGNPLAFGQSK.A
16191	195 – 215	923.4301	1844.8456	1844.8476	-1.05	0	47	4.3e-05	1	U	R.SGGGMGGPGGPGNPLAFGQSK.A + Oxidation (M)
156	254 – 260	361.2032	720.3918	720.3919	-0.039	0	16	0.03	1	U	R.FTAVGAR.I
5527	264 – 275	547.8326	1093.6506	1093.6496	0.94	0	26	0.0057	1	U	K.GVLLVGPPGTGK.T
5528	264 – 275	547.8329	1093.6512	1093.6496	1.51	0	23	0.012	1	U	K.GVLLVGPPGTGK.T
11257	377 – 389	698.9000	1395.7854	1395.7834	1.43	0	7	0.2	1	U	R.ADILDSALLRPGR.F
5910	393 – 402	557.2983	1112.5821	1112.5826	-0.50	0	38	0.0003	1	U	R.QVSVDVPDVR.G
13838	416 – 429	790.4117	1578.8089	1578.8076	0.82	1	48	4.5e-05	1	U	K.KFDGDVSLEVIAMR.T
13839	416 – 429	527.2773	1578.8101	1578.8076	1.62	1	55	3.3e-05	1	U	K.KFDGDVSLEVIAMR.T
13990	416 – 429	532.6091	1594.8055	1594.8025	1.88	1	2	0.67	1	U	K.KFDGDVSLEVIAMR.T + Oxidation (M)
12179	417 – 429	726.3633	1450.7121	1450.7126	-0.34	0	73	1.4e-07	1	U	K.FDGDVSLEVIAMR.T
18059	430 – 451	724.3880	2170.1422	2170.1382	1.87	0	17	0.031	1	U	R.TPGFSGADLANLLNEAAILAGR.R
11958	469 – 482	719.8515	1437.6885	1437.6844	2.88	0	31	0.0013	1	U	R.IVAGMEGTVMTDSK.S
15695	520 – 535	895.4734	1788.9323	1788.9298	1.38	0	30	0.0016	1	U	R.GLTWFIPSDDPTLISK.Q
562	536 – 541	381.7165	761.4184	761.4184	0.022	0	10	0.13	1	U	K.QQLFAR.I
21034	550 – 577	940.4862	2818.4368	2818.4236	4.67	0	100	3.9e-10	1	U	R.AAEEIIFGESEVTTGAAGDLQQITGLAK.Q
15576	627 – 641	591.3293	1770.9662	1770.9628	1.92	1	24	0.0058	1	U	K.KITDEAYQIALTHIR.N
14433	628 – 641	548.6304	1642.8693	1642.8678	0.89	0	38	0.00027	1	U	K.ITDEAYQIALTHIR.N
14307	667 – 680	544.6270	1630.8591	1630.8566	1.51	0	19	0.016	1	U	R.TLLAEFTEIPVENR.V

14308 667 – 680 816.4395 1630.8644 1630.8566 4.75 0 54 7.9e-06 1 U R.TLLAEFTEIPVENR.V

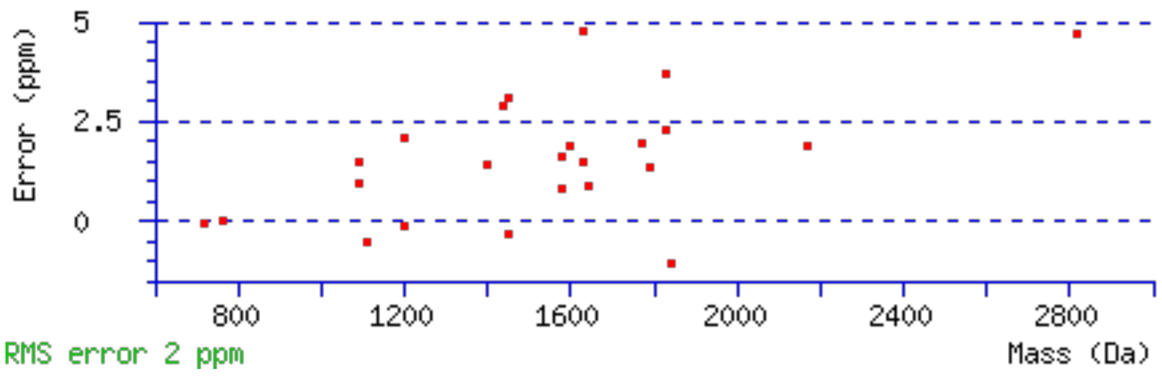

Mascot: http://www.matrixscience.com/

MASCOT Search Results

Protein View: tr|A0A0K9RHN1|A0A0K9RHN1_SPIOL

>tr|A0A0K9RHN1|A0A0K9RHN1_SPIOL Uncharacterized protein OS=Spinacia oleracea OX=3562 GN=SOVF_065780 PE=4 SV=1

**Database:**	Uni-Spinach
**Score:**	435
**Nominal mass (M_r):**	13359
**Calculated pI:**	9.99

Sequence similarity is available as an NCBI BLAST search of tr|A0A0K9RHN1|A0A0K9RHN1_SPIOL against nr.

Search parameters

**MS data file:**	\\128.97.66.218\tank\windowsVM\Bill Cramer\MGF\wc_QE_021417_Cramer_Gel_3S_4a.mgf
**Enzyme:**	Trypsin: cuts C-term side of KR unless next residue is P.
**Fixed modifications:**	Carbamidomethyl (C)
**Variable modifications:**	Oxidation (M)

Protein sequence coverage: 60%

Matched peptides shown in ***bold red***.

```
  1 MASIASSVAV RLGLTQVLPN KNFSSPRSTR LVVRAAEEAA AAPAAASPEG
 51 EAPKAAAKPP PIGPKRGSKV RIMRKESYWY KGVGSVVAVD QDPKTRYPVV
101 VRFNKVNYAN VSTNNYALDE IQEVA
```

Unformatted sequence string: 125 residues (for pasting into other applications).

Sort peptides by ● Residue Number ○ Increasing Mass ○ Decreasing Mass

Show predicted peptides also

Query	Start – End	Observed	Mr(expt)	Mr(calc)	ppm	M	Score	Expect	Rank	U	Peptide
15851	35 – 54	603.6269	1807.8588	1807.8587	0.0017	0	54	8.2e-06	1	U	R.AAEEAAAAPAAASPEGEAPK.A
15852	35 – 54	904.9369	1807.8592	1807.8587	0.23	0	57	4.6e-06	1	U	R.AAEEAAAAPAAASPEGEAPK.A
15853	35 – 54	904.9376	1807.8607	1807.8587	1.07	0	54	9e-06	1	U	R.AAEEAAAAPAAASPEGEAPK.A
15855	35 – 54	904.9382	1807.8619	1807.8587	1.74	0	51	1.7e-05	1	U	R.AAEEAAAAPAAASPEGEAPK.A
15856	35 – 54	603.6280	1807.8621	1807.8587	1.85	0	77	5.8e-08	1	U	R.AAEEAAAAPAAASPEGEAPK.A
15857	35 – 54	603.6280	1807.8623	1807.8587	1.97	0	81	2.7e-08	1	U	R.AAEEAAAAPAAASPEGEAPK.A
4591	55 – 65	523.8212	1045.6279	1045.6284	-0.46	0	21	0.02	1	U	K.AAAKPPPIGPK.R
4592	55 – 65	523.8216	1045.6286	1045.6284	0.21	0	8	0.39	1	U	K.AAAKPPPIGPK.R
4593	55 – 65	523.8217	1045.6288	1045.6284	0.43	0	23	0.015	1	U	K.AAAKPPPIGPK.R
4594	55 – 65	523.8220	1045.6294	1045.6284	0.98	0	19	0.041	1	U	K.AAAKPPPIGPK.R
4595	55 – 65	523.8221	1045.6296	1045.6284	1.15	0	22	0.024	1	U	K.AAAKPPPIGPK.R
4596	55 – 65	523.8221	1045.6297	1045.6284	1.26	0	19	0.046	1	U	K.AAAKPPPIGPK.R
4598	55 – 65	523.8224	1045.6303	1045.6284	1.82	0	21	0.027	1	U	K.AAAKPPPIGPK.R
4599	55 – 65	523.8226	1045.6306	1045.6284	2.09	0	4	1.5	1	U	K.AAAKPPPIGPK.R
9117	82 – 94	424.2263	1269.6569	1269.6565	0.33	0	22	0.0085	1	U	K.GVGSVVAVDQDPK.T
9118	82 – 94	635.8358	1269.6571	1269.6565	0.49	0	32	0.0011	1	U	K.GVGSVVAVDQDPK.T
9119	82 – 94	635.8362	1269.6578	1269.6565	1.00	0	50	2.3e-05	1	U	K.GVGSVVAVDQDPK.T
9120	82 – 94	424.2266	1269.6578	1269.6565	1.04	0	13	0.067	1	U	K.GVGSVVAVDQDPK.T
18431	82 – 102	561.0648	2240.2301	2240.2278	1.05	2	4	2	1	U	K.GVGSVVAVDQDPKTRYPVVVR.F
3702	95 – 102	495.2981	988.5816	988.5818	-0.17	1	18	0.085	1	U	K.TRYPVVVR.F
3704	95 – 102	495.2985	988.5825	988.5818	0.70	1	20	0.013	1	U	K.TRYPVVVR.F
303	97 – 102	366.7239	731.4333	731.4330	0.33	0	24	0.024	1	U	R.YPVVVR.F
304	97 – 102	366.7243	731.4341	731.4330	1.54	0	30	0.0096	1	U	R.YPVVVR.F
305	97 – 102	366.7246	731.4346	731.4330	2.12	0	26	0.025	1	U	R.YPVVVR.F
306	97 – 102	366.7246	731.4346	731.4330	2.18	0	2	0.69	1	U	R.YPVVVR.F
20249	103 – 125	872.7592	2615.2558	2615.2503	2.09	1	20	0.013	1	U	R.FNKVNYANVSTNNYALDEIQEVA.-
20250	103 – 125	1308.6361	2615.2576	2615.2503	2.77	1	17	0.028	1	U	R.FNKVNYANVSTNNYALDEIQEVA.-
18339	106 – 125	1114.0310	2226.0474	2226.0440	1.53	0	45	6.2e-05	1	U	K.VNYANVSTNNYALDEIQEVA.-
18340	106 – 125	743.0241	2226.0505	2226.0440	2.90	0	21	0.012	1	U	K.VNYANVSTNNYALDEIQEVA.-

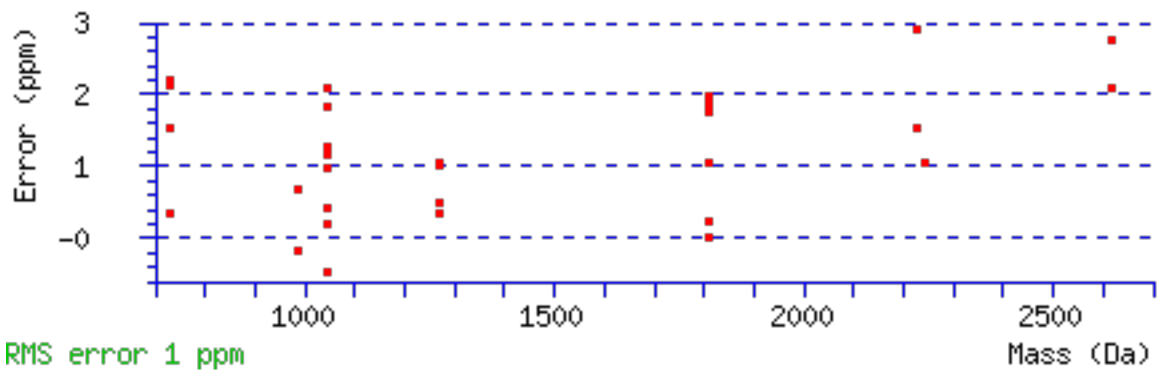

Mascot: http://www.matrixscience.com/

MASCOT Search Results

Protein View: tr|A0A0K9RU56|A0A0K9RU56_SPIOL

>tr|A0A0K9RU56|A0A0K9RU56_SPIOL Chlorophyll a-b binding protein, chloroplastic OS=Spinacia oleracea OX=3562 GN=SOVF_028160 PE=3 SV=1

**Database:**	Uni-Spinach
**Score:**	431
**Nominal mass (M_r):**	29397
**Calculated pI:**	6.90

Sequence similarity is available as an NCBI BLAST search of tr|A0A0K9RU56|A0A0K9RU56_SPIOL against nr.

Search parameters

**MS data file:**	\\128.97.66.218\tank\windowsVM\Bill Cramer\MGF\wc_QE_021417_Cramer_Gel_3S_4a.mgf
**Enzyme:**	Trypsin: cuts C-term side of KR unless next residue is P.
**Fixed modifications:**	Carbamidomethyl (C)
**Variable modifications:**	Oxidation (M)

Protein sequence coverage: 27%

Matched peptides shown in ***bold red***.

**1**	MSSVCASSAT	TAVCASSASS	QKKGNVLGAT	RASFLGGKKL	RDSSKYRTPV
**51**	SSNSFSVSAA	AAEPERPIWF	PGSTPPSWLD	GSLPADFGFD	PLGFGSDPET
**101**	LR**WMVQSEIV**	**HCRWAMLGAA**	**GIFIPEFLTK**	LGILNTPSWY	TAGELDYFTD
**151**	TTTLFVVELV	LIGWAEGR**RW**	**ADILKPGCVN**	**TDPIFPNNKL**	**TGTDVGYPGG**
**201**	**LWFDPLGWGS**	**GSPQK**VKELR	TKEIKNGRLA	MLAVMGAWFQ	HIYTGTGPID
**251**	NLFAHLADPG	HATIFSAFTP	K		

Unformatted sequence string: 271 residues (for pasting into other applications).

Sort peptides by ● Residue Number ○ Increasing Mass ○ Decreasing Mass

Show predicted peptides also

Query	Start – End	Observed	Mr(expt)	Mr(calc)	ppm	M	Score	Expect	Rank	U	Peptide
12050	103 – 113	722.8447	1443.6748	1443.6751	-0.19	0	47	8.9e-05	1	U	R.WMVQSEIVHCR.W
12051	103 – 113	482.2326	1443.6761	1443.6751	0.66	0	31	0.0039	1	U	R.WMVQSEIVHCR.W
12054	103 – 113	482.2332	1443.6779	1443.6751	1.90	0	18	0.081	1	U	R.WMVQSEIVHCR.W
12317	103 – 113	487.5630	1459.6670	1459.6700	-2.05	0	23	0.018	1	U	R.WMVQSEIVHCR.W + Oxidation (M)
12318	103 – 113	730.8424	1459.6702	1459.6700	0.14	0	28	0.0067	1	U	R.WMVQSEIVHCR.W + Oxidation (M)
12320	103 – 113	487.5652	1459.6737	1459.6700	2.49	0	47	0.00011	1	U	R.WMVQSEIVHCR.W + Oxidation (M)
12321	103 – 113	730.8444	1459.6742	1459.6700	2.82	0	55	1.7e-05	1	U	R.WMVQSEIVHCR.W + Oxidation (M)
12322	103 – 113	487.5655	1459.6747	1459.6700	3.17	0	28	0.0083	1	U	R.WMVQSEIVHCR.W + Oxidation (M)
16353	114 – 130	933.0061	1863.9976	1863.9957	1.04	0	59	2.7e-06	1	U	R.WAMLGAAGIFIPEFLTK.L
16456	114 – 130	941.0059	1879.9973	1879.9906	3.56	0	91	2.7e-09	1	U	R.WAMLGAAGIFIPEFLTK.L + Oxidation (M)
16457	114 – 130	627.6736	1879.9990	1879.9906	4.44	0	41	0.00016	1	U	R.WAMLGAAGIFIPEFLTK.L + Oxidation (M)
19649	169 – 189	819.0898	2454.2476	2454.2478	-0.088	1	8	0.17	1	U	R.RWADILKPGCVNTDPIFPNNK.L
18872	170 – 189	767.0566	2298.1480	2298.1467	0.56	0	30	0.0014	1	U	R.WADILKPGCVNTDPIFPNNK.L
20599	190 – 215	898.1090	2691.3050	2691.2970	3.00	0	76	7.7e-08	1	U	K.LTGTDVGYPGGLWFDPLGWGSGSPQK.V
20601	190 – 215	1346.6603	2691.3060	2691.2970	3.34	0	87	6.3e-09	1	U	K.LTGTDVGYPGGLWFDPLGWGSGSPQK.V

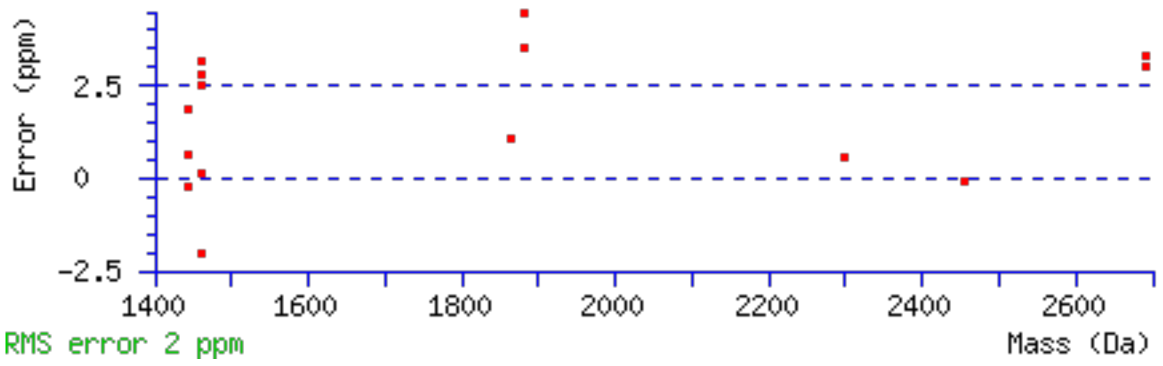

Mascot: http://www.matrixscience.com/

MASCOT Search Results

Protein View: tr|A0A0K9RHQ2|A0A0K9RHQ2_SPIOL

>tr|A0A0K9RHQ2|A0A0K9RHQ2_SPIOL Uncharacterized protein OS=Spinacia oleracea OX=3562 GN=SOVF_071290 PE=4 SV=1

**Database:**	Uni-Spinach
**Score:**	349
**Nominal mass (M_r):**	18374
**Calculated pI:**	9.33

Sequence similarity is available as an NCBI BLAST search of tr|A0A0K9RHQ2|A0A0K9RHQ2_SPIOL against nr.

Search parameters

**MS data file:**	\\128.97.66.218\tank\windowsVM\Bill Cramer\MGF\wc_QE_021417_Cramer_Gel_3S_4a.mgf
**Enzyme:**	Trypsin: cuts C-term side of KR unless next residue is P.
**Fixed modifications:**	Carbamidomethyl (C)
**Variable modifications:**	Oxidation (M)

Protein sequence coverage: 23%

Matched peptides shown in ***bold red***.

```
  1 MAAATASLSS TLLAPCSSKQ PQPQQQHQHQ QLKCKSFSGL RPLKLNISSN
 51 NSSSSLSMSS ARRSMTCRAE LSPSLVISLS TGLSLFLGRF VFFNFQRENM
101 AKQVPEQNGM SHFEAGDTRA KEYVSLLKSN DPVGFNIVDV LAWGSIGHIV
151 AYYILATASN GYDPSFF
```

Unformatted sequence string: 167 residues (for pasting into other applications).

Sort peptides by ◉ Residue Number ○ Increasing Mass ○ Decreasing Mass

Show predicted peptides also

Query	Start – End	Observed	Mr(expt)	Mr(calc)	ppm	M	Score	Expect	Rank	U	Peptide
5738	90 – 97	552.7857	1103.5568	1103.5553	1.43	0	44	7e-05	1	U	R.FVFFNFQR.E
14756	90 – 102	559.9471	1676.8195	1676.8133	3.68	1	24	0.006	1	U	R.FVFFNFQRENMAK.Q
14890	90 – 102	565.2783	1692.8130	1692.8082	2.79	1	4	0.43	1	U	R.FVFFNFQRENMAK.Q + Oxidation (M)
16613	103 – 119	634.9456	1901.8150	1901.8326	-9.28	0	53	9.6e-06	1	U	K.QVPEQNGMSHFEAGDTR.A
16614	103 – 119	951.9227	1901.8309	1901.8326	-0.89	0	52	1.9e-05	1	U	K.QVPEQNGMSHFEAGDTR.A
16615	103 – 119	951.9241	1901.8336	1901.8326	0.54	0	8	0.42	1	U	K.QVPEQNGMSHFEAGDTR.A
16616	103 – 119	634.9521	1901.8344	1901.8326	0.95	0	54	1.1e-05	1	U	K.QVPEQNGMSHFEAGDTR.A
16617	103 – 119	634.9524	1901.8355	1901.8326	1.50	0	48	5.2e-05	1	U	K.QVPEQNGMSHFEAGDTR.A
16697	103 – 119	640.2818	1917.8236	1917.8275	-2.07	0	1	1.6	1	U	K.QVPEQNGMSHFEAGDTR.A + Oxidation (M)
16698	103 – 119	640.2831	1917.8273	1917.8275	-0.11	0	34	0.00086	1	U	K.QVPEQNGMSHFEAGDTR.A + Oxidation (M)
16699	103 – 119	640.2837	1917.8292	1917.8275	0.88	0	44	7.1e-05	1	U	K.QVPEQNGMSHFEAGDTR.A + Oxidation (M)
16700	103 – 119	640.2840	1917.8303	1917.8275	1.44	0	51	1.5e-05	1	U	K.QVPEQNGMSHFEAGDTR.A + Oxidation (M)
16701	103 – 119	640.2845	1917.8316	1917.8275	2.13	0	44	7.7e-05	1	U	K.QVPEQNGMSHFEAGDTR.A + Oxidation (M)
16702	103 – 119	640.2846	1917.8319	1917.8275	2.25	0	50	2e-05	1	U	K.QVPEQNGMSHFEAGDTR.A + Oxidation (M)
4674	120 – 128	525.8142	1049.6139	1049.6121	1.73	1	23	0.014	1	U	R.AKEYVSLLK.S
4676	120 – 128	350.8792	1049.6157	1049.6121	3.47	1	11	0.25	1	U	R.AKEYVSLLK.S
4677	120 – 128	350.8792	1049.6158	1049.6121	3.60	1	14	0.11	1	U	R.AKEYVSLLK.S
1802	122 – 128	426.2479	850.4812	850.4800	1.37	0	4	1.5	1	U	K.EYVSLLK.S
1803	122 – 128	426.2480	850.4814	850.4800	1.60	0	21	0.026	1	U	K.EYVSLLK.S

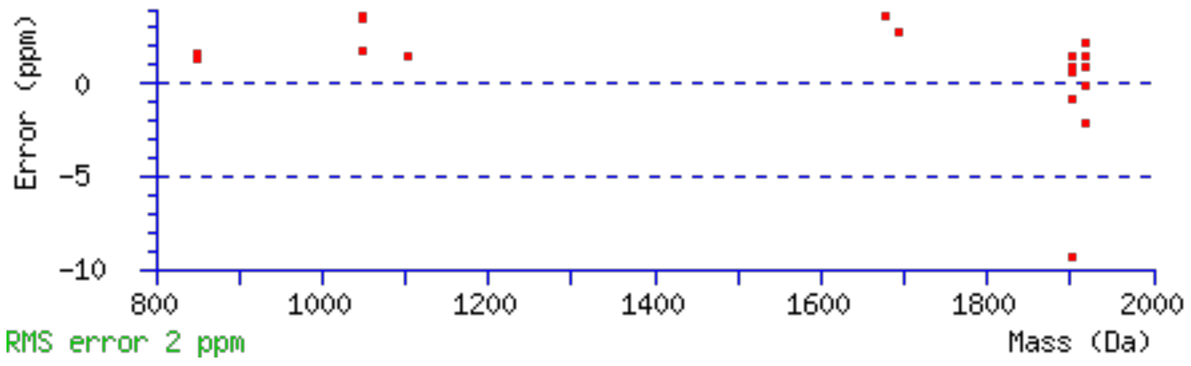

Mascot: http://www.matrixscience.com/

MASCOT Search Results

Protein View: tr|A0A0K9RRT4|A0A0K9RRT4_SPIOL

>tr|A0A0K9RRT4|A0A0K9RRT4_SPIOL Chlorophyll a-b binding protein, chloroplastic OS=Spinacia oleracea OX=3562 GN=SOVF_041770 PE=3 SV=1

**Database:**	Uni-Spinach
**Score:**	348
**Nominal mass (M_r):**	26370
**Calculated pI:**	5.97

Sequence similarity is available as **an NCBI BLAST search of tr|A0A0K9RRT4|A0A0K9RRT4_SPIOL against nr**.

Search parameters

**MS data file:**	\\128.97.66.218\tank\windowsVM\Bill Cramer\MGF\wc_QE_021417_Cramer_Gel_3S_4a.mgf
**Enzyme:**	Trypsin: cuts C-term side of KR unless next residue is P.
**Fixed modifications:**	Carbamidomethyl (C)
**Variable modifications:**	Oxidation (M)

Protein sequence coverage: 30%

Matched peptides shown in ***bold red***.

1 MASNALMSCG IAAVFPSSLS SSKSK**FAASV** **PLGNVSCNAS** **SR**FTMSAEWM
51 PGQPRPAHLD GSAPGDFGFD PLGLGEVPEN LER**FKESELI** **HCRWAMLAVP**
101 **GILVPEALGL** **GNWVK**AQEWA ALPGGQATYL GNPVPWGNLP TILAIEFLAI
151 AFVEHQRSME KDSEK**KKYPG** **GAFDPLGYSK** **DPKKFEELK**L KEIKNGRLAL
201 LAFVGFCIQQ SAYPGTGPLE NLATHLADPW HNNIGDIVIP RAL

Unformatted sequence string: **243 residues** (for pasting into other applications).

Sort peptides by ◉ Residue Number ○ Increasing Mass ○ Decreasing Mass

Show predicted peptides also

Query	Start – End	Observed	Mr(expt)	Mr(calc)	ppm	M	Score	Expect	Rank	U	Peptide
15282	26 – 42	868.9290	1735.8434	1735.8312	7.08	0	1	0.81	1	U	K.FAASVPLGNVSCNASSR.F
9968	84 – 93	440.2243	1317.6512	1317.6499	0.93	1	36	0.00043	1	U	R.FKESELIHCR.W
9969	84 – 93	440.2250	1317.6531	1317.6499	2.37	1	44	7.9e-05	1	U	R.FKESELIHCR.W
4485	86 – 93	522.2505	1042.4864	1042.4866	-0.16	0	22	0.019	1	U	K.ESELIHCR.W
4486	86 – 93	522.2512	1042.4878	1042.4866	1.19	0	39	0.00044	1	U	K.ESELIHCR.W
19208	94 – 115	784.1053	2349.2941	2349.2919	0.94	0	37	0.00077	1	U	R.WAMLAVPGILVPEALGLGNWVK.A + Oxidation (M)
14283	166 – 180	814.4280	1626.8414	1626.8406	0.49	2	31	0.0013	1	U	K.KKYPGGAFDPLGYSK.D
14284	166 – 180	543.2881	1626.8425	1626.8406	1.16	2	34	0.00065	1	U	K.KKYPGGAFDPLGYSK.D
14285	166 – 180	543.2883	1626.8431	1626.8406	1.55	2	19	0.016	1	U	K.KKYPGGAFDPLGYSK.D
12908	167 – 180	750.3798	1498.7451	1498.7456	-0.34	1	59	3e-06	1	U	K.KYPGGAFDPLGYSK.D
12909	167 – 180	750.3805	1498.7465	1498.7456	0.60	1	42	0.00011	1	U	K.KYPGGAFDPLGYSK.D
12911	167 – 180	750.3808	1498.7471	1498.7456	0.98	1	4	0.41	1	U	K.KYPGGAFDPLGYSK.D
12912	167 – 180	500.5898	1498.7476	1498.7456	1.30	1	28	0.0026	1	U	K.KYPGGAFDPLGYSK.D
12913	167 – 180	500.5901	1498.7486	1498.7456	1.97	1	23	0.0074	1	U	K.KYPGGAFDPLGYSK.D
12914	167 – 180	500.5904	1498.7493	1498.7456	2.47	1	20	0.014	1	U	K.KYPGGAFDPLGYSK.D
12915	167 – 180	500.5907	1498.7504	1498.7456	3.19	1	38	0.00029	1	U	K.KYPGGAFDPLGYSK.D
10847	168 – 180	686.3334	1370.6523	1370.6507	1.19	0	14	0.051	1	U	K.YPGGAFDPLGYSK.D
10849	168 – 180	686.3342	1370.6539	1370.6507	2.39	0	57	4.3e-06	1	U	K.YPGGAFDPLGYSK.D
10850	168 – 180	686.3347	1370.6549	1370.6507	3.06	0	51	1.6e-05	1	U	K.YPGGAFDPLGYSK.D
15065	168 – 183	571.2832	1710.8278	1710.8253	1.43	1	2	0.67	1	U	K.YPGGAFDPLGYSKDPK.K
884	184 – 189	397.2268	792.4390	792.4381	1.16	1	5	0.32	1	U	K.KFEELK.L

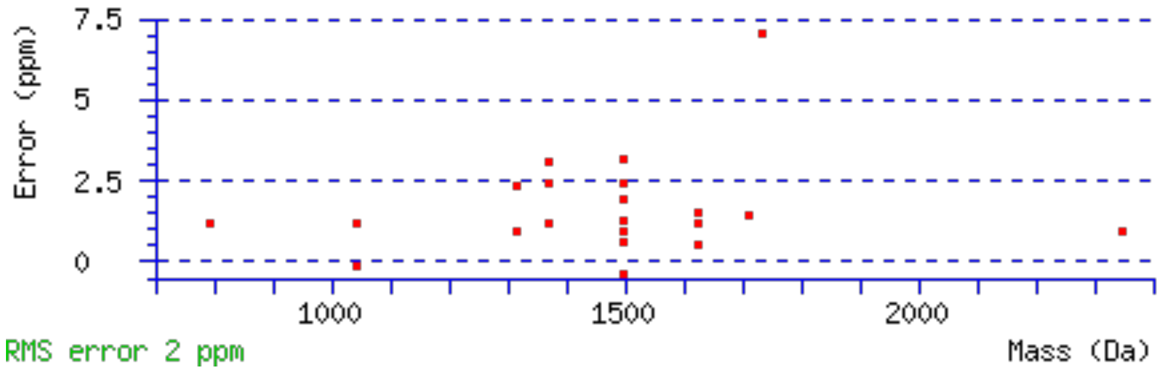

Mascot: http://www.matrixscience.com/

(MATRIX SCIENCE) MASCOT Search Results

Protein View: sp|P00833|ATPE_SPIOL

>sp|P00833|ATPE_SPIOL ATP synthase epsilon chain, chloroplastic OS=Spinacia oleracea OX=3562 GN=atpE PE=1 SV=1

**Database:**	Uni-Spinach
**Score:**	344
**Nominal mass (M_r):**	14748
**Calculated pI:**	6.59

Sequence similarity is available as **an NCBI BLAST search of sp|P00833|ATPE_SPIOL against nr**.

Search parameters

**MS data file:**	\\128.97.66.218\tank\windowsVM\Bill Cramer\MGF\wc_QE_021417_Cramer_Gel_3S_4a.mgf
**Enzyme:**	Trypsin: cuts C-term side of KR unless next residue is P.
**Fixed modifications:**	**Carbamidomethyl (C)**
**Variable modifications:**	**Oxidation (M)**

Protein sequence coverage: 62%

Matched peptides shown in ***bold red***.

```
  1 MTLNLCVLTP NRSIWNSEVK  EIILSTNSGQ  IGVLPNHAPT  ATAVDIGILR
 51 IRLNDQWLTL ALMGGFARIG NNEITILVND AERGSDIDPQ EAQQTLEIAE
101 ANLRKAEGKR QKIEANLALR RARTRVEASN TISS
```

Unformatted sequence string: **134 residues** (for pasting into other applications).

Sort peptides by ◉ Residue Number ○ Increasing Mass ○ Decreasing Mass

Show predicted peptides also

Query	Start – End	Observed	Mr(expt)	Mr(calc)	ppm	M	Score	Expect	Rank	U	Peptide
9619	2 – 12	650.8570	1299.6995	1299.6969	2.03	0	37	0.00034	1	U	M.TLNLCVLTPNR.S
15957	53 – 68	607.9828	1820.9267	1820.9243	1.28	0	12	0.075	1	U	R.LNDQWLTLALMGGFAR.I + Oxidation (M)
15958	53 – 68	911.4712	1820.9279	1820.9243	1.95	0	93	2.1e-09	1	U	R.LNDQWLTLALMGGFAR.I + Oxidation (M)
14676	69 – 83	835.9404	1669.8663	1669.8635	1.66	0	103	2.1e-10	1	U	R.IGNNEITILVNDAER.G
14677	69 – 83	557.6295	1669.8667	1669.8635	1.95	0	64	9e-07	1	U	R.IGNNEITILVNDAER.G
18859	84 – 104	766.7130	2297.1172	2297.1135	1.62	0	106	1.3e-10	1	U	R.GSDIDPQEAQQTLEIAEANLR.K
6728	111 – 120	385.9004	1154.6792	1154.6771	1.84	1	11	0.36	1	U	R.QKIEANLALR.R
6729	111 – 120	578.3472	1154.6798	1154.6771	2.33	1	16	0.1	1	U	R.QKIEANLALR.R
6869	124 – 134	582.7971	1163.5797	1163.5782	1.31	1	27	0.0033	1	U	R.TRVEASNTISS.-

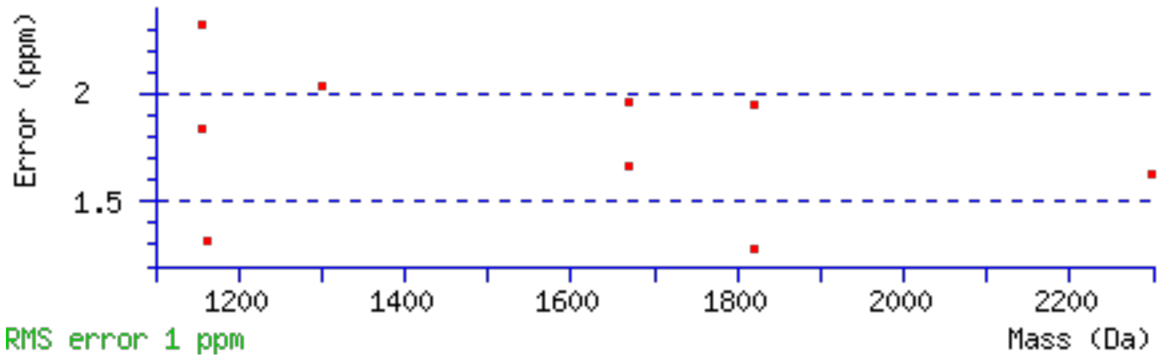

MASCOT Search Results

Protein View: tr|A0A0K9RFL8|A0A0K9RFL8_SPIOL

>tr|A0A0K9RFL8|A0A0K9RFL8_SPIOL AAA domain-containing protein OS=Spinacia oleracea OX=3562 GN=SOVF_078600 PE=3 SV=1

**Database:**	Uni-Spinach
**Score:**	330
**Nominal mass (M_r):**	75763
**Calculated pI:**	5.65

Sequence similarity is available as an NCBI BLAST search of tr|A0A0K9RFL8|A0A0K9RFL8_SPIOL against nr.

Search parameters

**MS data file:**	\\128.97.66.218\tank\windowsVM\Bill Cramer\MGF\wc_QE_021417_Cramer_Gel_3S_4a.mgf
**Enzyme:**	Trypsin: cuts C-term side of KR unless next residue is P.
**Fixed modifications:**	Carbamidomethyl (C)
**Variable modifications:**	Oxidation (M)

Protein sequence coverage: 29%

Matched peptides shown in ***bold red***.

1 MAANPLLSSS FLGNGVLFYP PTPKTTKPIL PSSSRRKQFI ITQSLIPNSQ
51 NPKFLSKSAL TALLLSSTLA SQQAFAADNL SPPPQSPQVI EAQPTNPGLP
101 NSSPFSQNLV LNAPKPLDPD LPEGSQWRYS EFLNAVKKGK VERVRFSK**DG**
151 **SVLQLTAVDG** **KR**ASVVVPND PDLIDILAMN GVDISVSEGE GGNGLFGFIG
201 NLLFPLLAFA GLFFLFRR**AQ** **GGPGGPGGLG** **GPMDFGR**SKS K**FQEVPETGV**
251 **SFADVAGADQ** **AK**LELQEVVD FLK**NPDKYTA** **LGAK**IPKGCL LVGPPGTGKT
301 LLARAVAGEA GTPFFSCAAS EFVELFVGVG ASRVRDLFEK AKSK**APCIVF**
351 **IDEIDAVGR**Q RGAGMGGGND EREQTINQLL TEMDGFSGNS GVIVLAATNR
401 PDVLDSALLR PGRFDRQVTV DRPDVAGRVK **ILQVHSR**GKA LAKDVDFDKV
451 AR**RTPGFTGA** **DLQNLMNEAA** **ILAAR**RELK**E** **ISKDEISDAL** **ER**IIAGPEKK
501 NAVVSEEKKR **LVAYHEAGHA** **LVGALMPEYD** **PVAK**ISIIPR GQAGGLTFFA
551 PSEER**LESGL** **YSRSYLENQM** **AVALGGR**VAE EVIFGENNVT TGASSDFMQV
601 SRVARQMVER FGFSKKIGQL AVGGAGGNPF LGGQMSSAK**D** **YSMATADIVD**
651 **GEVRELVEVA** **YK**R**ATEIINT** **HIDILHK**LAQ LLIEKETIDG EEFMSLFIDG
701 QAELYVS

Unformatted sequence string: 707 residues (for pasting into other applications).

Sort peptides by ◉ Residue Number ○ Increasing Mass ○ Decreasing Mass

Show predicted peptides also

Query	Start – End	Observed	Mr(expt)	Mr(calc)	ppm	M	Score	Expect	Rank	U	Peptide
9664	149 – 161	651.8496	1301.6846	1301.6827	1.49	0	68	4.4e-07	1	U	K.DGSVLQLTAVDGK.R
12290	149 – 162	486.9356	1457.7850	1457.7838	0.80	1	2	1.1	2	U	K.DGSVLQLTAVDGKR.A
14817	219 – 237	842.9000	1683.7854	1683.7788	3.94	0	4	0.45	1	U	R.AQGGPGGPGGLGGPMDFGR.S
14958	219 – 237	850.8986	1699.7826	1699.7737	5.27	0	12	0.085	1	U	R.AQGGPGGPGGLGGPMDFGR.S + Oxidation (M)
18026	242 – 262	722.6854	2165.0344	2165.0277	3.08	0	41	0.00013	1	U	K.FQEVPETGVSFADVAGADQAK.L
7123	274 – 284	393.2123	1176.6150	1176.6139	0.99	1	18	0.021	1	U	K.NPDKYTALGAK.I
14722	345 – 359	837.9300	1673.8455	1673.8447	0.49	0	7	0.21	1	U	K.APCIVFIDEIDAVGR.Q
1812	431 – 437	426.7565	851.4985	851.4977	0.93	0	15	0.098	1	U	K.ILQVHSR.G
19569	453 – 475	810.7623	2429.2651	2429.2485	6.85	1	9	0.14	1	U	R.RTPGFTGADLQNLMNEAAILAAR.R
18674	454 – 475	758.7260	2273.1562	2273.1474	3.86	0	86	8.4e-09	1	U	R.TPGFTGADLQNLMNEAAILAAR.R
12993	480 – 492	752.8798	1503.7450	1503.7416	2.28	1	30	0.0015	1	U	K.EISKDEISDALER.I
12994	480 – 492	502.2559	1503.7458	1503.7416	2.81	1	26	0.0034	1	U	K.EISKDEISDALER.I
4605	484 – 492	524.2527	1046.4908	1046.4880	2.73	0	8	0.17	1	U	K.DEISDALER.I
20054	511 – 534	642.5809	2566.2944	2566.2890	2.10	0	23	0.0069	1	U	R.LVAYHEAGHALVGALMPEYDPVAK.I + Oxidation (M)
2800	556 – 563	462.7437	923.4729	923.4712	1.85	0	6	0.25	1	U	R.LESGLYSR.S
13032	564 – 577	754.8841	1507.7536	1507.7453	5.55	0	65	8.6e-07	1	U	R.SYLENQMAVALGGR.V
13264	564 – 577	762.8770	1523.7395	1523.7402	-0.43	0	11	0.087	1	U	R.SYLENQMAVALGGR.V + Oxidation (M)
13266	564 – 577	762.8785	1523.7424	1523.7402	1.44	0	33	0.00073	1	U	R.SYLENQMAVALGGR.V +

											Oxidation (M)
14405	640 - 654	821.3773	1640.7401	1640.7352	2.99	0	13	0.19	1	U	K.DYSMATADIVDGEVR.E
14561	640 - 654	829.3737	1656.7329	1656.7301	1.70	0	76	7.7e-08	1	U	K.DYSMATADIVDGEVR.E + Oxidation (M)
14562	640 - 654	553.2521	1656.7345	1656.7301	2.63	0	21	0.028	1	U	K.DYSMATADIVDGEVR.E + Oxidation (M)
3152	655 - 662	475.7641	949.5136	949.5120	1.64	0	7	0.87	1	U	R.ELVEVAYK.R
14168	664 - 677	539.9714	1616.8925	1616.8886	2.43	0	6	0.26	1	U	R.ATEIINTHIDILHK.L

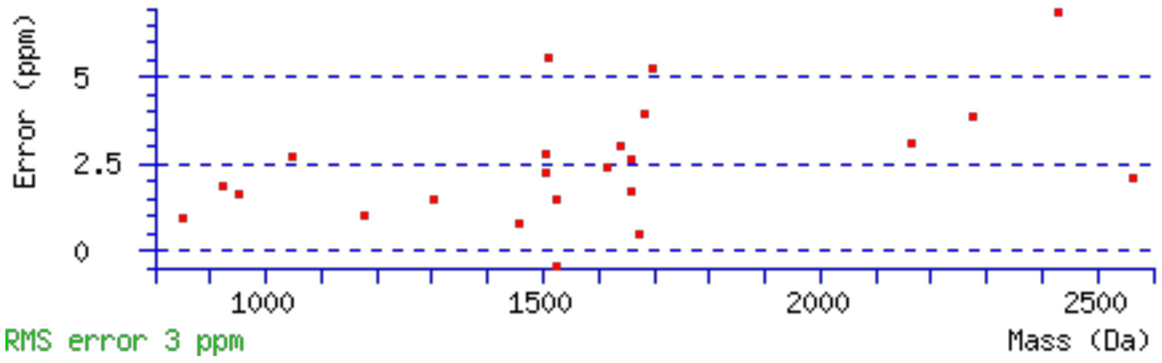

Mascot: http://www.matrixscience.com/

MASCOT Search Results

Protein View: tr|A0A0K9R772|A0A0K9R772_SPIOL

>tr|A0A0K9R772|A0A0K9R772_SPIOL Chlorophyll a-b binding protein, chloroplastic OS=Spinacia oleracea OX=3562 GN=SOVF_099030 PE=3 SV=1

**Database:**	Uni-Spinach
**Score:**	310
**Nominal mass (M_r):**	27852
**Calculated pI:**	6.10

Sequence similarity is available as **an NCBI BLAST search of tr|A0A0K9R772|A0A0K9R772_SPIOL against nr**.

Search parameters

**MS data file:**	\\128.97.66.218\tank\windowsVM\Bill Cramer\MGF\wc_QE_021417_Cramer_Gel_3S_4a.mgf
**Enzyme:**	Trypsin: cuts C-term side of KR unless next residue is P.
**Fixed modifications:**	Carbamidomethyl (C)
**Variable modifications:**	Oxidation (M)

Protein sequence coverage: 32%

Matched peptides shown in ***bold red***.

```
  1 MAAVTTQASI AGFRPCASKP RFLTGVPGKL NKESSGVRLP STSSTTSFKV
 51 EAKKGEWLPG LASPGYLTGS LPGDNGFDPL ALAEDPENLR WFVQAELVNG
101 RWAMLGVAGM LLPEVFTSIG IIDVPKWYDA GKSEYFASSS TLFVIEFILF
151 HYVEIRRWQD IKNPGCVNQD PIFKQYSLPP NECGYPGGIF NPLNFAPTTE
201 AKEKELANGR LAMLAFLGFI VQHNVTGKGP FDNLQQHLSD PWHNTIIQTF
251 GGN
```

Unformatted sequence string: **253 residues** (for pasting into other applications).

Sort peptides by ◉ Residue Number ○ Increasing Mass ○ Decreasing Mass

Show predicted peptides also

Query	Start – End	Observed	Mr(expt)	Mr(calc)	ppm	M	Score	Expect	Rank	U	Peptide
9972	91 – 101	659.8499	1317.6853	1317.6830	1.77	0	55	6.9e-06	1	U	R.WFVQAELVNGR.W
9974	91 – 101	440.2361	1317.6864	1317.6830	2.63	0	29	0.0038	1	U	R.WFVQAELVNGR.W
355	127 – 132	370.1748	738.3350	738.3337	1.79	0	13	0.17	1	U	K.WYDAGK.S
356	127 – 132	370.1749	738.3353	738.3337	2.13	0	3	1.7	1	U	K.WYDAGK.S
1710	157 – 162	423.2356	844.4566	844.4555	1.25	1	11	0.4	1	U	R.RWQDIK.N
17522	158 – 174	687.0069	2057.9988	2057.9993	-0.23	1	22	0.0081	1	U	R.WQDIKNPGCVNQDPIFK.Q
17523	158 – 174	1030.0082	2058.0018	2057.9993	1.24	1	4	0.39	1	U	R.WQDIKNPGCVNQDPIFK.Q
17524	158 – 174	687.0086	2058.0041	2057.9993	2.32	1	12	0.068	1	U	R.WQDIKNPGCVNQDPIFK.Q
11106	163 – 174	694.8361	1387.6576	1387.6554	1.53	0	60	2.3e-06	1	U	K.NPGCVNQDPIFK.Q
11108	163 – 174	694.8366	1387.6587	1387.6554	2.38	0	57	9.7e-06	1	U	K.NPGCVNQDPIFK.Q
11109	163 – 174	694.8373	1387.6600	1387.6554	3.32	0	10	0.44	1	U	K.NPGCVNQDPIFK.Q
21436	175 – 202	1028.1619	3081.4638	3081.4542	3.13	0	35	0.00056	1	U	K.QYSLPPNECGYPGGIFNPLNFAPTTEAK.E
16998	211 – 228	980.0500	1958.0854	1958.0812	2.16	0	57	6.3e-06	1	U	R.LAMLAFLGFIVQHNVTGK.G
16999	211 – 228	653.7027	1958.0861	1958.0812	2.54	0	47	6.4e-05	1	U	R.LAMLAFLGFIVQHNVTGK.G
17112	211 – 228	659.0348	1974.0825	1974.0761	3.26	0	20	0.012	1	U	R.LAMLAFLGFIVQHNVTGK.G + Oxidation (M)
17113	211 – 228	988.0494	1974.0843	1974.0761	4.16	0	75	8.4e-08	1	U	R.LAMLAFLGFIVQHNVTGK.G + Oxidation (M)

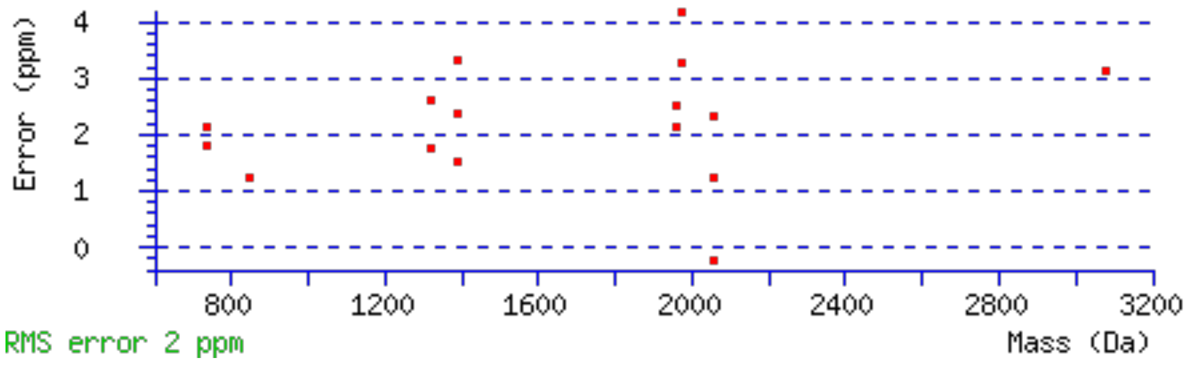

Mascot: http://www.matrixscience.com/

MASCOT Search Results

Protein View: tr|A0A0K9QQW2|A0A0K9QQW2_SPIOL

>tr|A0A0K9QQW2|A0A0K9QQW2_SPIOL Uncharacterized protein OS=Spinacia oleracea OX=3562 GN=SOVF_151500 PE=3 SV=1

**Database:**	Uni-Spinach
**Score:**	301
**Nominal mass (M_r):**	24444
**Calculated pI:**	6.00

Sequence similarity is available as **an NCBI BLAST search of tr|A0A0K9QQW2|A0A0K9QQW2_SPIOL against nr**.

Search parameters

**MS data file:**	\\128.97.66.218\tank\windowsVM\Bill Cramer\MGF\wc_QE_021417_Cramer_Gel_3S_4a.mgf
**Enzyme:**	Trypsin: cuts C-term side of KR unless next residue is P.
**Fixed modifications:**	Carbamidomethyl (C)
**Variable modifications:**	Oxidation (M)

Protein sequence coverage: 39%

Matched peptides shown in ***bold red***.

```
  1 MANMLVASSS KTLPTTTTTT ITPKPKFPLL KTPLLKLSPP QLPPLKHLNL
 51 SVLKSAAITA TPLTLSFLLP YPSLAEEIEK ASLFDFNLTL PIIMAEFLFL
101 MFALDKIYYT PLGDFMDKRD ASIKEQLSGV KDTSSEVKQL EEQANAVMRA
151 ARAEISAALN KMKKETQLEV EAKLAEGRKK IEVELQEALG SLEQQKEDTI
201 KSLDSQISAL SDDIVKKVLP VS
```

Unformatted sequence string: 222 residues (for pasting into other applications).

Sort peptides by ● Residue Number ○ Increasing Mass ○ Decreasing Mass

Show predicted peptides also

Query	Start – End	Observed	Mr(expt)	Mr(calc)	ppm	M	Score	Expect	Rank	U	Peptide
14319	107 – 119	545.6013	1633.7822	1633.7810	0.73	1	9	0.16	1	U	K.IYYTPLGDFMDKR.D + Oxidation (M)
9243	120 – 131	425.5701	1273.6885	1273.6878	0.56	1	21	0.01	1	U	R.DASIKEQLSGVK.D
536	125 – 131	380.7137	759.4128	759.4127	0.25	0	22	0.061	1	U	K.EQLSGVK.D
537	125 – 131	380.7139	759.4132	759.4127	0.78	0	3	0.53	1	U	K.EQLSGVK.D
9401	139 – 149	644.8205	1287.6264	1287.6241	1.78	0	27	0.0027	1	U	K.QLEEQANAVMR.A
9402	139 – 149	644.8214	1287.6283	1287.6241	3.23	0	5	0.35	1	U	K.QLEEQANAVMR.A
9698	139 – 149	652.8174	1303.6202	1303.6190	0.90	0	62	1.5e-06	1	U	K.QLEEQANAVMR.A + Oxidation (M)
9699	139 – 149	435.5477	1303.6213	1303.6190	1.77	0	0	0.91	1	U	K.QLEEQANAVMR.A + Oxidation (M)
2732	153 – 161	458.7592	915.5039	915.5025	1.52	0	5	0.31	1	U	R.AEISAALNK.M
7081	164 – 173	587.8194	1173.6242	1173.6241	0.11	1	1	0.73	1	U	K.KETQLEVEAK.L
4572	165 – 173	523.7716	1045.5286	1045.5291	-0.49	0	19	0.018	1	U	K.ETQLEVEAK.L
4573	165 – 173	523.7720	1045.5294	1045.5291	0.28	0	22	0.009	1	U	K.ETQLEVEAK.L
15911	181 – 196	605.3245	1812.9518	1812.9469	2.70	0	46	5.4e-05	1	U	K.IEVELQEALGSLEQQK.E
15912	181 – 196	907.4839	1812.9533	1812.9469	3.54	0	86	9.2e-09	1	U	K.IEVELQEALGSLEQQK.E
13958	202 – 216	795.9172	1589.8199	1589.8148	3.22	0	76	7.1e-08	1	U	K.SLDSQISALSDDIVK.K
13959	202 – 216	530.9478	1589.8217	1589.8148	4.31	0	43	9.3e-05	1	U	K.SLDSQISALSDDIVK.K
15151	202 – 217	573.6440	1717.9102	1717.9098	0.25	1	35	0.00049	1	U	K.SLDSQISALSDDIVKK.V

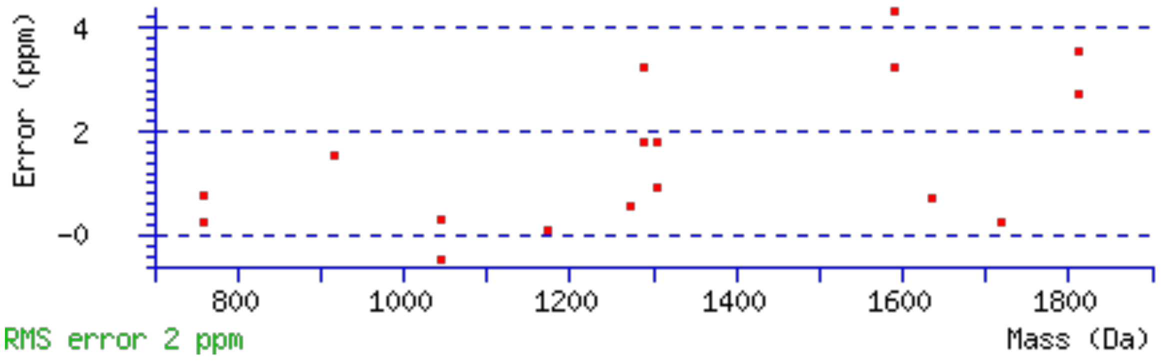

RMS error 2 ppm

Mascot: http://www.matrixscience.com/

MASCOT Search Results

Protein View: tr|A0A0K9RFL8|A0A0K9RFL8_SPIOL

>tr|A0A0K9RFL8|A0A0K9RFL8_SPIOL AAA domain-containing protein OS=Spinacia oleracea OX=3562 GN=SOVF_078600 PE=3 SV=1

**Database:**	Uni-Spinach
**Score:**	4138
**Nominal mass (M_r):**	75763
**Calculated pI:**	5.65

Sequence similarity is available as an NCBI BLAST search of tr|A0A0K9RFL8|A0A0K9RFL8_SPIOL against nr.

Search parameters

**MS data file:**	\\172.16.0.213\tank\windowsVM\Bill Cramer\052418\MGF\wc_QE_052418_Cramer_gel_4.mgf
**Enzyme:**	Trypsin: cuts C-term side of KR unless next residue is P.
**Fixed modifications:**	Carbamidomethyl (C)
**Variable modifications:**	Acetyl (K), Acetyl (Protein N-term), Deamidated (NQ), Oxidation (M)

Protein sequence coverage: 51%

Matched peptides shown in ***bold red***.

```
  1 MAANPLLSSS FLGNGVLFYP PTPKTTKPIL PSSSRRKQFI ITQSLIPNSQ
 51 NPKFLSKSAL TALLLSSTLA SQQAFAADNL SPPPQSPQVI EAQPTNPGLP
101 NSSPFSQNLV LNAPKPLDPD LPEGSQWRYS EFLNAVKKGK VERVRFSKDG
151 SVLQLTAVDG KRASVVVPND PDLIDILAMN GVDISVSEGE GGNGLFGFIG
201 NLLFPLLAFA GLFFLFRRAQ GGPGGPGGLG GPMDFGRSKS KFQEVPETGV
251 SFADVAGADQ AKLELQEVVD FLKNPDKYTA LGAKIPKGCL LVGPPGTGKT
301 LLARAVAGEA GTPFFSCAAS EFVELFVGVG ASRVRDLFEK AKSKAPCIVF
351 IDEIDAVGRQ RGAGMGGGND EREQTINQLL TEMDGFSGNS GVIVLAATNR
401 PDVLDSALLR PGRFDRQVTV DRPDVAGRVK ILQVHSRGKA LAKDVDFDKV
451 ARRTPGFTGA DLQNLMNEAA ILAARRELKE ISKDEISDAL ERIIAGPEKK
501 NAVVSEEKKR LVAYHEAGHA LVGALMPEYD PVAKISIIPR GQAGGLTFFA
551 PSEERLESGL YSRSYLENQM AVALGGRVAE EVIFGENNVT TGASSDFMQV
601 SRVARQMVER FGFSKKIGQL AVGGAGGNPF LGQQMSSAKD YSMATADIVD
651 GEVRELVEVA YKRATEIINT HIDILHKLAQ LLIEKETIDG EEFMSLFIDG
701 QAELYVS
```

Unformatted sequence string: 707 residues (for pasting into other applications).

Sort peptides by ◉ Residue Number ○ Increasing Mass ○ Decreasing Mass

Show predicted peptides also

Query	Start – End	Observed	Mr(expt)	Mr(calc)	ppm	M	Score	Expect	Rank	U	Peptide
4872	129 – 137	535.7790	1069.5435	1069.5444	-0.85	0	3	0.5	1	U	R.YSEFLNAVK.K
4873	129 – 137	535.7797	1069.5449	1069.5444	0.51	0	45	6.7e-05	1	U	R.YSEFLNAVK.K
4886	129 – 137	536.2714	1070.5282	1070.5284	-0.17	0	25	0.0049	1	U	R.YSEFLNAVK.K + Deamidated (NQ)
7487	129 – 138	599.8277	1197.6408	1197.6393	1.19	1	38	0.00025	1	U	R.YSEFLNAVKK.G
17725	146 – 162	607.6680	1819.9820	1819.9792	1.55	2	31	0.0013	1	U	R.FSKDGSVLQLTAVDGKR.A
9535	149 – 161	651.8489	1301.6833	1301.6827	0.45	0	58	3.5e-06	1	U	K.DGSVLQLTAVDGK.R
9538	149 – 161	651.8510	1301.6874	1301.6827	3.60	0	59	3.1e-06	1	U	K.DGSVLQLTAVDGK.R
12607	149 – 162	729.8989	1457.7832	1457.7838	-0.44	1	30	0.0017	1	U	K.DGSVLQLTAVDGKR.A
12608	149 – 162	486.9353	1457.7840	1457.7838	0.16	1	17	0.024	1	U	K.DGSVLQLTAVDGKR.A
12609	149 – 162	486.9355	1457.7848	1457.7838	0.69	1	42	0.00012	1	U	K.DGSVLQLTAVDGKR.A
12610	149 – 162	729.9010	1457.7875	1457.7838	2.51	1	81	2.8e-08	1	U	K.DGSVLQLTAVDGKR.A
18011	218 – 237	614.3012	1839.8818	1839.8799	1.02	1	77	6.4e-08	1	U	R.RAQGGPGGPGGLGGPMDFGR.S
18012	218 – 237	614.3013	1839.8822	1839.8799	1.27	1	5	0.5	1	U	R.RAQGGPGGPGGLGGPMDFGR.S
18021	218 – 237	921.4473	1840.8800	1840.8639	8.74	1	52	1.4e-05	1	U	R.RAQGGPGGPGGLGGPMDFGR.S + Deamidated (NQ)
18214	218 – 237	619.6327	1855.8764	1855.8748	0.87	1	57	4.4e-06	1	U	R.RAQGGPGGPGGLGGPMDFGR.S + Oxidation (M)
18215	218 – 237	619.6331	1855.8774	1855.8748	1.40	1	35	0.00051	1	U	R.RAQGGPGGPGGLGGPMDFGR.S + Oxidation (M)
18216	218 – 237	619.6331	1855.8776	1855.8748	1.48	1	50	2.1e-05	1	U	R.RAQGGPGGPGGLGGPMDFGR.S + Oxidation (M)
16080	219 – 237	842.8965	1683.7784	1683.7788	-0.24	0	112	3.4e-11	1	U	R.AQGGPGGPGGLGGPMDFGR.S

16081	219 - 237	562.2668	1683.7787	1683.7788	-0.040	0	30	0.0015	1	U	R.AQGGPGGPGGLGGPMDFGR.S
16082	219 - 237	562.2678	1683.7817	1683.7788	1.71	0	18	0.02	1	U	R.AQGGPGGPGGLGGPMDFGR.S
16084	219 - 237	842.8988	1683.7831	1683.7788	2.54	0	108	7.8e-11	1	U	R.AQGGPGGPGGLGGPMDFGR.S
16252	219 - 237	850.8938	1699.7731	1699.7737	-0.35	0	95	1.3e-09	1	U	R.AQGGPGGPGGLGGPMDFGR.S + Oxidation (M)
16253	219 - 237	850.8940	1699.7734	1699.7737	-0.16	0	93	2e-09	1	U	R.AQGGPGGPGGLGGPMDFGR.S + Oxidation (M)
16254	219 - 237	567.5987	1699.7742	1699.7737	0.28	0	55	7.5e-06	1	U	R.AQGGPGGPGGLGGPMDFGR.S + Oxidation (M)
16255	219 - 237	850.8947	1699.7748	1699.7737	0.63	0	93	2e-09	1	U	R.AQGGPGGPGGLGGPMDFGR.S + Oxidation (M)
16256	219 - 237	567.5992	1699.7758	1699.7737	1.22	0	58	3.8e-06	1	U	R.AQGGPGGPGGLGGPMDFGR.S + Oxidation (M)
16258	219 - 237	850.8961	1699.7776	1699.7737	2.32	0	84	1.2e-08	1	U	R.AQGGPGGPGGLGGPMDFGR.S + Oxidation (M)
22567	240 - 262	794.3933	2380.1580	2380.1547	1.39	1	71	2.3e-07	1	U	K.SKFQEVPETGVSFADVAGADQAK.L
22572	240 - 262	794.7261	2381.1566	2381.1387	7.51	1	40	0.00018	1	U	K.SKFQEVPETGVSFADVAGADQAK.L + Deamidated (NQ)
20855	242 - 262	722.6842	2165.0309	2165.0277	1.48	0	58	3.4e-06	1	U	K.FQEVPETGVSFADVAGADQAK.L
20856	242 - 262	1083.5237	2165.0329	2165.0277	2.39	0	80	2.9e-08	1	U	K.FQEVPETGVSFADVAGADQAK.L
20857	242 - 262	722.6854	2165.0345	2165.0277	3.12	0	50	2.1e-05	1	U	K.FQEVPETGVSFADVAGADQAK.L
20860	242 - 262	1084.0220	2166.0294	2166.0117	8.17	0	70	2.5e-07	1	U	K.FQEVPETGVSFADVAGADQAK.L + Deamidated (NQ)
10122	263 - 273	666.8745	1331.7344	1331.7337	0.57	0	75	7.9e-07	1	U	K.LELQEVVDFLK.N
17268	263 - 277	596.3251	1785.9534	1785.9512	1.18	1	66	6.9e-07	1	U	K.LELQEVVDFLKNPDK.Y
23062	263 - 284	623.8430	2491.3430	2491.3210	8.83	2	53	1.2e-05	1	U	K.LELQEVVDFLKNPDKYTALGAK.I + Deamidated (NQ)
7021	274 - 284	393.2118	1176.6136	1176.6139	-0.24	1	21	0.011	1	U	K.NPDKYTALGAK.I
7023	274 - 284	589.3142	1176.6139	1176.6139	0.060	1	45	7.8e-05	1	U	K.NPDKYTALGAK.I
7024	274 - 284	393.2120	1176.6141	1176.6139	0.18	1	37	0.00035	1	U	K.NPDKYTALGAK.I
7025	274 - 284	589.3146	1176.6146	1176.6139	0.59	1	50	1.9e-05	1	U	K.NPDKYTALGAK.I
6572	288 - 299	578.3146	1154.6147	1154.6118	2.52	0	29	0.0024	1	U	K.GCLLVGPPGTGK.T
2444	334 - 340	453.7556	905.4967	905.4971	-0.43	1	13	0.7	1	U	R.VRDLFEK.A
2445	334 - 340	453.7558	905.4971	905.4971	0.085	1	15	0.77	1	U	R.VRDLFEK.A
18673	343 - 359	630.6651	1888.9734	1888.9717	0.90	1	66	7.5e-07	1	U	K.SKAPCIVFIDEIDAVGR.Q
9714	417 - 428	656.8502	1311.6859	1311.6895	-2.76	0	28	0.0029	1	U	R.QVTVDRPDVAGR.V
9715	417 - 428	656.8517	1311.6889	1311.6895	-0.46	0	34	0.003	1	U	R.QVTVDRPDVAGR.V
9716	417 - 428	438.2373	1311.6900	1311.6895	0.32	0	26	0.0038	1	U	R.QVTVDRPDVAGR.V
5066	429 - 437	540.3374	1078.6603	1078.6611	-0.78	1	24	0.014	1	U	R.VKILQVHSR.G
5067	429 - 437	360.5609	1078.6608	1078.6611	-0.31	1	15	0.045	1	U	R.VKILQVHSR.G
1682	431 - 437	426.7555	851.4965	851.4977	-1.39	0	23	0.0072	1	U	K.ILQVHSR.G
1683	431 - 437	426.7558	851.4971	851.4977	-0.73	0	31	0.0014	1	U	K.ILQVHSR.G
5911	440 - 449	374.5332	1120.5779	1120.5764	1.30	1	10	0.11	1	U	K.ALAKDVDFDK.V
5912	440 - 449	561.2971	1120.5796	1120.5764	2.83	1	25	0.0048	1	U	K.ALAKDVDFDK.V
4745	444 - 452	532.7724	1063.5302	1063.5298	0.36	1	29	0.0033	1	U	K.DVDFDKVAR.R
4747	444 - 452	355.5176	1063.5309	1063.5298	1.04	1	26	0.0033	1	U	K.DVDFDKVAR.R
22798	453 - 475	810.7572	2429.2497	2429.2485	0.49	1	90	4.8e-09	1	U	R.RTPGFTGADLQNLMNEAAILAAR.R
22855	453 - 475	816.0932	2445.2578	2445.2434	5.90	1	62	1.5e-06	1	U	R.RTPGFTGADLQNLMNEAAILAAR.R + Oxidation (M)
21707	454 - 475	1137.5812	2273.1479	2273.1474	0.25	0	165	3.2e-16	1	U	R.TPGFTGADLQNLMNEAAILAAR.R
21708	454 - 475	758.7239	2273.1498	2273.1474	1.05	0	91	3e-09	1	U	R.TPGFTGADLQNLMNEAAILAAR.R
21870	454 - 475	764.0547	2289.1424	2289.1423	0.022	0	78	4.6e-08	1	U	R.TPGFTGADLQNLMNEAAILAAR.R + Oxidation (M)
18453	477 - 492	937.9879	1873.9612	1873.9632	-1.10	2	96	1.4e-09	1	U	R.ELKEISKDEISDALER.I
18455	477 - 492	625.6618	1873.9635	1873.9632	0.14	2	92	2.2e-09	1	U	R.ELKEISKDEISDALER.I
18456	477 - 492	469.4991	1873.9673	1873.9632	2.16	2	14	0.32	1	U	R.ELKEISKDEISDALER.I
13498	480 - 492	502.2551	1503.7435	1503.7416	1.22	1	58	3e-05	1	U	K.EISKDEISDALER.I
13499	480 - 492	502.2554	1503.7442	1503.7416	1.73	1	45	0.0004	1	U	K.EISKDEISDALER.I
4385	484 - 492	524.2533	1046.4920	1046.4880	3.82	0	3	0.55	1	U	K.DEISDALER.I
215	493 - 499	364.2207	726.4268	726.4276	-1.04	0	1	0.87	1	U	R.IIAGPEK.K
216	493 - 499	364.2211	726.4276	726.4276	-0.0069	0	27	0.0044	1	U	R.IIAGPEK.K
1718	493 - 500	428.2686	854.5227	854.5225	0.22	1	13	0.055	1	U	R.IIAGPEKK.N
23261	511 - 534	638.5806	2550.2934	2550.2941	-0.25	0	3	0.51	1	U	R.LVAYHEAGHALVGALMPEYDPVAK.I
23262	511 - 534	638.5811	2550.2954	2550.2941	0.53	0	12	0.076	1	U	R.LVAYHEAGHALVGALMPEYDPVAK.I
23263	511 - 534	851.1059	2550.2959	2550.2941	0.74	0	56	5.5e-06	1	U	R.LVAYHEAGHALVGALMPEYDPVAK.I
23264	511 - 534	851.1060	2550.2962	2550.2941	0.82	0	47	4e-05	1	U	R.LVAYHEAGHALVGALMPEYDPVAK.I
23265	511 - 534	638.5816	2550.2972	2550.2941	1.22	0	44	7.6e-05	1	U	R.LVAYHEAGHALVGALMPEYDPVAK.I
23266	511 - 534	638.5819	2550.2986	2550.2941	1.77	0	27	0.0029	1	U	R.LVAYHEAGHALVGALMPEYDPVAK.I
23267	511 - 534	638.5822	2550.2996	2550.2941	2.16	0	11	0.087	1	U	R.LVAYHEAGHALVGALMPEYDPVAK.I
23268	511 - 534	851.1074	2550.3004	2550.2941	2.50	0	48	3.2e-05	1	U	R.LVAYHEAGHALVGALMPEYDPVAK.I
23270	511 - 534	638.5832	2550.3037	2550.2941	3.77	0	7	0.2	1	U	R.LVAYHEAGHALVGALMPEYDPVAK.I
23271	511 - 534	638.5837	2550.3056	2550.2941	4.53	0	14	0.052	1	U	R.LVAYHEAGHALVGALMPEYDPVAK.I
23344	511 - 534	642.5797	2566.2898	2566.2890	0.31	0	50	1.9e-05	1	U	R.LVAYHEAGHALVGALMPEYDPVAK.I + Oxidation (M)
23345	511 - 534	856.4381	2566.2924	2566.2890	1.34	0	62	1.7e-06	1	U	R.LVAYHEAGHALVGALMPEYDPVAK.I + Oxidation (M)

23346	511 – 534	642.5807	2566.2938	2566.2890	1.87	0	46	4.7e-05	1	U	R.LVAYHEAGHALVGALMPEYDPVAK.I + Oxidation (M)
14471	541 – 555	783.8821	1565.7496	1565.7474	1.41	0	69	3.5e-07	1	U	R.GQAGGLTFFAPSEER.L
14472	541 – 555	783.8826	1565.7507	1565.7474	2.09	0	77	6.4e-08	1	U	R.GQAGGLTFFAPSEER.L
14473	541 – 555	522.9243	1565.7509	1565.7474	2.25	0	27	0.0032	1	U	R.GQAGGLTFFAPSEER.L
22959	541 – 563	824.7415	2471.2025	2471.2081	-2.24	1	13	0.055	1	U	R.GQAGGLTFFAPSEERLESGLYSR.S
22960	541 – 563	824.7430	2471.2071	2471.2081	-0.40	1	32	0.00094	1	U	R.GQAGGLTFFAPSEERLESGLYSR.S
2664	556 – 563	462.7430	923.4715	923.4712	0.28	0	7	0.23	1	U	R.LESGLYSR.S
2665	556 – 563	462.7431	923.4717	923.4712	0.50	0	24	0.016	1	U	R.LESGLYSR.S
13558	564 – 577	754.8805	1507.7464	1507.7453	0.73	0	79	3.7e-08	1	U	R.SYLENQMAVALGGR.V
13560	564 – 577	503.5895	1507.7466	1507.7453	0.90	0	42	0.00061	1	U	R.SYLENQMAVALGGR.V
13561	564 – 577	754.8807	1507.7468	1507.7453	1.02	0	84	1.6e-08	1	U	R.SYLENQMAVALGGR.V
13562	564 – 577	503.5899	1507.7479	1507.7453	1.74	0	36	0.00084	1	U	R.SYLENQMAVALGGR.V
13572	564 – 577	755.3746	1508.7347	1508.7293	3.59	0	30	0.0015	1	U	R.SYLENQMAVALGGR.V + Deamidated (NQ)
13824	564 – 577	762.8780	1523.7415	1523.7402	0.86	0	79	4.2e-08	1	U	R.SYLENQMAVALGGR.V + Oxidation (M)
13825	564 – 577	762.8783	1523.7420	1523.7402	1.19	0	72	1.9e-07	1	U	R.SYLENQMAVALGGR.V + Oxidation (M)
13826	564 – 577	508.9216	1523.7429	1523.7402	1.79	0	17	0.028	1	U	R.SYLENQMAVALGGR.V + Oxidation (M)
13834	564 – 577	763.3690	1524.7235	1524.7242	-0.48	0	43	9.3e-05	1	U	R.SYLENQMAVALGGR.V + Deamidated (NQ); Oxidation (M)
23773	578 – 602	1344.6335	2687.2525	2687.2385	5.23	0	90	3.8e-09	1	U	R.VAEEVIFGENNVTTGASSDFMQVSR.V + Deamidated (NQ)
23774	578 – 602	896.7582	2687.2527	2687.2385	5.30	0	53	1.1e-05	1	U	R.VAEEVIFGENNVTTGASSDFMQVSR.V + Deamidated (NQ)
23775	578 – 602	896.7603	2687.2589	2687.2385	7.61	0	100	4.7e-10	1	U	R.VAEEVIFGENNVTTGASSDFMQVSR.V + Deamidated (NQ)
23807	578 – 602	902.0884	2703.2433	2703.2334	3.67	0	12	0.069	1	U	R.VAEEVIFGENNVTTGASSDFMQVSR.V + Deamidated (NQ); Oxidation (M)
22099	616 – 639	772.7441	2315.2105	2315.2056	2.14	1	39	0.00022	1	U	K.KIGQLAVGGAGGNPFLGQQMSSAK.D
22100	616 – 639	772.7455	2315.2145	2315.2056	3.86	1	35	0.00057	1	U	K.KIGQLAVGGAGGNPFLGQQMSSAK.D
22242	616 – 639	778.4084	2332.2033	2332.1845	8.06	1	27	0.0029	1	U	K.KIGQLAVGGAGGNPFLGQQMSSAK.D + Deamidated (NQ); Oxidation (M)
21004	617 – 639	1094.5619	2187.1092	2187.1106	-0.68	0	86	9.5e-09	1	U	K.IGQLAVGGAGGNPFLGQQMSSAK.D
21005	617 – 639	1094.5623	2187.1100	2187.1106	-0.31	0	59	2.8e-06	1	U	K.IGQLAVGGAGGNPFLGQQMSSAK.D
21006	617 – 639	730.0440	2187.1102	2187.1106	-0.22	0	49	2.8e-05	1	U	K.IGQLAVGGAGGNPFLGQQMSSAK.D
21094	617 – 639	735.3747	2203.1023	2203.1056	-1.50	0	11	0.1	1	U	K.IGQLAVGGAGGNPFLGQQMSSAK.D + Oxidation (M)
21099	617 – 639	1103.0612	2204.1078	2204.0896	8.25	0	44	7.9e-05	1	U	K.IGQLAVGGAGGNPFLGQQMSSAK.D + Deamidated (NQ); Oxidation (M)
15512	640 – 654	821.3731	1640.7317	1640.7352	-2.15	0	93	2e-09	1	U	K.DYSMATADIVDGEVR.E
15513	640 – 654	821.3748	1640.7351	1640.7352	-0.051	0	111	4.2e-11	1	U	K.DYSMATADIVDGEVR.E
15514	640 – 654	547.9195	1640.7366	1640.7352	0.84	0	48	3.3e-05	1	U	K.DYSMATADIVDGEVR.E
15515	640 – 654	821.3758	1640.7370	1640.7352	1.12	0	97	8.6e-10	1	U	K.DYSMATADIVDGEVR.E
15718	640 – 654	829.3719	1656.7292	1656.7301	-0.53	0	106	1.2e-10	1	U	K.DYSMATADIVDGEVR.E + Oxidation (M)
15719	640 – 654	553.2506	1656.7300	1656.7301	-0.083	0	19	0.018	1	U	K.DYSMATADIVDGEVR.E + Oxidation (M)
15720	640 – 654	829.3733	1656.7320	1656.7301	1.12	0	110	4.4e-11	1	U	K.DYSMATADIVDGEVR.E + Oxidation (M)
3014	655 – 662	475.7630	949.5114	949.5120	-0.67	0	36	0.0039	1	U	R.ELVEVAYK.R
3015	655 – 662	475.7637	949.5129	949.5120	0.89	0	34	0.0065	1	U	R.ELVEVAYK.R
5639	655 – 663	553.8143	1105.6140	1105.6131	0.76	1	43	0.00011	1	U	R.ELVEVAYKR.A
5640	655 – 663	369.5453	1105.6142	1105.6131	0.93	1	20	0.014	1	U	R.ELVEVAYKR.A
15185	664 – 677	405.2298	1616.8903	1616.8886	1.03	0	34	0.00061	1	U	R.ATEIINTHIDILHK.L
15186	664 – 677	539.9708	1616.8906	1616.8886	1.24	0	54	7.9e-06	1	U	R.ATEIINTHIDILHK.L
15187	664 – 677	539.9709	1616.8908	1616.8886	1.37	0	32	0.00095	1	U	R.ATEIINTHIDILHK.L
15188	664 – 677	809.4530	1616.8914	1616.8886	1.73	0	37	0.00032	1	U	R.ATEIINTHIDILHK.L
2689	678 – 685	464.2979	926.5812	926.5800	1.26	0	44	0.00029	1	U	K.LAQLLIEK.E
2690	678 – 685	464.2979	926.5812	926.5800	1.29	0	43	0.00038	1	U	K.LAQLLIEK.E
2710	678 – 685	464.7905	927.5664	927.5640	2.59	0	21	0.15	1	U	K.LAQLLIEK.E + Deamidated (NQ)

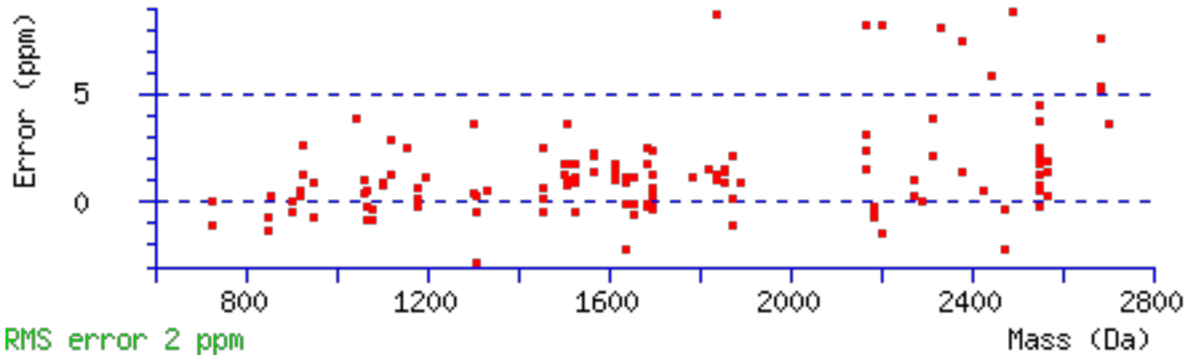

Mascot: http://www.matrixscience.com/

MATRIX SCIENCE MASCOT Search Results

Protein View: tr|A0A0K9Q765|A0A0K9Q765_SPIOL

>tr|A0A0K9Q765|A0A0K9Q765_SPIOL ATPase_AAA_core domain-containing protein OS=Spinacia oleracea OX=3562 GN=SOVF_213350 PE=4 SV=1

**Database:**	Uni-Spinach
**Score:**	4065
**Nominal mass (M_r):**	47740
**Calculated pI:**	6.97

Sequence similarity is available as **an NCBI BLAST search of tr|A0A0K9Q765|A0A0K9Q765_SPIOL against nr**.

Search parameters

**MS data file:**	\\172.16.0.213\tank\windowsVM\Bill Cramer\052418\MGF\wc_QE_052418_Cramer_gel_4.mgf
**Enzyme:**	Trypsin: cuts C-term side of KR unless next residue is P.
**Fixed modifications:**	Carbamidomethyl (C)
**Variable modifications:**	Acetyl (K), Acetyl (Protein N-term), Deamidated (NQ), Oxidation (M)

Protein sequence coverage: 78%

Matched peptides shown in ***bold red***.

```
  1 MATAVSTVGA ATRAPLNLNG SSAGASVPTS GFLGSSLKKH TNVRFPSSSR
 51 TTSMTVKAAE NEEKNTDKWA HLAKDFSDDQ LDIRRGKGMV DSLFQAPADA
101 GTHVPIQSSF EYESQGLRKY DIDNMLGDFY IAPAFMDKLV VHITKNFLNL
151 PNIKIPLILG VWGGKGQGKS FQCELVFAKL GINPIMMSAG ELESGNAGEP
201 AKLIRQRYRE AADLIAKGKM CALFINDLDA GAGRMGGTTQ YTVNNQMVNA
251 TLMNIADNPT NVQLPGMYNK QDNARVPIIV TGNDFSTLYA PLIRDGRMEK
301 FYWAPTREDR IGVCTGIFKT DKVPAEHVVK LVDAFPGQSI DFFGALRARV
351 YDDEVRKWVN SVGVDNVGKK LVNSKDGPPV FEQPEMTLQK LMEYGNMLVQ
401 EQENVKRVQL ADQYMSSAAL GDANKDAIDR GTFFG
```

Unformatted sequence string: **435 residues** (for pasting into other applications).

Sort peptides by ◉ Residue Number ○ Increasing Mass ○ Decreasing Mass

Show predicted peptides also

Query	Start – End	Observed	Mr(expt)	Mr(calc)	ppm	M	Score	Expect	Rank	U	Peptide
19372	58 – 74	489.4971	1953.9591	1953.9544	2.43	2	11	0.085	1	U	K.AAENEEKNTDKWAHLAK.D
7138	65 – 74	395.2115	1182.6128	1182.6145	-1.45	1	26	0.0036	1	U	K.NTDKWAHLAK.D
7139	65 – 74	395.2121	1182.6146	1182.6145	0.036	1	2	0.7	1	U	K.NTDKWAHLAK.D
7140	65 – 74	592.3147	1182.6148	1182.6145	0.20	1	35	0.0018	1	U	K.NTDKWAHLAK.D
202	69 – 74	363.2077	724.4009	724.4020	-1.56	0	4	0.39	1	U	K.WAHLAK.D
8027	75 – 84	612.2810	1222.5475	1222.5466	0.72	0	41	0.00015	1	U	K.DFSDDQLDIR.R
8028	75 – 84	612.2816	1222.5486	1222.5466	1.63	0	53	1.1e-05	1	U	K.DFSDDQLDIR.R
10996	75 – 85	690.3308	1378.6471	1378.6477	-0.46	1	12	0.082	1	U	K.DFSDDQLDIRR.G
10997	75 – 85	690.3312	1378.6478	1378.6477	0.047	1	27	0.0077	1	U	K.DFSDDQLDIRR.G
10998	75 – 85	460.5570	1378.6493	1378.6477	1.17	1	19	0.018	1	U	K.DFSDDQLDIRR.G
10999	75 – 85	460.5575	1378.6506	1378.6477	2.10	1	20	0.014	1	U	K.DFSDDQLDIRR.G
24741	86 – 118	881.6796	3522.6894	3522.6725	4.78	1	50	2.2e-05	1	U	R.GKGMVDSLFQAPADAGTHVPIQSSFEYESQGLR.K + Deamidated (NQ)
24750	86 – 118	885.4295	3537.6889	3537.6835	1.54	1	51	1.5e-05	1	U	R.GKGMVDSLFQAPADAGTHVPIQSSFEYESQGLR.K + Oxidation (M)
24814	86 – 119	917.9506	3667.7734	3667.7464	7.35	2	19	0.018	1	U	R.GKGMVDSLFQAPADAGTHVPIQSSFEYESQGLRK.Y + 2 Deamidated (NQ); Oxidation (M)
24594	88 – 118	835.1523	3336.5800	3336.5721	2.37	0	44	6.8e-05	1	U	K.GMVDSLFQAPADAGTHVPIQSSFEYESQGLR.K
24625	88 – 118	1118.5316	3352.5729	3352.5670	1.74	0	50	2.2e-05	1	U	K.GMVDSLFQAPADAGTHVPIQSSFEYESQGLR.K + Oxidation (M)
24720	88 – 119	867.4249	3465.6706	3465.6511	5.63	1	14	0.053	1	U	K.GMVDSLFQAPADAGTHVPIQSSFEYESQGLRK.Y + Deamidated (NQ)
24721	88 – 119	867.4265	3465.6767	3465.6511	7.40	1	23	0.0068	1	U	K.GMVDSLFQAPADAGTHVPIQSSFEYESQGLRK.Y + Deamidated (NQ)
24727	88 – 119	871.4244	3481.6684	3481.6460	6.44	1	16	0.035	1	U	K.GMVDSLFQAPADAGTHVPIQSSFEYESQGLRK.Y + Deamidated (NQ); Oxidation (M)
22511	119 – 138	789.7066	2366.0979	2366.0963	0.68	1	58	3.5e-06	1	U	R.KYDIDNMLGDFYIAPAFMDK.L
22576	119 – 138	795.0371	2382.0896	2382.0912	-0.67	1	48	3.3e-05	1	U	R.KYDIDNMLGDFYIAPAFMDK.L + Oxidation (M)
22577	119 – 138	795.0385	2382.0938	2382.0912	1.09	1	36	0.00038	1	U	R.KYDIDNMLGDFYIAPAFMDK.L + Oxidation (M)
22646	119 – 138	800.3716	2398.0930	2398.0861	2.89	1	35	0.00057	1	U	R.KYDIDNMLGDFYIAPAFMDK.L + 2 Oxidation (M)
22656	119 – 138	800.7043	2399.0912	2399.0701	8.79	1	15	0.035	1	U	R.KYDIDNMLGDFYIAPAFMDK.L + Deamidated (NQ); 2 Oxidation (M)
21547	120 – 138	752.3392	2253.9959	2253.9962	-0.14	0	16	0.03	1	U	K.YDIDNMLGDFYIAPAFMDK.L + Oxidation (M)
21548	120 – 138	1128.0053	2253.9960	2253.9962	-0.10	0	73	1.5e-07	1	U	K.YDIDNMLGDFYIAPAFMDK.L + Oxidation (M)
1076	139 – 145	405.2657	808.5168	808.5171	-0.34	0	7	0.18	1	U	K.LVVHITK.N
1077	139 – 145	405.2659	808.5173	808.5171	0.22	0	8	0.15	1	U	K.LVVHITK.N
4920	146 – 154	536.8109	1071.6073	1071.6077	-0.34	0	22	0.04		U	K.NFLNLPNIK.I

									1		
4922	146 – 154	536.8116	1071.6087	1071.6077	0.96	0	14	0.047	1	U	K.NFLNLPNIK.I
4950	146 – 154	537.3039	1072.5932	1072.5917	1.41	0	16	0.033	1	U	K.NFLNLPNIK.I + Deamidated (NQ)
4951	146 – 154	537.3042	1072.5938	1072.5917	2.02	0	17	0.025	1	U	K.NFLNLPNIK.I + Deamidated (NQ)
6523	155 – 165	576.8608	1151.7071	1151.7067	0.40	0	56	8.1e-06	1	U	K.IPLILGVWGGK.G
13792	155 – 169	508.3087	1521.9041	1521.9031	0.66	1	1	4	1	U	K.IPLILGVWGGKGQGK.S
8125	170 – 179	614.8062	1227.5978	1227.5958	1.67	0	25	0.0042	1	U	K.SFQCELVFAK.L
21838	180 – 202	1143.5602	2285.1058	2285.1031	1.18	0	111	4.1e-11	1	U	K.LGINPIMMSAGELESGNAGEPAK.L
21857	180 – 202	763.3694	2287.0863	2287.0711	6.64	0	20	0.013	1	U	K.LGINPIMMSAGELESGNAGEPAK.L + 2 Deamidated (NQ)
21963	180 – 202	1151.5531	2301.0917	2301.0980	-2.75	0	68	3.9e-07	1	U	K.LGINPIMMSAGELESGNAGEPAK.L + Oxidation (M)
21964	180 – 202	1151.5570	2301.0995	2301.0980	0.65	0	107	1e-10	1	U	K.LGINPIMMSAGELESGNAGEPAK.L + Oxidation (M)
21965	180 – 202	768.0412	2301.1019	2301.0980	1.67	0	84	1.3e-08	1	U	K.LGINPIMMSAGELESGNAGEPAK.L + Oxidation (M)
21966	180 – 202	1151.5584	2301.1023	2301.0980	1.83	0	122	4e-12	1	U	K.LGINPIMMSAGELESGNAGEPAK.L + Oxidation (M)
21974	180 – 202	1152.0486	2302.0826	2302.0820	0.25	0	15	0.036	1	U	K.LGINPIMMSAGELESGNAGEPAK.L + Deamidated (NQ); Oxidation (M)
21976	180 – 202	768.3717	2302.0933	2302.0820	4.88	0	15	0.039	1	U	K.LGINPIMMSAGELESGNAGEPAK.L + Deamidated (NQ); Oxidation (M)
22107	180 – 202	1159.5529	2317.0912	2317.0930	-0.78	0	96	1e-09	1	U	K.LGINPIMMSAGELESGNAGEPAK.L + 2 Oxidation (M)
22108	180 – 202	773.3715	2317.0927	2317.0930	-0.11	0	76	7.9e-08	1	U	K.LGINPIMMSAGELESGNAGEPAK.L + 2 Oxidation (M)
22115	180 – 202	773.7051	2318.0934	2318.0770	7.09	0	26	0.0039	1	U	K.LGINPIMMSAGELESGNAGEPAK.L + Deamidated (NQ); 2 Oxidation (M)
22116	180 – 202	1160.0548	2318.0951	2318.0770	7.81	0	78	5.2e-08	1	U	K.LGINPIMMSAGELESGNAGEPAK.L + Deamidated (NQ); 2 Oxidation (M)
22117	180 – 202	1160.0549	2318.0953	2318.0770	7.91	0	79	3.9e-08	1	U	K.LGINPIMMSAGELESGNAGEPAK.L + Deamidated (NQ); 2 Oxidation (M)
6455	208 – 217	575.3151	1148.6157	1148.6189	-2.81	1	21	0.25	1	U	R.YREAADLIAK.G
6456	208 – 217	383.8803	1148.6189	1148.6189	0.017	1	31	0.0061	1	U	R.YREAADLIAK.G
6457	208 – 217	383.8803	1148.6191	1148.6189	0.11	1	24	0.027	1	U	R.YREAADLIAK.G
6458	208 – 217	575.3168	1148.6191	1148.6189	0.12	1	24	0.15	1	U	R.YREAADLIAK.G
1321	210 – 217	415.7345	829.4545	829.4545	-0.031	0	7	0.2	1	U	R.EAADLIAK.G
24883	235 – 270	987.2173	3944.8400	3944.8053	8.81	0	69	3.7e-07	1	U	R.MGGTTQYTVNNQMVNATLMNIADNPTNVQLPGMYNK.Q + 2 Deamidated (NQ)
24884	235 – 270	1315.9551	3944.8434	3944.8053	9.67	0	72	1.9e-07	1	U	R.MGGTTQYTVNNQMVNATLMNIADNPTNVQLPGMYNK.Q + 2 Deamidated (NQ)
24887	235 – 270	991.2170	3960.8388	3960.8002	9.75	0	65	7.8e-07	1	U	R.MGGTTQYTVNNQMVNATLMNIADNPTNVQLPGMYNK.Q + 2 Deamidated (NQ); Oxidation (M)
24892	235 – 270	994.7147	3974.8298	3974.8271	0.68	0	39	0.00021	1	U	R.MGGTTQYTVNNQMVNATLMNIADNPTNVQLPGMYNK.Q + 2 Oxidation (M)
24893	235 – 270	994.7175	3974.8409	3974.8271	3.47	0	40	0.00018	1	U	R.MGGTTQYTVNNQMVNATLMNIADNPTNVQLPGMYNK.Q + 2 Oxidation (M)
24894	235 – 270	994.9651	3975.8315	3975.8111	5.13	0	46	4.5e-05	1	U	R.MGGTTQYTVNNQMVNATLMNIADNPTNVQLPGMYNK.Q + Deamidated (NQ); 2 Oxidation (M)
20342	276 – 294	697.0622	2088.1649	2088.1619	1.43	0	58	3.5e-06	1	U	R.VPIIVTGNDFSTLYAPLIR.D
20343	276 – 294	1045.0899	2088.1652	2088.1619	1.57	0	94	1.5e-09	1	U	R.VPIIVTGNDFSTLYAPLIR.D
20348	276 – 294	697.3895	2089.1467	2089.1459	0.37	0	53	1e-05	1	U	R.VPIIVTGNDFSTLYAPLIR.D + Deamidated (NQ)
2886	301 – 307	470.7377	939.4609	939.4603	0.68	0	29	0.0019	1	U	K.FYWAPTR.E
2887	301 – 307	470.7380	939.4615	939.4603	1.28	0	14	0.044	1	U	K.FYWAPTR.E
10226	301 – 310	670.8226	1339.6306	1339.6309	-0.21	1	21	0.018	1	U	K.FYWAPTREDR.I
10227	301 – 310	447.5509	1339.6309	1339.6309	-0.0067	1	9	0.16	1	U	K.FYWAPTREDR.I
10228	301 – 310	447.5509	1339.6310	1339.6309	0.040	1	6	0.25	1	U	K.FYWAPTREDR.I
10230	301 – 310	670.8240	1339.6335	1339.6309	1.90	1	13	0.055	1	U	K.FYWAPTREDR.I
3597	311 – 319	497.7733	993.5321	993.5318	0.31	0	33	0.00076	1	U	R.IGVCTGIFK.T
3598	311 – 319	497.7744	993.5343	993.5318	2.56	0	19	0.033	1	U	R.IGVCTGIFK.T
8018	320 – 330	408.2306	1221.6699	1221.6717	-1.54	1	14	0.092	1	U	K.TDKVPAEHVVK.L
8019	320 – 330	611.8424	1221.6702	1221.6717	-1.25	1	25	0.0065	1	U	K.TDKVPAEHVVK.L
8020	320 – 330	408.2308	1221.6706	1221.6717	-0.92	1	39	0.00068	1	U	K.TDKVPAEHVVK.L
8021	320 – 330	408.2311	1221.6716	1221.6717	-0.14	1	18	0.056	1	U	K.TDKVPAEHVVK.L
8022	320 – 330	611.8434	1221.6723	1221.6717	0.45	1	6	0.43	1	U	K.TDKVPAEHVVK.L
2084	323 – 330	439.7580	877.5014	877.5022	-0.90	0	39	0.00022	1	U	K.VPAEHVVK.L
18172	331 – 347	926.9837	1851.9528	1851.9519	0.47	0	61	7.7e-06	1	U	K.LVDAFPGQSIDFFGALR.A
18173	331 – 347	618.3252	1851.9536	1851.9519	0.90	0	66	1.3e-06	1	U	K.LVDAFPGQSIDFFGALR.A
2245	350 – 356	448.2102	894.4059	894.4083	-2.73	0	24	0.0058	1	U	R.VYDDEVR.K
4007	350 – 357	512.2587	1022.5028	1022.5033	-0.49	1	20	0.013	1	U	R.VYDDEVRK.W
4008	350 – 357	512.2590	1022.5035	1022.5033	0.26	1	23	0.0074	1	U	R.VYDDEVRK.W
11444	357 – 369	467.9185	1400.7336	1400.7412	-5.42	1	18	0.022	1	U	R.KWVNSVGVDNVGK.K
11450	357 – 369	701.3785	1400.7424	1400.7412	0.84	1	65	8e-07	1	U	R.KWVNSVGVDNVGK.K
13903	357 – 370	765.4228	1528.8310	1528.8362	-3.37	2	28	0.0026	1	U	R.KWVNSVGVDNVGKK.L
13904	357 – 370	383.2161	1528.8351	1528.8362	-0.70	2	35	0.00056	1	U	R.KWVNSVGVDNVGKK.L
8943	358 – 369	637.3307	1272.6468	1272.6463	0.42	0	52	2.8e-05	1	U	K.WVNSVGVDNVGK.K
8944	358 – 369	637.3309	1272.6473	1272.6463	0.80	0	54	1.6e-05	1	U	K.WVNSVGVDNVGK.K
8945	358 – 369	637.3316	1272.6487	1272.6463	1.91	0	43	0.00017	1	U	K.WVNSVGVDNVGK.K
8946	358 – 369	637.3328	1272.6510	1272.6463	3.70	0	36	0.00041	1	U	K.WVNSVGVDNVGK.K
8958	358 – 369	637.8239	1273.6332	1273.6303	2.27	0	37	0.0087	1	U	K.WVNSVGVDNVGK.K + Deamidated (NQ)
11445	358 – 370	701.3757	1400.7369	1400.7412	-3.08	1	28	0.0024	1	U	K.WVNSVGVDNVGKK.L
11447	358 – 370	467.9211	1400.7416	1400.7412	0.24	1	61	2e-06	1	U	K.WVNSVGVDNVGKK.L
11449	358 – 370	701.3783	1400.7421	1400.7412	0.64	1	46	5.4e-05	1	U	K.WVNSVGVDNVGKK.L

11451	358 – 370	467.9218	1400.7436	1400.7412	1.72	1	53	1.2e-05	1	U	K.WVNSVGVDNVGKK.L
11462	358 – 370	468.2455	1401.7146	1401.7252	-7.57	1	18	0.028	1	U	K.WVNSVGVDNVGKK.L + Deamidated (NQ)
11465	358 – 370	468.2472	1401.7198	1401.7252	-3.89	1	21	0.01	1	U	K.WVNSVGVDNVGKK.L + Deamidated (NQ)
11466	358 – 370	701.8709	1401.7273	1401.7252	1.45	1	31	0.0013	1	U	K.WVNSVGVDNVGKK.L + Deamidated (NQ)
11467	358 – 370	701.8720	1401.7295	1401.7252	3.02	1	46	5.4e-05	1	U	K.WVNSVGVDNVGKK.L + Deamidated (NQ)
21580	371 – 390	753.7183	2258.1331	2258.1140	8.46	1	0	0.95	1	U	K.LVNSKDGPPVFEQPEMTLQK.L + 2 Deamidated (NQ)
16427	376 – 390	858.4150	1714.8154	1714.8236	-4.79	0	12	0.081	1	U	K.DGPPVFEQPEMTLQK.L
16428	376 – 390	858.4174	1714.8203	1714.8236	-1.93	0	47	3.8e-05	1	U	K.DGPPVFEQPEMTLQK.L
16429	376 – 390	572.6149	1714.8229	1714.8236	-0.43	0	32	0.00093	1	U	K.DGPPVFEQPEMTLQK.L
16446	376 – 390	858.9192	1715.8238	1715.8076	9.44	0	21	0.01	1	U	K.DGPPVFEQPEMTLQK.L + Deamidated (NQ)
16619	376 – 390	866.4168	1730.8191	1730.8185	0.35	0	43	0.0001	1	U	K.DGPPVFEQPEMTLQK.L + Oxidation (M)
16620	376 – 390	866.4174	1730.8202	1730.8185	0.96	0	44	8.3e-05	1	U	K.DGPPVFEQPEMTLQK.L + Oxidation (M)
16622	376 – 390	866.4176	1730.8206	1730.8185	1.16	0	44	7e-05	1	U	K.DGPPVFEQPEMTLQK.L + Oxidation (M)
16623	376 – 390	577.9475	1730.8206	1730.8185	1.17	0	29	0.002	1	U	K.DGPPVFEQPEMTLQK.L + Oxidation (M)
16624	376 – 390	577.9490	1730.8251	1730.8185	3.79	0	15	0.041	1	U	K.DGPPVFEQPEMTLQK.L + Oxidation (M)
19084	391 – 406	963.4611	1924.9076	1924.8910	8.60	0	44	7.5e-05	1	U	K.LMEYGNMLVQEQENVK.R + Deamidated (NQ)
19215	391 – 406	647.6406	1939.9000	1939.9019	-0.96	0	12	0.075	1	U	K.LMEYGNMLVQEQENVK.R + Oxidation (M)
19216	391 – 406	647.6408	1939.9006	1939.9019	-0.66	0	10	0.11	1	U	K.LMEYGNMLVQEQENVK.R + Oxidation (M)
19218	391 – 406	970.9587	1939.9028	1939.9019	0.46	0	62	1.4e-06	1	U	K.LMEYGNMLVQEQENVK.R + Oxidation (M)
19219	391 – 406	970.9597	1939.9048	1939.9019	1.47	0	71	2e-07	1	U	K.LMEYGNMLVQEQENVK.R + Oxidation (M)
19220	391 – 406	647.6429	1939.9070	1939.9019	2.61	0	10	0.13	1	U	K.LMEYGNMLVQEQENVK.R + Oxidation (M)
19406	391 – 406	652.9728	1955.8967	1955.8968	-0.082	0	32	0.00096	1	U	K.LMEYGNMLVQEQENVK.R + 2 Oxidation (M)
19426	391 – 406	979.4557	1956.8968	1956.8808	8.17	0	71	2.4e-07	1	U	K.LMEYGNMLVQEQENVK.R + Deamidated (NQ); 2 Oxidation (M)
20290	391 – 407	694.3436	2080.0089	2080.0081	0.37	1	55	6.9e-06	1	U	K.LMEYGNMLVQEQENVKR.V
20291	391 – 407	694.3443	2080.0110	2080.0081	1.41	1	41	0.00015	1	U	K.LMEYGNMLVQEQENVKR.V
20297	391 – 407	694.6725	2080.9956	2080.9921	1.69	1	28	0.0026	1	U	K.LMEYGNMLVQEQENVKR.V + Deamidated (NQ)
20311	391 – 407	695.0052	2081.9937	2081.9761	8.46	1	16	0.031	1	U	K.LMEYGNMLVQEQENVKR.V + 2 Deamidated (NQ)
20385	391 – 407	699.6753	2096.0042	2096.0030	0.54	1	71	2.4e-07	1	U	K.LMEYGNMLVQEQENVKR.V + Oxidation (M)
20386	391 – 407	699.6755	2096.0046	2096.0030	0.75	1	77	5.5e-08	1	U	K.LMEYGNMLVQEQENVKR.V + Oxidation (M)
20387	391 – 407	699.6757	2096.0053	2096.0030	1.09	1	64	1e-06	1	U	K.LMEYGNMLVQEQENVKR.V + Oxidation (M)
20388	391 – 407	699.6761	2096.0064	2096.0030	1.63	1	22	0.0085	1	U	K.LMEYGNMLVQEQENVKR.V + Oxidation (M)
20389	391 – 407	699.6768	2096.0084	2096.0030	2.59	1	17	0.026	1	U	K.LMEYGNMLVQEQENVKR.V + Oxidation (M)
20402	391 – 407	700.0045	2096.9917	2096.9870	2.24	1	5	0.6	1	U	K.LMEYGNMLVQEQENVKR.V + Deamidated (NQ); Oxidation (M)
20403	391 – 407	700.0065	2096.9978	2096.9870	5.12	1	25	0.0075	1	U	K.LMEYGNMLVQEQENVKR.V + Deamidated (NQ); Oxidation (M)
20404	391 – 407	1049.5085	2097.0025	2096.9870	7.40	1	66	6.8e-07	1	U	K.LMEYGNMLVQEQENVKR.V + Deamidated (NQ); Oxidation (M)
20405	391 – 407	700.0089	2097.0049	2096.9870	8.52	1	27	0.0029	1	U	K.LMEYGNMLVQEQENVKR.V + Deamidated (NQ); Oxidation (M)
20406	391 – 407	1049.5099	2097.0052	2096.9870	8.68	1	74	1.2e-07	1	U	K.LMEYGNMLVQEQENVKR.V + Deamidated (NQ); Oxidation (M)
20514	391 – 407	1057.0049	2111.9952	2111.9979	-1.29	1	108	7.4e-11	1	U	K.LMEYGNMLVQEQENVKR.V + 2 Oxidation (M)
20515	391 – 407	705.0062	2111.9969	2111.9979	-0.49	1	46	4.9e-05	1	U	K.LMEYGNMLVQEQENVKR.V + 2 Oxidation (M)
20516	391 – 407	705.0071	2111.9996	2111.9979	0.78	1	52	1.2e-05	1	U	K.LMEYGNMLVQEQENVKR.V + 2 Oxidation (M)
20527	391 – 407	705.3357	2112.9853	2112.9819	1.56	1	0	0.91	1	U	K.LMEYGNMLVQEQENVKR.V + Deamidated (NQ); 2 Oxidation (M)
20545	391 – 407	705.6693	2113.9861	2113.9660	9.53	1	17	0.023	1	U	K.LMEYGNMLVQEQENVKR.V + 2 Deamidated (NQ); 2 Oxidation (M)
19979	407 – 425	680.0057	2036.9954	2036.9949	0.24	1	21	0.0099	1	U	K.RVQLADQYMSSAALGDANK.D
23503	407 – 430	870.0993	2607.2761	2607.2711	1.92	2	93	2e-09	1	U	K.RVQLADQYMSSAALGDANKDAIDR.G
23512	407 – 430	653.0770	2608.2790	2608.2551	9.17	2	9	0.15	1	U	K.RVQLADQYMSSAALGDANKDAIDR.G + Deamidated (NQ)
23518	407 – 430	653.3231	2609.2634	2609.2391	9.31	2	19	0.016	1	U	K.RVQLADQYMSSAALGDANKDAIDR.G + 2 Deamidated (NQ)
23585	407 – 430	656.8242	2623.2676	2623.2660	0.63	2	43	9.7e-05	1	U	K.RVQLADQYMSSAALGDANKDAIDR.G + Oxidation (M)
23586	407 – 430	875.7592	2624.2558	2624.2500	2.22	2	32	0.0011	1	U	K.RVQLADQYMSSAALGDANKDAIDR.G + Deamidated (NQ); Oxidation (M)
18542	408 – 425	941.4527	1880.8908	1880.8938	-1.57	0	125	2.5e-12	1	U	R.VQLADQYMSSAALGDANK.D
18544	408 – 425	941.4543	1880.8940	1880.8938	0.086	0	128	1.4e-12	1	U	R.VQLADQYMSSAALGDANK.D
18545	408 – 425	627.9722	1880.8946	1880.8938	0.44	0	73	1.5e-07	1	U	R.VQLADQYMSSAALGDANK.D
18753	408 – 425	949.4510	1896.8875	1896.8887	-0.65	0	60	2.4e-06	1	U	R.VQLADQYMSSAALGDANK.D + Oxidation (M)
18754	408 – 425	949.4541	1896.8936	1896.8887	2.59	0	111	3.9e-11	1	U	R.VQLADQYMSSAALGDANK.D + Oxidation (M)
22882	408 – 430	818.0628	2451.1666	2451.1700	-1.38	1	70	2.6e-07	1	U	R.VQLADQYMSSAALGDANKDAIDR.G
22883	408 – 430	818.0647	2451.1723	2451.1700	0.95	1	98	6.6e-10	1	U	R.VQLADQYMSSAALGDANKDAIDR.G
22884	408 – 430	613.8005	2451.1729	2451.1700	1.20	1	19	0.018	1	U	R.VQLADQYMSSAALGDANKDAIDR.G
22940	408 – 430	1234.5898	2467.1651	2467.1649	0.10	1	121	4.3e-12	1	U	R.VQLADQYMSSAALGDANKDAIDR.G + Oxidation (M)
22941	408 – 430	823.3957	2467.1653	2467.1649	0.17	1	106	1.1e-10	1	U	R.VQLADQYMSSAALGDANKDAIDR.G + Oxidation (M)
22945	408 – 430	823.7249	2468.1527	2468.1489	1.56	1	84	1.2e-08	1	U	R.VQLADQYMSSAALGDANKDAIDR.G + Deamidated (NQ); Oxidation (M)
22946	408 – 430	823.7279	2468.1619	2468.1489	5.26	1	35	0.00051	1	U	R.VQLADQYMSSAALGDANKDAIDR.G + Deamidated (NQ); Oxidation (M)
24248	408 – 435	987.8079	2960.4019	2960.3974	1.50	2	45	6.2e-05	1	U	R.VQLADQYMSSAALGDANKDAIDRGTFFG.-

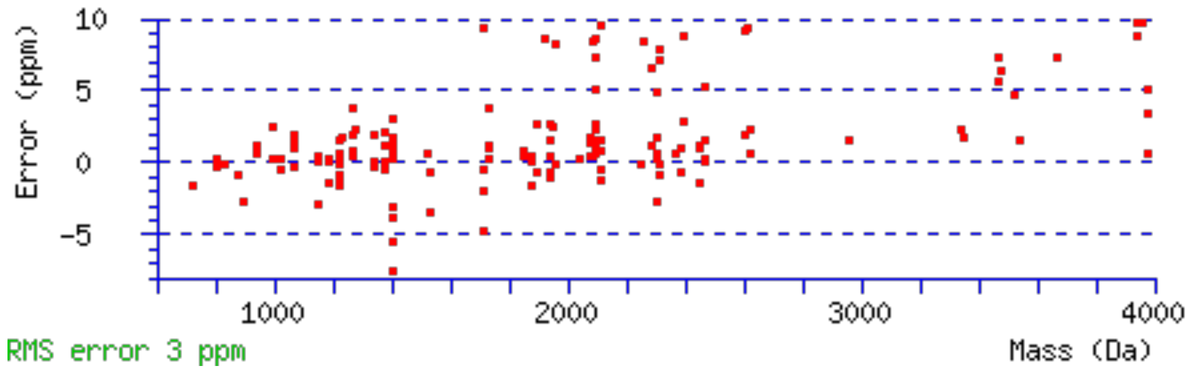

Mascot: http://www.matrixscience.com/

MASCOT Search Results

Protein View: sp|P06512|PSAB_SPIOL

>sp|P06512|PSAB_SPIOL Photosystem I P700 chlorophyll a apoprotein A2 OS=Spinacia oleracea OX=3562 GN=psaB PE=3 SV=1

**Database:**	Uni-Spinach
**Score:**	3707
**Nominal mass (M_r):**	82491
**Calculated pI:**	6.72

Sequence similarity is available as an NCBI BLAST search of sp|P06512|PSAB_SPIOL against nr.

Search parameters

**MS data file:**	\\172.16.0.213\tank\windowsVM\Bill Cramer\052418\MGF\wc_QE_052418_Cramer_gel_4.mgf
**Enzyme:**	Trypsin: cuts C-term side of KR unless next residue is P.
**Fixed modifications:**	Carbamidomethyl (C)
**Variable modifications:**	Acetyl (K), Acetyl (Protein N-term), Deamidated (NQ), Oxidation (M)

Protein sequence coverage: 22%

Matched peptides shown in ***bold red***.

```
  1 MALRFPRFSQ GLAQDPTTRR IWFGIATAHD FESHDDITEE RLYQNIFASH
 51 FGQLAIIFLW TSGNLFHVAW QGNFESWVQD PLHVRPIAHA IWDPHFGQPA
101 VEAFTRGGAL GPVNIAYSGV YQWWYTIGLR TNEDLYTGAL FLLFLSVISL
151 LGGWLHLQPK WKPSVSWFKN AESRLNHHLS GLFGVSSLAW TGHLVHVAIP
201 GSRGEYVRWN NFLDVLPHPQ GLGPLFTGQW NLYAQNPDSS SHLFGTSQGA
251 GTAILTLLGG FHPQTQSLWL TDMAHHHLAI AFVFLVAGHM YRTNFGIGHS
301 MKDLLEAHIP PGGRLGRGHK GLYDTINNSL HFQLGLALAS LGVITSLVAQ
351 HMYSLPAYAF IAQDFTTQAA LYTHHQYIAG FIMTGAFAHG AIFFIRDYNP
401 EQNEDNVLAR MLDHKEAIIS HLSWASLFLG FHTLGLYVHN DVMLAFGTPE
451 KQILIEPIFA QWIQSAHGKT SYGFDVLLSS TSGPAFNAGR SIWLPGWLNA
501 VNENSNSLFL TIGPGDFLVH HAIALGLHTT TLILVKGALD ARGSKLMPDK
551 KDFGYSFPCD GPGRGGTCDI SAWDAFYLAV FWMLNTIGWV TFYWHWKHIT
601 LWQGNVSQFN ESSTYLMGWL RDYLWLNSSQ LINGYNPFGM NSLSVWAWMF
651 LFGHLVWATG FMFLISWRGY WQELIETLAW AHERTPLANL IRWRDKPVAL
701 SIVQARLVGL AHFSVGYIFT YAAFLIASTS GKFG
```

Unformatted sequence string: 734 residues (for pasting into other applications).

Sort peptides by ◉ Residue Number ○ Increasing Mass ○ Decreasing Mass

Show predicted peptides also

Query	Start – End	Observed	Mr(expt)	Mr(calc)	ppm	M	Score	Expect	Rank	U	Peptide
494	2 – 7	380.2348	758.4551	758.4551	-0.055	1	7	1.2	2	U	M.ALRFPR.F
495	2 – 7	380.2352	758.4559	758.4551	1.03	1	3	0.77	1	U	M.ALRFPR.F
960	2 – 7	401.2402	800.4659	800.4657	0.21	1	16	0.22	1	U	M.ALRFPR.F + Acetyl (Protein N-term)
961	2 – 7	401.2403	800.4660	800.4657	0.46	1	9	0.13	1	U	M.ALRFPR.F + Acetyl (Protein N-term)
962	2 – 7	401.2407	800.4668	800.4657	1.36	1	20	0.015	1	U	M.ALRFPR.F + Acetyl (Protein N-term)
963	2 – 7	401.2410	800.4674	800.4657	2.11	1	23	0.052	1	U	M.ALRFPR.F + Acetyl (Protein N-term)
9905	8 – 19	660.8301	1319.6456	1319.6470	-1.02	0	28	0.0048	1	U	R.FSQGLAQDPTTR.R
9906	8 – 19	660.8301	1319.6457	1319.6470	-0.96	0	34	0.00072	1	U	R.FSQGLAQDPTTR.R
9907	8 – 19	440.8892	1319.6458	1319.6470	-0.87	0	6	0.27	1	U	R.FSQGLAQDPTTR.R
9908	8 – 19	440.8895	1319.6466	1319.6470	-0.29	0	9	0.14	1	U	R.FSQGLAQDPTTR.R
9909	8 – 19	660.8307	1319.6469	1319.6470	-0.074	0	32	0.00099	1	U	R.FSQGLAQDPTTR.R
9910	8 – 19	660.8310	1319.6474	1319.6470	0.29	0	56	5.5e-06	1	U	R.FSQGLAQDPTTR.R
9911	8 – 19	660.8311	1319.6476	1319.6470	0.44	0	39	0.00022	1	U	R.FSQGLAQDPTTR.R
9912	8 – 19	660.8313	1319.6480	1319.6470	0.78	0	26	0.0054	1	U	R.FSQGLAQDPTTR.R
9913	8 – 19	660.8314	1319.6483	1319.6470	1.01	0	43	0.0001	1	U	R.FSQGLAQDPTTR.R
9915	8 – 19	660.8317	1319.6489	1319.6470	1.45	0	58	3.6e-06	1	U	R.FSQGLAQDPTTR.R
9916	8 – 19	660.8319	1319.6493	1319.6470	1.75	0	60	5.6e-06	1	U	R.FSQGLAQDPTTR.R

9917	8 – 19	660.8356	1319.6567	1319.6470	7.33	0	1	0.78	1	U	R.FSQGLAQDPTTR.R
9931	8 – 19	661.3246	1320.6347	1320.6310	2.79	0	43	9.5e-05	1	U	R.FSQGLAQDPTTR.R + Deamidated (NQ)
12952	8 – 20	492.9229	1475.7470	1475.7481	-0.75	1	13	0.13	1	U	R.FSQGLAQDPTTRR.I
12953	8 – 20	738.8808	1475.7470	1475.7481	-0.71	1	10	0.63	1	U	R.FSQGLAQDPTTRR.I
12954	8 – 20	738.8811	1475.7477	1475.7481	-0.28	1	4	0.67	1	U	R.FSQGLAQDPTTRR.I
12955	8 – 20	738.8815	1475.7484	1475.7481	0.24	1	23	0.054	1	U	R.FSQGLAQDPTTRR.I
12956	8 – 20	492.9235	1475.7488	1475.7481	0.45	1	20	0.014	1	U	R.FSQGLAQDPTTRR.I
12957	8 – 20	492.9239	1475.7500	1475.7481	1.27	1	12	0.067	1	U	R.FSQGLAQDPTTRR.I
12959	8 – 20	492.9246	1475.7521	1475.7481	2.68	1	13	0.055	1	U	R.FSQGLAQDPTTRR.I
12960	8 – 20	492.9247	1475.7524	1475.7481	2.90	1	27	0.003	1	U	R.FSQGLAQDPTTRR.I
12976	8 – 20	493.2509	1476.7307	1476.7321	-0.93	1	17	0.027	1	U	R.FSQGLAQDPTTRR.I + Deamidated (NQ)
23635	20 – 41	662.0657	2644.2337	2644.2306	1.18	1	79	4.1e-08	1	U	R.RIWFGIATAHDFESHDDITEER.L
23636	20 – 41	662.0658	2644.2341	2644.2306	1.33	1	55	6.9e-06	1	U	R.RIWFGIATAHDFESHDDITEER.L
23637	20 – 41	662.0660	2644.2351	2644.2306	1.69	1	48	3.5e-05	1	U	R.RIWFGIATAHDFESHDDITEER.L
23638	20 – 41	662.0666	2644.2371	2644.2306	2.45	1	84	1.5e-08	1	U	R.RIWFGIATAHDFESHDDITEER.L
23037	21 – 41	830.3830	2488.1272	2488.1295	-0.93	0	24	0.0056	1	U	R.IWFGIATAHDFESHDDITEER.L
23038	21 – 41	830.3842	2488.1307	2488.1295	0.47	0	97	7.4e-10	1	U	R.IWFGIATAHDFESHDDITEER.L
23039	21 – 41	623.0404	2488.1323	2488.1295	1.12	0	91	4.3e-09	1	U	R.IWFGIATAHDFESHDDITEER.L
6768	161 – 169	582.8134	1163.6122	1163.6128	-0.51	0	32	0.0009	1	U	K.WKPSVSWFK.N
6769	161 – 169	388.8781	1163.6126	1163.6128	-0.18	0	1	0.78	1	U	K.WKPSVSWFK.N
6770	161 – 169	388.8783	1163.6130	1163.6128	0.22	0	20	0.014	1	U	K.WKPSVSWFK.N
6772	161 – 169	388.8783	1163.6132	1163.6128	0.37	0	33	0.00079	1	U	K.WKPSVSWFK.N
6773	161 – 169	388.8785	1163.6138	1163.6128	0.87	0	2	0.63	1	U	K.WKPSVSWFK.N
6776	161 – 169	388.8790	1163.6150	1163.6128	1.95	0	8	0.18	1	U	K.WKPSVSWFK.N
6777	161 – 169	582.8148	1163.6151	1163.6128	2.01	0	20	0.012	1	U	K.WKPSVSWFK.N
5340	293 – 302	364.5153	1090.5242	1090.5230	1.14	0	11	0.092	1	U	R.TNFGIGHSMK.D
5341	293 – 302	546.2696	1090.5246	1090.5230	1.52	0	20	0.035	1	U	R.TNFGIGHSMK.D
5342	293 – 302	364.5156	1090.5250	1090.5230	1.90	0	11	0.098	1	U	R.TNFGIGHSMK.D
5343	293 – 302	546.2708	1090.5270	1090.5230	3.66	0	39	0.0012	1	U	R.TNFGIGHSMK.D
5355	293 – 302	546.7609	1091.5073	1091.5070	0.30	0	14	0.046	1	U	R.TNFGIGHSMK.D + Deamidated (NQ)
5356	293 – 302	364.8431	1091.5074	1091.5070	0.40	0	4	0.44	1	U	R.TNFGIGHSMK.D + Deamidated (NQ)
5650	293 – 302	369.8464	1106.5173	1106.5179	-0.56	0	20	0.013	1	U	R.TNFGIGHSMK.D + Oxidation (M)
5651	293 – 302	554.2660	1106.5175	1106.5179	-0.38	0	38	0.00026	1	U	R.TNFGIGHSMK.D + Oxidation (M)
5652	293 – 302	554.2665	1106.5185	1106.5179	0.54	0	40	0.00018	1	U	R.TNFGIGHSMK.D + Oxidation (M)
5667	293 – 302	554.7589	1107.5032	1107.5019	1.17	0	15	0.04	1	U	R.TNFGIGHSMK.D + Deamidated (NQ); Oxidation (M)
22339	293 – 314	587.5557	2346.1937	2346.1903	1.47	1	17	0.041	1	U	R.TNFGIGHSMKDLLEAHIPPGGR.L
22340	293 – 314	587.5557	2346.1939	2346.1903	1.52	1	21	0.011	1	U	R.TNFGIGHSMKDLLEAHIPPGGR.L
22341	293 – 314	587.5561	2346.1954	2346.1903	2.17	1	10	0.11	1	U	R.TNFGIGHSMKDLLEAHIPPGGR.L
22342	293 – 314	587.5562	2346.1956	2346.1903	2.28	1	13	0.063	1	U	R.TNFGIGHSMKDLLEAHIPPGGR.L
22343	293 – 314	587.5568	2346.1980	2346.1903	3.27	1	15	0.04	1	U	R.TNFGIGHSMKDLLEAHIPPGGR.L
22352	293 – 314	587.8049	2347.1904	2347.1743	6.86	1	1	0.85	1	U	R.TNFGIGHSMKDLLEAHIPPGGR.L + Deamidated (NQ)
22353	293 – 314	587.8049	2347.1906	2347.1743	6.96	1	2	0.6	1	U	R.TNFGIGHSMKDLLEAHIPPGGR.L + Deamidated (NQ)
22355	293 – 314	783.4046	2347.1920	2347.1743	7.54	1	18	0.022	1	U	R.TNFGIGHSMKDLLEAHIPPGGR.L + Deamidated (NQ)
22356	293 – 314	587.8057	2347.1938	2347.1743	8.31	1	26	0.0052	1	U	R.TNFGIGHSMKDLLEAHIPPGGR.L + Deamidated (NQ)
22357	293 – 314	587.8059	2347.1945	2347.1743	8.62	1	21	0.0099	1	U	R.TNFGIGHSMKDLLEAHIPPGGR.L + Deamidated (NQ)
22358	293 – 314	587.8065	2347.1967	2347.1743	9.56	1	5	0.32	1	U	R.TNFGIGHSMKDLLEAHIPPGGR.L + Deamidated (NQ)
22463	293 – 314	591.5532	2362.1839	2362.1852	-0.55	1	17	0.023	1	U	R.TNFGIGHSMKDLLEAHIPPGGR.L + Oxidation (M)
22464	293 – 314	788.4020	2362.1841	2362.1852	-0.45	1	39	0.00024	1	U	R.TNFGIGHSMKDLLEAHIPPGGR.L + Oxidation (M)
22465	293 – 314	788.4022	2362.1847	2362.1852	-0.23	1	32	0.00092	1	U	R.TNFGIGHSMKDLLEAHIPPGGR.L + Oxidation (M)
22466	293 – 314	591.5537	2362.1858	2362.1852	0.26	1	14	0.055	1	U	R.TNFGIGHSMKDLLEAHIPPGGR.L + Oxidation (M)
22467	293 – 314	473.4447	2362.1872	2362.1852	0.84	1	12	0.075	1	U	R.TNFGIGHSMKDLLEAHIPPGGR.L + Oxidation (M)
22468	293 – 314	473.4448	2362.1875	2362.1852	0.96	1	10	0.18	1	U	R.TNFGIGHSMKDLLEAHIPPGGR.L + Oxidation (M)
22469	293 – 314	591.5542	2362.1877	2362.1852	1.06	1	8	0.2	1	U	R.TNFGIGHSMKDLLEAHIPPGGR.L + Oxidation (M)

22470	293 – 314	591.5543	2362.1881	2362.1852	1.25	1	34	0.00068	1	U	R.TNFGIGHSMKDLLEAHIPPGGR.L + Oxidation (M)
22473	293 – 314	591.5544	2362.1884	2362.1852	1.37	1	21	0.011	1	U	R.TNFGIGHSMKDLLEAHIPPGGR.L + Oxidation (M)
22474	293 – 314	788.4034	2362.1885	2362.1852	1.39	1	59	3e-06	1	U	R.TNFGIGHSMKDLLEAHIPPGGR.L + Oxidation (M)
22475	293 – 314	591.5544	2362.1887	2362.1852	1.47	1	26	0.0035	1	U	R.TNFGIGHSMKDLLEAHIPPGGR.L + Oxidation (M)
22476	293 – 314	591.5544	2362.1887	2362.1852	1.48	1	16	0.032	1	U	R.TNFGIGHSMKDLLEAHIPPGGR.L + Oxidation (M)
22477	293 – 314	591.5546	2362.1892	2362.1852	1.70	1	9	0.16	1	U	R.TNFGIGHSMKDLLEAHIPPGGR.L + Oxidation (M)
22479	293 – 314	591.5547	2362.1899	2362.1852	1.98	1	3	0.79	1	U	R.TNFGIGHSMKDLLEAHIPPGGR.L + Oxidation (M)
22480	293 – 314	591.5548	2362.1901	2362.1852	2.09	1	12	0.082	1	U	R.TNFGIGHSMKDLLEAHIPPGGR.L + Oxidation (M)
22481	293 – 314	591.5550	2362.1909	2362.1852	2.40	1	1	0.77	1	U	R.TNFGIGHSMKDLLEAHIPPGGR.L + Oxidation (M)
22482	293 – 314	591.5555	2362.1928	2362.1852	3.23	1	10	0.11	1	U	R.TNFGIGHSMKDLLEAHIPPGGR.L + Oxidation (M)
22489	293 – 314	591.8032	2363.1838	2363.1692	6.17	1	5	0.37	1	U	R.TNFGIGHSMKDLLEAHIPPGGR.L + Deamidated (NQ); Oxidation (M)
22491	293 – 314	788.7358	2363.1855	2363.1692	6.90	1	10	0.13	1	U	R.TNFGIGHSMKDLLEAHIPPGGR.L + Deamidated (NQ); Oxidation (M)
22492	293 – 314	788.7363	2363.1870	2363.1692	7.52	1	15	0.039	1	U	R.TNFGIGHSMKDLLEAHIPPGGR.L + Deamidated (NQ); Oxidation (M)
22493	293 – 314	788.7365	2363.1877	2363.1692	7.83	1	18	0.02	1	U	R.TNFGIGHSMKDLLEAHIPPGGR.L + Deamidated (NQ); Oxidation (M)
22494	293 – 314	591.8044	2363.1884	2363.1692	8.13	1	3	0.5	1	U	R.TNFGIGHSMKDLLEAHIPPGGR.L + Deamidated (NQ); Oxidation (M)
22495	293 – 314	788.7377	2363.1914	2363.1692	9.38	1	16	0.032	1	U	R.TNFGIGHSMKDLLEAHIPPGGR.L + Deamidated (NQ); Oxidation (M)
8962	303 – 314	637.8453	1273.6760	1273.6779	-1.50	0	55	3e-05	1	U	K.DLLEAHIPPGGR.L
8963	303 – 314	637.8456	1273.6767	1273.6779	-0.95	0	57	1.9e-05	1	U	K.DLLEAHIPPGGR.L
8964	303 – 314	637.8459	1273.6773	1273.6779	-0.44	0	35	0.0024	1	U	K.DLLEAHIPPGGR.L
8965	303 – 314	637.8460	1273.6773	1273.6779	-0.41	0	59	1.6e-05	1	U	K.DLLEAHIPPGGR.L
8966	303 – 314	637.8460	1273.6774	1273.6779	-0.35	0	59	1.6e-05	1	U	K.DLLEAHIPPGGR.L
8967	303 – 314	425.5665	1273.6776	1273.6779	-0.23	0	32	0.0011	1	U	K.DLLEAHIPPGGR.L
8968	303 – 314	637.8461	1273.6776	1273.6779	-0.20	0	44	0.00013	1	U	K.DLLEAHIPPGGR.L
8969	303 – 314	425.5665	1273.6776	1273.6779	-0.20	0	23	0.0076	1	U	K.DLLEAHIPPGGR.L
8970	303 – 314	425.5665	1273.6778	1273.6779	-0.077	0	15	0.04	1	U	K.DLLEAHIPPGGR.L
8971	303 – 314	425.5665	1273.6778	1273.6779	-0.065	0	16	0.032	1	U	K.DLLEAHIPPGGR.L
8972	303 – 314	637.8462	1273.6778	1273.6779	-0.050	0	45	0.00032	1	U	K.DLLEAHIPPGGR.L
8973	303 – 314	637.8462	1273.6779	1273.6779	0.014	0	8	0.18	1	U	K.DLLEAHIPPGGR.L
8974	303 – 314	637.8462	1273.6779	1273.6779	0.014	0	59	1.6e-05	1	U	K.DLLEAHIPPGGR.L
8975	303 – 314	637.8462	1273.6779	1273.6779	0.033	0	50	0.00013	1	U	K.DLLEAHIPPGGR.L
8976	303 – 314	425.5666	1273.6779	1273.6779	0.034	0	18	0.023	1	U	K.DLLEAHIPPGGR.L
8977	303 – 314	637.8463	1273.6780	1273.6779	0.090	0	54	4.9e-05	1	U	K.DLLEAHIPPGGR.L
8978	303 – 314	637.8463	1273.6780	1273.6779	0.097	0	57	1.9e-05	1	U	K.DLLEAHIPPGGR.L
8979	303 – 314	425.5666	1273.6781	1273.6779	0.14	0	32	0.0085	1	U	K.DLLEAHIPPGGR.L
8980	303 – 314	637.8463	1273.6781	1273.6779	0.14	0	41	0.00044	1	U	K.DLLEAHIPPGGR.L
8981	303 – 314	425.5666	1273.6781	1273.6779	0.18	0	19	0.02	1	U	K.DLLEAHIPPGGR.L
8982	303 – 314	637.8463	1273.6781	1273.6779	0.19	0	45	8.8e-05	1	U	K.DLLEAHIPPGGR.L
8983	303 – 314	425.5667	1273.6781	1273.6779	0.22	0	29	0.0022	1	U	K.DLLEAHIPPGGR.L
8984	303 – 314	425.5667	1273.6782	1273.6779	0.27	0	32	0.0012	1	U	K.DLLEAHIPPGGR.L
8985	303 – 314	637.8464	1273.6783	1273.6779	0.33	0	54	4.8e-05	1	U	K.DLLEAHIPPGGR.L
8986	303 – 314	637.8464	1273.6783	1273.6779	0.35	0	50	8.8e-05	1	U	K.DLLEAHIPPGGR.L
8987	303 – 314	425.5667	1273.6784	1273.6779	0.41	0	29	0.0022	1	U	K.DLLEAHIPPGGR.L
8988	303 – 314	425.5668	1273.6785	1273.6779	0.50	0	32	0.0013	1	U	K.DLLEAHIPPGGR.L
8989	303 – 314	425.5668	1273.6785	1273.6779	0.50	0	32	0.0011	1	U	K.DLLEAHIPPGGR.L
8990	303 – 314	425.5668	1273.6785	1273.6779	0.51	0	32	0.0013	1	U	K.DLLEAHIPPGGR.L
8991	303 – 314	425.5668	1273.6786	1273.6779	0.56	0	17	0.024	1	U	K.DLLEAHIPPGGR.L
8992	303 – 314	637.8466	1273.6786	1273.6779	0.59	0	54	4.7e-05	1	U	K.DLLEAHIPPGGR.L
8993	303 – 314	637.8466	1273.6787	1273.6779	0.61	0	59	1.6e-05	1	U	K.DLLEAHIPPGGR.L
8994	303 – 314	425.5668	1273.6787	1273.6779	0.67	0	31	0.0014	1	U	K.DLLEAHIPPGGR.L
8995	303 – 314	425.5669	1273.6787	1273.6779	0.69	0	10	0.7	1	U	K.DLLEAHIPPGGR.L
8996	303 – 314	425.5669	1273.6789	1273.6779	0.81	0	19	0.02	1	U	K.DLLEAHIPPGGR.L
8997	303 – 314	425.5669	1273.6790	1273.6779	0.87	0	31	0.0014	1	U	K.DLLEAHIPPGGR.L
8998	303 – 314	425.5669	1273.6790	1273.6779	0.89	0	17	0.03	1	U	K.DLLEAHIPPGGR.L

8999	303 – 314	425.5670	1273.6791	1273.6779	0.99	0	29	0.002	1	U	K.DLLEAHIPPGGR.L
9000	303 – 314	637.8469	1273.6791	1273.6779	1.00	0	30	0.0014	1	U	K.DLLEAHIPPGGR.L
9001	303 – 314	637.8469	1273.6792	1273.6779	1.01	0	54	3.3e-05	1	U	K.DLLEAHIPPGGR.L
9003	303 – 314	425.5670	1273.6793	1273.6779	1.12	0	29	0.0023	1	U	K.DLLEAHIPPGGR.L
9004	303 – 314	425.5671	1273.6793	1273.6779	1.14	0	29	0.0022	1	U	K.DLLEAHIPPGGR.L
9005	303 – 314	637.8469	1273.6793	1273.6779	1.16	0	30	0.0016	1	U	K.DLLEAHIPPGGR.L
9006	303 – 314	425.5671	1273.6794	1273.6779	1.18	0	19	0.023	1	U	K.DLLEAHIPPGGR.L
9007	303 – 314	637.8470	1273.6795	1273.6779	1.24	0	54	4.8e-05	1	U	K.DLLEAHIPPGGR.L
9008	303 – 314	425.5671	1273.6795	1273.6779	1.26	0	15	0.043	1	U	K.DLLEAHIPPGGR.L
9009	303 – 314	637.8470	1273.6795	1273.6779	1.27	0	57	1.9e-05	1	U	K.DLLEAHIPPGGR.L
9010	303 – 314	425.5671	1273.6795	1273.6779	1.30	0	29	0.0021	1	U	K.DLLEAHIPPGGR.L
9011	303 – 314	425.5671	1273.6796	1273.6779	1.33	0	18	0.023	1	U	K.DLLEAHIPPGGR.L
9012	303 – 314	637.8471	1273.6796	1273.6779	1.34	0	46	0.00012	1	U	K.DLLEAHIPPGGR.L
9013	303 – 314	425.5672	1273.6796	1273.6779	1.39	0	32	0.0011	1	U	K.DLLEAHIPPGGR.L
9014	303 – 314	425.5672	1273.6797	1273.6779	1.40	0	29	0.011	1	U	K.DLLEAHIPPGGR.L
9015	303 – 314	425.5672	1273.6797	1273.6779	1.41	0	32	0.0086	1	U	K.DLLEAHIPPGGR.L
9016	303 – 314	637.8471	1273.6797	1273.6779	1.43	0	59	1.6e-05	1	U	K.DLLEAHIPPGGR.L
9017	303 – 314	425.5672	1273.6798	1273.6779	1.49	0	32	0.0013	1	U	K.DLLEAHIPPGGR.L
9018	303 – 314	637.8472	1273.6798	1273.6779	1.52	0	59	1.6e-05	1	U	K.DLLEAHIPPGGR.L
9019	303 – 314	425.5672	1273.6799	1273.6779	1.57	0	40	0.00019	1	U	K.DLLEAHIPPGGR.L
9020	303 – 314	637.8473	1273.6800	1273.6779	1.64	0	37	0.00043	1	U	K.DLLEAHIPPGGR.L
9021	303 – 314	637.8474	1273.6802	1273.6779	1.85	0	50	0.00012	1	U	K.DLLEAHIPPGGR.L
9022	303 – 314	425.5674	1273.6803	1273.6779	1.87	0	16	0.033	1	U	K.DLLEAHIPPGGR.L
9023	303 – 314	637.8475	1273.6805	1273.6779	2.02	0	36	0.00051	1	U	K.DLLEAHIPPGGR.L
9024	303 – 314	637.8476	1273.6806	1273.6779	2.15	0	41	0.00014	1	U	K.DLLEAHIPPGGR.L
9025	303 – 314	637.8477	1273.6809	1273.6779	2.39	0	47	4.1e-05	1	U	K.DLLEAHIPPGGR.L
9026	303 – 314	637.8478	1273.6811	1273.6779	2.55	0	53	4.5e-05	1	U	K.DLLEAHIPPGGR.L
9027	303 – 314	425.5677	1273.6811	1273.6779	2.55	0	15	0.037	1	U	K.DLLEAHIPPGGR.L
9028	303 – 314	425.5678	1273.6815	1273.6779	2.82	0	29	0.0019	1	U	K.DLLEAHIPPGGR.L
9029	303 – 314	637.8484	1273.6823	1273.6779	3.45	0	27	0.003	1	U	K.DLLEAHIPPGGR.L
9030	303 – 314	637.8487	1273.6829	1273.6779	3.98	0	39	0.00021	1	U	K.DLLEAHIPPGGR.L
15983	397 – 410	838.8758	1675.7371	1675.7438	-3.97	0	21	0.011	1	U	R.DYNPEQNEDNVLAR.M
15984	397 – 410	838.8780	1675.7414	1675.7438	-1.39	0	89	4.7e-09	1	U	R.DYNPEQNEDNVLAR.M
15985	397 – 410	838.8782	1675.7418	1675.7438	-1.17	0	76	8e-08	1	U	R.DYNPEQNEDNVLAR.M
15986	397 – 410	838.8790	1675.7434	1675.7438	-0.22	0	43	8.6e-05	1	U	R.DYNPEQNEDNVLAR.M
15987	397 – 410	838.8792	1675.7438	1675.7438	0.027	0	99	5.1e-10	1	U	R.DYNPEQNEDNVLAR.M
15988	397 – 410	559.5889	1675.7448	1675.7438	0.59	0	71	2.2e-07	1	U	R.DYNPEQNEDNVLAR.M
15989	397 – 410	559.5891	1675.7455	1675.7438	1.01	0	67	5.4e-07	1	U	R.DYNPEQNEDNVLAR.M
15990	397 – 410	838.8816	1675.7486	1675.7438	2.89	0	67	4.8e-07	1	U	R.DYNPEQNEDNVLAR.M
16009	397 – 410	839.3725	1676.7305	1676.7278	1.65	0	66	6.5e-07	1	U	R.DYNPEQNEDNVLAR.M + Deamidated (NQ)
16010	397 – 410	839.3750	1676.7354	1676.7278	4.58	0	89	4.8e-09	1	U	R.DYNPEQNEDNVLAR.M + Deamidated (NQ)
20270	452 – 469	1040.0728	2078.1310	2078.1313	-0.12	0	69	3.2e-07	1	U	K.QILIEPIFAQWIQSAHGK.T
20271	452 – 469	520.5405	2078.1328	2078.1313	0.73	0	11	0.1	1	U	K.QILIEPIFAQWIQSAHGK.T
20272	452 – 469	693.7192	2078.1356	2078.1313	2.11	0	67	4.9e-07	1	U	K.QILIEPIFAQWIQSAHGK.T
20762	470 – 490	1074.0238	2146.0331	2146.0331	0.016	0	129	8.8e-13	1	U	K.TSYGFDVLLSSTSGPAFNAGR.S
20763	470 – 490	716.3521	2146.0344	2146.0331	0.60	0	86	8.6e-09	1	U	K.TSYGFDVLLSSTSGPAFNAGR.S
20764	470 – 490	1074.0248	2146.0350	2146.0331	0.90	0	122	3.9e-12	1	U	K.TSYGFDVLLSSTSGPAFNAGR.S
20765	470 – 490	716.3525	2146.0356	2146.0331	1.16	0	81	2.8e-08	1	U	K.TSYGFDVLLSSTSGPAFNAGR.S
20770	470 – 490	1074.5158	2147.0171	2147.0171	-0.031	0	106	1.2e-10	1	U	K.TSYGFDVLLSSTSGPAFNAGR.S + Deamidated (NQ)
20771	470 – 490	716.6803	2147.0191	2147.0171	0.91	0	76	6.9e-08	1	U	K.TSYGFDVLLSSTSGPAFNAGR.S + Deamidated (NQ)
257	546 – 551	366.2097	730.4048	730.4047	0.12	1	2	0.61	1	U	K.LMPDKK.D
258	546 – 551	366.2098	730.4051	730.4047	0.46	1	16	0.052	1	U	K.LMPDKK.D
259	546 – 551	366.2101	730.4057	730.4047	1.30	1	3	1	2	U	K.LMPDKK.D
352	546 – 551	374.2063	746.3980	746.3996	-2.15	1	1	0.79	1	U	K.LMPDKK.D + Oxidation (M)
15012	551 – 564	534.9070	1601.6991	1601.6933	3.60	1	22	0.0083	1	U	K.KDFGYSFPCDGPGR.G
12910	552 – 564	737.8067	1473.5989	1473.5983	0.38	0	51	1.7e-05	1	U	K.DFGYSFPCDGPGR.G
12911	552 – 564	737.8071	1473.5996	1473.5983	0.84	0	45	6.1e-05	1	U	K.DFGYSFPCDGPGR.G
2292	685 – 692	449.2758	896.5371	896.5443	-8.08	0	1	0.76	1	U	R.TPLANLIR.W
2294	685 – 692	449.2794	896.5442	896.5443	-0.18	0	10	0.34	1	U	R.TPLANLIR.W
2295	685 – 692	449.2796	896.5446	896.5443	0.30	0	11	0.28	1	U	R.TPLANLIR.W
2297	685 – 692	449.2797	896.5449	896.5443	0.69	0	26	0.0098	1	U	R.TPLANLIR.W
2299	685 – 692	449.2798	896.5451	896.5443	0.83	0	6	0.98	1	U	R.TPLANLIR.W
2300	685 – 692	449.2799	896.5453	896.5443	1.10	0	35	0.0011	1	U	R.TPLANLIR.W
2301	685 – 692	449.2801	896.5456	896.5443	1.40	0	40	0.00039	1	U	R.TPLANLIR.W
2302	685 – 692	449.2802	896.5458	896.5443	1.65	0	32	0.0022	1	U	R.TPLANLIR.W
2303	685 – 692	449.2802	896.5458	896.5443	1.69	0	26	0.0089	1	U	R.TPLANLIR.W
2304	685 – 692	449.2807	896.5469	896.5443	2.83	0	29	0.0048	1	U	R.TPLANLIR.W
2315	685 – 692	449.7715	897.5284	897.5283	0.091	0	24	0.015	1	U	R.TPLANLIR.W + Deamidated (NQ)

2317	685 - 692	449.7719	897.5293	897.5283	1.11	0	16	0.044	1	U	R.TPLANLIR.W + Deamidated (NQ)
15475	693 - 706	546.9857	1637.9353	1637.9365	-0.76	1	46	6.8e-05	1	U	R.WRDKPVALSIVQAR.L
15476	693 - 706	819.9753	1637.9361	1637.9365	-0.25	1	30	0.01	1	U	R.WRDKPVALSIVQAR.L
15477	693 - 706	410.4917	1637.9377	1637.9365	0.70	1	21	0.092	1	U	R.WRDKPVALSIVQAR.L
15478	693 - 706	546.9866	1637.9379	1637.9365	0.84	1	48	0.00011	1	U	R.WRDKPVALSIVQAR.L
15479	693 - 706	410.4918	1637.9381	1637.9365	0.98	1	16	0.033	1	U	R.WRDKPVALSIVQAR.L
9417	695 - 706	432.9260	1295.7561	1295.7561	-0.017	0	36	0.00044	1	U	R.DKPVALSIVQAR.L
9418	695 - 706	648.8854	1295.7562	1295.7561	0.042	0	41	0.00016	1	U	R.DKPVALSIVQAR.L
9419	695 - 706	432.9261	1295.7566	1295.7561	0.38	0	35	0.00052	1	U	R.DKPVALSIVQAR.L
9420	695 - 706	432.9262	1295.7568	1295.7561	0.52	0	4	0.46	1	U	R.DKPVALSIVQAR.L
9421	695 - 706	432.9262	1295.7568	1295.7561	0.55	0	4	0.42	1	U	R.DKPVALSIVQAR.L
9422	695 - 706	648.8858	1295.7571	1295.7561	0.77	0	57	4.8e-06	1	U	R.DKPVALSIVQAR.L
9423	695 - 706	432.9264	1295.7574	1295.7561	1.02	0	25	0.0049	1	U	R.DKPVALSIVQAR.L
9424	695 - 706	432.9268	1295.7586	1295.7561	1.89	0	45	6.5e-05	1	U	R.DKPVALSIVQAR.L
9435	695 - 706	433.2543	1296.7412	1296.7401	0.80	0	23	0.098	1	U	R.DKPVALSIVQAR.L + Deamidated (NQ)

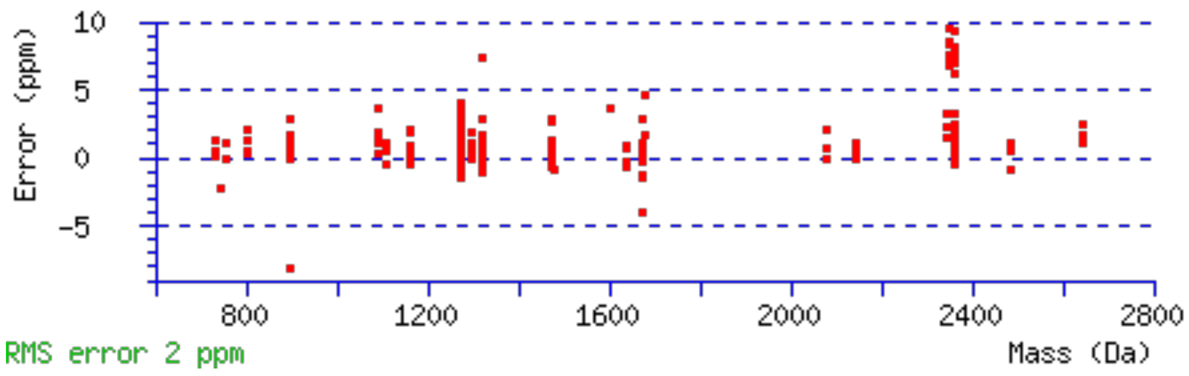

Mascot: http://www.matrixscience.com/

MASCOT Search Results

Protein View: sp|P00825|ATPB_SPIOL

>sp|P00825|ATPB_SPIOL ATP synthase subunit beta, chloroplastic OS=Spinacia oleracea OX=3562 GN=atpB PE=1 SV=2

**Database:**	Uni-Spinach
**Score:**	3573
**Nominal mass (M_r):**	53768
**Calculated pI:**	5.22

Sequence similarity is available as **an NCBI BLAST search of sp|P00825|ATPB_SPIOL against nr**.

Search parameters

**MS data file:**	\\172.16.0.213\tank\windowsVM\Bill Cramer\052418\MGF\wc_QE_052418_Cramer_gel_4.mgf
**Enzyme:**	Trypsin: cuts C-term side of KR unless next residue is P.
**Fixed modifications:**	Carbamidomethyl (C)
**Variable modifications:**	Acetyl (K), Acetyl (Protein N-term), Deamidated (NQ), Oxidation (M)

Protein sequence coverage: 71%

Matched peptides shown in ***bold red***.

```
  1 MRINPTTSDP GVSTLEKKNL GRIAQIIGPV LDVAFPPGKM PNIYNALIVK
 51 GRDTAGQPMN VTCEVQQLLG NNRVRAVAMS ATDGLTRGME VIDTGAPLSV
101 PVGGATLGRI FNVLGEPVDN LGPVDTRTTS PIHRSAPAFT QLDTKLSIFE
151 TGIKVVDLLA PYRRGGKIGL FGGAGVGKTV LIMELINNIA KAHGGVSVFG
201 GVGERTREGN DLYMEMKESG VINEQNIAES KVALVYGQMN EPPGARMRVG
251 LTALTMAEYF RDVNEQDVLL FIDNIFRFVQ AGSEVSALLG RMPSAVGYQP
301 TLSTEMGSLQ ERITSTKEGS ITSIQAVYVP ADDLTDPAPA TTFAHLDATT
351 VLSRGLAAKG IYPAVDPLDS TSTMLQPRIV GEEHYEIAQR VKETLQRYKE
401 LQDIIAILGL DELSEEDRLT VARARKIERF LSQPFFVAEV FTGSPGKYVG
451 LAETIRGFQL ILSGELDSLP EQAFYLVGNI DEATAKAMNL EMESKLKK
```

Unformatted sequence string: **498 residues** (for pasting into other applications).

Sort peptides by ◉ Residue Number ○ Increasing Mass ○ Decreasing Mass

Show predicted peptides also

Query	Start – End	Observed	Mr(expt)	Mr(calc)	ppm	M	Score	Expect	Rank	U	Peptide
14369	3 – 17	779.9019	1557.7892	1557.7886	0.35	0	65	7.4e-07	1	U	R.INPTTSDPGVSTLEK.K
14370	3 – 17	779.9019	1557.7893	1557.7886	0.41	0	66	6e-07	1	U	R.INPTTSDPGVSTLEK.K
16103	3 – 18	562.9680	1685.8823	1685.8836	-0.74	1	31	0.0011	1	U	R.INPTTSDPGVSTLEKK.N
16105	3 – 18	562.9686	1685.8840	1685.8836	0.26	1	56	6.2e-06	1	U	R.INPTTSDPGVSTLEKK.N
16106	3 – 18	843.9497	1685.8849	1685.8836	0.76	1	42	0.00011	1	U	R.INPTTSDPGVSTLEKK.N
16667	23 – 39	579.0100	1734.0083	1734.0080	0.18	0	32	0.0011	1	U	R.IAQIIGPVLDVAFPPGK.M
16668	23 – 39	868.0120	1734.0095	1734.0080	0.84	0	29	0.0018	1	U	R.IAQIIGPVLDVAFPPGK.M
16670	23 – 39	868.0123	1734.0100	1734.0080	1.18	0	43	9.7e-05	1	U	R.IAQIIGPVLDVAFPPGK.M
9044	40 – 50	638.3584	1274.7022	1274.7056	-2.71	0	11	0.1	1	U	K.MPNIYNALIVK.G
9045	40 – 50	638.3596	1274.7047	1274.7056	-0.72	0	46	4.6e-05	1	U	K.MPNIYNALIVK.G
9046	40 – 50	638.3602	1274.7058	1274.7056	0.14	0	55	6.8e-06	1	U	K.MPNIYNALIVK.G
9056	40 – 50	638.8515	1275.6885	1275.6897	-0.92	0	51	1.5e-05	1	U	K.MPNIYNALIVK.G + Deamidated (NQ)
9319	40 – 50	646.3586	1290.7026	1290.7006	1.60	0	76	7.9e-08	1	U	K.MPNIYNALIVK.G + Oxidation (M)
7353	76 – 87	596.8032	1191.5919	1191.5918	0.15	0	58	3.9e-06	1	U	R.AVAMSATDGLTR.G
7354	76 – 87	596.8033	1191.5921	1191.5918	0.29	0	45	0.00015	1	U	R.AVAMSATDGLTR.G
7732	76 – 87	604.7975	1207.5804	1207.5867	-5.19	0	30	0.0017	1	U	R.AVAMSATDGLTR.G + Oxidation (M)
20394	88 – 109	1049.0554	2096.0963	2096.0936	1.29	0	95	1.3e-09	1	U	R.GMEVIDTGAPLSVPVGGATLGR.I
20395	88 – 109	699.7063	2096.0970	2096.0936	1.59	0	42	0.00011	1	U	R.GMEVIDTGAPLSVPVGGATLGR.I
20396	88 – 109	699.7066	2096.0979	2096.0936	2.03	0	13	0.061	1	U	R.GMEVIDTGAPLSVPVGGATLGR.I
20397	88 – 109	1049.0564	2096.0983	2096.0936	2.25	0	78	5.1e-08	1	U	R.GMEVIDTGAPLSVPVGGATLGR.I
20519	88 – 109	1057.0527	2112.0908	2112.0885	1.08	0	95	1.3e-09	1	U	R.GMEVIDTGAPLSVPVGGATLGR.I + Oxidation (M)
20520	88 – 109	1057.0529	2112.0912	2112.0885	1.28	0	82	2e-08	1	U	R.GMEVIDTGAPLSVPVGGATLGR.I + Oxidation (M)
19378	110 – 127	978.0165	1954.0185	1954.0160	1.29	0	105	5.6e-10	1	U	R.IFNVLGEPVDNLGPVDTR.T
19379	110 – 127	652.3473	1954.0200	1954.0160	2.04	0	45	6.8e-05	1	U	R.IFNVLGEPVDNLGPVDTR.T
19380	110 – 127	652.3475	1954.0208	1954.0160	2.46	0	97	8.8e-10	1	U	R.IFNVLGEPVDNLGPVDTR.T
1089	128 – 134	406.2246	810.4346	810.4348	-0.21	0	30	0.0029	1	U	R.TTSPIHR.S
7034	135 – 145	589.8065	1177.5983	1177.5979	0.38	0	34	0.00061	1	U	R.SAPAFTQLDTK.L
7035	135 – 145	589.8065	1177.5984	1177.5979	0.39	0	45	6.3e-05	1	U	R.SAPAFTQLDTK.L
3792	146 – 154	504.2930	1006.5714	1006.5699	1.48	0	43	0.00059	1	U	K.LSIFETGIK.V
3793	146 – 154	504.2933	1006.5720	1006.5699	2.10	0	44	0.00048	1	U	K.LSIFETGIK.V
4338	155 – 163	523.3059	1044.5972	1044.5968	0.37	0	51	0.00016	1	U	K.VVDLLAPYR.R
4341	155 – 163	523.3067	1044.5988	1044.5968	1.97	0	40	0.0021	1	U	K.VVDLLAPYR.R
4342	155 – 163	523.3069	1044.5992	1044.5968	2.35	0	51	0.00018	1	U	K.VVDLLAPYR.R
7570	155 – 164	601.3563	1200.6980	1200.6979	0.14	1	25	0.005	1	U	K.VVDLLAPYRR.G
7571	155 – 164	401.2400	1200.6982	1200.6979	0.25	1	33	0.00074	1	U	K.VVDLLAPYRR.G
7572	155 – 164	401.2400	1200.6983	1200.6979	0.35	1	20	0.013	1	U	K.VVDLLAPYRR.G
3370	168 – 178	488.2853	974.5560	974.5549	1.07	0	46	0.0002	1		K.IGLFGGAGVGK.T
3371	168 – 178	488.2855	974.5565	974.5549	1.64	0	25	0.011	1		K.IGLFGGAGVGK.T
12864	179 – 191	736.4332	1470.8519	1470.8479	2.67	0	55	3.5e-05	1	U	K.TVLIMELINNIAK.A

12865	179 – 191	491.2916	1470.8529	1470.8479	3.38	0	28	0.0073	1	U	K.TVLIMELINNIAK.A
13175	179 – 191	744.4295	1486.8444	1486.8429	1.03	0	58	6.7e-06	1	U	K.TVLIMELINNIAK.A + Oxidation (M)
13194	179 – 191	744.9227	1487.8308	1487.8269	2.61	0	55	7.4e-06	1	U	K.TVLIMELINNIAK.A + Deamidated (NQ); Oxidation (M)
10028	192 – 205	443.5595	1327.6567	1327.6633	-5.00	0	33	0.00076	1	U	K.AHGGVSVFGGVGER.T
10029	192 – 205	443.5615	1327.6626	1327.6633	-0.57	0	43	9.7e-05	1	U	K.AHGGVSVFGGVGER.T
10030	192 – 205	664.8386	1327.6626	1327.6633	-0.56	0	32	0.00095	1	U	K.AHGGVSVFGGVGER.T
10031	192 – 205	664.8389	1327.6633	1327.6633	-0.0060	0	20	0.013	1	U	K.AHGGVSVFGGVGER.T
13143	206 – 217	496.2256	1485.6550	1485.6592	-2.79	1	26	0.0037	1	U	R.TREGNDLYMEMK.E
13144	206 – 217	743.8363	1485.6581	1485.6592	-0.71	1	8	0.19	1	U	R.TREGNDLYMEMK.E
13145	206 – 217	496.2273	1485.6601	1485.6592	0.63	1	23	0.0076	1	U	R.TREGNDLYMEMK.E
13162	206 – 217	496.5557	1486.6453	1486.6432	1.40	1	8	0.19	1	U	R.TREGNDLYMEMK.E + Deamidated (NQ)
13445	206 – 217	501.5583	1501.6531	1501.6541	-0.63	1	5	0.33	1	U	R.TREGNDLYMEMK.E + Oxidation (M)
13446	206 – 217	751.8343	1501.6541	1501.6541	-0.0100	1	19	0.016	1	U	R.TREGNDLYMEMK.E + Oxidation (M)
13447	206 – 217	751.8353	1501.6560	1501.6541	1.26	1	18	0.13	1	U	R.TREGNDLYMEMK.E + Oxidation (M)
13448	206 – 217	501.5593	1501.6562	1501.6541	1.39	1	3	0.48	1	U	R.TREGNDLYMEMK.E + Oxidation (M)
13738	206 – 217	759.8298	1517.6450	1517.6490	-2.64	1	4	0.38	1	U	R.TREGNDLYMEMK.E + 2 Oxidation (M)
13739	206 – 217	506.8895	1517.6467	1517.6490	-1.54	1	8	0.17	1	U	R.TREGNDLYMEMK.E + 2 Oxidation (M)
24310	206 – 231	755.3517	3017.3776	3017.3593	6.07	2	57	5e-06	1	U	R.TREGNDLYMEMKESGVINEQNIAESK.V + Deamidated (NQ); 2 Oxidation (M)
8131	208 – 217	615.2632	1228.5119	1228.5104	1.26	0	34	0.00066	1	U	R.EGNDLYMEMK.E
8468	208 – 217	623.2589	1244.5033	1244.5053	-1.63	0	38	0.00054	1	U	R.EGNDLYMEMK.E + Oxidation (M)
8469	208 – 217	623.2608	1244.5071	1244.5053	1.41	0	14	0.16	1	U	R.EGNDLYMEMK.E + Oxidation (M)
23958	208 – 231	921.0834	2760.2283	2760.2105	6.43	1	24	0.0051	1	U	R.EGNDLYMEMKESGVINEQNIAESK.V + Deamidated (NQ); 2 Oxidation (M)
23960	208 – 231	921.0844	2760.2312	2760.2105	7.49	1	2	0.65	1	U	R.EGNDLYMEMKESGVINEQNIAESK.V + Deamidated (NQ); 2 Oxidation (M)
13722	218 – 231	759.3758	1516.7370	1516.7369	0.100	0	53	9.7e-06	1	U	K.ESGVINEQNIAESK.V
13723	218 – 231	759.3768	1516.7390	1516.7369	1.39	0	46	4.4e-05	1	U	K.ESGVINEQNIAESK.V
13743	218 – 231	759.8676	1517.7206	1517.7209	-0.17	0	18	0.02	1	U	K.ESGVINEQNIAESK.V + Deamidated (NQ)
14994	232 – 246	534.6082	1600.8028	1600.8031	-0.21	0	26	0.0036	1	U	K.VALVYGQMNEPPGAR.M
14995	232 – 246	801.4088	1600.8031	1600.8031	-0.032	0	55	6.3e-06	1	U	K.VALVYGQMNEPPGAR.M
14997	232 – 246	801.4103	1600.8060	1600.8031	1.78	0	90	4e-09	1	U	K.VALVYGQMNEPPGAR.M
14998	232 – 246	801.4106	1600.8067	1600.8031	2.22	0	72	1.9e-07	1	U	K.VALVYGQMNEPPGAR.M
14999	232 – 246	801.4108	1600.8070	1600.8031	2.44	0	79	3.6e-08	1	U	K.VALVYGQMNEPPGAR.M
15001	232 – 246	801.4120	1600.8094	1600.8031	3.92	0	45	5.8e-05	1	U	K.VALVYGQMNEPPGAR.M
15002	232 – 246	801.4123	1600.8101	1600.8031	4.37	0	43	8.9e-05	1	U	K.VALVYGQMNEPPGAR.M
15003	232 – 246	801.4132	1600.8119	1600.8031	5.45	0	28	0.0024	1	U	K.VALVYGQMNEPPGAR.M
15172	232 – 246	809.4042	1616.7939	1616.7981	-2.56	0	41	0.00013	1	U	K.VALVYGQMNEPPGAR.M + Oxidation (M)
15173	232 – 246	539.9391	1616.7955	1616.7981	-1.57	0	17	0.023	1	U	K.VALVYGQMNEPPGAR.M + Oxidation (M)
15174	232 – 246	809.4055	1616.7965	1616.7981	-0.98	0	20	0.44	2	U	K.VALVYGQMNEPPGAR.M + Oxidation (M)
15175	232 – 246	809.4056	1616.7966	1616.7981	-0.94	0	53	1.1e-05	1	U	K.VALVYGQMNEPPGAR.M + Oxidation (M)
15176	232 – 246	809.4056	1616.7966	1616.7981	-0.90	0	38	0.00025	1	U	K.VALVYGQMNEPPGAR.M + Oxidation (M)
15177	232 – 246	809.4058	1616.7970	1616.7981	-0.67	0	59	3.5e-06	1	U	K.VALVYGQMNEPPGAR.M + Oxidation (M)
15178	232 – 246	809.4062	1616.7979	1616.7981	-0.12	0	42	0.00012	1	U	K.VALVYGQMNEPPGAR.M + Oxidation (M)
15179	232 – 246	809.4069	1616.7992	1616.7981	0.68	0	36	0.00044	1	U	K.VALVYGQMNEPPGAR.M + Oxidation (M)
12861	249 – 261	736.3826	1470.7506	1470.7541	-2.38	0	45	6.3e-05	1	U	R.VGLTALTMAEYFR.D
12862	249 – 261	491.2591	1470.7555	1470.7541	1.00	0	32	0.0011	1	U	R.VGLTALTMAEYFR.D
13170	249 – 261	744.3826	1486.7507	1486.7490	1.18	0	74	1.1e-07	1	U	R.VGLTALTMAEYFR.D + Oxidation (M)
13171	249 – 261	744.3840	1486.7534	1486.7490	2.95	0	57	4.5e-06	1	U	R.VGLTALTMAEYFR.D + Oxidation (M)
12098	278 – 291	717.3910	1432.7675	1432.7674	0.038	0	91	3.1e-09	1	U	R.FVQAGSEVSALLGR.M
12099	278 – 291	478.5968	1432.7686	1432.7674	0.80	0	4	0.41	1	U	R.FVQAGSEVSALLGR.M
12101	278 – 291	717.3925	1432.7704	1432.7674	2.05	0	47	3.6e-05	1	U	R.FVQAGSEVSALLGR.M
21761	292 – 312	761.3644	2281.0715	2281.0719	-0.16	0	57	4.3e-06	1	U	R.MPSAVGYQPTLSTEMGSLQER.I
21762	292 – 312	1141.5436	2281.0726	2281.0719	0.33	0	94	1.6e-09	1	U	R.MPSAVGYQPTLSTEMGSLQER.I
21763	292 – 312	761.3649	2281.0729	2281.0719	0.45	0	89	4.9e-09	1	U	R.MPSAVGYQPTLSTEMGSLQER.I
21766	292 – 312	1142.0431	2282.0716	2282.0559	6.91	0	44	6.9e-05	1	U	R.MPSAVGYQPTLSTEMGSLQER.I + Deamidated (NQ)
21903	292 – 312	1149.5364	2297.0582	2297.0668	-3.73	0	112	9.8e-11	1	U	R.MPSAVGYQPTLSTEMGSLQER.I + Oxidation (M)
21904	292 – 312	766.6956	2297.0650	2297.0668	-0.77	0	91	3.3e-09	1	U	R.MPSAVGYQPTLSTEMGSLQER.I + Oxidation (M)
21906	292 – 312	1149.5421	2297.0695	2297.0668	1.21	0	78	4.8e-08	1	U	R.MPSAVGYQPTLSTEMGSLQER.I + Oxidation (M)
21907	292 – 312	766.6971	2297.0696	2297.0668	1.21	0	64	9.7e-07	1	U	R.MPSAVGYQPTLSTEMGSLQER.I + Oxidation (M)
22073	292 – 312	772.0274	2313.0604	2313.0617	-0.56	0	51	1.8e-05	1	U	R.MPSAVGYQPTLSTEMGSLQER.I + 2 Oxidation (M)
22084	292 – 312	772.3601	2314.0585	2314.0457	5.53	0	71	2.2e-07	1	U	R.MPSAVGYQPTLSTEMGSLQER.I + Deamidated (NQ); 2 Oxidation (M)
24948	313 – 354	1094.5681	4374.2434	4374.2119	7.20	1	61	1.7e-06	1	U	R.ITSTKEGSITSIQAVYVPADDLTDPAPATTFAHLDATTVLSR.[illegible] + Deamidated (NQ)
24860	318 – 354	1282.3134	3843.9182	3843.9055	3.32	0	53	1.2e-05	1	U	K.EGSITSIQAVYVPADDLTDPAPATTFAHLDATTVLSR.G + Deamidated (NQ)
24861	318 – 354	961.9879	3843.9223	3843.9055	4.38	0	42	0.00011	1	U	K.EGSITSIQAVYVPADDLTDPAPATTFAHLDATTVLSR.G + Deamidated (NQ)
20141	360 – 378	1031.0184	2060.0222	2060.0248	-1.29	0	111	3.6e-11	1	U	K.GIYPAVDPLDSTSTMLQPR.I
20142	360 – 378	1031.0188	2060.0230	2060.0248	-0.87	0	107	8.6e-11	1	U	K.GIYPAVDPLDSTSTMLQPR.I
20143	360 – 378	687.6819	2060.0240	2060.0248	-0.40	0	84	1.2e-08	1	U	K.GIYPAVDPLDSTSTMLQPR.I
20144	360 – 378	687.6828	2060.0265	2060.0248	0.81	0	95	1.2e-09	1	U	K.GIYPAVDPLDSTSTMLQPR.I
20251	360 – 378	1039.0171	2076.0196	2076.0198	-0.064	0	54	8.9e-06	1	U	K.GIYPAVDPLDSTSTMLQPR.I + Oxidation (M)
20252	360 – 378	693.0143	2076.0212	2076.0198	0.67	0	59	2.8e-06	1	U	K.GIYPAVDPLDSTSTMLQPR.I + Oxidation (M)
12305	379 – 390	722.3641	1442.7137	1442.7154	-1.17	0	55	2.9e-05	1	U	R.IVGEEHYEIAQR.V
12306	379 – 390	481.9121	1442.7145	1442.7154	-0.58	0	31	0.0012	1	U	R.IVGEEHYEIAQR.V
12308	379 – 390	722.3674	1442.7203	1442.7154	3.42	0	67	8.4e-07	1	U	R.IVGEEHYEIAQR.V
2000	391 – 397	437.2615	872.5084	872.5080	0.47	1	12	1.2	2	U	R.VKETLQR.Y
3987	448 – 456	511.2891	1020.5637	1020.5604	3.24	0	44	0.00028	1	U	K.YVGLAETIR.G
4490	487 – 495	526.7413	1051.4681	1051.4678	0.35	0	24	0.0059	1	U	K.AMNLEMESK.L
4819	487 – 495	534.7387	1067.4628	1067.4627	0.13	0	8	0.16	1	U	K.AMNLEMESK.L + Oxidation (M)

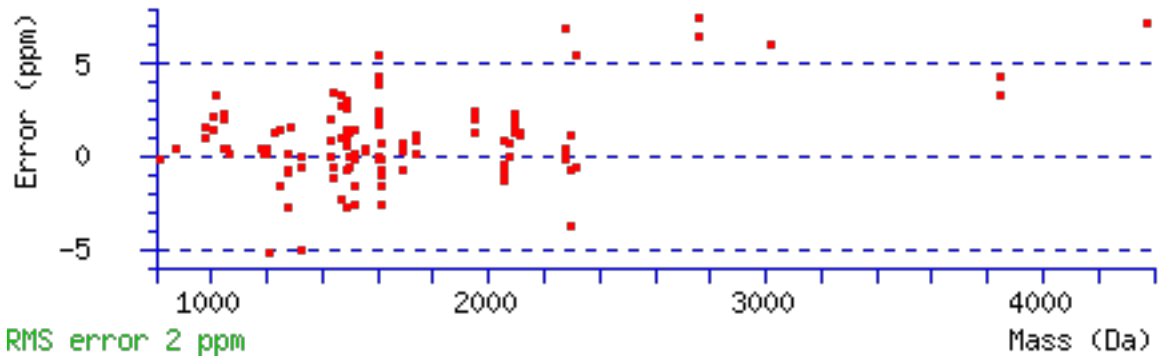

Mascot: http://www.matrixscience.com/

MATRIX SCIENCE MASCOT Search Results

Protein View: tr|A0A0K9RD58|A0A0K9RD58_SPIOL

>tr|A0A0K9RD58|A0A0K9RD58_SPIOL AAA domain-containing protein OS=Spinacia oleracea OX=3562 GN=SOVF_080200 PE=3 SV=1

**Database:**	Uni-Spinach
**Score:**	3281
**Nominal mass (M_r):**	73817
**Calculated pI:**	5.70

Sequence similarity is available as an NCBI BLAST search of tr|A0A0K9RD58|A0A0K9RD58_SPIOL against nr.

Search parameters

**MS data file:**	\\172.16.0.213\tank\windowsVM\Bill Cramer\052418\MGF\wc_QE_052418_Cramer_gel_4.mgf
**Enzyme:**	Trypsin: cuts C-term side of KR unless next residue is P.
**Fixed modifications:**	Carbamidomethyl (C)
**Variable modifications:**	Acetyl (K), Acetyl (Protein N-term), Deamidated (NQ), Oxidation (M)

Protein sequence coverage: 65%

Matched peptides shown in ***bold red***.

```
  1 MAASSACLLG HGSSTSSSKT ILKERLFHGH LFAGRSVLLK SQKAFIVKAS
 51 SDKAQSDGRR GFLKLLLGNA GVASTLVASG NANADEQGVS SSRMSYSRFL
101 EYLDKDRVQK VDLFENGTIA IVEAISPELG NRVQRVRVQL PGLSQELLQK
151 LREKNIDFAA HNAQEDSGSV LFNLIGNLAF PLILIGGLFL LSRRSGGGMG
201 GPGGPGNPLA FGQSKAKFQM EPNTGVTFDD VAGVDEAKQD FMEVVEFLKK
251 PERFTAVGAR IPKGVLLVGP PGTGKTLLAK AIAGEAGVPF FSISGSEFVE
301 MFVGVGASRV RDLFKKAKEN APCIVFVDEI DAVGRSRGTG IGGGNDEREQ
351 TLNQLLTEMD GFEGNTGIIV VAATNRADIL DSALLRPGRF DRQVSVDVPD
401 VRGRTDILKV HASNKKFDGD VSLEVIAMRT PGFSGADLAN LLNEAAILAG
451 RRGKTAICSK EIDDSIDRIV AGMEGTVMTD SKSKSLVAYH EVGHAICGTL
501 TPGHDAVQKV TLVPRGQARG LTWFIPSDDP TLISKQQLFA RIVGGLGGRA
551 AEEIIFGESE VTTGAAGDLQ QITGLAKQMV TTFGMSDIGP WSLMDASAQS
601 GDVIMRMMAR NSMSERLAED IDNAVKKITD EAYQIALTHI RNNREAIDKI
651 VDVLLETETV SGDEFRTLLA EFTEIPVENR VPAAAASPVT V
```

Unformatted sequence string: 691 residues (for pasting into other applications).

Sort peptides by ◉ Residue Number ○ Increasing Mass ○ Decreasing Mass

Show predicted peptides also

Query	Start – End	Observed	Mr(expt)	Mr(calc)	ppm	M	Score	Expect	Rank	U	Peptide
7479	99 – 107	599.8074	1197.6003	1197.6030	-2.21	1	44	0.00013	1	U	R.FLEYLDKDR.V
7480	99 – 107	599.8094	1197.6042	1197.6030	1.02	1	47	8.3e-05	1	U	R.FLEYLDKDR.V
7481	99 – 107	400.2089	1197.6048	1197.6030	1.50	1	24	0.0058	1	U	R.FLEYLDKDR.V
7482	99 – 107	400.2089	1197.6048	1197.6030	1.53	1	43	0.0001	1	U	R.FLEYLDKDR.V
7483	99 – 107	400.2093	1197.6061	1197.6030	2.64	1	11	0.084	1	U	R.FLEYLDKDR.V
23835	108 – 132	904.8254	2711.4542	2711.4494	1.80	1	28	0.0025	1	U	R.VQKVDLFENGTIAIVEAISPELGNR.V
23838	108 – 132	905.1541	2712.4404	2712.4334	2.60	1	27	0.0031	1	U	R.VQKVDLFENGTIAIVEAISPELGNR.V + Deamidated (NQ)
22430	111 – 132	1179.6150	2357.2154	2357.2114	1.68	0	108	7e-11	1	U	K.VDLFENGTIAIVEAISPELGNR.V + Deamidated (NQ)
22431	111 – 132	786.7458	2357.2156	2357.2114	1.79	0	86	2.7e-08	1	U	K.VDLFENGTIAIVEAISPELGNR.V + Deamidated (NQ)
12496	138 – 150	726.9245	1451.8345	1451.8348	-0.20	0	50	4.1e-05	1	U	R.VQLPGLSQELLQK.L
12497	138 – 150	484.9523	1451.8350	1451.8348	0.16	0	62	1.6e-06	1	U	R.VQLPGLSQELLQK.L
12498	138 – 150	726.9249	1451.8353	1451.8348	0.36	0	50	3.2e-05	1	U	R.VQLPGLSQELLQK.L
12499	138 – 150	484.9534	1451.8385	1451.8348	2.59	0	14	0.044	1	U	R.VQLPGLSQELLQK.L
12500	138 – 150	726.9270	1451.8395	1451.8348	3.28	0	10	0.26	1	U	R.VQLPGLSQELLQK.L
19643	194 – 215	662.6580	1984.9520	1984.9538	-0.87	1	37	0.00036	1	U	R.RSGGGMGGPGGPGNPLAFGQSK.A
19644	194 – 215	993.4841	1984.9537	1984.9538	-0.031	1	49	2.7e-05	1	U	R.RSGGGMGGPGGPGNPLAFGQSK.A
19645	194 – 215	662.6595	1984.9566	1984.9538	1.41	1	55	6.9e-06	1	U	R.RSGGGMGGPGGPGNPLAFGQSK.A
19735	194 – 215	667.9904	2000.9495	2000.9487	0.41	1	73	1.5e-07	1	U	R.RSGGGMGGPGGPGNPLAFGQSK.A + Oxidation (M)
19736	194 – 215	667.9909	2000.9509	2000.9487	1.09	1	44	7.2e-05	1	U	R.RSGGGMGGPGGPGNPLAFGQSK.A + Oxidation (M)
19750	194 – 215	1001.9819	2001.9492	2001.9327	8.24	1	19	0.017	1	U	R.RSGGGMGGPGGPGNPLAFGQSK.A + Deamidated (NQ); Oxidation (M)
17848	195 – 215	915.4318	1828.8490	1828.8527	-2.02	0	98	7e-10	1	U	R.SGGGMGGPGGPGNPLAFGQSK.A
17849	195 – 215	915.4337	1828.8528	1828.8527	0.071	0	100	4.1e-10	1	U	R.SGGGMGGPGGPGNPLAFGQSK.A
17850	195 – 215	610.6253	1828.8542	1828.8527	0.85	0	11	0.092	1	U	R.SGGGMGGPGGPGNPLAFGQSK.A
17851	195 – 215	610.6258	1828.8555	1828.8527	1.58	0	12	0.075	1	U	R.SGGGMGGPGGPGNPLAFGQSK.A
18063	195 – 215	923.4251	1844.8357	1844.8476	-6.46	0	67	5.7e-07	1	U	R.SGGGMGGPGGPGNPLAFGQSK.A + Oxidation (M)
18065	195 – 215	923.4307	1844.8468	1844.8476	-0.41	0	107	9.1e-11	1	U	R.SGGGMGGPGGPGNPLAFGQSK.A + Oxidation (M)
18066	195 – 215	615.9568	1844.8487	1844.8476	0.60	0	23	0.0073	1	U	R.SGGGMGGPGGPGNPLAFGQSK.A + Oxidation (M)
18067	195 – 215	615.9578	1844.8517	1844.8476	2.22	0	16	0.034	1	U	R.SGGGMGGPGGPGNPLAFGQSK.A + Oxidation (M)
21686	218 – 238	1135.5177	2269.0209	2269.0209	-0.00044	0	127	1.3e-12	1	U	K.FQMEPNTGVTFDDVAGVDEAK.Q

11401	239 – 249	700.8422	1399.6699	1399.6694	0.37	0	39	0.00021	1	U	K.QDFMEVVEFLK.K + Oxidation (M)
165	254 – 260	361.2032	720.3919	720.3919	0.019	0	28	0.0025	1	U	R.FTAVGAR.I
166	254 – 260	361.2033	720.3920	720.3919	0.12	0	27	0.0029	1	U	R.FTAVGAR.I
5400	264 – 275	547.8321	1093.6496	1093.6496	0.017	0	24	0.012	1	U	K.GVLLVGPPGTGK.T
5401	264 – 275	547.8322	1093.6498	1093.6496	0.20	0	42	0.00021	1	U	K.GVLLVGPPGTGK.T
5402	264 – 275	547.8324	1093.6502	1093.6496	0.62	0	18	0.083	1	U	K.GVLLVGPPGTGK.T
24211	281 – 309	978.8150	2933.4232	2933.4270	-1.30	0	50	4.1e-05	1	U	K.AIAGEAGVPFFSISGSEFVEMFVGVGASR.V + Oxidation (M)
708	310 – 315	389.2342	776.4538	776.4545	-0.88	1	18	0.093	1	U	R.VRDLFK.K
2425	310 – 316	453.2821	904.5496	904.5494	0.14	2	14	0.16	1	U	R.VRDLFKK.A
20466	317 – 335	701.6900	2102.0482	2102.0466	0.74	1	5	0.31	1	U	K.AKENAPCIVFVDEIDAVGR.S
20467	317 – 335	701.6903	2102.0492	2102.0466	1.23	1	27	0.0032	1	U	K.AKENAPCIVFVDEIDAVGR.S
18839	319 – 335	952.4650	1902.9155	1902.9146	0.50	0	75	2.1e-07	1	U	K.ENAPCIVFVDEIDAVGR.S
24910	338 – 376	1017.2477	4064.9616	4064.9233	9.43	1	50	2e-05	1	U	R.GTGIGGGNDEREQTLNQLLTEMDGFEGNTGIIVVAATNR.A + 2 Deamidated (NQ); Oxidation (M)
11351	377 – 389	698.8983	1395.7820	1395.7834	-1.02	0	38	0.001	1	U	R.ADILDSALLRPGR.F
11352	377 – 389	466.2685	1395.7836	1395.7834	0.16	0	25	0.0048	1	U	R.ADILDSALLRPGR.F
11353	377 – 389	698.9000	1395.7855	1395.7834	1.54	0	21	0.017	1	U	R.ADILDSALLRPGR.F
11354	377 – 389	466.2692	1395.7856	1395.7834	1.61	0	34	0.0007	1	U	R.ADILDSALLRPGR.F
5750	393 – 402	557.2938	1112.5731	1112.5826	-8.52	0	5	0.31	1	U	R.QVSVDVPDVR.G
5752	393 – 402	557.2962	1112.5779	1112.5826	-4.20	0	26	0.0042	1	U	R.QVSVDVPDVR.G
5753	393 – 402	557.2994	1112.5842	1112.5826	1.46	0	64	1.8e-06	1	U	R.QVSVDVPDVR.G
14671	416 – 429	790.4105	1578.8064	1578.8076	-0.72	1	56	7.3e-05	1	U	K.KFDGDVSLEVIAMR.T
14672	416 – 429	527.2768	1578.8084	1578.8076	0.54	1	33	0.004	1	U	K.KFDGDVSLEVIAMR.T
14673	416 – 429	790.4120	1578.8094	1578.8076	1.19	1	38	0.0021	1	U	K.KFDGDVSLEVIAMR.T
14675	416 – 429	527.2775	1578.8108	1578.8076	2.02	1	34	0.00086	1	U	K.KFDGDVSLEVIAMR.T
14898	416 – 429	532.6085	1594.8037	1594.8025	0.73	1	45	0.00032	1	U	K.KFDGDVSLEVIAMR.T + Oxidation (M)
14899	416 – 429	798.4094	1594.8042	1594.8025	1.06	1	52	1.2e-05	1	U	K.KFDGDVSLEVIAMR.T + Oxidation (M)
14900	416 – 429	798.4106	1594.8066	1594.8025	2.59	1	68	4.3e-07	1	U	K.KFDGDVSLEVIAMR.T + Oxidation (M)
12468	417 – 429	726.3642	1450.7138	1450.7126	0.83	0	61	1.8e-06	1	U	K.FDGDVSLEVIAMR.T
12780	417 – 429	734.3596	1466.7046	1466.7075	-2.03	0	34	0.00068	1	U	K.FDGDVSLEVIAMR.T + Oxidation (M)
20904	430 – 451	724.3878	2170.1414	2170.1382	1.49	0	68	8.1e-07	1	U	R.TPGFSGADLANLLNEAAILAGR.R
3177	461 – 468	481.7246	961.4346	961.4352	-0.62	0	36	0.0044	1	U	K.EIDDSIDR.I
3178	461 – 468	481.7249	961.4352	961.4352	-0.027	0	17	0.028	1	U	K.EIDDSIDR.I
22570	461 – 482	794.7109	2381.1109	2381.1090	0.77	1	42	0.00011	1	U	K.EIDDSIDRIVAGMEGTVMTDSK.S
22641	461 – 482	800.0401	2397.0985	2397.1040	-2.26	1	35	0.00048	1	U	K.EIDDSIDRIVAGMEGTVMTDSK.S + Oxidation (M)
12179	469 – 482	719.8473	1437.6800	1437.6844	-3.01	0	14	0.049	1	U	R.IVAGMEGTVMTDSK.S
12180	469 – 482	719.8485	1437.6825	1437.6844	-1.31	0	5	0.36	1	U	R.IVAGMEGTVMTDSK.S
12182	469 – 482	719.8507	1437.6868	1437.6844	1.73	0	83	1.5e-08	1	U	R.IVAGMEGTVMTDSK.S
12183	469 – 482	719.8509	1437.6873	1437.6844	2.07	0	98	6.3e-10	1	U	R.IVAGMEGTVMTDSK.S
12184	469 – 482	719.8512	1437.6878	1437.6844	2.42	0	48	2.9e-05	1	U	R.IVAGMEGTVMTDSK.S
12521	469 – 482	727.8466	1453.6786	1453.6793	-0.47	0	55	6.6e-06	1	U	R.IVAGMEGTVMTDSK.S + Oxidation (M)
12522	469 – 482	727.8474	1453.6803	1453.6793	0.73	0	84	1.4e-08	1	U	R.IVAGMEGTVMTDSK.S + Oxidation (M)
12523	469 – 482	727.8502	1453.6858	1453.6793	4.46	0	53	1.1e-05	1	U	R.IVAGMEGTVMTDSK.S + Oxidation (M)
12830	469 – 482	735.8434	1469.6722	1469.6742	-1.38	0	41	0.00014	1	U	R.IVAGMEGTVMTDSK.S + 2 Oxidation (M)
12833	469 – 482	735.8443	1469.6740	1469.6742	-0.10	0	33	0.00088	1	U	R.IVAGMEGTVMTDSK.S + 2 Oxidation (M)
17299	520 – 535	597.3167	1788.9282	1788.9298	-0.92	0	14	0.046	1	U	R.GLTWFIPSDDPTLISK.Q
17300	520 – 535	895.4723	1788.9301	1788.9298	0.18	0	28	0.0025	1	U	R.GLTWFIPSDDPTLISK.Q
516	536 – 541	381.7165	761.4185	761.4184	0.096	0	22	0.093	1	U	K.QQLFAR.I
517	536 – 541	381.7167	761.4188	761.4184	0.47	0	22	0.059	1	U	K.QQLFAR.I
227	542 – 549	364.7240	727.4334	727.4341	-0.91	0	37	0.0018	1	U	R.IVGGLGGR.A
228	542 – 549	364.7241	727.4336	727.4341	-0.70	0	34	0.0048	1	U	R.IVGGLGGR.A
24035	550 – 577	705.6137	2818.4258	2818.4236	0.78	0	103	2.1e-10	1	U	R.AAEEIIFGESEVTTGAAGDLQQITGLAK.Q
24036	550 – 577	940.4831	2818.4274	2818.4236	1.34	0	121	4.8e-12	1	U	R.AAEEIIFGESEVTTGAAGDLQQITGLAK.Q
24434	578 – 606	1049.8141	3146.4206	3146.4181	0.79	0	96	1e-09	1	U	K.QMVTTFGMSDIGPWSLMDASAQSGDVIMR.M + Oxidation (M)
24436	578 – 606	1050.1484	3147.4235	3147.4021	6.78	0	103	2.3e-10	1	U	K.QMVTTFGMSDIGPWSLMDASAQSGDVIMR.M + Deamidated (NQ); Oxidation (M)
5258	617 – 626	544.2850	1086.5555	1086.5557	-0.16	0	44	0.0001	1	U	R.LAEDIDNAVK.K
5259	617 – 626	544.2854	1086.5562	1086.5557	0.52	0	60	1.6e-05	1	U	R.LAEDIDNAVK.K
5260	617 – 626	544.2856	1086.5566	1086.5557	0.86	0	19	0.016	1	U	R.LAEDIDNAVK.K
5279	617 – 626	544.7771	1087.5396	1087.5397	-0.064	0	41	0.00013	1	U	R.LAEDIDNAVK.K + Deamidated (NQ)
7859	617 – 627	405.8899	1214.6477	1214.6506	-2.39	1	22	0.0082	1	U	R.LAEDIDNAVKK.I
7860	617 – 627	405.8905	1214.6498	1214.6506	-0.69	1	17	0.068	1	U	R.LAEDIDNAVKK.I
7862	617 – 627	608.3330	1214.6514	1214.6506	0.64	1	35	0.0024	1	U	R.LAEDIDNAVKK.I
17102	627 – 641	886.4880	1770.9614	1770.9628	-0.79	1	91	6.4e-09	1	U	K.KITDEAYQIALTHIR.N
17103	627 – 641	591.3282	1770.9628	1770.9628	-0.0017	1	73	1.6e-07	1	U	K.KITDEAYQIALTHIR.N
17104	627 – 641	591.3283	1770.9631	1770.9628	0.17	1	26	0.0037	1	U	K.KITDEAYQIALTHIR.N
17105	627 – 641	443.7481	1770.9633	1770.9628	0.26	1	58	4.8e-06	1	U	K.KITDEAYQIALTHIR.N
17106	627 – 641	591.3287	1770.9643	1770.9628	0.82	1	47	3.7e-05	1	U	K.KITDEAYQIALTHIR.N
17107	627 – 641	443.7484	1770.9646	1770.9628	1.04	1	54	9.4e-06	1	U	K.KITDEAYQIALTHIR.N
15537	628 – 641	822.4398	1642.8651	1642.8678	-1.68	0	64	1.1e-06	1	U	K.ITDEAYQIALTHIR.N
15538	628 – 641	548.6302	1642.8689	1642.8678	0.65	0	41	0.00015	1	U	K.ITDEAYQIALTHIR.N
15539	628 – 641	548.6309	1642.8709	1642.8678	1.88	0	39	0.00022	1	U	K.ITDEAYQIALTHIR.N
15540	628 – 641	548.6313	1642.8720	1642.8678	2.54	0	61	2e-06	1	U	K.ITDEAYQIALTHIR.N
22991	645 – 666	826.7604	2477.2595	2477.2537	2.33	1	85	9.9e-09	1	U	R.EAIDKIVDVLLETETVSGDEFR.T
19032	650 – 666	641.3311	1920.9715	1920.9680	1.81	0	71	2e-07	1	U	K.IVDVLLETETVSGDEFR.T
19033	650 – 666	961.4932	1920.9718	1920.9680	1.94	0	111	4.1e-11	1	U	K.IVDVLLETETVSGDEFR.T
15361	667 – 680	816.4364	1630.8582	1630.8566	0.97	0	67	5.7e-07	1	U	R.TLLAEFTEIPVENR.V
15362	667 – 680	544.6269	1630.8587	1630.8566	1.30	0	49	3e-05	1	U	R.TLLAEFTEIPVENR.V
23450	667 – 691	1298.2039	2594.3932	2594.3956	-0.92	1	61	2.1e-06	1	U	R.TLLAEFTEIPVENRVPAAAASPVTV.-
23451	667 – 691	865.8061	2594.3965	2594.3956	0.36	1	18	0.021	1	U	R.TLLAEFTEIPVENRVPAAAASPVTV.-
3472	681 – 691	491.7826	981.5507	981.5495	1.23	0	12	0.073	1	U	R.VPAAAASPVTV.-
3473	681 – 691	491.7826	981.5507	981.5495	1.25	0	15	0.041	1	U	R.VPAAAASPVTV.-

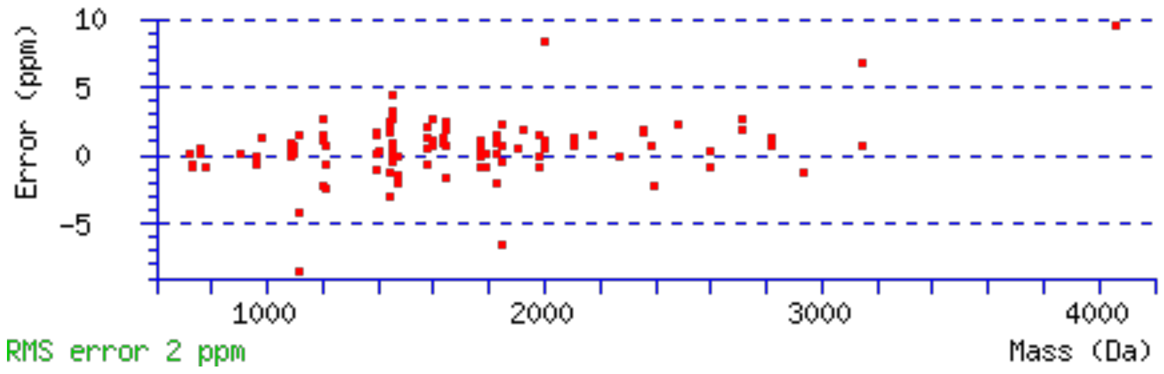
10
5
0
-5
Error (ppm)
1000
2000
3000
4000
RMS error 2 ppm
Mass (Da)

MASCOT Search Results

Protein View: tr|A0A0K9QED7|A0A0K9QED7_SPIOL

>tr|A0A0K9QED7|A0A0K9QED7_SPIOL Uncharacterized protein OS=Spinacia oleracea OX=3562 GN=SOVF_188660 PE=3 SV=1

**Database:**	Uni-Spinach
**Score:**	2548
**Nominal mass (M_r):**	82369
**Calculated pI:**	5.79

Sequence similarity is available as **an NCBI BLAST search of tr|A0A0K9QED7|A0A0K9QED7_SPIOL against nr**.

Search parameters

**MS data file:**	\\172.16.0.213\tank\windowsVM\Bill Cramer\052418\MGF\wc_QE_052418_Cramer_gel_4.mgf
**Enzyme:**	Trypsin: cuts C-term side of KR unless next residue is P.
**Fixed modifications:**	Carbamidomethyl (C)
**Variable modifications:**	Acetyl (K), Acetyl (Protein N-term), Deamidated (NQ), Oxidation (M)

Protein sequence coverage: 51%

Matched peptides shown in ***bold red***.

```
  1 MGLGSLTSRA MIRRASTILN TTRSHNFSLV RSIVSTPELK NTASAAAEAT
 51 PDPPLPPPRP PVNNARVHFP NPEDAIEVFV DGYSVKVPKG FTVLQACEVA
101 GVDIPRFCYH SRLSIAGNCR MCLVEVEKSP KPVASCAMPA LPGMKIKTDT
151 PIAKKAREGV MEFLLMNHPL DCPICDQGGE CDLQDQSMAF GSDRGRFTEM
201 KRSVVDKNLG PLVKTVMTRC IQCTRCVRFA SEVAGVEDLG ILGRGSGEEI
251 GTYVEQLMTS ELSGNVIDIC PVGALTSKPF AFKARNWELK GTESIDVTDA
301 VGSNIRIDSR GPEVMRITPR LNEDVNEEWI SDKTRFCYDG LKRQRLNDPM
351 IRGSDGRFKA VSWRDALDVI AEVMHKVKPE EIVGVAGKLS DAESMMALKD
401 FLNKMGSNNI WCEGNGGQPQ ADLRSGYLLN TGIADLETAD VFLLIGTQPR
451 VEAAMVNARI RKAVRSNHAK VGYIGPATDL NYDYEHLGTG PQTLQEIAEG
501 THSFFSAIKN AKNPAIVVGA GLFEREDKDA ILSIVETIAK SANVIRPDWN
551 GLNVLLLNAA QAASLDLGLV PESDKSIESA KFLYLMGADD VNMDKVPSDA
601 FVVYQGHHGD QGVYRANVIL PASAFSEKEG TYANTEGRAQ QTVPAVPTVG
651 DARDDWKIIR ALSETAGVNL PYDSLIDVRE RIRTVAPNLL SMDEREPATF
701 STLIKPEIKK EINPVPFKPS IENFYMTDSI TRASKIMAQC SSQLLKK
```

Unformatted sequence string: **747 residues** (for pasting into other applications).

Sort peptides by ◉ Residue Number ○ Increasing Mass ○ Decreasing Mass

Show predicted peptides also

Query	Start – End	Observed	Mr(expt)	Mr(calc)	ppm	M	Score	Expect	Rank	U	Peptide
23554	41 – 66	874.4514	2620.3325	2620.3357	-1.24	0	30	0.0014	1	U	K.NTASAAAEATPDPPLPPPRPPVNNAR.V
23557	41 – 66	874.4527	2620.3362	2620.3357	0.19	0	34	0.00058	1	U	K.NTASAAAEATPDPPLPPPRPPVNNAR.V
23558	41 – 66	874.4527	2620.3364	2620.3357	0.26	0	38	0.00028	1	U	K.NTASAAAEATPDPPLPPPRPPVNNAR.V
23559	41 – 66	874.4536	2620.3391	2620.3357	1.30	0	31	0.0011	1	U	K.NTASAAAEATPDPPLPPPRPPVNNAR.V
23561	41 – 66	874.4542	2620.3407	2620.3357	1.89	0	44	6.9e-05	1	U	K.NTASAAAEATPDPPLPPPRPPVNNAR.V
23565	41 – 66	874.7850	2621.3331	2621.3197	5.10	0	44	7.2e-05	1	U	K.NTASAAAEATPDPPLPPPRPPVNNAR.V + Deamidated (NQ)
23566	41 – 66	874.7864	2621.3373	2621.3197	6.70	0	32	0.00098	1	U	K.NTASAAAEATPDPPLPPPRPPVNNAR.V + Deamidated (NQ)
23567	41 – 66	874.7880	2621.3421	2621.3197	8.52	0	2	0.58	1	U	K.NTASAAAEATPDPPLPPPRPPVNNAR.V + Deamidated (NQ)
23573	41 – 66	875.1129	2622.3169	2622.3037	5.02	0	8	0.17	1	U	K.NTASAAAEATPDPPLPPPRPPVNNAR.V + 2 Deamidated (NQ)
23574	41 – 66	875.1130	2622.3173	2622.3037	5.16	0	22	0.0095	1	U	K.NTASAAAEATPDPPLPPPRPPVNNAR.V + 2 Deamidated (NQ)
23575	41 – 66	875.1136	2622.3191	2622.3037	5.86	0	5	0.32	1	U	K.NTASAAAEATPDPPLPPPRPPVNNAR.V + 2 Deamidated (NQ)
23576	41 – 66	875.1154	2622.3244	2622.3037	7.89	0	11	0.087	1	U	K.NTASAAAEATPDPPLPPPRPPVNNAR.V + 2 Deamidated (NQ)
21600	67 – 86	754.7087	2261.1044	2261.1005	1.75	0	66	6.5e-07	1	U	R.VHFPNPEDAIEVFVDGYSVK.V
17876	90 – 106	916.4737	1830.9328	1830.9298	1.64	0	37	0.00035	1	U	K.GFTVLQACEVAGVDIPR.F
16746	129 – 145	581.2957	1740.8653	1740.8725	-4.12	0	4	0.46	1	U	K.SPKPVASCAMPALPGMK.I
3509	146 – 154	493.7974	985.5802	985.5808	-0.64	1	27	0.044	1	U	K.IKTDTPIAK.K
3510	146 – 154	493.7975	985.5805	985.5808	-0.26	1	30	0.013	1	U	K.IKTDTPIAK.K

1087	197 – 202	406.2101	810.4056	810.4058	-0.29	1	23	0.0097	1	U	R.FTEMKR.S
11896	202 – 214	475.6247	1423.8523	1423.8511	0.82	2	2	0.71	1	U	K.RSVVDKNLGPLVK.T
8871	203 – 214	634.8821	1267.7497	1267.7500	-0.23	1	32	0.00093	1	U	R.SVVDKNLGPLVK.T
8872	203 – 214	634.8827	1267.7509	1267.7500	0.74	1	32	0.00099	1	U	R.SVVDKNLGPLVK.T
8873	203 – 214	423.5909	1267.7510	1267.7500	0.77	1	25	0.0047	1	U	R.SVVDKNLGPLVK.T
15369	229 – 244	816.9327	1631.8509	1631.8519	-0.60	0	89	4.8e-09	1	U	R.FASEVAGVEDLGILGR.G
15370	229 – 244	544.9584	1631.8534	1631.8519	0.91	0	50	2.6e-05	1	U	R.FASEVAGVEDLGILGR.G
15371	229 – 244	816.9344	1631.8542	1631.8519	1.42	0	99	5.7e-10	1	U	R.FASEVAGVEDLGILGR.G
21993	286 – 306	768.7221	2303.1445	2303.1393	2.25	1	71	2e-07	1	U	R.NWELKGTESIDVTDAVGSNIR.I
15378	291 – 306	817.4031	1632.7917	1632.7955	-2.32	0	108	2.4e-10	1	U	K.GTESIDVTDAVGSNIR.I
15379	291 – 306	817.4047	1632.7948	1632.7955	-0.46	0	94	1.5e-09	1	U	K.GTESIDVTDAVGSNIR.I
15380	291 – 306	545.2727	1632.7963	1632.7955	0.48	0	7	0.21	1	U	K.GTESIDVTDAVGSNIR.I
6653	307 – 316	387.2008	1158.5806	1158.5815	-0.83	1	4	0.54	1	U	R.IDSRGPEVMR.I
6654	307 – 316	387.2012	1158.5817	1158.5815	0.13	1	4	0.44	1	U	R.IDSRGPEVMR.I
6655	307 – 316	580.2982	1158.5818	1158.5815	0.19	1	8	0.26	1	U	R.IDSRGPEVMR.I
6993	307 – 316	588.2960	1174.5775	1174.5764	0.89	1	12	0.14	1	U	R.IDSRGPEVMR.I + Oxidation (M)
14820	321 – 333	795.8678	1589.7210	1589.7209	0.060	0	78	5.3e-08	1	U	R.LNEDVNEEWISDK.T
14821	321 – 333	795.8692	1589.7238	1589.7209	1.81	0	57	4.3e-06	1	U	R.LNEDVNEEWISDK.T
18100	321 – 335	924.4408	1846.8670	1846.8697	-1.43	1	94	1.7e-09	1	U	R.LNEDVNEEWISDKTR.F
18101	321 – 335	616.6314	1846.8724	1846.8697	1.45	1	65	8.4e-07	1	U	R.LNEDVNEEWISDKTR.F
18102	321 – 335	616.6317	1846.8732	1846.8697	1.88	1	7	0.2	1	U	R.LNEDVNEEWISDKTR.F
4614	336 – 343	353.5078	1057.5016	1057.5015	0.13	1	16	0.033	1	U	R.FCYDGLKR.Q
4615	336 – 343	529.7590	1057.5035	1057.5015	1.91	1	19	0.015	1	U	R.FCYDGLKR.Q
9767	343 – 352	439.2369	1314.6889	1314.6826	4.76	2	0	1.9	5	U	K.RQRLNDPMIR.G + Deamidated (NQ); Oxidation (M)
1776	346 – 352	429.7287	857.4428	857.4429	-0.12	0	26	0.059	1	U	R.LNDPMIR.G
2010	346 – 352	437.7254	873.4363	873.4378	-1.69	0	20	0.014	1	U	R.LNDPMIR.G + Oxidation (M)
2011	346 – 352	437.7259	873.4372	873.4378	-0.74	0	3	0.54	1	U	R.LNDPMIR.G + Oxidation (M)
10240	365 – 376	670.8486	1339.6826	1339.6806	1.54	0	85	5.3e-08	1	U	R.DALDVIAEVMHK.V
10241	365 – 376	447.5683	1339.6830	1339.6806	1.83	0	37	0.00035	1	U	R.DALDVIAEVMHK.V
10533	365 – 376	678.8442	1355.6738	1355.6755	-1.28	0	46	5.3e-05	1	U	R.DALDVIAEVMHK.V + Oxidation (M)
10534	365 – 376	678.8450	1355.6755	1355.6755	0.0052	0	11	0.095	1	U	R.DALDVIAEVMHK.V + Oxidation (M)
10535	365 – 376	452.8991	1355.6756	1355.6755	0.055	0	25	0.0049	1	U	R.DALDVIAEVMHK.V + Oxidation (M)
8072	377 – 388	613.3613	1224.7080	1224.7078	0.19	0	32	0.0011	1	U	K.VKPEEIVGVAGK.L
8073	377 – 388	613.3615	1224.7084	1224.7078	0.48	0	43	0.0001	1	U	K.VKPEEIVGVAGK.L
8075	377 – 388	409.2436	1224.7089	1224.7078	0.88	0	39	0.00022	1	U	K.VKPEEIVGVAGK.L
8076	377 – 388	613.3619	1224.7093	1224.7078	1.21	0	51	1.8e-05	1	U	K.VKPEEIVGVAGK.L
7401	389 – 399	598.2887	1194.5629	1194.5624	0.37	0	41	0.00013	1	U	K.LSDAESMMALK.D
7402	389 – 399	598.2894	1194.5643	1194.5624	1.59	0	12	0.078	1	U	K.LSDAESMMALK.D
17619	389 – 404	906.9483	1811.8821	1811.8797	1.31	1	99	5.7e-10	1	U	K.LSDAESMMALKDFLNK.M
17620	389 – 404	604.9683	1811.8831	1811.8797	1.85	1	86	9.5e-09	1	U	K.LSDAESMMALKDFLNK.M
17829	389 – 404	610.2978	1827.8716	1827.8746	-1.65	1	7	1.2	2	U	K.LSDAESMMALKDFLNK.M + Oxidation (M)
17830	389 – 404	610.2995	1827.8767	1827.8746	1.11	1	59	2.9e-06	1	U	K.LSDAESMMALKDFLNK.M + Oxidation (M)
17831	389 – 404	610.3002	1827.8789	1827.8746	2.32	1	35	0.00057	1	U	K.LSDAESMMALKDFLNK.M + Oxidation (M)
18053	389 – 404	615.6310	1843.8713	1843.8696	0.94	1	32	0.0011	1	U	K.LSDAESMMALKDFLNK.M + 2 Oxidation (M)
18054	389 – 404	615.6312	1843.8718	1843.8696	1.23	1	24	0.0054	1	U	K.LSDAESMMALKDFLNK.M + 2 Oxidation (M)
18055	389 – 404	615.6314	1843.8723	1843.8696	1.49	1	16	0.03	1	U	K.LSDAESMMALKDFLNK.M + 2 Oxidation (M)
3142	451 – 459	480.7503	959.4861	959.4858	0.24	0	40	0.00069	1	U	R.VEAAMVNAR.I
3143	451 – 459	480.7504	959.4863	959.4858	0.53	0	48	9e-05	1	U	R.VEAAMVNAR.I
3381	451 – 459	488.7476	975.4806	975.4807	-0.19	0	41	0.00036	1	U	R.VEAAMVNAR.I + Oxidation (M)
3382	451 – 459	488.7477	975.4809	975.4807	0.13	0	31	0.0011	1	U	R.VEAAMVNAR.I + Oxidation (M)
10287	513 – 525	671.8788	1341.7431	1341.7405	1.96	0	85	3.7e-08	1	U	K.NPAIVVGAGLFER.E
10288	513 – 525	671.8799	1341.7452	1341.7405	3.54	0	45	0.00011	1	U	K.NPAIVVGAGLFER.E
10289	513 – 525	448.2564	1341.7475	1341.7405	5.22	0	13	0.061	1	U	K.NPAIVVGAGLFER.E
15549	526 – 540	822.9568	1643.8990	1643.8981	0.52	1	69	3.5e-07	1	U	R.EDKDAILSIVETIAK.S
15550	526 – 540	548.9745	1643.9016	1643.8981	2.10	1	42	0.00011	1	U	R.EDKDAILSIVETIAK.S
10370	616 – 628	673.8697	1345.7249	1345.7241	0.54	0	65	9.1e-07	1	U	R.ANVILPASAFSEK.E
10372	616 – 628	673.8702	1345.7259	1345.7241	1.28	0	49	2.8e-05	1	U	R.ANVILPASAFSEK.E
22783	616 – 638	809.0718	2424.1936	2424.1921	0.64	1	62	1.6e-06	1	U	R.ANVILPASAFSEKEGTYANTEGR.A
22784	616 – 638	809.0721	2424.1946	2424.1921	1.05	1	77	6.1e-08	1	U	R.ANVILPASAFSEKEGTYANTEGR.A
5443	629 – 638	549.2467	1096.4788	1096.4785	0.25	0	24	0.0061	1	U	K.EGTYANTEGR.A
13573	639 – 653	755.4044	1508.7942	1508.7947	-0.38	0	53	9.7e-06	1	U	R.AQQTVPAVPTVGDAR.D
13574	639 – 653	755.4047	1508.7949	1508.7947	0.11	0	39	0.00022	1	U	R.AQQTVPAVPTVGDAR.D
13575	639 – 653	503.9392	1508.7959	1508.7947	0.77	0	16	0.037	1	U	R.AQQTVPAVPTVGDAR.D
13576	639 – 653	503.9395	1508.7967	1508.7947	1.29	0	20	0.015	1	U	R.AQQTVPAVPTVGDAR.D
20088	639 – 657	1027.5173	2053.0200	2053.0229	-1.39	1	39	0.00021	1	U	R.AQQTVPAVPTVGDARDDWK.I
20089	639 – 657	685.3488	2053.0247	2053.0229	0.89	1	30	0.0016	1	U	R.AQQTVPAVPTVGDARDDWK.I
20090	639 – 657	685.3492	2053.0257	2053.0229	1.38	1	43	9.6e-05	1	U	R.AQQTVPAVPTVGDARDDWK.I

2943	654 - 660	473.2592	944.5039	944.5080	-4.25	1	0	8.3	6	U	R.DDWKIIR.A
19940	661 - 679	678.3560	2032.0462	2032.0477	-0.71	0	88	1.6e-08	1	U	R.ALSETAGVNLPYDSLIDVR.E
19941	661 - 679	1017.0307	2032.0468	2032.0477	-0.43	0	85	1.1e-08	1	U	R.ALSETAGVNLPYDSLIDVR.E
19942	661 - 679	1017.0312	2032.0478	2032.0477	0.087	0	112	3.3e-11	1	U	R.ALSETAGVNLPYDSLIDVR.E
19943	661 - 679	678.3569	2032.0487	2032.0477	0.53	0	72	1.9e-07	1	U	R.ALSETAGVNLPYDSLIDVR.E
10334	684 - 695	673.3427	1344.6707	1344.6707	0.014	0	55	6.5e-06	1	U	R.TVAPNLLSMDER.E
10336	684 - 695	673.3448	1344.6751	1344.6707	3.28	0	38	0.0003	1	U	R.TVAPNLLSMDER.E
10672	684 - 695	681.3416	1360.6686	1360.6656	2.13	0	20	0.013	1	U	R.TVAPNLLSMDER.E + Oxidation (M)
24145	684 - 709	725.8903	2899.5322	2899.5365	-1.48	1	15	0.04	1	U	R.TVAPNLLSMDEREPATFSTLIKPEIK.K
24146	684 - 709	967.5188	2899.5347	2899.5365	-0.60	1	19	0.016	1	U	R.TVAPNLLSMDEREPATFSTLIKPEIK.K
24148	684 - 709	726.1426	2900.5415	2900.5205	7.24	1	6	0.26	1	U	R.TVAPNLLSMDEREPATFSTLIKPEIK.K + Deamidated (NQ)
24182	684 - 709	729.8910	2915.5349	2915.5314	1.22	1	10	0.11	1	U	R.TVAPNLLSMDEREPATFSTLIKPEIK.K + Oxidation (M)
24184	684 - 709	730.1403	2916.5322	2916.5154	5.76	1	15	0.036	1	U	R.TVAPNLLSMDEREPATFSTLIKPEIK.K + Deamidated (NQ); Oxidation (M)
24185	684 - 709	973.1858	2916.5355	2916.5154	6.91	1	19	0.018	1	U	R.TVAPNLLSMDEREPATFSTLIKPEIK.K + Deamidated (NQ); Oxidation (M)
24323	684 - 710	758.1682	3028.6435	3028.6154	9.27	2	0	0.95	1	U	R.TVAPNLLSMDEREPATFSTLIKPEIKK.E + Deamidated (NQ)
14597	696 - 709	525.3001	1572.8783	1572.8763	1.30	0	46	5.2e-05	1	U	R.EPATFSTLIKPEIK.K
23882	710 - 732	909.8005	2726.3798	2726.3625	6.33	1	4	0.4	1	U	K.KEINPVPFKPSIENFYMTDSITR.A + Deamidated (NQ)
23883	710 - 732	682.6030	2726.3830	2726.3625	7.51	1	26	0.0038	1	U	K.KEINPVPFKPSIENFYMTDSITR.A + Deamidated (NQ)
23914	710 - 732	686.6028	2742.3823	2742.3574	9.06	1	3	0.56	1	U	K.KEINPVPFKPSIENFYMTDSITR.A + Deamidated (NQ); Oxidation (M)
23462	711 - 732	1300.1479	2598.2813	2598.2676	5.30	0	43	9.9e-05	1	U	K.EINPVPFKPSIENFYMTDSITR.A + Deamidated (NQ)
23463	711 - 732	867.1027	2598.2862	2598.2676	7.16	0	40	0.00017	1	U	K.EINPVPFKPSIENFYMTDSITR.A + Deamidated (NQ)
23464	711 - 732	867.1029	2598.2869	2598.2676	7.44	0	32	0.00092	1	U	K.EINPVPFKPSIENFYMTDSITR.A + Deamidated (NQ)
23529	711 - 732	872.1010	2613.2811	2613.2785	1.01	0	31	0.0012	1	U	K.EINPVPFKPSIENFYMTDSITR.A + Oxidation (M)
9079	736 - 746	639.8322	1277.6498	1277.6472	2.06	0	47	3.9e-05	1	U	K.IMAQCSSQLLK.K

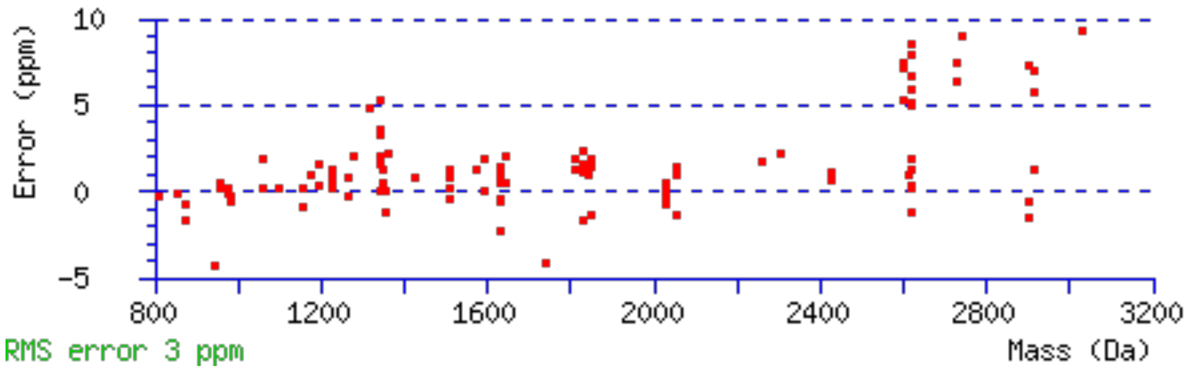

Mascot: http://www.matrixscience.com/

MASCOT Search Results

Protein View: tr|A0A0K9R886|A0A0K9R886_SPIOL

>tr|A0A0K9R886|A0A0K9R886_SPIOL Uncharacterized protein OS=Spinacia oleracea OX=3562 GN=SOVF_102250 PE=4 SV=1

**Database:**	Uni-Spinach
**Score:**	2135
**Nominal mass (M_r):**	23145
**Calculated pI:**	9.76

Sequence similarity is available as an NCBI BLAST search of tr|A0A0K9R886|A0A0K9R886_SPIOL against nr.

Search parameters

**MS data file:**	\\172.16.0.213\tank\windowsVM\Bill Cramer\052418\MGF\wc_QE_052418_Cramer_gel_4.mgf
**Enzyme:**	Trypsin: cuts C-term side of KR unless next residue is P.
**Fixed modifications:**	Carbamidomethyl (C)
**Variable modifications:**	Acetyl (K), Acetyl (Protein N-term), Deamidated (NQ), Oxidation (M)

Protein sequence coverage: 70%

Matched peptides shown in ***bold red***.

```
  1 MAMATQATLF SPSSLSSAKP IDTRLTTSFK QPSAVTFASK PASRHHSIRA
 51 AAAAEGKAAA ATETKEAPKG FTPPELDPNT PSPIFAGSTG GLLRKAQVEE
101 FYVITWESPK EQIFEMPTGG AAIMREGPNL LKLARKEQCL ALGTRLRSKY
151 KIKYQFYRVF PSGEVQYLHP KDGVYPEKVN PGRQGVGLNM RSIGKNVSPI
201 EVKFTGKQPY DL
```

Unformatted sequence string: 212 residues (for pasting into other applications).

Sort peptides by ◉ Residue Number ○ Increasing Mass ○ Decreasing Mass

Show predicted peptides also

Query	Start – End	Observed	Mr(expt)	Mr(calc)	ppm	M	Score	Expect	Rank	U	Peptide
7220	58 – 69	594.3149	1186.6153	1186.6193	-3.43	1	46	8.9e-05	1	U	K.AAAATETKEAPK.G
24831	58 – 94	928.4812	3709.8957	3709.8839	3.18	2	22	0.0085	1	U	K.AAAATETKEAPKGFTPPELDPNTPSPIFAGSTGGLLR.K + Deamidated (NQ)
24254	66 – 94	989.8489	2966.5250	2966.5025	7.56	1	35	0.00057	1	U	K.EAPKGFTPPELDPNTPSPIFAGSTGGLLR.K + Deamidated (NQ)
23240	70 – 94	847.7709	2540.2908	2540.2911	-0.14	0	56	6.1e-06	1	U	K.GFTPPELDPNTPSPIFAGSTGGLLR.K
23241	70 – 94	1271.1529	2540.2913	2540.2911	0.080	0	68	4.5e-07	1	U	K.GFTPPELDPNTPSPIFAGSTGGLLR.K
23245	70 – 94	636.3286	2541.2851	2541.2751	3.92	0	51	1.7e-05	1	U	K.GFTPPELDPNTPSPIFAGSTGGLLR.K + Deamidated (NQ)
23246	70 – 94	848.1041	2541.2905	2541.2751	6.07	0	51	1.8e-05	1	U	K.GFTPPELDPNTPSPIFAGSTGGLLR.K + Deamidated (NQ)
23731	70 – 95	890.4682	2668.3826	2668.3861	-1.29	1	29	0.0019	1	U	K.GFTPPELDPNTPSPIFAGSTGGLLRK.A
23733	70 – 95	890.4707	2668.3904	2668.3861	1.63	1	68	4.6e-07	1	U	K.GFTPPELDPNTPSPIFAGSTGGLLRK.A
23735	70 – 95	668.1060	2668.3949	2668.3861	3.32	1	31	0.0012	1	U	K.GFTPPELDPNTPSPIFAGSTGGLLRK.A
23736	70 – 95	668.3542	2669.3876	2669.3701	6.58	1	40	0.00018	1	U	K.GFTPPELDPNTPSPIFAGSTGGLLRK.A + Deamidated (NQ)
19362	95 – 110	977.5014	1952.9883	1952.9884	-0.027	1	117	1e-11	1	U	R.KAQVEEFYVITWESPK.E
19363	95 – 110	652.0038	1952.9897	1952.9884	0.68	1	57	4.7e-06	1	U	R.KAQVEEFYVITWESPK.E
19364	95 – 110	652.0042	1952.9908	1952.9884	1.27	1	58	4e-06	1	U	R.KAQVEEFYVITWESPK.E
19373	95 – 110	978.0014	1953.9883	1953.9724	8.13	1	64	1.1e-06	1	U	R.KAQVEEFYVITWESPK.E + Deamidated (NQ)
24776	95 – 125	897.4507	3585.7736	3585.7523	5.94	2	31	0.0014	1	U	R.KAQVEEFYVITWESPKEQIFEMPTGGAAIMR.E + Deamidated (NQ)
24780	95 – 125	901.1992	3600.7676	3600.7632	1.21	2	36	0.00041	1	U	R.KAQVEEFYVITWESPKEQIFEMPTGGAAIMR.E + Oxidation (M)
24786	95 – 125	905.1981	3616.7631	3616.7582	1.38	2	15	0.042	1	U	R.KAQVEEFYVITWESPKEQIFEMPTGGAAIMR.E + 2 Oxidation (M)
17771	96 – 110	913.4562	1824.8978	1824.8934	2.39	0	102	2.9e-10	1	U	K.AQVEEFYVITWESPK.E
17772	96 – 110	609.3074	1824.9004	1824.8934	3.83	0	66	6.5e-07	1	U	K.AQVEEFYVITWESPK.E
15624	111 – 125	825.9028	1649.7910	1649.7905	0.28	0	51	2.1e-05	1	U	K.EQIFEMPTGGAAIMR.E
15625	111 – 125	550.9379	1649.7920	1649.7905	0.88	0	49	2.4e-05	1	U	K.EQIFEMPTGGAAIMR.E
15626	111 – 125	825.9033	1649.7920	1649.7905	0.91	0	61	2.4e-06	1	U	K.EQIFEMPTGGAAIMR.E
15627	111 – 125	825.9042	1649.7938	1649.7905	1.97	0	65	1.2e-06	1	U	K.EQIFEMPTGGAAIMR.E
15628	111 – 125	550.9386	1649.7939	1649.7905	2.04	0	44	7.8e-05	1	U	K.EQIFEMPTGGAAIMR.E
15634	111 – 125	826.3869	1650.7593	1650.7745	-9.26	0	2	0.68	1	U	K.EQIFEMPTGGAAIMR.E + Deamidated (NQ)
15635	111 – 125	826.3958	1650.7771	1650.7745	1.58	0	23	0.0065	1	U	K.EQIFEMPTGGAAIMR.E + Deamidated (NQ)
15848	111 – 125	833.8978	1665.7810	1665.7854	-2.64	0	13	0.059	1	U	K.EQIFEMPTGGAAIMR.E + Oxidation (M)
15849	111 – 125	833.8987	1665.7828	1665.7854	-1.60	0	24	0.0051	1	U	K.EQIFEMPTGGAAIMR.E + Oxidation (M)
15850	111 – 125	833.8994	1665.7842	1665.7854	-0.76	0	73	1.6e-07	1	U	K.EQIFEMPTGGAAIMR.E + Oxidation (M)
15853	111 – 125	833.9003	1665.7860	1665.7854	0.32	0	23	0.0063	1	U	K.EQIFEMPTGGAAIMR.E + Oxidation (M)
15855	111 – 125	556.2693	1665.7862	1665.7854	0.46	0	34	0.0006	1	U	K.EQIFEMPTGGAAIMR.E + Oxidation (M)
15856	111 – 125	833.9006	1665.7866	1665.7854	0.67	0	65	7.5e-06	1	U	K.EQIFEMPTGGAAIMR.E + Oxidation (M)
15857	111 – 125	833.9006	1665.7866	1665.7854	0.71	0	42	0.00011	1	U	K.EQIFEMPTGGAAIMR.E + Oxidation (M)

15858	111 - 125	556.2698	1665.7875	1665.7854	1.26	0	27	0.003	1	U	K.EQIFEMPTGGAAIMR.E + Oxidation (M)
15859	111 - 125	833.9011	1665.7877	1665.7854	1.35	0	13	0.056	1	U	K.EQIFEMPTGGAAIMR.E + Oxidation (M)
15860	111 - 125	556.2699	1665.7880	1665.7854	1.51	0	49	2.4e-05	1	U	K.EQIFEMPTGGAAIMR.E + Oxidation (M)
15861	111 - 125	833.9013	1665.7881	1665.7854	1.57	0	18	0.022	1	U	K.EQIFEMPTGGAAIMR.E + Oxidation (M)
15864	111 - 125	833.9030	1665.7914	1665.7854	3.55	0	31	0.0012	1	U	K.EQIFEMPTGGAAIMR.E + Oxidation (M)
16055	111 - 125	841.8980	1681.7815	1681.7804	0.69	0	55	6.6e-06	1	U	K.EQIFEMPTGGAAIMR.E + 2 Oxidation (M)
16056	111 - 125	841.8981	1681.7817	1681.7804	0.82	0	29	0.0018	1	U	K.EQIFEMPTGGAAIMR.E + 2 Oxidation (M)
16057	111 - 125	561.6013	1681.7821	1681.7804	1.05	0	2	0.61	1	U	K.EQIFEMPTGGAAIMR.E + 2 Oxidation (M)
16058	111 - 125	841.8987	1681.7829	1681.7804	1.52	0	40	0.00019	1	U	K.EQIFEMPTGGAAIMR.E + 2 Oxidation (M)
16059	111 - 125	561.6016	1681.7829	1681.7804	1.53	0	33	0.00074	1	U	K.EQIFEMPTGGAAIMR.E + 2 Oxidation (M)
16060	111 - 125	841.8988	1681.7830	1681.7804	1.57	0	14	0.048	1	U	K.EQIFEMPTGGAAIMR.E + 2 Oxidation (M)
16062	111 - 125	841.9030	1681.7915	1681.7804	6.64	0	11	0.09	1	U	K.EQIFEMPTGGAAIMR.E + 2 Oxidation (M)
613	126 - 132	385.7237	769.4329	769.4334	-0.60	0	21	0.087	1	U	R.EGPNLLK.L
614	126 - 132	385.7239	769.4332	769.4334	-0.19	0	15	0.21	1	U	R.EGPNLLK.L
615	126 - 132	385.7240	769.4334	769.4334	0.018	0	7	0.28	1	U	R.EGPNLLK.L
616	126 - 132	385.7241	769.4336	769.4334	0.27	0	1	0.77	1	U	R.EGPNLLK.L
617	126 - 132	385.7243	769.4341	769.4334	0.90	0	34	0.005	1	U	R.EGPNLLK.L
618	126 - 132	385.7244	769.4343	769.4334	1.22	0	21	0.094	1	U	R.EGPNLLK.L
619	126 - 132	385.7245	769.4345	769.4334	1.46	0	30	0.012	1	U	R.EGPNLLK.L
634	126 - 132	386.2163	770.4181	770.4174	0.88	0	13	0.73	2	U	R.EGPNLLK.L + Deamidated (NQ)
6521	126 - 135	576.8406	1151.6666	1151.6662	0.30	1	26	0.004	1	U	R.EGPNLLKLAR.K + Acetyl (K)
6997	136 - 145	392.5443	1174.6112	1174.6128	-1.39	1	20	0.012	1	U	R.KEQCLALGTR.L
6998	136 - 145	588.3134	1174.6122	1174.6128	-0.51	1	57	1.7e-05	1	U	R.KEQCLALGTR.L
6999	136 - 145	392.5448	1174.6126	1174.6128	-0.16	1	27	0.0027	1	U	R.KEQCLALGTR.L
7897	136 - 145	609.3206	1216.6267	1216.6234	2.76	1	6	0.25	1	U	R.KEQCLALGTR.L + Acetyl (K)
4387	137 - 145	524.2659	1046.5173	1046.5179	-0.54	0	16	0.078	1	U	K.EQCLALGTR.L
3925	152 - 158	509.2796	1016.5447	1016.5443	0.35	1	38	0.0034	1	U	K.IKYQFYR.V
3926	152 - 158	509.2804	1016.5462	1016.5443	1.88	1	6	0.26	1	U	K.IKYQFYR.V
684	154 - 158	388.6886	775.3627	775.3653	-3.36	0	18	0.14	1	U	K.YQFYR.V
685	154 - 158	388.6898	775.3651	775.3653	-0.27	0	22	0.049	1	U	K.YQFYR.V
686	154 - 158	388.6899	775.3653	775.3653	-0.043	0	17	0.12	1	U	K.YQFYR.V
687	154 - 158	388.6899	775.3653	775.3653	-0.025	0	22	0.051	1	U	K.YQFYR.V
688	154 - 158	388.6901	775.3656	775.3653	0.35	0	16	0.1	1	U	K.YQFYR.V
689	154 - 158	388.6902	775.3659	775.3653	0.70	0	14	0.18	1	U	K.YQFYR.V
690	154 - 158	388.6902	775.3659	775.3653	0.73	0	13	0.19	1	U	K.YQFYR.V
691	154 - 158	388.6904	775.3663	775.3653	1.29	0	1	0.82	1	U	K.YQFYR.V
692	154 - 158	388.6905	775.3664	775.3653	1.40	0	3	0.58	1	U	K.YQFYR.V
693	154 - 158	388.6905	775.3665	775.3653	1.52	0	17	0.062	1	U	K.YQFYR.V
13394	159 - 171	750.8946	1499.7746	1499.7773	-1.77	0	41	0.00014	1	U	R.VFPSGEVQYLHPK.D
13395	159 - 171	750.8952	1499.7759	1499.7773	-0.93	0	38	0.00025	1	U	R.VFPSGEVQYLHPK.D
13396	159 - 171	750.8954	1499.7763	1499.7773	-0.65	0	42	0.00012	1	U	R.VFPSGEVQYLHPK.D
13397	159 - 171	750.8956	1499.7767	1499.7773	-0.37	0	48	3.2e-05	1	U	R.VFPSGEVQYLHPK.D
13398	159 - 171	750.8956	1499.7767	1499.7773	-0.37	0	48	3.2e-05	1	U	R.VFPSGEVQYLHPK.D
13399	159 - 171	750.8957	1499.7769	1499.7773	-0.25	0	41	0.00015	1	U	R.VFPSGEVQYLHPK.D
13400	159 - 171	750.8960	1499.7775	1499.7773	0.18	0	40	0.00017	1	U	R.VFPSGEVQYLHPK.D
13401	159 - 171	750.8962	1499.7778	1499.7773	0.38	0	46	4.6e-05	1	U	R.VFPSGEVQYLHPK.D
13402	159 - 171	500.9332	1499.7779	1499.7773	0.42	0	37	0.00033	1	U	R.VFPSGEVQYLHPK.D
13403	159 - 171	750.8964	1499.7782	1499.7773	0.60	0	48	3.2e-05	1	U	R.VFPSGEVQYLHPK.D
13404	159 - 171	500.9335	1499.7786	1499.7773	0.90	0	25	0.0041	1	U	R.VFPSGEVQYLHPK.D
13405	159 - 171	500.9335	1499.7788	1499.7773	1.03	0	37	0.00033	1	U	R.VFPSGEVQYLHPK.D
13406	159 - 171	500.9336	1499.7790	1499.7773	1.14	0	28	0.0024	1	U	R.VFPSGEVQYLHPK.D
13407	159 - 171	500.9336	1499.7790	1499.7773	1.17	0	25	0.005	1	U	R.VFPSGEVQYLHPK.D
13408	159 - 171	500.9337	1499.7791	1499.7773	1.25	0	33	0.00078	1	U	R.VFPSGEVQYLHPK.D
13409	159 - 171	500.9338	1499.7796	1499.7773	1.55	0	35	0.00052	1	U	R.VFPSGEVQYLHPK.D
13410	159 - 171	500.9340	1499.7802	1499.7773	1.97	0	37	0.00033	1	U	R.VFPSGEVQYLHPK.D
13411	159 - 171	750.8975	1499.7805	1499.7773	2.14	0	28	0.0026	1	U	R.VFPSGEVQYLHPK.D
13412	159 - 171	500.9342	1499.7808	1499.7773	2.35	0	37	0.00032	1	U	R.VFPSGEVQYLHPK.D
13413	159 - 171	500.9343	1499.7810	1499.7773	2.47	0	35	0.00048	1	U	R.VFPSGEVQYLHPK.D
13414	159 - 171	500.9344	1499.7813	1499.7773	2.68	0	37	0.00032	1	U	R.VFPSGEVQYLHPK.D
13415	159 - 171	500.9345	1499.7816	1499.7773	2.86	0	37	0.00032	1	U	R.VFPSGEVQYLHPK.D
13416	159 - 171	500.9345	1499.7817	1499.7773	2.95	0	37	0.00033	1	U	R.VFPSGEVQYLHPK.D
1033	172 - 178	404.1975	806.3804	806.3810	-0.81	0	6	0.3	1	U	K.DGVYPEK.V
1034	172 - 178	404.1977	806.3809	806.3810	-0.20	0	14	0.066	1	U	K.DGVYPEK.V
10071	172 - 183	665.8402	1329.6659	1329.6677	-1.34	1	21	0.011	1	U	K.DGVYPEKVNPGR.Q
10072	172 - 183	444.2294	1329.6663	1329.6677	-1.07	1	26	0.0034	1	U	K.DGVYPEKVNPGR.Q
10073	172 - 183	665.8411	1329.6676	1329.6677	-0.10	1	44	8.2e-05	1	U	K.DGVYPEKVNPGR.Q
10074	172 - 183	444.2300	1329.6682	1329.6677	0.38	1	33	0.00075	1	U	K.DGVYPEKVNPGR.Q
20987	172 - 191	547.2856	2185.1134	2185.1062	3.28	2	19	0.017	1	U	K.DGVYPEKVNPGRQGVGLNMR.S
11370	179 - 191	699.3751	1396.7356	1396.7357	-0.13	1	19	0.018	1	U	K.VNPGRQGVGLNMR.S
11371	179 - 191	466.5859	1396.7359	1396.7357	0.12	1	6	0.25	1	U	K.VNPGRQGVGLNMR.S
2012	184 - 191	437.7314	873.4482	873.4491	-1.03	0	37	0.0015	1	U	R.QGVGLNMR.S
2013	184 - 191	437.7315	873.4485	873.4491	-0.69	0	34	0.0009	1	U	R.QGVGLNMR.S
2014	184 - 191	437.7319	873.4493	873.4491	0.32	0	40	0.00079	1	U	R.QGVGLNMR.S
2015	184 - 191	437.7321	873.4496	873.4491	0.57	0	52	0.00011	1	U	R.QGVGLNMR.S
2016	184 - 191	437.7323	873.4500	873.4491	1.06	0	31	0.019	1	U	R.QGVGLNMR.S
2017	184 - 191	437.7325	873.4505	873.4491	1.66	0	45	0.00036	1	U	R.QGVGLNMR.S
2045	184 - 191	438.2229	874.4313	874.4331	-2.02	0	3	0.47	1	U	R.QGVGLNMR.S + Deamidated (NQ)
2046	184 - 191	438.2235	874.4324	874.4331	-0.73	0	23	0.067	1	U	R.QGVGLNMR.S + Deamidated (NQ)
2047	184 - 191	438.2238	874.4331	874.4331	0.049	0	18	0.32	1	U	R.QGVGLNMR.S + Deamidated (NQ)
2203	184 - 191	445.7275	889.4405	889.4440	-3.96	0	3	0.57	1	U	R.QGVGLNMR.S + Oxidation (M)
2204	184 - 191	445.7287	889.4429	889.4440	-1.18	0	17	0.028	1	U	R.QGVGLNMR.S + Oxidation (M)
2205	184 - 191	445.7292	889.4438	889.4440	-0.20	0	29	0.0019	1	U	R.QGVGLNMR.S + Oxidation (M)
2206	184 - 191	445.7297	889.4449	889.4440	1.02	0	36	0.0016	1	U	R.QGVGLNMR.S + Oxidation (M)

2217	184 - 191	446.2200	890.4254	890.4280	-2.86	0	13	0.058	1	U	R.QGVGLNMR.S + Deamidated (NQ); Oxidation (M)
9820	184 - 195	659.3496	1316.6847	1316.6871	-1.80	1	0	2.4	2	U	R.QGVGLNMRSIGK.N + Acetyl (K); Oxidation (M)
8909	192 - 203	424.2502	1269.7288	1269.7292	-0.33	1	21	0.044	1	U	R.SIGKNVSPIEVK.F
8911	192 - 203	635.8728	1269.7311	1269.7292	1.43	1	35	0.0014	1	U	R.SIGKNVSPIEVK.F
2179	196 - 203	443.2554	884.4963	884.4967	-0.46	0	9	0.14	1	U	K.NVSPIEVK.F
2180	196 - 203	443.2555	884.4965	884.4967	-0.22	0	4	0.39	1	U	K.NVSPIEVK.F
2182	196 - 203	443.2559	884.4973	884.4967	0.71	0	16	0.2	1	U	K.NVSPIEVK.F
2183	196 - 203	443.2561	884.4976	884.4967	1.02	0	25	0.07	1	U	K.NVSPIEVK.F
2184	196 - 203	443.2564	884.4983	884.4967	1.78	0	28	0.031	1	U	K.NVSPIEVK.F
2189	196 - 203	443.7459	885.4772	885.4807	-4.03	0	15	0.5	1	U	K.NVSPIEVK.F + Deamidated (NQ)
2190	196 - 203	443.7482	885.4818	885.4807	1.25	0	15	0.32	1	U	K.NVSPIEVK.F + Deamidated (NQ)
9863	196 - 207	659.8729	1317.7312	1317.7292	1.46	1	25	0.005	1	U	K.NVSPIEVKFTGK.Q
4829	204 - 212	534.7724	1067.5302	1067.5288	1.31	1	22	0.008	1	U	K.FTGKQPYDL.-
4831	204 - 212	534.7732	1067.5319	1067.5288	2.97	1	19	0.016	1	U	K.FTGKQPYDL.-

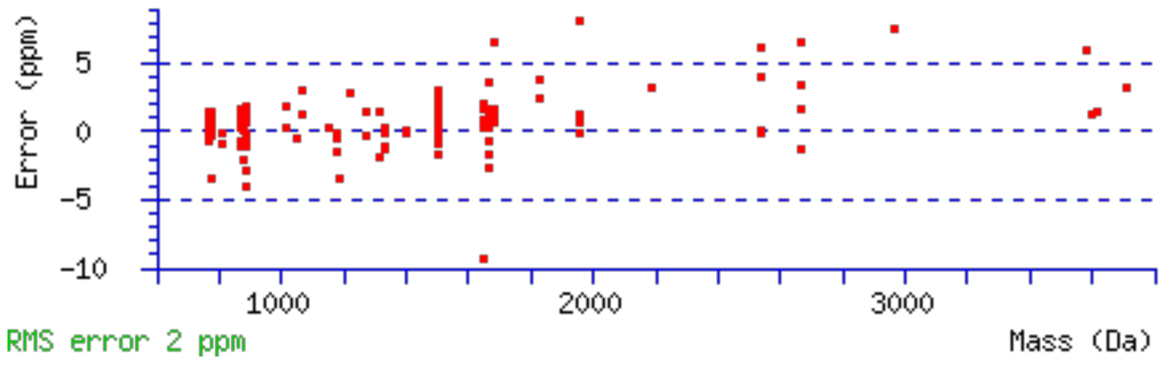

MASCOT Search Results

Protein View: tr|A0A0K9QKT8|A0A0K9QKT8_SPIOL

>tr|A0A0K9QKT8|A0A0K9QKT8_SPIOL Uncharacterized protein OS=Spinacia oleracea OX=3562 GN=SOVF_167830 PE=4 SV=1

**Database:**	Uni-Spinach
**Score:**	1892
**Nominal mass (M_r):**	40479
**Calculated pI:**	6.17

Sequence similarity is available as an NCBI BLAST search of tr|A0A0K9QKT8|A0A0K9QKT8_SPIOL against nr.

Search parameters

**MS data file:**	\\172.16.0.213\tank\windowsVM\Bill Cramer\052418\MGF\wc_QE_052418_Cramer_gel_4.mgf
**Enzyme:**	Trypsin: cuts C-term side of KR unless next residue is P.
**Fixed modifications:**	Carbamidomethyl (C)
**Variable modifications:**	Acetyl (K), Acetyl (Protein N-term), Deamidated (NQ), Oxidation (M)

Protein sequence coverage: 61%

Matched peptides shown in ***bold red***.

```
  1 MASLLPFSLL KPTTICKASS TAAPPAALVE SLNDQFARKG INFLESTDTS
 51 TNISSPIVEL SVRNGSSLKL QLSNAHVTSY KPKVYWKDDG FEEVLYTLPL
101 SKGGIGLVLN DVTQPVTTTK KPDPFRRSEP AAAAAAKGSL LAGAEWSVRD
151 VDSDSFDAVQ VELSCTSGSL EITYVVSLYP ESMASAVLVK NNGNKAIGLT
201 SAILSHIQFK KRSGSGIQGL QGCSYCSHPP LSSPFEIVSP AEAMKAEDPG
251 MFSFSSESPS KLGEWTTQEV PITILKNKLS RVYTAPPSER SKKFYRTTPS
301 KYETVDQGRE LVFRVIRMGY DDILVSSPGS LSEKYGRDYF ICTGPASILV
351 PVTVNPGEEW RGAQVIEHDN LT
```

Unformatted sequence string: 372 residues (for pasting into other applications).

Sort peptides by ◉ Residue Number ○ Increasing Mass ○ Decreasing Mass

Show predicted peptides also

Query	Start – End	Observed	Mr(expt)	Mr(calc)	ppm	M	Score	Expect	Rank	U	Peptide
20557	18 – 38	1058.5378	2115.0610	2115.0596	0.65	0	86	9e-09	1	U	K.ASSTAAPPAALVESLNDQFAR.K
20558	18 – 38	706.0283	2115.0631	2115.0596	1.62	0	88	6.2e-09	1	U	K.ASSTAAPPAALVESLNDQFAR.K
20559	18 – 38	706.0283	2115.0632	2115.0596	1.68	0	92	2.2e-09	1	U	K.ASSTAAPPAALVESLNDQFAR.K
20560	18 – 38	1058.5395	2115.0645	2115.0596	2.31	0	74	1.3e-07	1	U	K.ASSTAAPPAALVESLNDQFAR.K
20561	18 – 38	1058.5402	2115.0658	2115.0596	2.91	0	76	6.7e-08	1	U	K.ASSTAAPPAALVESLNDQFAR.K
20562	18 – 38	706.0293	2115.0662	2115.0596	3.11	0	88	5.8e-09	1	U	K.ASSTAAPPAALVESLNDQFAR.K
23816	39 – 63	903.1448	2706.4124	2706.4076	1.80	1	85	1.1e-08	1	U	R.KGINFLESTDTSTNISSPIVELSVR.N
23392	40 – 63	860.4458	2578.3155	2578.3126	1.10	0	75	8.7e-08	1	U	K.GINFLESTDTSTNISSPIVELSVR.N
14762	70 – 83	793.4390	1584.8635	1584.8624	0.72	0	49	2.5e-05	1	U	K.LQLSNAHVTSYKPK.V
14763	70 – 83	529.2954	1584.8644	1584.8624	1.31	0	48	2.9e-05	1	U	K.LQLSNAHVTSYKPK.V
14765	70 – 83	397.2236	1584.8655	1584.8624	1.97	0	37	0.00031	1	U	K.LQLSNAHVTSYKPK.V
14778	70 – 83	529.6236	1585.8489	1585.8464	1.57	0	28	0.0022	1	U	K.LQLSNAHVTSYKPK.V + Deamidated (NQ)
21971	84 – 102	768.0591	2301.1555	2301.1569	-0.59	1	39	0.00021	1	U	K.VYWKDDGFEEVLYTLPLSK.G
16557	88 – 102	863.4334	1724.8523	1724.8509	0.83	0	60	2.6e-06	1	U	K.DDGFEEVLYTLPLSK.G
16558	88 – 102	575.9581	1724.8525	1724.8509	0.92	0	17	0.028	1	U	K.DDGFEEVLYTLPLSK.G
17627	103 – 120	907.0063	1811.9980	1811.9993	-0.74	0	76	5e-07	1	U	K.GGIGLVLNDVTQPVTTTK.K
17628	103 – 120	605.0069	1811.9989	1811.9993	-0.23	0	43	8.5e-05	1	U	K.GGIGLVLNDVTQPVTTTK.K
17629	103 – 120	605.0071	1811.9993	1811.9993	0.020	0	34	0.00059	1	U	K.GGIGLVLNDVTQPVTTTK.K
17630	103 – 120	907.0085	1812.0024	1811.9993	1.73	0	58	3.9e-06	1	U	K.GGIGLVLNDVTQPVTTTK.K
17643	103 – 120	907.4987	1812.9829	1812.9833	-0.24	0	30	0.0014	1	U	K.GGIGLVLNDVTQPVTTTK.K + Deamidated (NQ)
17645	103 – 120	907.5009	1812.9872	1812.9833	2.15	0	46	4.5e-05	1	U	K.GGIGLVLNDVTQPVTTTK.K + Deamidated (NQ)
17664	103 – 120	907.9995	1813.9845	1813.9673	9.45	0	6	0.3	1	U	K.GGIGLVLNDVTQPVTTTK.K + 2 Deamidated (NQ)
487	121 – 126	380.2106	758.4066	758.4075	-1.19	0	29	0.0043	1	U	K.KPDPFR.R
488	121 – 126	380.2109	758.4073	758.4075	-0.31	0	28	0.0033	1	U	K.KPDPFR.R
489	121 – 126	380.2110	758.4075	758.4075	-0.032	0	30	0.016	1	U	K.KPDPFR.R

2564	121 - 127	458.2610	914.5075	914.5086	-1.23	1	6	0.29	1	U	K.KPDPFRR.S
2565	121 - 127	458.2613	914.5080	914.5086	-0.71	1	16	0.032	1	U	K.KPDPFRR.S
2566	121 - 127	458.2617	914.5088	914.5086	0.24	1	6	0.27	1	U	K.KPDPFRR.S
4265	127 - 137	521.7858	1041.5570	1041.5567	0.34	1	14	0.045	1	U	R.RSEPAAAAAAK.G
8483	138 - 149	623.3329	1244.6513	1244.6513	0.0024	0	42	0.00012	1	U	K.GSLLAGAEWSVR.D
8484	138 - 149	623.3337	1244.6528	1244.6513	1.18	0	60	1.2e-05	1	U	K.GSLLAGAEWSVR.D
14940	196 - 210	799.9666	1597.9186	1597.9192	-0.35	0	104	1.7e-10	1	U	K.AIGLTSAILSHIQFK.K
14941	196 - 210	533.6473	1597.9200	1597.9192	0.51	0	45	5.6e-05	1	U	K.AIGLTSAILSHIQFK.K
16266	246 - 261	851.8666	1701.7186	1701.7192	-0.35	0	75	8.8e-08	1	U	K.AEDPGMFSFSSESPSK.L
16267	246 - 261	851.8669	1701.7192	1701.7192	-0.032	0	74	1.2e-07	1	U	K.AEDPGMFSFSSESPSK.L
16469	246 - 261	859.8647	1717.7148	1717.7141	0.37	0	79	3.5e-08	1	U	K.AEDPGMFSFSSESPSK.L + Oxidation (M)
16470	246 - 261	859.8657	1717.7168	1717.7141	1.55	0	57	4.7e-06	1	U	K.AEDPGMFSFSSESPSK.L + Oxidation (M)
16471	246 - 261	859.8674	1717.7203	1717.7141	3.59	0	51	1.6e-05	1	U	K.AEDPGMFSFSSESPSK.L + Oxidation (M)
16585	262 - 276	864.4834	1726.9522	1726.9505	0.97	0	44	8.1e-05	1	U	K.LGEWTTQEVPITILK.N
16586	262 - 276	864.4834	1726.9523	1726.9505	1.00	0	66	6.8e-07	1	U	K.LGEWTTQEVPITILK.N
16587	262 - 276	576.6589	1726.9547	1726.9505	2.43	0	27	0.0034	1	U	K.LGEWTTQEVPITILK.N
3945	282 - 290	510.2611	1018.5077	1018.5083	-0.66	0	22	0.0092	1	U	R.VYTAPPSER.S
3946	282 - 290	510.2614	1018.5083	1018.5083	-0.017	0	23	0.0071	1	U	R.VYTAPPSER.S
3947	282 - 290	510.2616	1018.5086	1018.5083	0.28	0	17	0.025	1	U	R.VYTAPPSER.S
3948	282 - 290	510.2618	1018.5091	1018.5083	0.70	0	25	0.0046	1	U	R.VYTAPPSER.S
3949	282 - 290	510.2622	1018.5099	1018.5083	1.53	0	24	0.0054	1	U	R.VYTAPPSER.S
13054	297 - 309	494.5785	1480.7138	1480.7158	-1.34	1	69	3.7e-07	1	U	R.TTPSKYETVDQGR.E
13055	297 - 309	741.3654	1480.7162	1480.7158	0.27	1	57	4.6e-06	1	U	R.TTPSKYETVDQGR.E
13056	297 - 309	494.5794	1480.7163	1480.7158	0.36	1	48	3.7e-05	1	U	R.TTPSKYETVDQGR.E
13057	297 - 309	741.3655	1480.7164	1480.7158	0.40	1	44	8.1e-05	1	U	R.TTPSKYETVDQGR.E
20614	297 - 314	709.3669	2125.0788	2125.0804	-0.73	2	6	0.28	1	U	R.TTPSKYETVDQGRELVFR.V
20615	297 - 314	532.2781	2125.0832	2125.0804	1.34	2	19	0.015	1	U	R.TTPSKYETVDQGRELVFR.V
3257	302 - 309	484.2276	966.4406	966.4407	-0.027	0	24	0.0062	1	U	K.YETVDQGR.E
15100	302 - 314	537.9429	1610.8070	1610.8053	1.06	1	9	0.14	1	U	K.YETVDQGRELVFR.V
15101	302 - 314	806.4116	1610.8087	1610.8053	2.13	1	14	0.054	1	U	K.YETVDQGRELVFR.V
17425	318 - 334	899.4312	1796.8479	1796.8502	-1.27	0	80	3.3e-08	1	U	R.MGYDDILVSSPGSLSEK.Y
17426	318 - 334	899.4339	1796.8532	1796.8502	1.67	0	53	1e-05	1	U	R.MGYDDILVSSPGSLSEK.Y
17427	318 - 334	599.9585	1796.8538	1796.8502	1.97	0	32	0.001	1	U	R.MGYDDILVSSPGSLSEK.Y
17635	318 - 334	605.2885	1812.8437	1812.8451	-0.79	0	33	0.00073	1	U	R.MGYDDILVSSPGSLSEK.Y + Oxidation (M)
17636	318 - 334	907.4300	1812.8454	1812.8451	0.12	0	66	6.6e-07	1	U	R.MGYDDILVSSPGSLSEK.Y + Oxidation (M)
17637	318 - 334	907.4310	1812.8474	1812.8451	1.24	0	56	5.2e-06	1	U	R.MGYDDILVSSPGSLSEK.Y + Oxidation (M)
7426	362 - 372	598.7985	1195.5825	1195.5833	-0.71	0	17	0.023	1	U	R.GAQVIEHDNLT.-
7427	362 - 372	598.7993	1195.5841	1195.5833	0.68	0	13	0.057	1	U	R.GAQVIEHDNLT.-

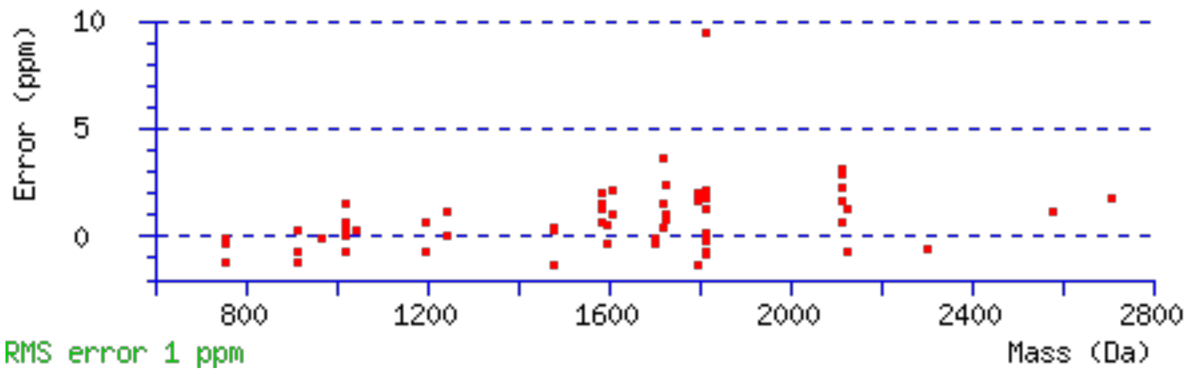

Mascot: http://www.matrixscience.com/

MASCOT Search Results

Protein View: tr|A0A0K9R1K7|A0A0K9R1K7_SPIOL

>tr|A0A0K9R1K7|A0A0K9R1K7_SPIOL Chlorophyll a-b binding protein, chloroplastic OS=Spinacia oleracea OX=3562 GN=SOVF_123890 PE=3 SV=1

**Database:**	Uni-Spinach
**Score:**	1848
**Nominal mass (M_r):**	29569
**Calculated pI:**	7.85

Sequence similarity is available as **an NCBI BLAST search of tr|A0A0K9R1K7|A0A0K9R1K7_SPIOL against nr**.

Search parameters

**MS data file:**	\\172.16.0.213\tank\windowsVM\Bill Cramer\052418\MGF\wc_QE_052418_Cramer_gel_4.mgf
**Enzyme:**	Trypsin: cuts C-term side of KR unless next residue is P.
**Fixed modifications:**	Carbamidomethyl (C)
**Variable modifications:**	Acetyl (K), Acetyl (Protein N-term), Deamidated (NQ), Oxidation (M)

Protein sequence coverage: 48%

Matched peptides shown in ***bold red***.

```
  1 MATQALVSSS LTSSVETARQ ILGARSGLAP SSLRKNSFVV RAASTPPVKQ
 51 NANRPLWFAS KQSLSYLDGS LPGDFGFDPL GLSDPEGTGG FIEPRWLAYG
101 EIINGRYAML GAVGAIAPEI LGKAGLIPQE TALPWFQTGV IPPAGTYNYW
151 ADPFTLFVFE MALMGFAEHR RLQDWYNPGS MGKQYFLGLE KGFAGSGEPA
201 YPGGPIFNPL GFGKDEKSLK ELKLKEVKNG RLAMLAILGY FIQGLVTGVG
251 PYQNLLDHLA DPVNNNILTS LKFH
```

Unformatted sequence string: **274 residues** (for pasting into other applications).

Sort peptides by ◉ Residue Number ○ Increasing Mass ○ Decreasing Mass

Show predicted peptides also

Query	Start – End	Observed	Mr(expt)	Mr(calc)	ppm	M	Score	Expect	Rank	U	Peptide
12053	50 – 61	716.3781	1430.7417	1430.7419	-0.12	0	37	0.00081	1	U	K.QNANRPLWFASK.Q
12054	50 – 61	716.3784	1430.7423	1430.7419	0.30	0	39	0.0017	1	U	K.QNANRPLWFASK.Q
12055	50 – 61	477.9217	1430.7433	1430.7419	0.99	0	11	0.089	1	U	K.QNANRPLWFASK.Q
12056	50 – 61	477.9217	1430.7434	1430.7419	1.06	0	11	0.089	1	U	K.QNANRPLWFASK.Q
12057	50 – 61	477.9223	1430.7452	1430.7419	2.32	0	11	0.09	1	U	K.QNANRPLWFASK.Q
12058	50 – 61	716.3799	1430.7453	1430.7419	2.37	0	34	0.0011	1	U	K.QNANRPLWFASK.Q
12060	50 – 61	716.3821	1430.7496	1430.7419	5.43	0	4	0.56	1	U	K.QNANRPLWFASK.Q
12065	50 – 61	478.2491	1431.7253	1431.7259	-0.36	0	27	0.0032	1	U	K.QNANRPLWFASK.Q + Deamidated (NQ)
12066	50 – 61	716.8707	1431.7268	1431.7259	0.65	0	37	0.00099	1	U	K.QNANRPLWFASK.Q + Deamidated (NQ)
12067	50 – 61	716.8707	1431.7269	1431.7259	0.73	0	16	0.031	1	U	K.QNANRPLWFASK.Q + Deamidated (NQ)
12068	50 – 61	478.2498	1431.7274	1431.7259	1.10	0	11	0.086	1	U	K.QNANRPLWFASK.Q + Deamidated (NQ)
12069	50 – 61	478.2498	1431.7276	1431.7259	1.18	0	13	0.064	1	U	K.QNANRPLWFASK.Q + Deamidated (NQ)
12070	50 – 61	478.2498	1431.7277	1431.7259	1.25	0	16	0.03	1	U	K.QNANRPLWFASK.Q + Deamidated (NQ)
12071	50 – 61	478.2498	1431.7277	1431.7259	1.27	0	21	0.011	1	U	K.QNANRPLWFASK.Q + Deamidated (NQ)
12072	50 – 61	716.8718	1431.7290	1431.7259	2.21	0	24	0.0097	1	U	K.QNANRPLWFASK.Q + Deamidated (NQ)
12073	50 – 61	478.2505	1431.7298	1431.7259	2.74	0	22	0.0083	1	U	K.QNANRPLWFASK.Q + Deamidated (NQ)
12074	50 – 61	478.2510	1431.7311	1431.7259	3.65	0	6	0.29	1	U	K.QNANRPLWFASK.Q + Deamidated (NQ)
12075	50 – 61	716.8728	1431.7311	1431.7259	3.69	0	17	0.024	1	U	K.QNANRPLWFASK.Q + Deamidated (NQ)
12076	50 – 61	478.2513	1431.7320	1431.7259	4.29	0	12	0.069	1	U	K.QNANRPLWFASK.Q + Deamidated (NQ)
12077	50 – 61	716.8738	1431.7331	1431.7259	5.08	0	25	0.0042	1	U	K.QNANRPLWFASK.Q + Deamidated (NQ)
12094	50 – 61	478.5777	1432.7114	1432.7099	1.04	0	5	0.33	1	U	K.QNANRPLWFASK.Q + 2 Deamidated (NQ)
24751	62 – 95	1180.8999	3539.6777	3539.6733	1.25	0	69	3.1e-07	1	U	K.QSLSYLDGSLPGDFGFDPLGLSDPEGTGGFIEPR.W
24752	62 – 95	885.9276	3539.6815	3539.6733	2.31	0	70	2.8e-07	1	U	K.QSLSYLDGSLPGDFGFDPLGLSDPEGTGGFIEPR.W
24755	62 – 95	1181.2340	3540.6802	3540.6573	6.47	0	48	2.9e-05	1	U	K.QSLSYLDGSLPGDFGFDPLGLSDPEGTGGFIEPR.W + Deamidated (NQ)
24756	62 – 95	709.1448	3540.6878	3540.6573	8.61	0	23	0.007	1	U	K.QSLSYLDGSLPGDFGFDPLGLSDPEGTGGFIEPR.W + Deamidated (NQ)
9315	96 – 106	431.2315	1290.6728	1290.6720	0.57	0	22	0.096	1	U	R.WLAYGEIINGR.Y
9330	96 – 106	646.8349	1291.6552	1291.6561	-0.70	0	26	0.0033	1	U	R.WLAYGEIINGR.Y + Deamidated (NQ)
9332	96 – 106	646.8352	1291.6559	1291.6561	-0.085	0	41	0.00042	1	U	R.WLAYGEIINGR.Y + Deamidated (NQ)
9333	96 – 106	646.8359	1291.6573	1291.6561	0.95	0	49	2.8e-05	1	U	R.WLAYGEIINGR.Y + Deamidated (NQ)
9334	96 – 106	431.5600	1291.6581	1291.6561	1.58	0	10	0.11	1	U	R.WLAYGEIINGR.Y + Deamidated (NQ)
9335	96 – 106	646.8364	1291.6583	1291.6561	1.72	0	26	0.0035	1	U	R.WLAYGEIINGR.Y + Deamidated (NQ)
9336	96 – 106	646.8368	1291.6591	1291.6561	2.33	0	44	7.3e-05	1	U	R.WLAYGEIINGR.Y + Deamidated (NQ)
9337	96 – 106	646.8368	1291.6591	1291.6561	2.39	0	38	0.00027	1	U	R.WLAYGEIINGR.Y + Deamidated (NQ)
9338	96 – 106	646.8369	1291.6593	1291.6561	2.53	0	53	1.1e-05	1	U	R.WLAYGEIINGR.Y + Deamidated (NQ)
9339	96 – 106	646.8371	1291.6596	1291.6561	2.74	0	45	0.00018	1	U	R.WLAYGEIINGR.Y + Deamidated (NQ)
9340	96 – 106	646.8374	1291.6603	1291.6561	3.27	0	33	0.00084	1	U	R.WLAYGEIINGR.Y + Deamidated (NQ)

9343	96 – 106	646.8403	1291.6661	1291.6561	7.82	0	49	2.5e-05	1	U	R.WLAYGEIINGR.Y + Deamidated (NQ)
15951	107 – 123	837.4688	1672.9231	1672.9222	0.58	0	25	0.0047	1	U	R.YAMLGAVGAIAPEILGK.A
15952	107 – 123	558.6487	1672.9243	1672.9222	1.26	0	32	0.00091	1	U	R.YAMLGAVGAIAPEILGK.A
15953	107 – 123	837.4699	1672.9253	1672.9222	1.88	0	71	2.1e-07	1	U	R.YAMLGAVGAIAPEILGK.A
16137	107 – 123	845.4658	1688.9171	1688.9171	-0.016	0	12	0.083	1	U	R.YAMLGAVGAIAPEILGK.A + Oxidation (M)
16138	107 – 123	845.4666	1688.9187	1688.9171	0.92	0	111	4e-11	1	U	R.YAMLGAVGAIAPEILGK.A + Oxidation (M)
16139	107 – 123	845.4667	1688.9189	1688.9171	1.06	0	84	1.3e-08	1	U	R.YAMLGAVGAIAPEILGK.A + Oxidation (M)
16140	107 – 123	563.9806	1688.9200	1688.9171	1.72	0	42	0.00012	1	U	R.YAMLGAVGAIAPEILGK.A + Oxidation (M)
16141	107 – 123	563.9808	1688.9206	1688.9171	2.06	0	42	0.00011	1	U	R.YAMLGAVGAIAPEILGK.A + Oxidation (M)
14253	171 – 183	776.3721	1550.7296	1550.7300	-0.24	1	51	2e-05	1	U	R.RLQDWYNPGSMGK.Q
14254	171 – 183	776.3721	1550.7297	1550.7300	-0.21	1	56	7.2e-06	1	U	R.RLQDWYNPGSMGK.Q
14255	171 – 183	776.3724	1550.7302	1550.7300	0.14	1	12	0.08	1	U	R.RLQDWYNPGSMGK.Q
14257	171 – 183	517.9174	1550.7304	1550.7300	0.29	1	9	0.14	1	U	R.RLQDWYNPGSMGK.Q
14258	171 – 183	517.9175	1550.7307	1550.7300	0.43	1	19	0.015	1	U	R.RLQDWYNPGSMGK.Q
14259	171 – 183	517.9176	1550.7309	1550.7300	0.60	1	30	0.0015	1	U	R.RLQDWYNPGSMGK.Q
14487	171 – 183	784.3688	1566.7231	1566.7249	-1.14	1	25	0.0047	1	U	R.RLQDWYNPGSMGK.Q + Oxidation (M)
14488	171 – 183	784.3695	1566.7244	1566.7249	-0.34	1	6	0.26	1	U	R.RLQDWYNPGSMGK.Q + Oxidation (M)
14489	171 – 183	523.2491	1566.7254	1566.7249	0.31	1	2	0.61	1	U	R.RLQDWYNPGSMGK.Q + Oxidation (M)
14491	171 – 183	784.3701	1566.7256	1566.7249	0.47	1	62	1.4e-06	1	U	R.RLQDWYNPGSMGK.Q + Oxidation (M)
14492	171 – 183	523.2493	1566.7261	1566.7249	0.75	1	4	0.44	1	U	R.RLQDWYNPGSMGK.Q + Oxidation (M)
14493	171 – 183	784.3704	1566.7262	1566.7249	0.86	1	13	0.057	1	U	R.RLQDWYNPGSMGK.Q + Oxidation (M)
14495	171 – 183	523.2494	1566.7265	1566.7249	1.00	1	26	0.0037	1	U	R.RLQDWYNPGSMGK.Q + Oxidation (M)
14497	171 – 183	784.3714	1566.7282	1566.7249	2.09	1	54	7.9e-06	1	U	R.RLQDWYNPGSMGK.Q + Oxidation (M)
14499	171 – 183	523.2503	1566.7292	1566.7249	2.72	1	21	0.011	1	U	R.RLQDWYNPGSMGK.Q + Oxidation (M)
11321	172 – 183	698.3219	1394.6292	1394.6289	0.26	0	25	0.0047	1	U	R.LQDWYNPGSMGK.Q
11322	172 – 183	698.3220	1394.6294	1394.6289	0.39	0	59	2.7e-06	1	U	R.LQDWYNPGSMGK.Q
11323	172 – 183	698.3222	1394.6299	1394.6289	0.73	0	5	0.33	1	U	R.LQDWYNPGSMGK.Q
11324	172 – 183	698.3223	1394.6301	1394.6289	0.89	0	42	0.00011	1	U	R.LQDWYNPGSMGK.Q
11325	172 – 183	698.3225	1394.6304	1394.6289	1.08	0	27	0.0028	1	U	R.LQDWYNPGSMGK.Q
11326	172 – 183	698.3238	1394.6330	1394.6289	2.95	0	49	2.4e-05	1	U	R.LQDWYNPGSMGK.Q
11327	172 – 183	698.3240	1394.6335	1394.6289	3.33	0	3	0.53	1	U	R.LQDWYNPGSMGK.Q
11334	172 – 183	698.8132	1395.6119	1395.6129	-0.70	0	12	0.08	1	U	R.LQDWYNPGSMGK.Q + Deamidated (NQ)
11635	172 – 183	706.3188	1410.6231	1410.6238	-0.53	0	39	0.00022	1	U	R.LQDWYNPGSMGK.Q + Oxidation (M)
11638	172 – 183	706.3193	1410.6240	1410.6238	0.12	0	37	0.00034	1	U	R.LQDWYNPGSMGK.Q + Oxidation (M)
11639	172 – 183	706.3194	1410.6243	1410.6238	0.36	0	12	0.069	1	U	R.LQDWYNPGSMGK.Q + Oxidation (M)
11640	172 – 183	706.3194	1410.6243	1410.6238	0.39	0	37	0.00034	1	U	R.LQDWYNPGSMGK.Q + Oxidation (M)
11641	172 – 183	706.3195	1410.6245	1410.6238	0.49	0	8	0.2	1	U	R.LQDWYNPGSMGK.Q + Oxidation (M)
11642	172 – 183	706.3203	1410.6259	1410.6238	1.53	0	5	0.38	1	U	R.LQDWYNPGSMGK.Q + Oxidation (M)
11643	172 – 183	706.3204	1410.6262	1410.6238	1.70	0	10	0.12	1	U	R.LQDWYNPGSMGK.Q + Oxidation (M)
3634	184 – 191	499.2716	996.5287	996.5280	0.69	0	11	0.098	1	U	K.QYFLGLEK.G
3635	184 – 191	499.2717	996.5288	996.5280	0.81	0	13	0.064	1	U	K.QYFLGLEK.G
3636	184 – 191	499.2718	996.5291	996.5280	1.13	0	26	0.01	1	U	K.QYFLGLEK.G
3637	184 – 191	499.2723	996.5300	996.5280	1.95	0	28	0.0079	1	U	K.QYFLGLEK.G
3638	184 – 191	499.2727	996.5309	996.5280	2.90	0	20	0.033	1	U	K.QYFLGLEK.G
21374	192 – 214	746.3729	2236.0968	2236.0953	0.64	0	78	4.5e-08	1	U	K.GFAGSGEPAYPGGPIFNPLGFGK.D
21375	192 – 214	1119.0562	2236.0978	2236.0953	1.09	0	89	4.7e-09	1	U	K.GFAGSGEPAYPGGPIFNPLGFGK.D
23505	192 – 217	870.4276	2608.2609	2608.2598	0.41	1	46	4.7e-05	1	U	K.GFAGSGEPAYPGGPIFNPLGFGKDEK.S
23506	192 – 217	1305.1379	2608.2612	2608.2598	0.54	1	81	2.4e-08	1	U	K.GFAGSGEPAYPGGPIFNPLGFGKDEK.S
23507	192 – 217	870.4279	2608.2618	2608.2598	0.76	1	83	1.8e-08	1	U	K.GFAGSGEPAYPGGPIFNPLGFGKDEK.S
23508	192 – 217	870.4281	2608.2624	2608.2598	1.00	1	79	3.9e-08	1	U	K.GFAGSGEPAYPGGPIFNPLGFGKDEK.S
23509	192 – 217	870.4282	2608.2627	2608.2598	1.10	1	100	3.8e-10	1	U	K.GFAGSGEPAYPGGPIFNPLGFGKDEK.S
23511	192 – 217	870.4291	2608.2656	2608.2598	2.20	1	59	2.9e-06	1	U	K.GFAGSGEPAYPGGPIFNPLGFGKDEK.S
23514	192 – 217	1305.6356	2609.2567	2609.2438	4.93	1	46	5e-05	1	U	K.GFAGSGEPAYPGGPIFNPLGFGKDEK.S + Deamidated (NQ)
23515	192 – 217	870.7600	2609.2582	2609.2438	5.51	1	15	0.042	1	U	K.GFAGSGEPAYPGGPIFNPLGFGKDEK.S + Deamidated (NQ)
23516	192 – 217	870.7610	2609.2611	2609.2438	6.63	1	34	0.00058	1	U	K.GFAGSGEPAYPGGPIFNPLGFGKDEK.S + Deamidated (NQ)
23517	192 – 217	1305.6383	2609.2621	2609.2438	6.99	1	62	1.6e-06	1	U	K.GFAGSGEPAYPGGPIFNPLGFGKDEK.S + Deamidated (NQ)
23519	192 – 217	870.7626	2609.2659	2609.2438	8.46	1	26	0.0038	1	U	K.GFAGSGEPAYPGGPIFNPLGFGKDEK.S + Deamidated (NQ)
23520	192 – 217	653.3240	2609.2668	2609.2438	8.81	1	27	0.003	1	U	K.GFAGSGEPAYPGGPIFNPLGFGKDEK.S + Deamidated (NQ)
134	218 – 223	359.2288	716.4430	716.4432	-0.27	1	6	0.42	1	U	K.SLKELK.L
135	218 – 223	359.2290	716.4435	716.4432	0.38	1	18	0.27	1	U	K.SLKELK.L
3511	221 – 228	493.8160	985.6175	985.6171	0.38	2	13	0.23	1	U	K.ELKLKEVK.N

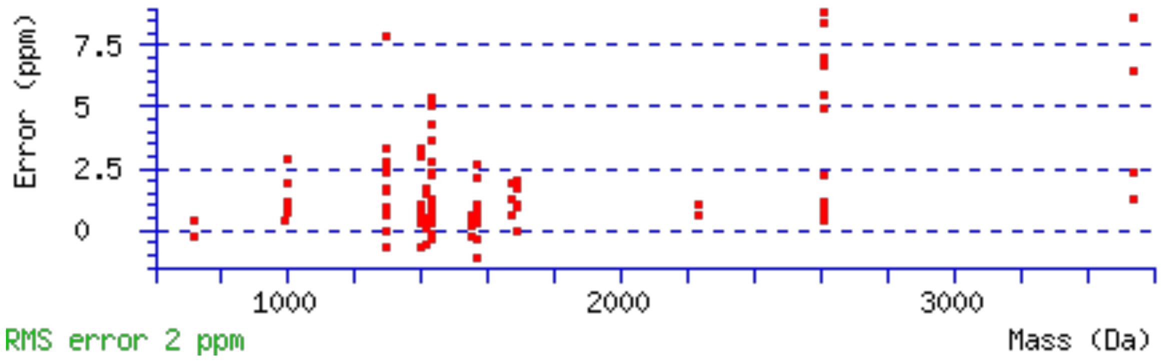

MASCOT Search Results

Protein View: sp|P06450|ATPA_SPIOL

>sp|P06450|ATPA_SPIOL ATP synthase subunit alpha, chloroplastic OS=Spinacia oleracea OX=3562 GN=atpA PE=1 SV=1

**Database:**	Uni-Spinach
**Score:**	1829
**Nominal mass (M_r):**	55474
**Calculated pI:**	5.16

Sequence similarity is available as an NCBI BLAST search of sp|P06450|ATPA_SPIOL against nr.

Search parameters

**MS data file:**	\\172.16.0.213\tank\windowsVM\Bill Cramer\052418\MGF\wc_QE_052418_Cramer_gel_4.mgf
**Enzyme:**	Trypsin: cuts C-term side of KR unless next residue is P.
**Fixed modifications:**	Carbamidomethyl (C)
**Variable modifications:**	Acetyl (K), Acetyl (Protein N-term), Deamidated (NQ), Oxidation (M)

Protein sequence coverage: 50%

Matched peptides shown in ***bold red***.

1 M**ATIRADEIS** **K**IIR**ERIEGY** **NREVKVVNTG** **TVLQVGDGIA** **R**IHGLDEVMA
51 GELVEFEEGT IGIALNLESN NVGVVLMGDG LMIQEGSSVK ATGR**IAQIPV**
101 **SEAYLGRVIN** **ALAKPIDGRG** **EITASESR**LI ESPAPGIMSR **RSVYEPLQTG**
151 **LIAIDAMIPV** **GR**GQR**ELIIG** **DRQTGK**TAVA TDTILNQQGQ NVICVYVAIG
201 QK**ASSVAQVV** **TNFQER**GAME YTIVVAETAD SPATLQYLAP YTGAALAEYF
251 MYRER**HTLII** **YDDLSKQAQA** **YRQMSLLLR**R PPGR**EAYPGD** **VFYLHSR**LLE
301 RAAKLSSLLG EGSMTALPIV ETQAGDVSAY IPTNVISITD GQIFLSADLF
351 NAGIRPAINV GISVSR**VGSA** **AQIK**AMKKVA GKLKLELAQF AELEAFAQFA
401 SDLDK**ATQNQ** **LAR**GQR**LREL** **LKQPQSAPLT** **VEEQVMTIYT** **GTNGYLDSLE**
451 **LDQVRKYLVE** **LR**TYVK**TNKP** **EFQEIISSTK** **TFTEEAEALL** **KEAIQEQMER**
501 **FLLQEQA**

Unformatted sequence string: 507 residues (for pasting into other applications).

Sort peptides by ◉ Residue Number ○ Increasing Mass ○ Decreasing Mass

Show predicted peptides also

Query	Start – End	Observed	Mr(expt)	Mr(calc)	ppm	M	Score	Expect	Rank	U	Peptide
6377	2 – 11	573.3119	1144.6092	1144.6088	0.40	1	6	0.26	1		M.ATIRADEISK.I + Acetyl (Protein N-term)
4200	15 – 22	518.7613	1035.5080	1035.5097	-1.61	1	3	0.49	1		R.ERIEGYNR.E
394	17 – 22	376.1903	750.3661	750.3660	0.080	0	14	0.34	1		R.IEGYNR.E
5663	17 – 25	554.2927	1106.5709	1106.5720	-1.00	1	4	0.91	1		R.IEGYNREVK.V
19381	23 – 41	652.3697	1954.0872	1954.0848	1.27	1	3	0.51	1		R.EVKVVNTGTVLQVGDGIAR.I
14936	26 – 41	799.9464	1597.8783	1597.8788	-0.30	0	76	7.1e-08	1		K.VVNTGTVLQVGDGIAR.I
14937	26 – 41	533.6336	1597.8791	1597.8788	0.20	0	64	1.1e-06	1		K.VVNTGTVLQVGDGIAR.I
14938	26 – 41	533.6341	1597.8806	1597.8788	1.13	0	17	0.026	1		K.VVNTGTVLQVGDGIAR.I
14939	26 – 41	799.9477	1597.8808	1597.8788	1.28	0	79	3.8e-08	1		K.VVNTGTVLQVGDGIAR.I
14969	26 – 41	800.4376	1598.8606	1598.8628	-1.41	0	14	0.097	1		K.VVNTGTVLQVGDGIAR.I + Deamidated (NQ)
11754	95 – 107	708.8963	1415.7781	1415.7772	0.61	0	44	7.4e-05	1		R.IAQIPVSEAYLGR.V
11755	95 – 107	708.8968	1415.7791	1415.7772	1.34	0	54	9.4e-06	1		R.IAQIPVSEAYLGR.V
11756	95 – 107	472.9341	1415.7804	1415.7772	2.25	0	40	0.00018	1		R.IAQIPVSEAYLGR.V
8861	108 – 119	634.3727	1266.7308	1266.7296	0.99	0	12	0.072	1		R.VINALAKPIDGR.G + Deamidated (NQ)
21057	108 – 128	733.0697	2196.1872	2196.1862	0.47	1	26	0.004	1		R.VINALAKPIDGRGEITASESR.L
21058	108 – 128	550.0543	2196.1882	2196.1862	0.91	1	36	0.00044	1		R.VINALAKPIDGRGEITASESR.L
21064	108 – 128	550.3016	2197.1774	2197.1702	3.27	1	4	0.39	1		R.VINALAKPIDGRGEITASESR.L + Deamidated (NQ)
21065	108 – 128	550.3032	2197.1838	2197.1702	6.18	1	10	0.12	1		R.VINALAKPIDGRGEITASESR.L + Deamidated (NQ)
21066	108 – 128	550.3037	2197.1857	2197.1702	7.07	1	5	0.33	1		R.VINALAKPIDGRGEITASESR.L + Deamidated (NQ)
21067	108 – 128	733.4026	2197.1859	2197.1702	7.16	1	11	0.09	1		R.VINALAKPIDGRGEITASESR.L + Deamidated (NQ)
22651	141 – 162	800.4422	2398.3046	2398.3042	0.17	1	62	1.7e-06	1		R.RSVYEPLQTGLIAIDAMIPVGR.G
22726	141 – 162	805.7762	2414.3068	2414.2991	3.17	1	62	1.4e-06	1		R.RSVYEPLQTGLIAIDAMIPVGR.G + Oxidation (M)
22736	141 – 162	806.1091	2415.3054	2415.2832	9.20	1	53	1.2e-05	1		R.RSVYEPLQTGLIAIDAMIPVGR.G + Deamidated (NQ); Oxidation (M)
21451	142 – 162	748.4076	2242.2010	2242.2031	-0.97	0	64	9.9e-07	1		R.SVYEPLQTGLIAIDAMIPVGR.G
21452	142 – 162	1122.1082	2242.2019	2242.2031	-0.56	0	69	3.1e-07	1		R.SVYEPLQTGLIAIDAMIPVGR.G
21582	142 – 162	753.7394	2258.1964	2258.1980	-0.72	0	62	1.7e-06	1		R.SVYEPLQTGLIAIDAMIPVGR.G + Oxidation (M)

1135	166 – 172	408.2351	814.4556	814.4548	0.93	0	14	0.099	1		R.ELIIGDR.Q
1136	166 – 172	408.2352	814.4558	814.4548	1.15	0	23	0.081	1		R.ELIIGDR.Q
8143	166 – 176	410.5666	1228.6779	1228.6775	0.33	1	3	0.78	1		R.ELIIGDRQTGK.T
14020	203 – 216	768.3945	1534.7744	1534.7740	0.27	0	99	5.2e-10	1		K.ASSVAQVVTNFQER.G
14021	203 – 216	512.5990	1534.7752	1534.7740	0.78	0	59	2.8e-06	1		K.ASSVAQVVTNFQER.G
14022	203 – 216	512.5994	1534.7764	1534.7740	1.61	0	34	0.00059	1		K.ASSVAQVVTNFQER.G
14032	203 – 216	768.8903	1535.7661	1535.7580	5.28	0	64	1.1e-06	1		K.ASSVAQVVTNFQER.G + Deamidated (NQ)
9823	256 – 266	659.3546	1316.6947	1316.6976	-2.21	0	37	0.00087	1		R.HTLIIYDDLSK.Q
9825	256 – 266	659.3569	1316.6992	1316.6976	1.19	0	77	2e-07	1		R.HTLIIYDDLSK.Q
9826	256 – 266	439.9072	1316.6996	1316.6976	1.54	0	22	0.0078	1		R.HTLIIYDDLSK.Q
9827	256 – 266	439.9075	1316.7007	1316.6976	2.38	0	28	0.0031	1		R.HTLIIYDDLSK.Q
9829	256 – 266	659.3584	1316.7023	1316.6976	3.53	0	72	2.8e-07	1		R.HTLIIYDDLSK.Q
19963	256 – 272	679.0256	2034.0551	2034.0534	0.82	1	7	0.22	1		R.HTLIIYDDLSKQAQAYR.Q
1813	273 – 279	430.7548	859.4951	859.4949	0.22	0	47	0.00041	1		R.QMSLLLR.R
1814	273 – 279	430.7554	859.4963	859.4949	1.60	0	35	0.0053	1		R.QMSLLLR.R
2064	273 – 279	438.7523	875.4901	875.4899	0.34	0	41	0.0014	1		R.QMSLLLR.R + Oxidation (M)
2065	273 – 279	438.7531	875.4917	875.4899	2.06	0	42	0.0012	1		R.QMSLLLR.R + Oxidation (M)
14287	285 – 297	777.3726	1552.7307	1552.7310	-0.22	0	54	7.8e-06	1		R.EAYPGDVFYLHSR.L
14288	285 – 297	777.3734	1552.7322	1552.7310	0.73	0	45	6.6e-05	1		R.EAYPGDVFYLHSR.L
14289	285 – 297	518.5853	1552.7341	1552.7310	1.97	0	44	7.1e-05	1		R.EAYPGDVFYLHSR.L
14290	285 – 297	518.5855	1552.7345	1552.7310	2.25	0	50	2.1e-05	1		R.EAYPGDVFYLHSR.L
658	367 – 374	387.2293	772.4440	772.4443	-0.43	0	26	0.014	1	U	R.VGSAAQIK.A
2357	406 – 413	451.2462	900.4779	900.4777	0.23	0	31	0.0046	1	U	K.ATQNQLAR.G
642	417 – 422	386.2580	770.5014	770.5014	-0.022	1	17	0.26	2	U	R.LRELLK.Q
24823	423 – 455	1232.6110	3694.8112	3694.8036	2.05	0	93	2.1e-09	1	U	K.QPQSAPLTVEEQVMTIYTGTNGYLDSLELDQVR.K
24824	423 – 455	924.9549	3695.7905	3695.7876	0.79	0	39	0.00021	1	U	K.QPQSAPLTVEEQVMTIYTGTNGYLDSLELDQVR.K + Deamidated (NQ)
24825	423 – 455	1232.9403	3695.7991	3695.7876	3.12	0	106	1.3e-10	1	U	K.QPQSAPLTVEEQVMTIYTGTNGYLDSLELDQVR.K + Deamidated (NQ)
24856	423 – 456	1275.6438	3823.9096	3823.8826	7.06	1	61	1.8e-06	1	U	K.QPQSAPLTVEEQVMTIYTGTNGYLDSLELDQVRK.Y + Deamidated (NQ)
24857	423 – 456	957.2293	3824.8881	3824.8666	5.63	1	73	1.4e-07	1	U	K.QPQSAPLTVEEQVMTIYTGTNGYLDSLELDQVRK.Y + 2 Deamidated (NQ)
24858	423 – 456	957.2308	3824.8942	3824.8666	7.23	1	66	7.2e-07	1	U	K.QPQSAPLTVEEQVMTIYTGTNGYLDSLELDQVRK.Y + 2 Deamidated (NQ)
2625	456 – 462	460.7819	919.5493	919.5491	0.26	1	23	0.029	1	U	R.KYLVELR.T
2626	456 – 462	460.7823	919.5500	919.5491	0.97	1	34	0.0024	1	U	R.KYLVELR.T
867	457 – 462	396.7345	791.4545	791.4541	0.50	0	36	0.0035	1	U	K.YLVELR.T
871	457 – 462	396.7352	791.4558	791.4541	2.14	0	39	0.0019	1	U	K.YLVELR.T
15257	467 – 480	811.4252	1620.8358	1620.8359	-0.056	0	52	1.2e-05	1	U	K.TNKPEFQEIISSTK.T
15258	467 – 480	541.2860	1620.8361	1620.8359	0.14	0	87	6.5e-09	1	U	K.TNKPEFQEIISSTK.T
15259	467 – 480	811.4255	1620.8364	1620.8359	0.32	0	1	0.73	1	U	K.TNKPEFQEIISSTK.T
15260	467 – 480	541.2878	1620.8417	1620.8359	3.60	0	49	2.5e-05	1	U	K.TNKPEFQEIISSTK.T
8573	481 – 491	417.8874	1250.6403	1250.6394	0.74	0	10	0.11	1	U	K.TFTEEAEALLK.E
8574	481 – 491	626.3281	1250.6416	1250.6394	1.75	0	53	1.1e-05	1	U	K.TFTEEAEALLK.E
8575	481 – 491	626.3286	1250.6426	1250.6394	2.57	0	49	2.7e-05	1	U	K.TFTEEAEALLK.E
22571	481 – 500	794.7226	2381.1459	2381.1420	1.62	1	49	2.5e-05	1	U	K.TFTEEAEALLKEAIQEQMER.F + Oxidation (M)
6131	492 – 500	567.2667	1132.5189	1132.5182	0.62	0	59	3.1e-06	1	U	K.EAIQEQMER.F
6132	492 – 500	567.2671	1132.5197	1132.5182	1.32	0	40	0.00019	1	U	K.EAIQEQMER.F
6435	492 – 500	575.2638	1148.5130	1148.5132	-0.12	0	20	0.014	1	U	K.EAIQEQMER.F + Oxidation (M)
1641	501 – 507	424.7297	847.4449	847.4440	1.14	0	6	0.26	1	U	R.FLLQEQA.-

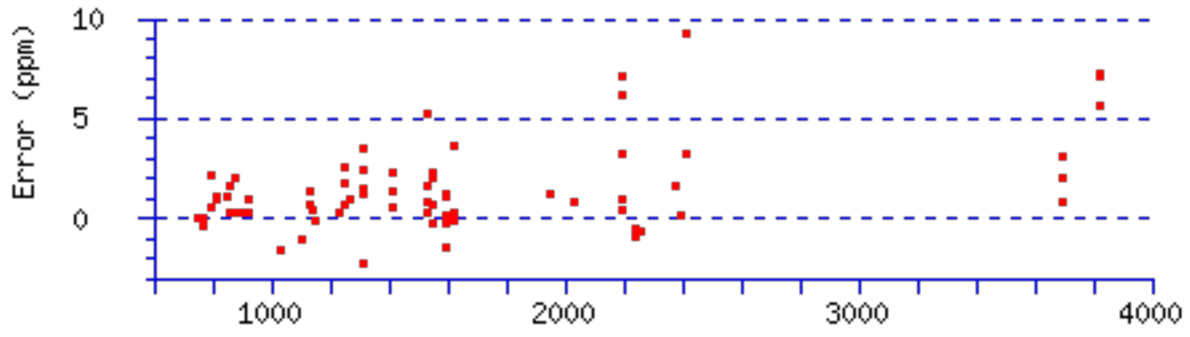

RMS error 2 ppm

Mascot: http://www.matrixscience.com/

MASCOT Search Results

Protein View: tr|A0A0K9RHQ2|A0A0K9RHQ2_SPIOL

>tr|A0A0K9RHQ2|A0A0K9RHQ2_SPIOL Uncharacterized protein OS=Spinacia oleracea OX=3562 GN=SOVF_071290 PE=4 SV=1

**Database:**	Uni-Spinach
**Score:**	1713
**Nominal mass (M_r):**	18374
**Calculated pI:**	9.33

Sequence similarity is available as **an NCBI BLAST search of tr|A0A0K9RHQ2|A0A0K9RHQ2_SPIOL against nr**.

Search parameters

**MS data file:**	\\172.16.0.213\tank\windowsVM\Bill Cramer\052418\MGF\wc_QE_052418_Cramer_gel_4.mgf
**Enzyme:**	Trypsin: cuts C-term side of KR unless next residue is P.
**Fixed modifications:**	**Carbamidomethyl (C)**
**Variable modifications:**	**Acetyl (K)**, **Acetyl (Protein N-term)**, **Deamidated (NQ)**, **Oxidation (M)**

Protein sequence coverage: 23%

Matched peptides shown in ***bold red***.

```
  1 MAAATASLSS TLLAPCSSKQ PQPQQQHQHQ QLKCKSFSGL RPLKLNISSN
 51 NSSSSLSMSS ARRSMTCRAE LSPSLVISLS TGLSLFLGRF VFFNFQRENM
101 AKQVPEQNGM SHFEAGDTRA KEYVSLLKSN DPVGFNIVDV LAWGSIGHIV
151 AYYILATASN GYDPSFF
```

Unformatted sequence string: **167 residues** (for pasting into other applications).

Sort peptides by ◉ Residue Number ○ Increasing Mass ○ Decreasing Mass

Show predicted peptides also

Query	Start – End	Observed	Mr(expt)	Mr(calc)	ppm	M	Score	Expect	Rank	U	Peptide
5601	90 – 97	552.7852	1103.5559	1103.5553	0.55	0	27	0.0028	1	U	R.FVFFNFQR.E
5602	90 – 97	552.7852	1103.5559	1103.5553	0.60	0	22	0.0082	1	U	R.FVFFNFQR.E
5603	90 – 97	552.7859	1103.5572	1103.5553	1.76	0	41	0.00014	1	U	R.FVFFNFQR.E
5614	90 – 97	553.2758	1104.5370	1104.5393	-2.05	0	27	0.0031	1	U	R.FVFFNFQR.E + Deamidated (NQ)
5617	90 – 97	553.2777	1104.5409	1104.5393	1.49	0	37	0.00051	1	U	R.FVFFNFQR.E + Deamidated (NQ)
16011	90 – 102	839.4128	1676.8110	1676.8133	-1.39	1	42	0.00012	1	U	R.FVFFNFQRENMAK.Q
16012	90 – 102	559.9459	1676.8160	1676.8133	1.58	1	10	0.11	1	U	R.FVFFNFQRENMAK.Q
16013	90 – 102	559.9465	1676.8175	1676.8133	2.52	1	17	0.025	1	U	R.FVFFNFQRENMAK.Q
16172	90 – 102	847.4098	1692.8050	1692.8082	-1.90	1	0	0.9	1	U	R.FVFFNFQRENMAK.Q + Oxidation (M)
16173	90 – 102	847.4107	1692.8067	1692.8082	-0.88	1	17	0.024	1	U	R.FVFFNFQRENMAK.Q + Oxidation (M)
16175	90 – 102	565.2773	1692.8101	1692.8082	1.07	1	22	0.0079	1	U	R.FVFFNFQRENMAK.Q + Oxidation (M)
18812	103 – 119	634.9501	1901.8285	1901.8326	-2.14	0	54	8.3e-06	1	U	K.QVPEQNGMSHFEAGDTR.A
18813	103 – 119	951.9222	1901.8298	1901.8326	-1.50	0	81	2.7e-08	1	U	K.QVPEQNGMSHFEAGDTR.A
18814	103 – 119	951.9229	1901.8312	1901.8326	-0.73	0	74	1.1e-07	1	U	K.QVPEQNGMSHFEAGDTR.A
18815	103 – 119	634.9516	1901.8331	1901.8326	0.25	0	55	7.5e-06	1	U	K.QVPEQNGMSHFEAGDTR.A
18816	103 – 119	634.9533	1901.8382	1901.8326	2.91	0	45	5.8e-05	1	U	K.QVPEQNGMSHFEAGDTR.A
18821	103 – 119	952.4103	1902.8060	1902.8166	-5.56	0	64	1.1e-06	1	U	K.QVPEQNGMSHFEAGDTR.A + Deamidated (NQ)
18822	103 – 119	952.4105	1902.8064	1902.8166	-5.38	0	74	1.1e-07	1	U	K.QVPEQNGMSHFEAGDTR.A + Deamidated (NQ)
18823	103 – 119	952.4143	1902.8141	1902.8166	-1.32	0	68	4.4e-07	1	U	K.QVPEQNGMSHFEAGDTR.A + Deamidated (NQ)
18824	103 – 119	952.4145	1902.8145	1902.8166	-1.14	0	72	1.7e-07	1	U	K.QVPEQNGMSHFEAGDTR.A + Deamidated (NQ)
18825	103 – 119	635.2791	1902.8153	1902.8166	-0.69	0	9	0.29	1	U	K.QVPEQNGMSHFEAGDTR.A + Deamidated (NQ)
18826	103 – 119	952.4150	1902.8154	1902.8166	-0.65	0	70	2.6e-07		U	

									1		K.QVPEQNGMSHFEAGDTR.A + Deamidated (NQ)
18827	103 - 119	635.2795	1902.8168	1902.8166	0.084	0	20	0.014	1	U	K.QVPEQNGMSHFEAGDTR.A + Deamidated (NQ)
18828	103 - 119	635.2796	1902.8171	1902.8166	0.24	0	47	3.5e-05	1	U	K.QVPEQNGMSHFEAGDTR.A + Deamidated (NQ)
18829	103 - 119	635.2796	1902.8171	1902.8166	0.25	0	66	6.7e-07	1	U	K.QVPEQNGMSHFEAGDTR.A + Deamidated (NQ)
18830	103 - 119	635.2797	1902.8173	1902.8166	0.32	0	52	1.5e-05	1	U	K.QVPEQNGMSHFEAGDTR.A + Deamidated (NQ)
18831	103 - 119	635.2797	1902.8173	1902.8166	0.35	0	43	9.1e-05	1	U	K.QVPEQNGMSHFEAGDTR.A + Deamidated (NQ)
18832	103 - 119	635.2798	1902.8177	1902.8166	0.56	0	22	0.0081	1	U	K.QVPEQNGMSHFEAGDTR.A + Deamidated (NQ)
18833	103 - 119	952.4162	1902.8178	1902.8166	0.59	0	71	2.3e-07	1	U	K.QVPEQNGMSHFEAGDTR.A + Deamidated (NQ)
18834	103 - 119	635.2800	1902.8183	1902.8166	0.85	0	66	6.7e-07	1	U	K.QVPEQNGMSHFEAGDTR.A + Deamidated (NQ)
18835	103 - 119	635.2802	1902.8189	1902.8166	1.18	0	66	6.8e-07	1	U	K.QVPEQNGMSHFEAGDTR.A + Deamidated (NQ)
18836	103 - 119	635.2820	1902.8242	1902.8166	3.98	0	66	7e-07	1	U	K.QVPEQNGMSHFEAGDTR.A + Deamidated (NQ)
18980	103 - 119	959.9201	1917.8257	1917.8275	-0.97	0	58	3.3e-06	1	U	K.QVPEQNGMSHFEAGDTR.A + Oxidation (M)
18981	103 - 119	640.2828	1917.8265	1917.8275	-0.54	0	26	0.0036	1	U	K.QVPEQNGMSHFEAGDTR.A + Oxidation (M)
18982	103 - 119	640.2829	1917.8269	1917.8275	-0.35	0	4	0.46	1	U	K.QVPEQNGMSHFEAGDTR.A + Oxidation (M)
18983	103 - 119	640.2829	1917.8269	1917.8275	-0.32	0	40	0.00017	1	U	K.QVPEQNGMSHFEAGDTR.A + Oxidation (M)
18984	103 - 119	640.2830	1917.8270	1917.8275	-0.26	0	57	4.7e-06	1	U	K.QVPEQNGMSHFEAGDTR.A + Oxidation (M)
18985	103 - 119	959.9211	1917.8276	1917.8275	0.033	0	77	6.1e-08	1	U	K.QVPEQNGMSHFEAGDTR.A + Oxidation (M)
18986	103 - 119	640.2841	1917.8306	1917.8275	1.60	0	45	5.7e-05	1	U	K.QVPEQNGMSHFEAGDTR.A + Oxidation (M)
18990	103 - 119	960.4102	1918.8059	1918.8116	-2.94	0	64	1.1e-06	1	U	K.QVPEQNGMSHFEAGDTR.A + Deamidated (NQ); Oxidation (M)
18991	103 - 119	640.6103	1918.8091	1918.8116	-1.30	0	60	2.6e-06	1	U	K.QVPEQNGMSHFEAGDTR.A + Deamidated (NQ); Oxidation (M)
18992	103 - 119	640.6103	1918.8092	1918.8116	-1.23	0	53	1e-05	1	U	K.QVPEQNGMSHFEAGDTR.A + Deamidated (NQ); Oxidation (M)
18993	103 - 119	960.4123	1918.8100	1918.8116	-0.83	0	44	7.7e-05	1	U	K.QVPEQNGMSHFEAGDTR.A + Deamidated (NQ); Oxidation (M)
18994	103 - 119	960.4123	1918.8101	1918.8116	-0.77	0	63	1.2e-06	1	U	K.QVPEQNGMSHFEAGDTR.A + Deamidated (NQ); Oxidation (M)
18995	103 - 119	960.4127	1918.8109	1918.8116	-0.36	0	63	1.3e-06	1	U	K.QVPEQNGMSHFEAGDTR.A + Deamidated (NQ); Oxidation (M)
18996	103 - 119	640.6109	1918.8109	1918.8116	-0.35	0	46	4.5e-05	1	U	K.QVPEQNGMSHFEAGDTR.A + Deamidated (NQ); Oxidation (M)
18997	103 - 119	640.6111	1918.8115	1918.8116	-0.027	0	40	0.00018	1	U	K.QVPEQNGMSHFEAGDTR.A + Deamidated (NQ); Oxidation (M)
18998	103 - 119	960.4131	1918.8116	1918.8116	0.041	0	53	1.1e-05	1	U	K.QVPEQNGMSHFEAGDTR.A + Deamidated (NQ); Oxidation (M)
18999	103 - 119	640.6112	1918.8118	1918.8116	0.11	0	53	9.9e-06	1	U	K.QVPEQNGMSHFEAGDTR.A + Deamidated (NQ); Oxidation (M)
19000	103 - 119	640.6112	1918.8118	1918.8116	0.15	0	22	0.0082	1	U	K.QVPEQNGMSHFEAGDTR.A + Deamidated (NQ); Oxidation (M)
19001	103 - 119	640.6114	1918.8123	1918.8116	0.41	0	14	0.047	1	U	K.QVPEQNGMSHFEAGDTR.A + Deamidated (NQ); Oxidation (M)
19002	103 - 119	640.6118	1918.8136	1918.8116	1.08	0	6	0.26	1	U	K.QVPEQNGMSHFEAGDTR.A + Deamidated (NQ); Oxidation (M)
19003	103 - 119	640.6119	1918.8138	1918.8116	1.17	0	21	0.011	1	U	K.QVPEQNGMSHFEAGDTR.A

											+ Deamidated (NQ); Oxidation (M)
19004	103 - 119	640.6121	1918.8144	1918.8116	1.46	0	5	0.37	1	U	K.QVPEQNGMSHFEAGDTR.A + Deamidated (NQ); Oxidation (M)
19005	103 - 119	640.6125	1918.8158	1918.8116	2.19	0	54	9.2e-06	1	U	K.QVPEQNGMSHFEAGDTR.A + Deamidated (NQ); Oxidation (M)
19006	103 - 119	640.6127	1918.8163	1918.8116	2.49	0	18	0.023	1	U	K.QVPEQNGMSHFEAGDTR.A + Deamidated (NQ); Oxidation (M)
19008	103 - 119	640.6138	1918.8197	1918.8116	4.23	0	2	0.61	1	U	K.QVPEQNGMSHFEAGDTR.A + Deamidated (NQ); Oxidation (M)
19010	103 - 119	640.6152	1918.8237	1918.8116	6.33	0	5	0.35	1	U	K.QVPEQNGMSHFEAGDTR.A + Deamidated (NQ); Oxidation (M)
19018	103 - 119	640.9431	1919.8075	1919.7956	6.23	0	14	0.085	1	U	K.QVPEQNGMSHFEAGDTR.A + 2 Deamidated (NQ); Oxidation (M)
19019	103 - 119	640.9446	1919.8119	1919.7956	8.52	0	9	0.13	1	U	K.QVPEQNGMSHFEAGDTR.A + 2 Deamidated (NQ); Oxidation (M)
19020	103 - 119	640.9447	1919.8123	1919.7956	8.71	0	20	0.015	1	U	K.QVPEQNGMSHFEAGDTR.A + 2 Deamidated (NQ); Oxidation (M)
20454	103 - 121	701.3287	2100.9644	2100.9647	-0.15	1	6	0.26	1	U	K.QVPEQNGMSHFEAGDTRAK.E
4431	120 - 128	350.8778	1049.6117	1049.6121	-0.37	1	15	0.042	1	U	R.AKEYVSLLK.S
4432	120 - 128	525.8135	1049.6124	1049.6121	0.37	1	38	0.00039	1	U	R.AKEYVSLLK.S
4433	120 - 128	350.8781	1049.6126	1049.6121	0.49	1	14	0.048	1	U	R.AKEYVSLLK.S
4434	120 - 128	525.8138	1049.6130	1049.6121	0.91	1	37	0.00062	1	U	R.AKEYVSLLK.S
4435	120 - 128	525.8138	1049.6130	1049.6121	0.93	1	40	0.00035	1	U	R.AKEYVSLLK.S
4436	120 - 128	350.8783	1049.6131	1049.6121	0.95	1	3	0.56	1	U	R.AKEYVSLLK.S
4437	120 - 128	350.8783	1049.6131	1049.6121	0.99	1	16	0.063	1	U	R.AKEYVSLLK.S
4438	120 - 128	350.8784	1049.6132	1049.6121	1.11	1	18	0.076	1	U	R.AKEYVSLLK.S
4439	120 - 128	350.8784	1049.6134	1049.6121	1.26	1	21	0.053	1	U	R.AKEYVSLLK.S
4441	120 - 128	350.8787	1049.6144	1049.6121	2.19	1	12	0.07	1	U	R.AKEYVSLLK.S
4442	120 - 128	350.8788	1049.6145	1049.6121	2.31	1	8	0.19	1	U	R.AKEYVSLLK.S
4443	120 - 128	525.8148	1049.6151	1049.6121	2.92	1	15	0.036	1	U	R.AKEYVSLLK.S
1676	122 - 128	426.2477	850.4809	850.4800	1.07	0	14	0.18	1	U	K.EYVSLLK.S
1677	122 - 128	426.2478	850.4811	850.4800	1.31	0	21	0.05	1	U	K.EYVSLLK.S
1678	122 - 128	426.2480	850.4815	850.4800	1.82	0	14	0.1	1	U	K.EYVSLLK.S

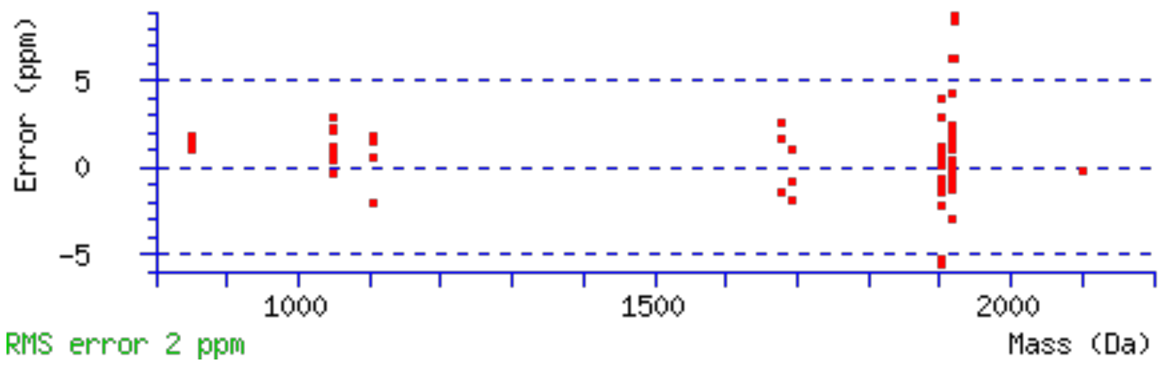

MASCOT Search Results

Protein View: tr|A0A0K9R5V6|A0A0K9R5V6_SPIOL

>tr|A0A0K9R5V6|A0A0K9R5V6_SPIOL Rhodanese domain-containing protein OS=Spinacia oleracea OX=3562 GN=SOVF_109900 PE=4 SV=1

**Database:**	Uni-Spinach
**Score:**	1584
**Nominal mass (M_r):**	43226
**Calculated pI:**	9.44

Sequence similarity is available as **an NCBI BLAST search of tr|A0A0K9R5V6|A0A0K9R5V6_SPIOL against nr**.

Search parameters

**MS data file:**	\\172.16.0.213\tank\windowsVM\Bill Cramer\052418\MGF\wc_QE_052418_Cramer_gel_4.mgf
**Enzyme:**	Trypsin: cuts C-term side of KR unless next residue is P.
**Fixed modifications:**	**Carbamidomethyl (C)**
**Variable modifications:**	**Acetyl (K)**, **Acetyl (Protein N-term)**, **Deamidated (NQ)**, **Oxidation (M)**

Protein sequence coverage: 55%

Matched peptides shown in ***bold red***.

```
  1 MAMEMMALRV SASAKPTISP SSSSSSSQKS QLLKQSSKLQ LKQAQNLFFP
 51 TSTSLSLLTL FTTPFDAKAV SLPKEQLVTS LTQVEQTIDQ VQEVGSSVFD
101 SAQKVFQVVA EALKPGIDAA TPIVQQAGQE AFKAASPLIS EASKKAQEAM
151 QNSGISSESM TTATQTVTSA AEQTTKAFED AKPLASSTFE TISNSDPALL
201 AEGAAALFLA YLLFPRVWSI VSFNLRGYKG GLTPAQTLEM LCTQNYYLID
251 MRSEKDKNKA GIPQLPSSAK SRMIAIPLEE LPSKVRNLVR NSKKVEAEIV
301 ALKVSYLKKL SKSTNIVIMD SYSDSAKTVA KSLTGLGFKN SWILTDGFSG
351 GKGWLQSRLG TESYNLSFGE IFSPSRIISG GTGRFGTTSS TVQIGRKMLP
401 GSN
```

Unformatted sequence string: **403 residues** (for pasting into other applications).

Sort peptides by ◉ Residue Number ○ Increasing Mass ○ Decreasing Mass

Show predicted peptides also

Query	Start – End	Observed	Mr(expt)	Mr(calc)	ppm	M	Score	Expect	Rank	U	Peptide
24530	75 – 104	1098.5508	3292.6307	3292.6311	-0.12	0	91	2.7e-09	1	U	K.EQLVTSLTQVEQTIDQVQEVGSSVFDSAQK.V
24531	75 – 104	824.4163	3293.6362	3293.6151	6.40	0	89	5e-09	1	U	K.EQLVTSLTQVEQTIDQVQEVGSSVFDSAQK.V + Deamidated (NQ)
24317	105 – 133	757.4147	3025.6298	3025.6124	5.75	0	39	0.00022	1	U	K.VFQVVAEALKPGIDAATPIVQQAGQEAFK.A + Deamidated (NQ)
24318	105 – 133	1009.5512	3025.6318	3025.6124	6.40	0	64	4.6e-06	1	U	K.VFQVVAEALKPGIDAATPIVQQAGQEAFK.A + Deamidated (NQ)
4942	134 – 144	537.2959	1072.5773	1072.5764	0.87	0	55	6.6e-06	1	U	K.AASPLISEASK.K
4943	134 – 144	537.2967	1072.5789	1072.5764	2.28	0	26	0.0034	1	U	K.AASPLISEASK.K
7560	134 – 145	601.3406	1200.6666	1200.6714	-4.00	1	48	3.2e-05	1	U	K.AASPLISEASKK.A
7561	134 – 145	601.3409	1200.6672	1200.6714	-3.45	1	31	0.0012	1	U	K.AASPLISEASKK.A
7562	134 – 145	401.2306	1200.6699	1200.6714	-1.23	1	29	0.0018	1	U	K.AASPLISEASKK.A
7563	134 – 145	401.2308	1200.6707	1200.6714	-0.56	1	17	0.023	1	U	K.AASPLISEASKK.A
24564	145 – 176	830.3909	3317.5346	3317.5239	3.24	1	72	1.6e-07	1	U	K.KAQEAMQNSGISSESMTTATQTVTSAAEQTTK.A + Deamidated (NQ)
24565	145 – 176	1106.8547	3317.5424	3317.5239	5.58	1	122	3.3e-12	1	U	K.KAQEAMQNSGISSESMTTATQTVTSAAEQTTK.A + Deamidated (NQ)
24586	145 – 176	1112.1863	3333.5370	3333.5188	5.47	1	98	5.9e-10	1	U	K.KAQEAMQNSGISSESMTTATQTVTSAAEQTTK.A + Deamidated (NQ); Oxidation (M)
24587	145 – 176	834.3926	3333.5415	3333.5188	6.80	1	53	1.2e-05	1	U	K.KAQEAMQNSGISSESMTTATQTVTSAAEQTTK.A + Deamidated (NQ); Oxidation (M)
24453	146 – 176	1064.1587	3189.4542	3189.4289	7.94	0	118	9.5e-12	1	U	K.AQEAMQNSGISSESMTTATQTVTSAAEQTTK.A + Deamidated (NQ)
24468	146 – 176	1069.4889	3205.4448	3205.4238	6.56	0	118	9.3e-12	1	U	K.AQEAMQNSGISSESMTTATQTVTSAAEQTTK.A + Deamidated (NQ); Oxidation (M)
24480	146 – 176	1075.1512	3222.4319	3222.4028	9.05	0	106	1.2e-10	1	U	K.AQEAMQNSGISSESMTTATQTVTSAAEQTTK.A + 2 Deamidated (NQ); 2 Oxidation (M)
4834	260 – 270	534.8063	1067.5980	1067.5975	0.49	0	37	0.00039	1	U	K.AGIPQLPSSAK.S
4837	260 – 270	534.8065	1067.5985	1067.5975	0.93	0	26	0.0039	1	U	K.AGIPQLPSSAK.S
10248	273 – 284	670.8779	1339.7412	1339.7421	-0.66	0	35	0.00049	1	U	R.MIAIPLEELPSK.V
10249	273 – 284	670.8790	1339.7435	1339.7421	1.07	0	48	2.9e-05	1	U	R.MIAIPLEELPSK.V
10544	273 – 284	678.8755	1355.7365	1355.7370	-0.39	0	44	7.9e-05	1	U	R.MIAIPLEELPSK.V + Oxidation (M)
10545	273 – 284	678.8760	1355.7374	1355.7370	0.31	0	33	0.0008	1	U	R.MIAIPLEELPSK.V + Oxidation (M)
14902	273 – 286	532.6442	1594.9107	1594.9116	-0.55	1	34	0.00072	1	U	R.MIAIPLEELPSKVR.N
14903	273 – 286	532.6447	1594.9121	1594.9116	0.33	1	29	0.0019	1	U	R.MIAIPLEELPSKVR.N

15107	273 - 286	537.9772	1610.9099	1610.9065	2.11	1	28	0.0021	1	U	R.MIAIPLEELPSKVR.N + Oxidation (M)
15108	273 - 286	537.9785	1610.9135	1610.9065	4.35	1	29	0.0019	1	U	R.MIAIPLEELPSKVR.N + Oxidation (M)
5270	285 - 293	363.2139	1086.6197	1086.6145	4.80	2	2	0.63	1	U	K.VRNLVRNSK.K + 2 Deamidated (NQ)
5513	294 - 303	367.2292	1098.6657	1098.6648	0.76	1	27	0.022	1	U	K.KVEAEIVALK.V
5514	294 - 303	550.3406	1098.6666	1098.6648	1.64	1	40	0.0014	1	U	K.KVEAEIVALK.V
5515	294 - 303	367.2300	1098.6680	1098.6648	2.93	1	19	0.15	1	U	K.KVEAEIVALK.V
3317	295 - 303	486.2926	970.5706	970.5699	0.78	0	3	0.58	1	U	K.VEAEIVALK.V
15351	313 - 327	815.8826	1629.7507	1629.7556	-2.99	0	40	0.00019	1	U	K.STNIVIMDSYSDSAK.T
15352	313 - 327	815.8857	1629.7568	1629.7556	0.74	0	65	8.1e-07	1	U	K.STNIVIMDSYSDSAK.T
15353	313 - 327	544.2598	1629.7577	1629.7556	1.27	0	6	0.3	1	U	K.STNIVIMDSYSDSAK.T
15565	313 - 327	823.8789	1645.7433	1645.7505	-4.40	0	50	2.2e-05	1	U	K.STNIVIMDSYSDSAK.T + Oxidation (M)
15566	313 - 327	823.8833	1645.7521	1645.7505	0.94	0	53	9.9e-06	1	U	K.STNIVIMDSYSDSAK.T + Oxidation (M)
1210	332 - 339	411.7398	821.4651	821.4647	0.47	0	42	0.00021	1	U	K.SLTGLGFK.N
1211	332 - 339	411.7404	821.4662	821.4647	1.83	0	1	0.74	1	U	K.SLTGLGFK.N
11029	340 - 352	691.3398	1380.6650	1380.6674	-1.70	0	31	0.0013	1	U	K.NSWILTDGFSGGK.G
11030	340 - 352	691.3408	1380.6671	1380.6674	-0.20	0	45	5.4e-05	1	U	K.NSWILTDGFSGGK.G
11050	340 - 352	691.8329	1381.6513	1381.6514	-0.049	0	14	0.052	1	U	K.NSWILTDGFSGGK.G + Deamidated (NQ)
339	353 - 358	373.7021	745.3897	745.3871	3.45	0	3	0.5	1	U	K.GWLQSR.L
19770	359 - 376	668.6612	2002.9616	2002.9636	-0.98	0	77	1.6e-07	1	U	R.LGTESYNLSFGEIFSPSR.I
19771	359 - 376	1002.4883	2002.9620	2002.9636	-0.80	0	85	9.7e-09	1	U	R.LGTESYNLSFGEIFSPSR.I
500	377 - 384	380.7190	759.4235	759.4239	-0.53	0	3	0.49	1	U	R.IISGGTGR.F
502	377 - 384	380.7192	759.4239	759.4239	-0.0079	0	5	0.59	1	U	R.IISGGTGR.F
8606	385 - 396	627.3262	1252.6379	1252.6412	-2.62	0	23	0.0068	1	U	R.FGTTSSTVQIGR.K
8607	385 - 396	627.3274	1252.6402	1252.6412	-0.77	0	79	4.3e-08	1	U	R.FGTTSSTVQIGR.K
8609	385 - 396	627.3297	1252.6449	1252.6412	2.95	0	65	8.7e-07	1	U	R.FGTTSSTVQIGR.K
11038	385 - 397	691.3744	1380.7343	1380.7361	-1.30	1	23	0.0069	1	U	R.FGTTSSTVQIGRK.M

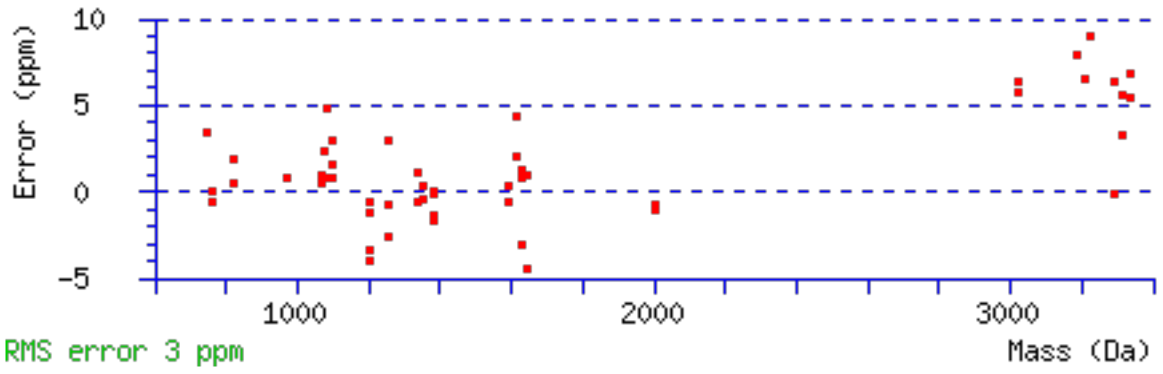

Mascot: http://www.matrixscience.com/

MATRIX SCIENCE MASCOT Search Results

Protein View: sp|P06511|PSAA_SPIOL

>sp|P06511|PSAA_SPIOL Photosystem I P700 chlorophyll a apoprotein A1 OS=Spinacia oleracea OX=3562 GN=psaA PE=3 SV=1

**Database:**	Uni-Spinach
**Score:**	1337
**Nominal mass (M_r):**	83161
**Calculated pI:**	6.74

Sequence similarity is available as **an NCBI BLAST search of sp|P06511|PSAA_SPIOL against nr**.

Search parameters

**MS data file:**	\\172.16.0.213\tank\windowsVM\Bill Cramer\052418\MGF\wc_QE_052418_Cramer_gel_4.mgf
**Enzyme:**	Trypsin: cuts C-term side of KR unless next residue is P.
**Fixed modifications:**	Carbamidomethyl (C)
**Variable modifications:**	Acetyl (K), Acetyl (Protein N-term), Deamidated (NQ), Oxidation (M)

Protein sequence coverage: 22%

Matched peptides shown in ***bold red***.

```
  1 MIIRSPEPEV KILVDRDPVK TSFEAWAKPG HFSRTIAKGP ETTTWIWNLH
 51 ADAHDFDSHT SDLEEISRKI FSAHFGQLSI IFLWLSGMYF HGARFSNYEA
101 WLSDPTHIGP SAQVVWPIVG QEILNGDVGG GFRGIQITSG FFQIWRASGI
151 TSELQLYCTA IGALVFAALM LFAGWFHYHK AAPKLAWFQD VESMLNHHLA
201 GLLGLGSLSW AGHQIHVSLP INQFLNAGVD PKEIPLPHEL ILNRDLLAQL
251 YPSFAEGATP FFTLNWSKYA DFLTFRGGLD PVTGGLWLTD TAHHHLAIAI
301 LFLIAGHMYR TNWGIGHGLK DILEAHKGPF TGQGHKGLYE ILTTSWHAQL
351 ALNLAMLGSL TIVVAHHMYA MPPYPYLATD YGTQLSLFTH HMWIGGFLIV
401 GAAAHAAIFM VRDYDPTTRY NDLLDRVLRH RDAIISHLNW ACIFLGFHSF
451 GLYIHNDTMS ALGRPQDMFS DTAIQLQPVF AQWIQNTHAL APSATAPGAT
501 ASTSLTWGGS DLVAVGGKVA LLPIPLGTAD FLVHHIHAFT IHVTVLILLK
551 GVLFARSSRL IPDKANLGFR FPCDGPGRGG TCQVSAWDHV FLGLFWMYNS
601 ISVVIFHFSW KMQSDVWGSI SDQGVVTHIT GGNFAQSSIT INGWLRDFLW
651 AQASQVIQSY GSSLSAYGLF FLGAHFVWAF SLMFLFSGRG YWQELIESIV
701 WAHNKLKVAP ATQPRALSIV QGRAVGVTHY LLGGIATTWA FFLARIIAVG
```

Unformatted sequence string: **750 residues** (for pasting into other applications).

Sort peptides by ◉ Residue Number ○ Increasing Mass ○ Decreasing Mass

Show predicted peptides also

Query	Start – End	Observed	Mr(expt)	Mr(calc)	ppm	M	Score	Expect	Rank	U	Peptide
7752	2 – 11	605.3460	1208.6775	1208.6765	0.89	1	34	0.00099	1	U	M.IIRSPEPEVK.I + Acetyl (Protein N-term)
7753	2 – 11	605.3465	1208.6785	1208.6765	1.72	1	39	0.00086	1	U	M.IIRSPEPEVK.I + Acetyl (Protein N-term)
769	5 – 11	393.2050	784.3955	784.3967	-1.48	0	2	0.61	1	U	R.SPEPEVK.I
770	5 – 11	393.2055	784.3965	784.3967	-0.17	0	17	0.044	1	U	R.SPEPEVK.I
17726	5 – 20	607.6749	1820.0030	1820.0043	-0.77	2	12	0.073	1	U	R.SPEPEVKILVDRDPVK.T
4529	12 – 20	527.8158	1053.6170	1053.6182	-1.14	1	34	0.0038	1	U	K.ILVDRDPVK.T
4533	12 – 20	352.2133	1053.6180	1053.6182	-0.24	1	4	0.44	1	U	K.ILVDRDPVK.T
4534	12 – 20	527.8163	1053.6181	1053.6182	-0.14	1	12	0.067	1	U	K.ILVDRDPVK.T
4535	12 – 20	352.2134	1053.6184	1053.6182	0.19	1	4	0.95	1	U	K.ILVDRDPVK.T
4537	12 – 20	352.2135	1053.6187	1053.6182	0.44	1	6	0.28	1	U	K.ILVDRDPVK.T
4539	12 – 20	352.2136	1053.6191	1053.6182	0.79	1	11	0.099	1	U	K.ILVDRDPVK.T
4541	12 – 20	527.8170	1053.6195	1053.6182	1.18	1	15	0.047	1	U	K.ILVDRDPVK.T
4543	12 – 20	527.8171	1053.6196	1053.6182	1.26	1	34	0.0024	1	U	K.ILVDRDPVK.T
4544	12 – 20	527.8172	1053.6198	1053.6182	1.45	1	34	0.0018	1	U	K.ILVDRDPVK.T
4545	12 – 20	352.2139	1053.6198	1053.6182	1.47	1	1	0.78	1	U	K.ILVDRDPVK.T
4546	12 – 20	352.2139	1053.6198	1053.6182	1.52	1	11	0.095	1	U	K.ILVDRDPVK.T
15214	21 – 34	810.8991	1619.7837	1619.7845	-0.47	0	69	3.2e-07	1	U	K.TSFEAWAKPGHFSR.T
15215	21 – 34	810.8994	1619.7843	1619.7845	-0.080	0	53	1.1e-05	1	U	K.TSFEAWAKPGHFSR.T
15216	21 – 34	540.9355	1619.7848	1619.7845	0.19	0	33	0.00087	1	U	K.TSFEAWAKPGHFSR.T
15217	21 – 34	405.9536	1619.7854	1619.7845	0.57	0	11	0.094	1	U	K.TSFEAWAKPGHFSR.T
15218	21 – 34	405.9536	1619.7855	1619.7845	0.62	0	26	0.0035	1	U	K.TSFEAWAKPGHFSR.T
15219	21 – 34	540.9358	1619.7857	1619.7845	0.76	0	27	0.0029	1	U	K.TSFEAWAKPGHFSR.T
15220	21 – 34	405.9537	1619.7858	1619.7845	0.80	0	9	0.15	1	U	K.TSFEAWAKPGHFSR.T
15221	21 – 34	405.9538	1619.7862	1619.7845	1.09	0	14	0.048	1	U	K.TSFEAWAKPGHFSR.T
15222	21 – 34	810.9005	1619.7865	1619.7845	1.28	0	71	2e-07	1	U	K.TSFEAWAKPGHFSR.T
15223	21 – 34	405.9540	1619.7868	1619.7845	1.41	0	17	0.024	1	U	K.TSFEAWAKPGHFSR.T

15224	21 - 34	405.9540	1619.7868	1619.7845	1.41	0	34	0.0007	1	U	K.TSFEAWAKPGHFSR.T
15225	21 - 34	405.9541	1619.7872	1619.7845	1.69	0	6	0.25	1	U	K.TSFEAWAKPGHFSR.T
15226	21 - 34	810.9009	1619.7872	1619.7845	1.70	0	38	0.00025	1	U	K.TSFEAWAKPGHFSR.T
15227	21 - 34	540.9364	1619.7873	1619.7845	1.72	0	46	5.4e-05	1	U	K.TSFEAWAKPGHFSR.T
15228	21 - 34	405.9542	1619.7876	1619.7845	1.94	0	32	0.0011	1	U	K.TSFEAWAKPGHFSR.T
15229	21 - 34	540.9368	1619.7887	1619.7845	2.59	0	23	0.0066	1	U	K.TSFEAWAKPGHFSR.T
15230	21 - 34	540.9368	1619.7887	1619.7845	2.62	0	44	8.1e-05	1	U	K.TSFEAWAKPGHFSR.T
24871	35 - 68	787.9827	3934.8771	3934.8398	9.48	1	50	1.8e-05	1	U	R.TIAKGPETTTWIWNLHADAHDFDSHTSDLEEISR.K + Acetyl (K)
24872	35 - 68	787.9828	3934.8775	3934.8398	9.59	1	28	0.0021	1	U	R.TIAKGPETTTWIWNLHADAHDFDSHTSDLEEISR.K + Acetyl (K)
14275	134 - 146	776.9176	1551.8206	1551.8198	0.50	0	90	3.6e-09	1	U	R.GIQITSGFFQIWR.A
14276	134 - 146	518.2816	1551.8231	1551.8198	2.11	0	64	9.1e-07	1	U	R.GIQITSGFFQIWR.A
4144	269 - 276	516.7615	1031.5085	1031.5076	0.88	0	49	2.8e-05	1	U	K.YADFLTFR.G
4145	269 - 276	516.7620	1031.5095	1031.5076	1.81	0	27	0.003	1	U	K.YADFLTFR.G
4146	269 - 276	516.7622	1031.5098	1031.5076	2.10	0	32	0.001	1	U	K.YADFLTFR.G
4147	269 - 276	516.7622	1031.5099	1031.5076	2.16	0	47	3.8e-05	1	U	K.YADFLTFR.G
4148	269 - 276	516.7635	1031.5124	1031.5076	4.65	0	11	0.097	1	U	K.YADFLTFR.G
5106	311 - 320	361.5294	1081.5663	1081.5669	-0.50	0	16	0.031	1	U	R.TNWGIGHGLK.D
5107	311 - 320	541.7905	1081.5665	1081.5669	-0.36	0	39	0.00021	1	U	R.TNWGIGHGLK.D
5108	311 - 320	361.5296	1081.5670	1081.5669	0.094	0	16	0.032	1	U	R.TNWGIGHGLK.D
5109	311 - 320	541.7910	1081.5675	1081.5669	0.53	0	38	0.00026	1	U	R.TNWGIGHGLK.D
5110	311 - 320	361.5307	1081.5704	1081.5669	3.21	0	17	0.025	1	U	R.TNWGIGHGLK.D
1243	321 - 327	413.2269	824.4393	824.4392	0.11	0	14	0.046	1	U	K.DILEAHK.G
1244	321 - 327	413.2274	824.4403	824.4392	1.33	0	12	0.082	1	U	K.DILEAHK.G
16655	321 - 336	578.9694	1733.8865	1733.8849	0.89	1	37	0.00037	1	U	K.DILEAHKGPFTGQGHK.G
16656	321 - 336	434.4789	1733.8865	1733.8849	0.89	1	36	0.00057	1	U	K.DILEAHKGPFTGQGHK.G
16657	321 - 336	578.9695	1733.8866	1733.8849	0.99	1	0	0.98	1	U	K.DILEAHKGPFTGQGHK.G
16658	321 - 336	578.9695	1733.8868	1733.8849	1.08	1	40	0.00017	1	U	K.DILEAHKGPFTGQGHK.G
16659	321 - 336	434.4791	1733.8874	1733.8849	1.44	1	34	0.00071	1	U	K.DILEAHKGPFTGQGHK.G
16660	321 - 336	867.9512	1733.8878	1733.8849	1.67	1	46	5.1e-05	1	U	K.DILEAHKGPFTGQGHK.G
2696	328 - 336	464.7354	927.4562	927.4563	-0.086	0	17	0.025	1	U	K.GPFTGQGHK.G
2697	328 - 336	464.7354	927.4563	927.4563	-0.0043	0	25	0.0042	1	U	K.GPFTGQGHK.G
1889	413 - 419	434.1954	866.3762	866.3770	-0.90	0	26	0.0033	1	U	R.DYDPTTR.Y
16913	413 - 426	878.9113	1755.8081	1755.8064	0.97	1	25	0.0056	1	U	R.DYDPTTRYNDLLDR.V
16914	413 - 426	586.2768	1755.8087	1755.8064	1.33	1	11	0.12	1	U	R.DYDPTTRYNDLLDR.V
16915	413 - 426	586.2771	1755.8094	1755.8064	1.72	1	13	0.058	1	U	R.DYDPTTRYNDLLDR.V
2455	420 - 426	454.7270	907.4395	907.4399	-0.49	0	56	6.9e-05	1	U	R.YNDLLDR.V
2456	420 - 426	454.7271	907.4396	907.4399	-0.31	0	51	0.00024	1	U	R.YNDLLDR.V
2457	420 - 426	454.7271	907.4397	907.4399	-0.29	0	56	6.7e-05	1	U	R.YNDLLDR.V
2458	420 - 426	454.7271	907.4397	907.4399	-0.22	0	51	0.0002	1	U	R.YNDLLDR.V
2459	420 - 426	454.7272	907.4398	907.4399	-0.11	0	56	7e-05	1	U	R.YNDLLDR.V
2460	420 - 426	454.7272	907.4398	907.4399	-0.093	0	38	0.0035	1	U	R.YNDLLDR.V
2461	420 - 426	454.7278	907.4411	907.4399	1.32	0	37	0.0055	1	U	R.YNDLLDR.V
2471	420 - 426	455.2193	908.4240	908.4239	0.063	0	50	0.00014	1	U	R.YNDLLDR.V + Deamidated (NQ)
2472	420 - 426	455.2195	908.4244	908.4239	0.54	0	55	4.4e-05	1	U	R.YNDLLDR.V + Deamidated (NQ)
2473	420 - 426	455.2197	908.4248	908.4239	0.95	0	33	0.0058	1	U	R.YNDLLDR.V + Deamidated (NQ)
9057	420 - 429	426.2383	1275.6932	1275.6935	-0.27	1	17	0.027	1	U	R.YNDLLDRVLR.H
8443	560 - 570	622.3616	1242.7087	1242.7084	0.22	1	29	0.0021	1	U	R.LIPDKANLGFR.F
8444	560 - 570	415.2436	1242.7089	1242.7084	0.34	1	38	0.00028	1	U	R.LIPDKANLGFR.F
8445	560 - 570	622.3617	1242.7089	1242.7084	0.36	1	41	0.00014	1	U	R.LIPDKANLGFR.F
8446	560 - 570	415.2438	1242.7095	1242.7084	0.82	1	45	5.7e-05	1	U	R.LIPDKANLGFR.F
8447	560 - 570	622.3623	1242.7100	1242.7084	1.28	1	23	0.007	1	U	R.LIPDKANLGFR.F
8465	560 - 570	622.8536	1243.6926	1243.6924	0.13	1	27	0.0028	1	U	R.LIPDKANLGFR.F + Deamidated (NQ)
14442	565 - 578	521.9217	1562.7432	1562.7412	1.27	1	15	0.04	1	U	K.ANLGFRFPCDGPGR.G
2410	571 - 578	453.1996	904.3846	904.3862	-1.69	0	21	0.012	1	U	R.FPCDGPGR.G
2411	571 - 578	453.2000	904.3855	904.3862	-0.72	0	29	0.0019	1	U	R.FPCDGPGR.G
2412	571 - 578	453.2001	904.3856	904.3862	-0.57	0	29	0.0017	1	U	R.FPCDGPGR.G
2413	571 - 578	453.2002	904.3858	904.3862	-0.35	0	26	0.0037	1	U	R.FPCDGPGR.G
2414	571 - 578	453.2003	904.3861	904.3862	-0.024	0	14	0.054	1	U	R.FPCDGPGR.G
2415	571 - 578	453.2004	904.3863	904.3862	0.12	0	32	0.00099	1	U	R.FPCDGPGR.G
2416	571 - 578	453.2006	904.3866	904.3862	0.52	0	35	0.00048	1	U	R.FPCDGPGR.G
2417	571 - 578	453.2011	904.3877	904.3862	1.68	0	25	0.0043	1	U	R.FPCDGPGR.G
5082	706 - 715	360.8889	1079.6449	1079.6451	-0.16	1	20	0.041	1	U	K.LKVAPATQPR.A
5083	706 - 715	540.8304	1079.6463	1079.6451	1.11	1	15	0.15	2	U	K.LKVAPATQPR.A
1445	708 - 715	420.2398	838.4651	838.4661	-1.16	0	17	0.07	1	U	K.VAPATQPR.A
1446	708 - 715	420.2399	838.4653	838.4661	-0.99	0	6	0.29	1	U	K.VAPATQPR.A
1584	716 - 723	422.2562	842.4978	842.4974	0.45	0	11	0.36	1	U	R.ALSIVQGR.A
1585	716 - 723	422.2563	842.4981	842.4974	0.89	0	19	0.093	1	U	R.ALSIVQGR.A
1587	716 - 723	422.2568	842.4991	842.4974	2.01	0	33	0.013	1	U	R.ALSIVQGR.A

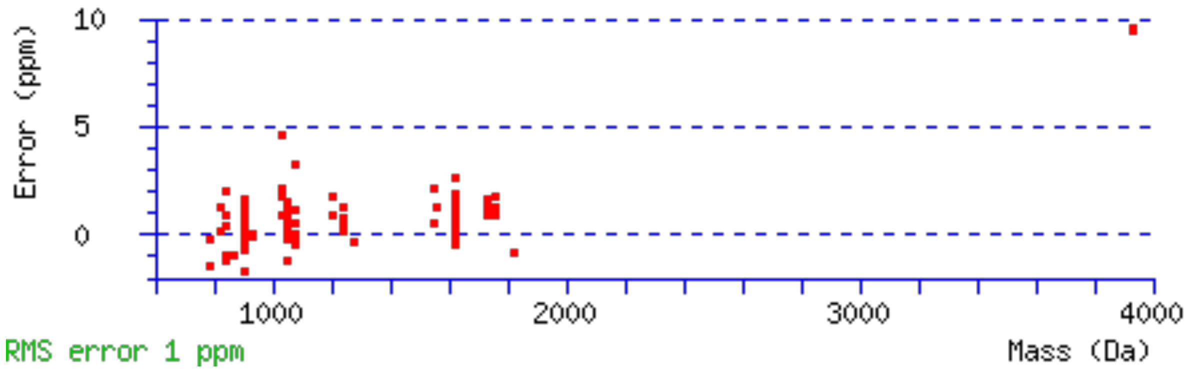

Mascot: http://www.matrixscience.com/

MASCOT Search Results

Protein View: tr|A0A0K9RHN1|A0A0K9RHN1_SPIOL

>tr|A0A0K9RHN1|A0A0K9RHN1_SPIOL Uncharacterized protein OS=Spinacia oleracea OX=3562 GN=SOVF_065780 PE=4 SV=1

**Database:**	Uni-Spinach
**Score:**	1094
**Nominal mass (M_r):**	13359
**Calculated pI:**	9.99

Sequence similarity is available as an NCBI BLAST search of tr|A0A0K9RHN1|A0A0K9RHN1_SPIOL against nr.

Search parameters

**MS data file:**	\\172.16.0.213\tank\windowsVM\Bill Cramer\052418\MGF\wc_QE_052418_Cramer_gel_4.mgf
**Enzyme:**	Trypsin: cuts C-term side of KR unless next residue is P.
**Fixed modifications:**	Carbamidomethyl (C)
**Variable modifications:**	Acetyl (K), Acetyl (Protein N-term), Deamidated (NQ), Oxidation (M)

Protein sequence coverage: 74%

Matched peptides shown in ***bold red***.

```
  1 MASIASSVAV RLGLTQVLPN KNFSSPRSTR LVVRAAEEAA AAPAAASPEG
 51 EAPKAAAKPP PIGPKRGSKV RIMRKESYWY KGVGSVVAVD QDPKTRYPVV
101 VRFNKVNYAN VSTNNYALDE IQEVA
```

Unformatted sequence string: 125 residues (for pasting into other applications).

Sort peptides by ◉ Residue Number ○ Increasing Mass ○ Decreasing Mass

Show predicted peptides also

Query	Start – End	Observed	Mr(expt)	Mr(calc)	ppm	M	Score	Expect	Rank	U	Peptide
5189	12 – 21	542.8201	1083.6257	1083.6176	7.52	0	0	0.9	1	U	R.LGLTQVLPNK.N + 2 Deamidated (NQ)
17546	35 – 54	904.9356	1807.8566	1807.8587	-1.21	0	56	5.2e-06	1	U	R.AAEEAAAAPAAASPEGEAPK.A
17547	35 – 54	904.9360	1807.8574	1807.8587	-0.75	0	45	6.1e-05	1	U	R.AAEEAAAAPAAASPEGEAPK.A
17548	35 – 54	904.9369	1807.8593	1807.8587	0.32	0	58	4e-06	1	U	R.AAEEAAAAPAAASPEGEAPK.A
17549	35 – 54	603.6274	1807.8604	1807.8587	0.89	0	74	1.2e-07	1	U	R.AAEEAAAAPAAASPEGEAPK.A
17550	35 – 54	603.6275	1807.8606	1807.8587	1.03	0	52	1.5e-05	1	U	R.AAEEAAAAPAAASPEGEAPK.A
17551	35 – 54	603.6278	1807.8617	1807.8587	1.61	0	73	1.3e-07	1	U	R.AAEEAAAAPAAASPEGEAPK.A
17552	35 – 54	603.6279	1807.8619	1807.8587	1.73	0	41	0.00013	1	U	R.AAEEAAAAPAAASPEGEAPK.A
17553	35 – 54	904.9384	1807.8623	1807.8587	1.95	0	48	3.3e-05	1	U	R.AAEEAAAAPAAASPEGEAPK.A
17554	35 – 54	904.9386	1807.8626	1807.8587	2.15	0	55	7.4e-06	1	U	R.AAEEAAAAPAAASPEGEAPK.A
4370	55 – 65	523.8214	1045.6282	1045.6284	-0.13	0	41	0.00024	1	U	K.AAAKPPPIGPK.R
4371	55 – 65	523.8215	1045.6285	1045.6284	0.10	0	43	0.00017	1	U	K.AAAKPPPIGPK.R
4372	55 – 65	523.8217	1045.6288	1045.6284	0.41	0	22	0.015	1	U	K.AAAKPPPIGPK.R
4373	55 – 65	523.8217	1045.6288	1045.6284	0.44	0	22	0.018	1	U	K.AAAKPPPIGPK.R
4374	55 – 65	523.8217	1045.6289	1045.6284	0.48	0	33	0.0015	1	U	K.AAAKPPPIGPK.R
4375	55 – 65	523.8218	1045.6290	1045.6284	0.54	0	33	0.0015	1	U	K.AAAKPPPIGPK.R
4376	55 – 65	523.8219	1045.6292	1045.6284	0.76	0	23	0.012	1	U	K.AAAKPPPIGPK.R
4377	55 – 65	523.8220	1045.6294	1045.6284	0.94	0	30	0.0025	1	U	K.AAAKPPPIGPK.R
4378	55 – 65	523.8221	1045.6297	1045.6284	1.27	0	18	0.023	1	U	K.AAAKPPPIGPK.R
4379	55 – 65	523.8223	1045.6301	1045.6284	1.61	0	20	0.017	1	U	K.AAAKPPPIGPK.R
7619	55 – 66	401.5832	1201.7279	1201.7295	-1.32	1	34	0.0006	1	U	K.AAAKPPPIGPKR.G
7620	55 – 66	401.5838	1201.7296	1201.7295	0.13	1	13	0.055	1	U	K.AAAKPPPIGPKR.G
7621	55 – 66	601.8725	1201.7304	1201.7295	0.79	1	6	0.68	1	U	K.AAAKPPPIGPKR.G
3718	75 – 81	502.2480	1002.4815	1002.4810	0.46	1	30	0.032	1	U	R.KESYWYK.G
3719	75 – 81	502.2484	1002.4823	1002.4810	1.26	1	4	0.38	1	U	R.KESYWYK.G
21550	75 – 94	752.3837	2254.1294	2254.1270	1.07	2	39	0.00021	1	U	R.KESYWYKGVGSVVAVDQDPK.T
20618	76 – 94	1064.0231	2126.0316	2126.0320	-0.21	1	84	1.4e-08	1	U	K.ESYWYKGVGSVVAVDQDPK.T
20619	76 – 94	709.6855	2126.0346	2126.0320	1.22	1	38	0.00026	1	U	K.ESYWYKGVGSVVAVDQDPK.T
20620	76 – 94	709.6858	2126.0355	2126.0320	1.64	1	35	0.00058	1	U	K.ESYWYKGVGSVVAVDQDPK.T
8901	82 – 94	635.8350	1269.6555	1269.6565	-0.79	0	27	0.011	1	U	K.GVGSVVAVDQDPK.T
8902	82 – 94	424.2261	1269.6564	1269.6565	-0.079	0	27	0.0032	1	U	K.GVGSVVAVDQDPK.T
8903	82 – 94	424.2262	1269.6568	1269.6565	0.24	0	9	0.13	1	U	K.GVGSVVAVDQDPK.T
8904	82 – 94	635.8357	1269.6568	1269.6565	0.25	0	37	0.00096	1	U	K.GVGSVVAVDQDPK.T

8905	82 – 94	635.8357	1269.6569	1269.6565	0.33	0	53	5.1e-05	1	U	K.GVGSVVAVDQDPK.T
13872	82 – 96	764.4109	1526.8072	1526.8053	1.26	1	59	2.8e-06	1	U	K.GVGSVVAVDQDPKTR.Y
13873	82 – 96	509.9440	1526.8102	1526.8053	3.20	1	4	0.47	1	U	K.GVGSVVAVDQDPKTR.Y
13874	82 – 96	509.9440	1526.8102	1526.8053	3.21	1	10	0.13	1	U	K.GVGSVVAVDQDPKTR.Y
21434	82 – 102	747.7502	2240.2287	2240.2278	0.41	2	17	0.025	1	U	K.GVGSVVAVDQDPKTRYPVVVR.F
21435	82 – 102	561.0653	2240.2320	2240.2278	1.89	2	17	0.025	1	U	K.GVGSVVAVDQDPKTRYPVVVR.F
21440	82 – 102	748.0824	2241.2254	2241.2118	6.07	2	15	0.041	1	U	K.GVGSVVAVDQDPKTRYPVVVR.F + Deamidated (NQ)
3552	95 – 102	495.2985	988.5825	988.5818	0.67	1	11	0.28	1	U	K.TRYPVVVR.F
3553	95 – 102	495.2988	988.5831	988.5818	1.33	1	31	0.0074	1	U	K.TRYPVVVR.F
3554	95 – 102	495.2989	988.5832	988.5818	1.46	1	30	0.01	1	U	K.TRYPVVVR.F
278	97 – 102	366.7238	731.4331	731.4330	0.14	0	26	0.028	1	U	R.YPVVVR.F
279	97 – 102	366.7241	731.4336	731.4330	0.82	0	24	0.038	1	U	R.YPVVVR.F
280	97 – 102	366.7242	731.4337	731.4330	1.00	0	23	0.029	1	U	R.YPVVVR.F
281	97 – 102	366.7242	731.4338	731.4330	1.09	0	31	0.0087	1	U	R.YPVVVR.F
23533	103 – 125	872.7565	2615.2477	2615.2503	-1.02	1	87	7.7e-09	1	U	R.FNKVNYANVSTNNYALDEIQEVA.-
23534	103 – 125	1308.6324	2615.2503	2615.2503	-0.012	1	68	4.3e-07	1	U	R.FNKVNYANVSTNNYALDEIQEVA.-
23540	103 – 125	873.0854	2616.2342	2616.2343	-0.034	1	28	0.0024	1	U	R.FNKVNYANVSTNNYALDEIQEVA.- + Deamidated (NQ)
23542	103 – 125	873.0909	2616.2508	2616.2343	6.30	1	19	0.016	1	U	R.FNKVNYANVSTNNYALDEIQEVA.- + Deamidated (NQ)
23543	103 – 125	1309.1340	2616.2535	2616.2343	7.33	1	114	2.2e-11	1	U	R.FNKVNYANVSTNNYALDEIQEVA.- + Deamidated (NQ)
21286	106 – 125	1114.0284	2226.0422	2226.0440	-0.80	0	38	0.00029	1	U	K.VNYANVSTNNYALDEIQEVA.-
21287	106 – 125	1114.0315	2226.0484	2226.0440	1.95	0	27	0.0028	1	U	K.VNYANVSTNNYALDEIQEVA.-
21306	106 – 125	1114.5229	2227.0313	2227.0280	1.48	0	24	0.0059	1	U	K.VNYANVSTNNYALDEIQEVA.- + Deamidated (NQ)
21308	106 – 125	743.3517	2227.0332	2227.0280	2.34	0	17	0.027	1	U	K.VNYANVSTNNYALDEIQEVA.- + Deamidated (NQ)
21310	106 – 125	1114.5243	2227.0340	2227.0280	2.70	0	27	0.0029	1	U	K.VNYANVSTNNYALDEIQEVA.- + Deamidated (NQ)

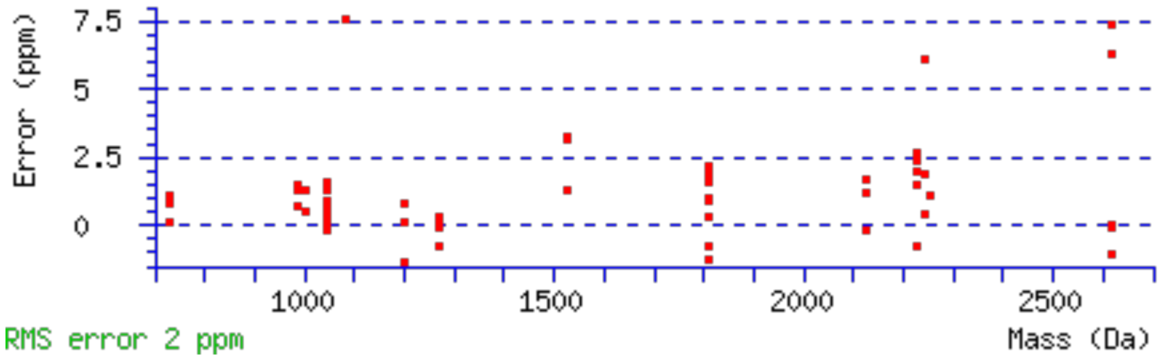

RMS error 2 ppm

Mascot: http://www.matrixscience.com/

MASCOT Search Results

Protein View: tr|A0A0K9RHN1|A0A0K9RHN1_SPIOL

>tr|A0A0K9RHN1|A0A0K9RHN1_SPIOL Uncharacterized protein OS=Spinacia oleracea OX=3562 GN=SOVF_065780 PE=4 SV=1

**Database:**	Uni-Spinach
**Score:**	1094
**Nominal mass (M_r):**	13359
**Calculated pI:**	9.99

Sequence similarity is available as an NCBI BLAST search of tr|A0A0K9RHN1|A0A0K9RHN1_SPIOL against nr.

Search parameters

**MS data file:**	\\172.16.0.213\tank\windowsVM\Bill Cramer\052418\MGF\wc_QE_052418_Cramer_gel_4.mgf
**Enzyme:**	Trypsin: cuts C-term side of KR unless next residue is P.
**Fixed modifications:**	Carbamidomethyl (C)
**Variable modifications:**	Acetyl (K), Acetyl (Protein N-term), Deamidated (NQ), Oxidation (M)

Protein sequence coverage: 74%

Matched peptides shown in ***bold red***.

```
  1 MASIASSVAV RLGLTQVLPN KNFSSPRSTR LVVRAAEEAA AAPAAASPEG
 51 EAPKAAAKPP PIGPKRGSKV RIMRKESYWY KGVGSVVAVD QDPKTRYPVV
101 VRFNKVNYAN VSTNNYALDE IQEVA
```

Unformatted sequence string: 125 residues (for pasting into other applications).

Sort peptides by ◉ Residue Number ○ Increasing Mass ○ Decreasing Mass

Show predicted peptides also

Query	Start – End	Observed	Mr(expt)	Mr(calc)	ppm	M	Score	Expect	Rank	U	Peptide
5189	12 – 21	542.8201	1083.6257	1083.6176	7.52	0	0	0.9	1	U	R.LGLTQVLPNK.N + 2 Deamidated (NQ)
17546	35 – 54	904.9356	1807.8566	1807.8587	-1.21	0	56	5.2e-06	1	U	R.AAEEAAAAPAAASPEGEAPK.A
17547	35 – 54	904.9360	1807.8574	1807.8587	-0.75	0	45	6.1e-05	1	U	R.AAEEAAAAPAAASPEGEAPK.A
17548	35 – 54	904.9369	1807.8593	1807.8587	0.32	0	58	4e-06	1	U	R.AAEEAAAAPAAASPEGEAPK.A
17549	35 – 54	603.6274	1807.8604	1807.8587	0.89	0	74	1.2e-07	1	U	R.AAEEAAAAPAAASPEGEAPK.A
17550	35 – 54	603.6275	1807.8606	1807.8587	1.03	0	52	1.5e-05	1	U	R.AAEEAAAAPAAASPEGEAPK.A
17551	35 – 54	603.6278	1807.8617	1807.8587	1.61	0	73	1.3e-07	1	U	R.AAEEAAAAPAAASPEGEAPK.A
17552	35 – 54	603.6279	1807.8619	1807.8587	1.73	0	41	0.00013	1	U	R.AAEEAAAAPAAASPEGEAPK.A
17553	35 – 54	904.9384	1807.8623	1807.8587	1.95	0	48	3.3e-05	1	U	R.AAEEAAAAPAAASPEGEAPK.A
17554	35 – 54	904.9386	1807.8626	1807.8587	2.15	0	55	7.4e-06	1	U	R.AAEEAAAAPAAASPEGEAPK.A
4370	55 – 65	523.8214	1045.6282	1045.6284	-0.13	0	41	0.00024	1	U	K.AAAKPPPIGPK.R
4371	55 – 65	523.8215	1045.6285	1045.6284	0.10	0	43	0.00017	1	U	K.AAAKPPPIGPK.R
4372	55 – 65	523.8217	1045.6288	1045.6284	0.41	0	22	0.015	1	U	K.AAAKPPPIGPK.R
4373	55 – 65	523.8217	1045.6288	1045.6284	0.44	0	22	0.018	1	U	K.AAAKPPPIGPK.R
4374	55 – 65	523.8217	1045.6289	1045.6284	0.48	0	33	0.0015	1	U	K.AAAKPPPIGPK.R
4375	55 – 65	523.8218	1045.6290	1045.6284	0.54	0	33	0.0015	1	U	K.AAAKPPPIGPK.R
4376	55 – 65	523.8219	1045.6292	1045.6284	0.76	0	23	0.012	1	U	K.AAAKPPPIGPK.R
4377	55 – 65	523.8220	1045.6294	1045.6284	0.94	0	30	0.0025	1	U	K.AAAKPPPIGPK.R
4378	55 – 65	523.8221	1045.6297	1045.6284	1.27	0	18	0.023	1	U	K.AAAKPPPIGPK.R
4379	55 – 65	523.8223	1045.6301	1045.6284	1.61	0	20	0.017	1	U	K.AAAKPPPIGPK.R
7619	55 – 66	401.5832	1201.7279	1201.7295	-1.32	1	34	0.0006	1	U	K.AAAKPPPIGPKR.G
7620	55 – 66	401.5838	1201.7296	1201.7295	0.13	1	13	0.055	1	U	K.AAAKPPPIGPKR.G
7621	55 – 66	601.8725	1201.7304	1201.7295	0.79	1	6	0.68	1	U	K.AAAKPPPIGPKR.G
3718	75 – 81	502.2480	1002.4815	1002.4810	0.46	1	30	0.032	1	U	R.KESYWYK.G
3719	75 – 81	502.2484	1002.4823	1002.4810	1.26	1	4	0.38	1	U	R.KESYWYK.G
21550	75 – 94	752.3837	2254.1294	2254.1270	1.07	2	39	0.00021	1	U	R.KESYWYKGVGSVVAVDQDPK.T
20618	76 – 94	1064.0231	2126.0316	2126.0320	-0.21	1	84	1.4e-08	1	U	K.ESYWYKGVGSVVAVDQDPK.T
20619	76 – 94	709.6855	2126.0346	2126.0320	1.22	1	38	0.00026	1	U	K.ESYWYKGVGSVVAVDQDPK.T
20620	76 – 94	709.6858	2126.0355	2126.0320	1.64	1	35	0.00058	1	U	K.ESYWYKGVGSVVAVDQDPK.T
8901	82 – 94	635.8350	1269.6555	1269.6565	-0.79	0	27	0.011	1	U	K.GVGSVVAVDQDPK.T
8902	82 – 94	424.2261	1269.6564	1269.6565	-0.079	0	27	0.0032	1	U	K.GVGSVVAVDQDPK.T
8903	82 – 94	424.2262	1269.6568	1269.6565	0.24	0	9	0.13	1	U	K.GVGSVVAVDQDPK.T
8904	82 – 94	635.8357	1269.6568	1269.6565	0.25	0	37	0.00096	1	U	K.GVGSVVAVDQDPK.T

8905	82 – 94	635.8357	1269.6569	1269.6565	0.33	0	53	5.1e-05	1	U	K.GVGSVVAVDQDPK.T
13872	82 – 96	764.4109	1526.8072	1526.8053	1.26	1	59	2.8e-06	1	U	K.GVGSVVAVDQDPKTR.Y
13873	82 – 96	509.9440	1526.8102	1526.8053	3.20	1	4	0.47	1	U	K.GVGSVVAVDQDPKTR.Y
13874	82 – 96	509.9440	1526.8102	1526.8053	3.21	1	10	0.13	1	U	K.GVGSVVAVDQDPKTR.Y
21434	82 – 102	747.7502	2240.2287	2240.2278	0.41	2	17	0.025	1	U	K.GVGSVVAVDQDPKTRYPVVVR.F
21435	82 – 102	561.0653	2240.2320	2240.2278	1.89	2	17	0.025	1	U	K.GVGSVVAVDQDPKTRYPVVVR.F
21440	82 – 102	748.0824	2241.2254	2241.2118	6.07	2	15	0.041	1	U	K.GVGSVVAVDQDPKTRYPVVVR.F + Deamidated (NQ)
3552	95 – 102	495.2985	988.5825	988.5818	0.67	1	11	0.28	1	U	K.TRYPVVVR.F
3553	95 – 102	495.2988	988.5831	988.5818	1.33	1	31	0.0074	1	U	K.TRYPVVVR.F
3554	95 – 102	495.2989	988.5832	988.5818	1.46	1	30	0.01	1	U	K.TRYPVVVR.F
278	97 – 102	366.7238	731.4331	731.4330	0.14	0	26	0.028	1	U	R.YPVVVR.F
279	97 – 102	366.7241	731.4336	731.4330	0.82	0	24	0.038	1	U	R.YPVVVR.F
280	97 – 102	366.7242	731.4337	731.4330	1.00	0	23	0.029	1	U	R.YPVVVR.F
281	97 – 102	366.7242	731.4338	731.4330	1.09	0	31	0.0087	1	U	R.YPVVVR.F
23533	103 – 125	872.7565	2615.2477	2615.2503	-1.02	1	87	7.7e-09	1	U	R.FNKVNYANVSTNNYALDEIQEVA.-
23534	103 – 125	1308.6324	2615.2503	2615.2503	-0.012	1	68	4.3e-07	1	U	R.FNKVNYANVSTNNYALDEIQEVA.-
23540	103 – 125	873.0854	2616.2342	2616.2343	-0.034	1	28	0.0024	1	U	R.FNKVNYANVSTNNYALDEIQEVA.- + Deamidated (NQ)
23542	103 – 125	873.0909	2616.2508	2616.2343	6.30	1	19	0.016	1	U	R.FNKVNYANVSTNNYALDEIQEVA.- + Deamidated (NQ)
23543	103 – 125	1309.1340	2616.2535	2616.2343	7.33	1	114	2.2e-11	1	U	R.FNKVNYANVSTNNYALDEIQEVA.- + Deamidated (NQ)
21286	106 – 125	1114.0284	2226.0422	2226.0440	-0.80	0	38	0.00029	1	U	K.VNYANVSTNNYALDEIQEVA.-
21287	106 – 125	1114.0315	2226.0484	2226.0440	1.95	0	27	0.0028	1	U	K.VNYANVSTNNYALDEIQEVA.-
21306	106 – 125	1114.5229	2227.0313	2227.0280	1.48	0	24	0.0059	1	U	K.VNYANVSTNNYALDEIQEVA.- + Deamidated (NQ)
21308	106 – 125	743.3517	2227.0332	2227.0280	2.34	0	17	0.027	1	U	K.VNYANVSTNNYALDEIQEVA.- + Deamidated (NQ)
21310	106 – 125	1114.5243	2227.0340	2227.0280	2.70	0	27	0.0029	1	U	K.VNYANVSTNNYALDEIQEVA.- + Deamidated (NQ)

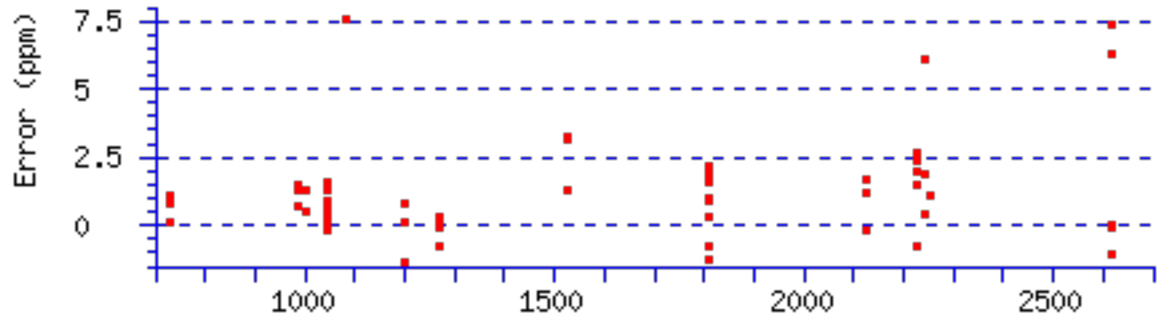

RMS error 2 ppm

Mascot: http://www.matrixscience.com/

MASCOT Search Results

Protein View: tr|A0A0K9QTC9|A0A0K9QTC9_SPIOL

>tr|A0A0K9QTC9|A0A0K9QTC9_SPIOL UVR domain-containing protein OS=Spinacia oleracea OX=3562 GN=SOVF_143680 PE=3 SV=1

**Database:**	Uni-Spinach
**Score:**	1066
**Nominal mass (M_r):**	102277
**Calculated pI:**	6.06

Sequence similarity is available as **an NCBI BLAST search of tr|A0A0K9QTC9|A0A0K9QTC9_SPIOL against nr**.

Search parameters

**MS data file:**	\\172.16.0.213\tank\windowsVM\Bill Cramer\052418\MGF\wc_QE_052418_Cramer_gel_4.mgf
**Enzyme:**	Trypsin: cuts C-term side of KR unless next residue is P.
**Fixed modifications:**	Carbamidomethyl (C)
**Variable modifications:**	Acetyl (K), Acetyl (Protein N-term), Deamidated (NQ), Oxidation (M)

Protein sequence coverage: 44%

Matched peptides shown in ***bold red***.

```
  1 MAGALIQSTN IPSLAYKVNG QFQRSDKGKK AVKMMASLQA PGYRMRSFSG
 51 LRGGNALDTL GTTSECFYSK MRAVLSVRKG TASRGVVRAM FERFTEKAIK
101 VIMLAQEEAR RLGHNFVGTE QILLGLIGEG TGIAAKVLKS MGINLKDARV
151 EVEKIIGRGS GFVAVEIPFT PRAKRVLELS LEEARQLGHN YIGSEHLLLG
201 LLREGEGVAA RVLENLGADP SNIRTQVIRM VGENTEAVGA GVGGGTTGNK
251 MPTLEEYGTN LTKLAEEGKL DPVVGRQAQI ERVTQILGRR TKNNPCLIGE
301 PGVGKTAIAE GLAQRIATGD VPETIEGKKV ITLDMGLLVA GTKYRGEFEE
351 RLKKLMEEIK QSDEIILFID EVHTLIGAGA AEGAIDAANI LKPALARGEL
401 QCIGATTLDE YRKHIEKDPA LERRFQPVKV PEPTVDETIQ ILKGLRERYE
451 IHHKLRYTDE ALVAAAQLSY QYISDRFLPD KAIDLIDEAG SRVRLQHAQL
501 PEEARELEKE LRQLTKEKNE AVRGQDFEKA GELRDREMDL KAQISALVEK
551 NKEMSKAETE AGDVGPMVTE SDIQHIVSSW TGIPVEKVST DESDRLLKME
601 DTLHTRVIGQ DEAVKAISRA IRRARVGLKN PNRPIASFIF SGPTGVGKSE
651 LAKALAAYYF GSEEAMIRLD MSEFMERHTV SKLIGSPPGY VGYTEGGQLT
701 EAVRRRPYTV VLFDEIEKAH PDVFNMMLQI LEDGRLTDSK GRTVDFKNTL
751 LIMTSNVGSS VIEKGGRRIG FDLDYDEKDS SYNRIKSLVT EELKQYFRPE
801 FLNRLDEMIV FRQLTKLEVK EIADIMLKEV FGRLKNKEIE LQVTERFRDR
851 VVDEGYNPSY GARPLRRAIM RLLEDSMAEK MLAREIKEGD SVIVDVDSDG
901 NVIVLNGSSG APPESLPEVL TV
```

Unformatted sequence string: **922 residues** (for pasting into other applications).

Sort peptides by ◉ Residue Number ○ Increasing Mass ○ Decreasing Mass

Show predicted peptides also

Query	Start – End	Observed	Mr(expt)	Mr(calc)	ppm	M	Score	Expect	Rank	U	Peptide
6659	101 – 110	580.3123	1158.6100	1158.6067	2.88	0	15	0.042	1	U	K.VIMLAQEEAR.R
515	140 – 146	381.7131	761.4116	761.4105	1.39	0	0	1.2	3	U	K.SMGINLK.D
12965	159 – 172	738.8990	1475.7835	1475.7773	4.24	0	54	8.3e-06	1	U	R.GSGFVAVEIPFTPR.A
9752	175 – 185	438.9174	1313.7304	1313.7303	0.062	1	51	0.00015	1	U	K.RVLELSLEEAR.Q
6643	176 – 185	579.8223	1157.6300	1157.6292	0.69	0	44	0.00018	1	U	R.VLELSLEEAR.Q
6644	176 – 185	579.8228	1157.6310	1157.6292	1.59	0	30	0.0017	1	U	R.VLELSLEEAR.Q
11367	212 – 224	699.3727	1396.7308	1396.7310	-0.18	0	41	0.00013	1	U	R.VLENLGADPSNIR.T
11368	212 – 224	699.3730	1396.7314	1396.7310	0.25	0	35	0.00048	1	U	R.VLENLGADPSNIR.T
18861	230 – 250	953.4524	1904.8902	1904.8898	0.18	0	116	1.4e-11	1	U	R.MVGENTEAVGAGVGGGTTGNK.M
18862	230 – 250	635.9711	1904.8914	1904.8898	0.84	0	65	8.4e-07	1	U	R.MVGENTEAVGAGVGGGTTGNK.M
13327	251 – 263	748.8683	1495.7220	1495.7228	-0.56	0	49	2.3e-05	1	U	K.MPTLEEYGTNLTK.L
13328	251 – 263	748.8687	1495.7228	1495.7228	0.017	0	49	2.7e-05	1	U	K.MPTLEEYGTNLTK.L
13623	251 – 263	756.8658	1511.7171	1511.7177	-0.42	0	41	0.00014	1	U	K.MPTLEEYGTNLTK.L + Oxidation (M)
11058	264 – 276	691.8871	1381.7596	1381.7565	2.25	1	64	1e-06	1	U	K.LAEEGKLDPVVGR.Q
11059	264 – 276	461.5939	1381.7598	1381.7565	2.37	1	58	3.4e-06	1	U	K.LAEEGKLDPVVGR.Q
793	283 – 289	393.7455	785.4765	785.4759	0.76	0	29	0.044	1	U	R.VTQILGR.R
4100	306 – 315	515.2881	1028.5616	1028.5614	0.18	0	28	0.0033	1	U	K.TAIAEGLAQR.I
4101	306 – 315	515.2887	1028.5629	1028.5614	1.46	0	38	0.0013	1	U	K.TAIAEGLAQR.I
10046	316 – 328	665.3471	1328.6797	1328.6824	-2.03	0	47	4.2e-05	1	U	R.IATGDVPETIEGK.K
12577	316 – 329	729.3939	1456.7732	1456.7773	-2.85	1	16	0.029	1	U	R.IATGDVPETIEGKK.V
12578	316 – 329	486.5997	1456.7773	1456.7773	-0.0034	1	15	0.04	1	U	R.IATGDVPETIEGKK.V

5196	344 - 351	543.2543	1084.4941	1084.4937	0.34	1	3	0.52	1	U	K.YRGEFEER.L
7708	414 - 423	604.3256	1206.6367	1206.6357	0.84	1	31	0.0012	1	U	K.HIEKDPALER.R
7709	414 - 423	403.2195	1206.6367	1206.6357	0.88	1	3	1.1	2	U	K.HIEKDPALER.R
14700	430 - 443	527.9622	1580.8648	1580.8661	-0.83	0	31	0.0011	1	U	K.VPEPTVDETIQILK.G
14701	430 - 443	791.4416	1580.8686	1580.8661	1.56	0	41	0.00013	1	U	K.VPEPTVDETIQILK.G
21739	457 - 476	759.7057	2276.0954	2276.0961	-0.28	0	105	1.5e-10	1	U	R.YTDEALVAAAQLSYQYISDR.F
6656	482 - 492	580.3014	1158.5882	1158.5880	0.11	0	31	0.0048	1	U	K.AIDLIDEAGSR.V
9314	495 - 505	431.2307	1290.6702	1290.6680	1.71	0	21	0.011	1	U	R.LQHAQLPEEAR.E
17310	495 - 509	597.6514	1789.9325	1789.9322	0.14	1	26	0.0034	1	U	R.LQHAQLPEEARELEK.E
12064	530 - 541	478.2479	1431.7218	1431.7140	5.46	2	2	0.69	1	U	K.AGELRDREMDLK.A
3122	542 - 550	479.7821	957.5496	957.5495	0.13	0	7	0.21	1	U	K.AQISALVEK.N
3125	542 - 550	479.7827	957.5508	957.5495	1.44	0	32	0.00092	1	U	K.AQISALVEK.N
24520	557 - 587	1095.2020	3282.5842	3282.5602	7.32	0	30	0.0014	1	U	K.AETEAGDVGPMVTESDIQHIVSSWTGIPVEK.V + Deamidated (NQ)
3702	599 - 606	501.7375	1001.4605	1001.4600	0.52	0	68	5.6e-07	1	U	K.MEDTLHTR.V
3115	607 - 615	479.7633	957.5121	957.5131	-1.07	0	14	0.085	1	U	R.VIGQDEAVK.A
16150	654 - 668	846.4097	1690.8049	1690.8024	1.44	0	45	6.1e-05	1	U	K.ALAAYYFGSEEAMIR.L
6599	669 - 677	579.2521	1156.4897	1156.4893	0.40	0	48	4.6e-05	1	U	R.LDMSEFMER.H
21634	683 - 704	755.7250	2264.1531	2264.1325	9.11	0	4	0.42	1	U	K.LIGSPPGYVGYTEGGQLTEAVR.R + Deamidated (NQ)
21635	683 - 704	1133.0841	2264.1537	2264.1325	9.36	0	21	0.011	1	U	K.LIGSPPGYVGYTEGGQLTEAVR.R + Deamidated (NQ)
21636	683 - 704	755.7255	2264.1547	2264.1325	9.84	0	13	0.056	1	U	K.LIGSPPGYVGYTEGGQLTEAVR.R + Deamidated (NQ)
22759	683 - 705	807.4230	2419.2471	2419.2496	-1.02	1	22	0.0092	1	U	K.LIGSPPGYVGYTEGGQLTEAVRR.R
17735	748 - 764	911.4852	1820.9559	1820.9554	0.33	0	81	1.9e-07	1	U	K.NTLLIMTSNVGSSVIEK.G + Oxidation (M)
19184	769 - 784	646.2908	1935.8505	1935.8486	0.96	1	47	3.7e-05	1	U	R.IGFDLDYDEKDSSYNR.I
10809	795 - 804	457.2387	1368.6943	1368.6938	0.36	0	10	0.1	1	U	K.QYFRPEFLNR.L
3996	805 - 812	511.7704	1021.5263	1021.5266	-0.29	0	39	0.00021	1	U	R.LDEMIVFR.Q
3997	805 - 812	511.7719	1021.5292	1021.5266	2.49	0	42	0.00018	1	U	R.LDEMIVFR.Q
4229	805 - 812	519.7690	1037.5234	1037.5216	1.79	0	31	0.0042	1	U	R.LDEMIVFR.Q + Oxidation (M)
2764	821 - 828	466.7619	931.5093	931.5048	4.78	0	4	0.63	1	U	K.EIADIMLK.E
14972	834 - 846	533.9720	1598.8941	1598.8991	-3.13	2	18	0.035	1	U	R.LKNKEIELQVTER.F
10615	836 - 846	679.8670	1357.7195	1357.7201	-0.48	1	39	0.0013	1	U	K.NKEIELQVTER.F
4195	872 - 880	518.2555	1034.4965	1034.4954	1.11	0	17	0.023	1	U	R.LLEDSMAEK.M

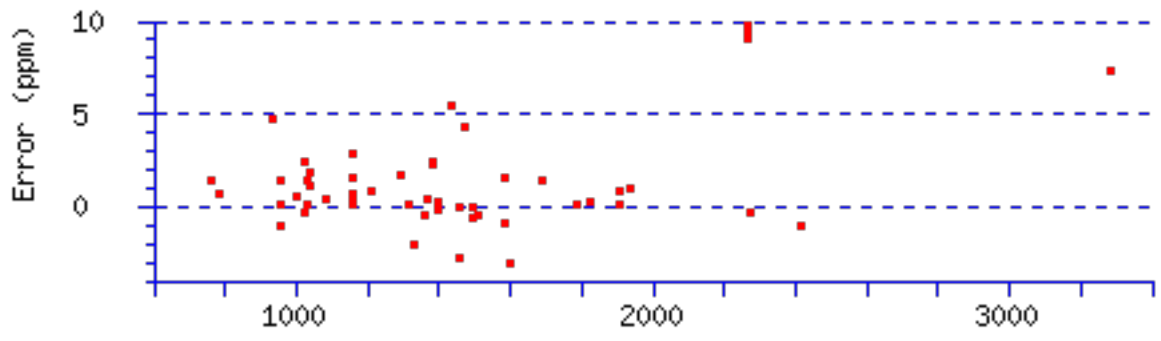

RMS error 3 ppm

MASCOT Search Results

Protein View: tr|A0A0K9Q7H2|A0A0K9Q7H2_SPIOL

>tr|A0A0K9Q7H2|A0A0K9Q7H2_SPIOL Uncharacterized protein OS=Spinacia oleracea OX=3562 GN=SOVF_216900 PE=4 SV=1

**Database:**	Uni-Spinach
**Score:**	1012
**Nominal mass (M_r):**	16238
**Calculated pI:**	9.73

Sequence similarity is available as an NCBI BLAST search of tr|A0A0K9Q7H2|A0A0K9Q7H2_SPIOL against nr.

Search parameters

**MS data file:**	\\172.16.0.213\tank\windowsVM\Bill Cramer\052418\MGF\wc_QE_052418_Cramer_gel_4.mgf
**Enzyme:**	Trypsin: cuts C-term side of KR unless next residue is P.
**Fixed modifications:**	Carbamidomethyl (C)
**Variable modifications:**	Acetyl (K), Acetyl (Protein N-term), Deamidated (NQ), Oxidation (M)

Protein sequence coverage: 55%

Matched peptides shown in ***bold red***.

```
  1 MTEAVIRNKP GMASVKDMPL LQDGPPPGGF APVRYARRIP TKGPSAIAIF
 51 LTTFGAFAWG MYQVGEGNKK RRVIKEEKYA ARRAILPLLQ AEEDERFVKE
101 WKKYLEDEAR IMKNVPGWKV GENVYNSGRW MPPATGELRP EVW
```

Unformatted sequence string: 143 residues (for pasting into other applications).

Sort peptides by ◉ Residue Number ○ Increasing Mass ○ Decreasing Mass

Show predicted peptides also

Query	Start – End	Observed	Mr(expt)	Mr(calc)	ppm	M	Score	Expect	Rank	U	Peptide
250	2 – 7	365.7085	729.4025	729.4021	0.60	0	41	0.0016	1	U	M.TEAVIR.N + Acetyl (Protein N-term)
18307	17 – 34	932.4734	1862.9323	1862.9349	-1.43	0	50	2.1e-05	1	U	K.DMPLLQDGPPPGGFAPVR.Y
18308	17 – 34	932.4741	1862.9336	1862.9349	-0.70	0	74	1.1e-07	1	U	K.DMPLLQDGPPPGGFAPVR.Y
18310	17 – 34	621.9855	1862.9348	1862.9349	-0.077	0	27	0.0027	1	U	K.DMPLLQDGPPPGGFAPVR.Y
18311	17 – 34	932.4750	1862.9354	1862.9349	0.28	0	44	8.2e-05	1	U	K.DMPLLQDGPPPGGFAPVR.Y
18312	17 – 34	932.4751	1862.9356	1862.9349	0.38	0	80	3.2e-08	1	U	K.DMPLLQDGPPPGGFAPVR.Y
18314	17 – 34	621.9865	1862.9377	1862.9349	1.48	0	65	7.9e-07	1	U	K.DMPLLQDGPPPGGFAPVR.Y
18315	17 – 34	621.9869	1862.9390	1862.9349	2.18	0	42	0.00011	1	U	K.DMPLLQDGPPPGGFAPVR.Y
18514	17 – 34	627.3145	1878.9217	1878.9298	-4.32	0	48	4.8e-05	1	U	K.DMPLLQDGPPPGGFAPVR.Y + Oxidation (M)
18517	17 – 34	940.4710	1878.9275	1878.9298	-1.26	0	86	6.4e-08	1	U	K.DMPLLQDGPPPGGFAPVR.Y + Oxidation (M)
18518	17 – 34	940.4731	1878.9316	1878.9298	0.92	0	55	4.9e-05	1	U	K.DMPLLQDGPPPGGFAPVR.Y + Oxidation (M)
18519	17 – 34	627.3182	1878.9327	1878.9298	1.50	0	39	0.00021	1	U	K.DMPLLQDGPPPGGFAPVR.Y + Oxidation (M)
18520	17 – 34	940.4739	1878.9332	1878.9298	1.79	0	87	7.6e-09	1	U	K.DMPLLQDGPPPGGFAPVR.Y + Oxidation (M)
15657	83 – 96	551.6372	1651.8899	1651.8893	0.34	1	26	0.0037	1	U	R.RAILPLLQAEEDER.F
15658	83 – 96	551.6382	1651.8927	1651.8893	2.05	1	34	0.00066	1	U	R.RAILPLLQAEEDER.F
19915	83 – 99	676.3818	2026.1235	2026.1211	1.19	2	32	0.00098	1	U	R.RAILPLLQAEEDERFVK.E
19916	83 – 99	507.5386	2026.1253	2026.1211	2.09	2	6	0.25	1	U	R.RAILPLLQAEEDERFVK.E
19917	83 – 99	676.3826	2026.1261	2026.1211	2.47	2	38	0.00027	1	U	R.RAILPLLQAEEDERFVK.E
13331	84 – 96	748.9022	1495.7898	1495.7882	1.07	0	67	4.7e-07	1	U	R.AILPLLQAEEDER.F
13332	84 – 96	748.9028	1495.7911	1495.7882	1.92	0	66	6.4e-07	1	U	R.AILPLLQAEEDER.F
18414	84 – 99	624.3482	1870.0229	1870.0200	1.57	1	42	0.00011	1	U	R.AILPLLQAEEDERFVK.E
18415	84 – 99	624.3489	1870.0250	1870.0200	2.68	1	30	0.0016	1	U	R.AILPLLQAEEDERFVK.E
4006	103 – 110	512.2585	1022.5025	1022.5032	-0.68	1	51	1.8e-05	1	U	K.KYLEDEAR.I
2246	104 – 110	448.2108	894.4071	894.4083	-1.34	0	31	0.0078	1	U	K.YLEDEAR.I
23	114 – 119	350.6924	699.3702	699.3704	-0.29	0	5	0.39	1	U	K.NVPGWK.V
24	114 – 119	350.6925	699.3704	699.3704	-0.044	0	6	0.8	1	U	K.NVPGWK.V

25	114 - 119	350.6927	699.3708	699.3704	0.54	0	12	0.079	1	U	K.NVPGWK.V
5382	120 - 129	547.7648	1093.5151	1093.5152	-0.16	0	53	1.1e-05	1	U	K.VGENVYNSGR.W
5383	120 - 129	547.7653	1093.5161	1093.5152	0.79	0	66	7e-07	1	U	K.VGENVYNSGR.W
5406	120 - 129	548.2565	1094.4984	1094.4992	-0.72	0	43	8.5e-05	1	U	K.VGENVYNSGR.W + Deamidated (NQ)
5407	120 - 129	548.2567	1094.4987	1094.4992	-0.44	0	32	0.001	1	U	K.VGENVYNSGR.W + Deamidated (NQ)
15882	130 - 143	834.9147	1667.8148	1667.8130	1.11	0	46	4.7e-05	1	U	R.WMPPATGELRPEVW.-
15883	130 - 143	556.9459	1667.8158	1667.8130	1.69	0	3	0.51	1	U	R.WMPPATGELRPEVW.-
15884	130 - 143	834.9156	1667.8166	1667.8130	2.19	0	32	0.00091	1	U	R.WMPPATGELRPEVW.-
15885	130 - 143	834.9156	1667.8167	1667.8130	2.24	0	36	0.00043	1	U	R.WMPPATGELRPEVW.-
15886	130 - 143	556.9462	1667.8169	1667.8130	2.33	0	7	0.22	1	U	R.WMPPATGELRPEVW.-
16088	130 - 143	842.9117	1683.8089	1683.8079	0.59	0	32	0.00097	1	U	R.WMPPATGELRPEVW.- + Oxidation (M)
16089	130 - 143	842.9118	1683.8091	1683.8079	0.72	0	36	0.00046	1	U	R.WMPPATGELRPEVW.- + Oxidation (M)

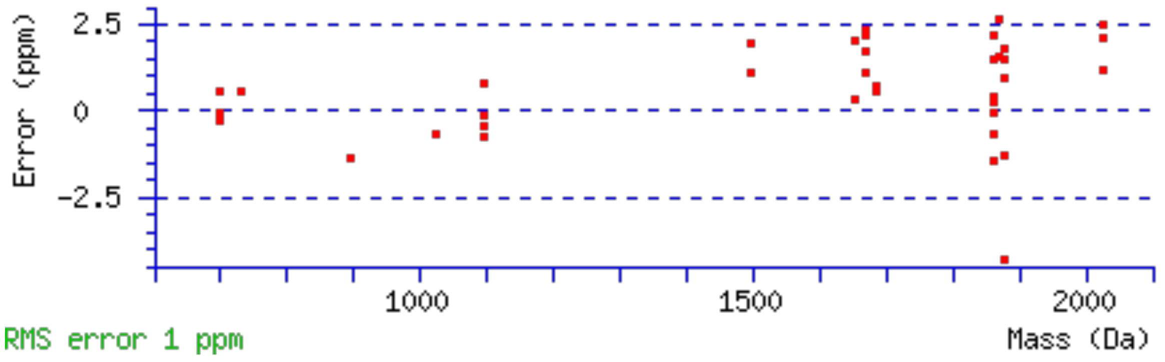

RMS error 1 ppm

Mascot: http://www.matrixscience.com/

MASCOT Search Results

Protein View: tr|A0A0K9RNJ7|A0A0K9RNJ7_SPIOL

>tr|A0A0K9RNJ7|A0A0K9RNJ7_SPIOL Uncharacterized protein OS=Spinacia oleracea OX=3562 GN=SOVF_052420 PE=4 SV=1

**Database:**	Uni-Spinach
**Score:**	1005
**Nominal mass (M_r):**	30114
**Calculated pI:**	5.99

Sequence similarity is available as **an NCBI BLAST search of tr|A0A0K9RNJ7|A0A0K9RNJ7_SPIOL against nr**.

Search parameters

**MS data file:**	\\172.16.0.213\tank\windowsVM\Bill Cramer\052418\MGF\wc_QE_052418_Cramer_gel_4.mgf
**Enzyme:**	Trypsin: cuts C-term side of KR unless next residue is P.
**Fixed modifications:**	Carbamidomethyl (C)
**Variable modifications:**	Acetyl (K), Acetyl (Protein N-term), Deamidated (NQ), Oxidation (M)

Protein sequence coverage: 58%

Matched peptides shown in ***bold red***.

```
  1 MGTLGRAIYR VGFWIRETGQ AMDRLGCRLQ GNYYFHEQLS RHRTLMNVFD
 51 KKPIVDKDAF VAPSASVIGD VQIGHGSSIW YGCVLRGDVN NISIGSGTNI
101 QDNSLVHVAK TNLAGKVLPT IIGDNVTVGH SAILHGCTVD DEAFVGMGAT
151 LLDGVVVEKH AMVAAGALVR QNTRIPSGQV WGGNPAKFLR NLTEEEKDFI
201 AVSASNYSNL AQVHAAENDK DFETIEYEKV LRKKYAHKDE EYDSMIGIVR
251 EIPPELVLPE NILPQNKDVQ KSS
```

Unformatted sequence string: **273 residues** (for pasting into other applications).

Sort peptides by ◉ Residue Number ○ Increasing Mass ○ Decreasing Mass

Show predicted peptides also

Query	Start – End	Observed	Mr(expt)	Mr(calc)	ppm	M	Score	Expect	Rank	U	Peptide
705	11 – 16	389.2245	776.4345	776.4334	1.51	0	22	0.0092	1		R.VGFWIR.E
706	11 – 16	389.2246	776.4346	776.4334	1.55	0	40	0.00098	1		R.VGFWIR.E
15835	11 – 24	555.9437	1664.8093	1664.8093	-0.016	1	14	0.047	1	U	R.VGFWIRETGQAMDR.L
15681	29 – 41	552.2700	1653.7880	1653.7899	-1.15	0	34	0.00072	1	U	R.LQGNYYFHEQLSR.H
15683	29 – 41	552.2709	1653.7910	1653.7899	0.64	0	35	0.00053	1	U	R.LQGNYYFHEQLSR.H
15684	29 – 41	827.9028	1653.7911	1653.7899	0.71	0	62	1.6e-06	1	U	R.LQGNYYFHEQLSR.H
15697	29 – 41	552.5996	1654.7771	1654.7739	1.88	0	5	0.33	1	U	R.LQGNYYFHEQLSR.H + Deamidated (NQ)
3265	44 – 51	484.2498	966.4851	966.4845	0.70	0	28	0.0025	1		R.TLMNVFDK.K
3266	44 – 51	484.2500	966.4855	966.4845	1.04	0	31	0.0013	1		R.TLMNVFDK.K
3477	44 – 51	492.2476	982.4806	982.4794	1.24	0	16	0.032	1		R.TLMNVFDK.K + Oxidation (M)
22885	87 – 110	818.0861	2451.2364	2451.2354	0.42	0	101	3.6e-10	1	U	R.GDVNNISIGSGTNIQDNSLVHVAK.T
22886	87 – 110	1226.6294	2451.2442	2451.2354	3.62	0	80	3.3e-08	1	U	R.GDVNNISIGSGTNIQDNSLVHVAK.T
22890	87 – 110	818.4146	2452.2221	2452.2194	1.10	0	74	1.3e-07	1	U	R.GDVNNISIGSGTNIQDNSLVHVAK.T + Deamidated (NQ)
22891	87 – 110	818.4155	2452.2246	2452.2194	2.11	0	48	3.3e-05	1	U	R.GDVNNISIGSGTNIQDNSLVHVAK.T + Deamidated (NQ)
22892	87 – 110	818.4209	2452.2409	2452.2194	8.76	0	54	8.8e-06	1	U	R.GDVNNISIGSGTNIQDNSLVHVAK.T + Deamidated (NQ)
5413	160 – 170	365.8750	1094.6032	1094.6019	1.18	0	32	0.00093	1	U	K.HAMVAAGALVR.Q
5414	160 – 170	365.8751	1094.6033	1094.6019	1.34	0	48	3.1e-05	1	U	K.HAMVAAGALVR.Q
5415	160 – 170	548.3092	1094.6038	1094.6019	1.74	0	26	0.01	1	U	K.HAMVAAGALVR.Q
5735	160 – 170	556.3061	1110.5976	1110.5968	0.77	0	23	0.0063	1	U	K.HAMVAAGALVR.Q + Oxidation (M)
9682	175 – 187	655.8429	1309.6712	1309.6779	-5.12	0	58	3.4e-06	1	U	R.IPSGQVWGGNPAK.F
9684	175 – 187	655.8459	1309.6772	1309.6779	-0.53	0	66	1.1e-06	1	U	R.IPSGQVWGGNPAK.F
9685	175 – 187	655.8477	1309.6808	1309.6779	2.19	0	39	0.00022	1	U	R.IPSGQVWGGNPAK.F
24970	191 – 229	893.4215	4462.0712	4462.0611	2.25	2	76	7.2e-08	1	U	R.NLTEEEKDFIAVSASNYSNLAQVHAAENDKDFETIEYEK.V + Deamidated (NQ)
24971	191 – 229	1116.7769	4463.0783	4463.0452	7.43	2	63	1.2e-06	1	U	R.NLTEEEKDFIAVSASNYSNLAQVHAAENDKDFETIEYEK.V + 2 Deamidated (NQ)
19080	235 – 250	963.4559	1924.8973	1924.8989	-0.83	1	72	6.6e-07	1	U	K.YAHKDEEYDSMIGIVR.E
19081	235 – 250	642.6404	1924.8993	1924.8989	0.23	1	58	3.8e-06	1	U	K.YAHKDEEYDSMIGIVR.E
19082	235 – 250	642.6404	1924.8995	1924.8989	0.32	1	14	0.051	1	U	K.YAHKDEEYDSMIGIVR.E
19083	235 – 250	482.2325	1924.9008	1924.8989	1.01	1	34	0.00062	1	U	K.YAHKDEEYDSMIGIVR.E
19238	235 – 250	647.9718	1940.8936	1940.8938	-0.11	1	55	7.7e-06	1	U	K.YAHKDEEYDSMIGIVR.E + Oxidation (M)
19239	235 – 250	971.4553	1940.8961	1940.8938	1.18	1	36	0.00042	1	U	K.YAHKDEEYDSMIGIVR.E + Oxidation (M)
19261	251 – 267	972.0455	1942.0764	1942.0775	-0.54	0	37	0.00036	1	U	R.EIPPELVLPENILPQNK.D
19262	251 – 267	648.3669	1942.0790	1942.0775	0.79	0	30	0.0016	1	U	R.EIPPELVLPENILPQNK.D
19263	251 – 267	972.0468	1942.0791	1942.0775	0.83	0	50	2e-05	1	U	R.EIPPELVLPENILPQNK.D
19264	251 – 267	648.3677	1942.0811	1942.0775	1.89	0	26	0.0038	1	U	R.EIPPELVLPENILPQNK.D
22710	251 – 271	805.1166	2412.3281	2412.3264	0.70	1	45	6.1e-05	1	U	R.EIPPELVLPENILPQNKDVQK.S
22711	251 – 271	805.1167	2412.3284	2412.3264	0.82	1	15	0.039	1	U	R.EIPPELVLPENILPQNKDVQK.S

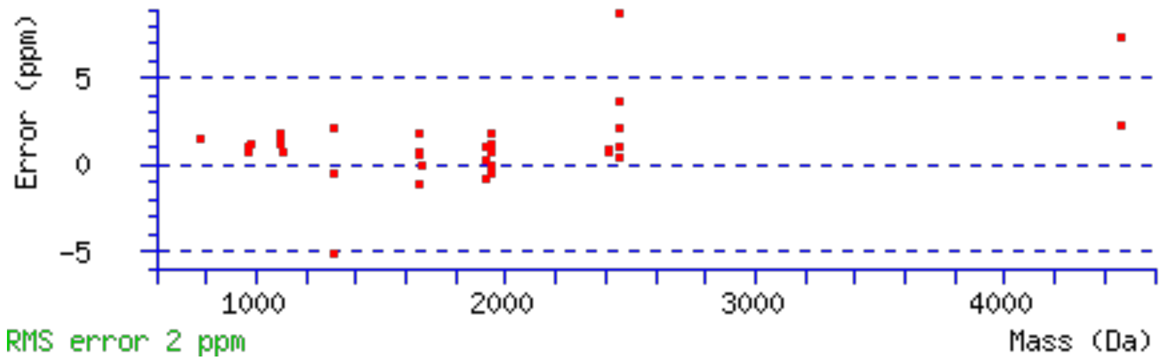

Mascot: http://www.matrixscience.com/

MASCOT Search Results

Protein View: tr|A0A0K9R772|A0A0K9R772_SPIOL

>tr|A0A0K9R772|A0A0K9R772_SPIOL Chlorophyll a-b binding protein, chloroplastic OS=Spinacia oleracea OX=3562 GN=SOVF_099030 PE=3 SV=1

**Database:**	Uni-Spinach
**Score:**	912
**Nominal mass (M_r):**	27852
**Calculated pI:**	6.10

Sequence similarity is available as **an NCBI BLAST search of tr|A0A0K9R772|A0A0K9R772_SPIOL against nr**.

Search parameters

**MS data file:**	\\172.16.0.213\tank\windowsVM\Bill Cramer\052418\MGF\wc_QE_052418_Cramer_gel_4.mgf
**Enzyme:**	Trypsin: cuts C-term side of KR unless next residue is P.
**Fixed modifications:**	Carbamidomethyl (C)
**Variable modifications:**	Acetyl (K), Acetyl (Protein N-term), Deamidated (NQ), Oxidation (M)

Protein sequence coverage: 59%

Matched peptides shown in ***bold red***.

```
  1 MAAVTTQASI AGFRPCASKP RFLTGVPGKL NKESSGVRLP STSSTTSFKV
 51 EAKKGEWLPG LASPGYLTGS LPGDNGFDPL ALAEDPENLR WFVQAELVNG
101 RWAMLGVAGM LLPEVFTSIG IIDVPKWYDA GKSEYFASSS TLFVIEFILF
151 HYVEIRRWQD IKNPGCVNQD PIFKQYSLPP NECGYPGGIF NPLNFAPTTE
201 AKEKELANGR LAMLAFLGFI VQHNVTGKGP FDNLQQHLSD PWHNTIIQTF
251 GGN
```

Unformatted sequence string: **253 residues** (for pasting into other applications).

Sort peptides by ◉ Residue Number ○ Increasing Mass ○ Decreasing Mass

Show predicted peptides also

Query	Start – End	Observed	Mr(expt)	Mr(calc)	ppm	M	Score	Expect	Rank	U	Peptide
24866	54 – 90	1290.3110	3867.9113	3867.8843	6.98	1	6	0.28	1	U	K.KGEWLPGLASPGYLTGSLPGDNGFDPLALAEDPENLR.W + 2 Deamidated (NQ)
24835	55 – 90	1246.9498	3737.8275	3737.8213	1.66	0	67	5.5e-07	1	U	K.GEWLPGLASPGYLTGSLPGDNGFDPLALAEDPENLR.W
9856	91 – 101	659.8489	1317.6832	1317.6830	0.17	0	52	1.3e-05	1	U	R.WFVQAELVNGR.W
9858	91 – 101	659.8492	1317.6838	1317.6830	0.65	0	52	1.5e-05	1	U	R.WFVQAELVNGR.W
9859	91 – 101	440.2352	1317.6838	1317.6830	0.65	0	30	0.0047	1	U	R.WFVQAELVNGR.W
9860	91 – 101	659.8495	1317.6844	1317.6830	1.08	0	52	1.4e-05	1	U	R.WFVQAELVNGR.W
9874	91 – 101	440.5622	1318.6648	1318.6670	-1.68	0	18	0.052	1	U	R.WFVQAELVNGR.W + Deamidated (NQ)
9875	91 – 101	660.3405	1318.6665	1318.6670	-0.37	0	14	0.046	1	U	R.WFVQAELVNGR.W + Deamidated (NQ)
9876	91 – 101	660.3405	1318.6665	1318.6670	-0.36	0	74	1.2e-07	1	U	R.WFVQAELVNGR.W + Deamidated (NQ)
9877	91 – 101	660.3407	1318.6669	1318.6670	-0.061	0	73	1.4e-07	1	U	R.WFVQAELVNGR.W + Deamidated (NQ)
9878	91 – 101	660.3410	1318.6674	1318.6670	0.34	0	74	1.3e-07	1	U	R.WFVQAELVNGR.W + Deamidated (NQ)
9879	91 – 101	440.5632	1318.6678	1318.6670	0.61	0	18	0.022	1	U	R.WFVQAELVNGR.W + Deamidated (NQ)
9880	91 – 101	660.3412	1318.6679	1318.6670	0.69	0	37	0.00031	1	U	R.WFVQAELVNGR.W + Deamidated (NQ)
9881	91 – 101	660.3415	1318.6684	1318.6670	1.11	0	74	1.3e-07	1	U	R.WFVQAELVNGR.W + Deamidated (NQ)
9882	91 – 101	660.3419	1318.6692	1318.6670	1.66	0	69	3.2e-07	1	U	R.WFVQAELVNGR.W + Deamidated (NQ)
9883	91 – 101	660.3422	1318.6699	1318.6670	2.23	0	69	3.4e-07	1	U	R.WFVQAELVNGR.W + Deamidated (NQ)
9884	91 – 101	440.5640	1318.6702	1318.6670	2.42	0	17	0.028	1	U	R.WFVQAELVNGR.W + Deamidated (NQ)
9885	91 – 101	660.3430	1318.6715	1318.6670	3.44	0	51	1.5e-05	1	U	R.WFVQAELVNGR.W + Deamidated (NQ)
317	127 – 132	370.1742	738.3339	738.3337	0.26	0	14	0.048	1	U	K.WYDAGK.S
318	127 – 132	370.1745	738.3344	738.3337	0.98	0	23	0.007	1	U	K.WYDAGK.S
1610	157 – 162	423.2351	844.4556	844.4555	0.14	1	10	0.28	1	U	R.RWQDIK.N
21220	157 – 174	739.3741	2215.1006	2215.0844	7.31	2	20	0.013	1	U	R.RWQDIKNPGCVNQDPIFK.Q + Deamidated (NQ)
20128	158 – 174	1030.0079	2058.0012	2057.9993	0.93	1	54	7.9e-06	1	U	R.WQDIKNPGCVNQDPIFK.Q
20129	158 – 174	687.0081	2058.0023	2057.9993	1.47	1	52	1.5e-05	1	U	R.WQDIKNPGCVNQDPIFK.Q
20130	158 – 174	687.0081	2058.0025	2057.9993	1.57	1	7	0.23	1	U	R.WQDIKNPGCVNQDPIFK.Q
20135	158 – 174	687.3356	2058.9849	2058.9833	0.77	1	23	0.0066	1	U	R.WQDIKNPGCVNQDPIFK.Q + Deamidated (NQ)
20136	158 – 174	687.3360	2058.9861	2058.9833	1.36	1	6	0.29	1	U	R.WQDIKNPGCVNQDPIFK.Q + Deamidated (NQ)
11163	163 – 174	694.8346	1387.6546	1387.6554	-0.64	0	37	0.00035	1	U	K.NPGCVNQDPIFK.Q
11164	163 – 174	694.8355	1387.6564	1387.6554	0.67	0	59	2.9e-06	1	U	K.NPGCVNQDPIFK.Q
11177	163 – 174	695.3261	1388.6376	1388.6394	-1.36	0	10	0.1	1	U	K.NPGCVNQDPIFK.Q + Deamidated (NQ)
24374	175 – 202	1028.1595	3081.4566	3081.4542	0.78	0	45	6.5e-05	1	U	K.QYSLPPNECGYPGGIFNPLNFAPTTEAK.E
2576	203 – 210	458.7460	915.4774	915.4774	0.046	1	13	0.38	1	U	K.EKELANGR.L
2585	203 – 210	459.2378	916.4610	916.4614	-0.35	1	1	0.89	1	U	K.EKELANGR.L + Deamidated (NQ)
2586	203 – 210	459.2381	916.4616	916.4614	0.22	1	27	0.0093	1	U	K.EKELANGR.L + Deamidated (NQ)
19574	211 – 228	988.0454	1974.0763	1974.0761	0.12	0	72	1.8e-07	1	U	R.LAMLAFLGFIVQHNVTGK.G + Oxidation (M)
19575	211 – 228	659.0342	1974.0808	1974.0761	2.37	0	65	7.5e-07	1	U	R.LAMLAFLGFIVQHNVTGK.G + Oxidation (M)
24058	229 – 253	946.1176	2835.3310	2835.3365	-1.93	0	21	0.01	1	U	K.GPFDNLQQHLSDPWHNTIIQTFGGN.-
24059	229 – 253	946.1200	2835.3383	2835.3365	0.62	0	35	0.0005	1	U	K.GPFDNLQQHLSDPWHNTIIQTFGGN.-

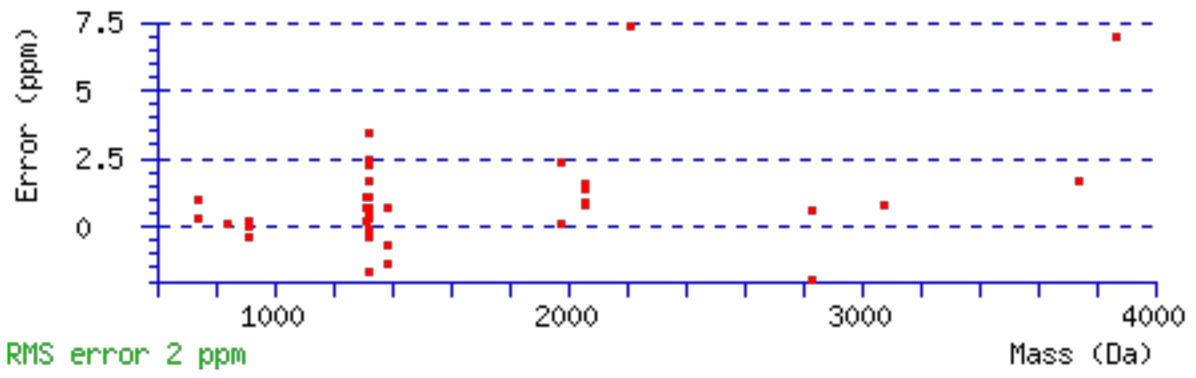

Mascot: http://www.matrixscience.com/

MASCOT Search Results

Protein View: tr|A0A0K9RRT4|A0A0K9RRT4_SPIOL

>tr|A0A0K9RRT4|A0A0K9RRT4_SPIOL Chlorophyll a-b binding protein, chloroplastic OS=Spinacia oleracea OX=3562 GN=SOVF_041770 PE=3 SV=1

**Database:**	Uni-Spinach
**Score:**	880
**Nominal mass (M_r):**	26370
**Calculated pI:**	5.97

Sequence similarity is available as an NCBI BLAST search of tr|A0A0K9RRT4|A0A0K9RRT4_SPIOL against nr.

Search parameters

**MS data file:**	\\172.16.0.213\tank\windowsVM\Bill Cramer\052418\MGF\wc_QE_052418_Cramer_gel_4.mgf
**Enzyme:**	Trypsin: cuts C-term side of KR unless next residue is P.
**Fixed modifications:**	Carbamidomethyl (C)
**Variable modifications:**	Acetyl (K), Acetyl (Protein N-term), Deamidated (NQ), Oxidation (M)

Protein sequence coverage: 17%

Matched peptides shown in ***bold red***.

```
  1 MASNALMSCG IAAVFPSSLS SSKSKFAASV PLGNVSCNAS SRFTMSAEWM
 51 PGQPRPAHLD GSAPGDFGFD PLGLGEVPEN LERFKESELI HCRWAMLAVP
101 GILVPEALGL GNWVKAQEWA ALPGGQATYL GNPVPWGNLP TILAIEFLAI
151 AFVEHQRSME KDSEKKKYPG GAFDPLGYSK DPKKFEELKL KEIKNGRLAL
201 LAFVGFCIQQ SAYPGTGPLE NLATHLADPW HNNIGDIVIP RAL
```

Unformatted sequence string: 243 residues (for pasting into other applications).

Sort peptides by ◉ Residue Number ○ Increasing Mass ○ Decreasing Mass

Show predicted peptides also

Query	Start – End	Observed	Mr(expt)	Mr(calc)	ppm	M	Score	Expect	Rank	U	Peptide
9846	84 – 93	659.8319	1317.6492	1317.6499	-0.54	1	58	2e-05	1	U	R.FKESELIHCR.W
9847	84 – 93	440.2238	1317.6497	1317.6499	-0.19	1	22	0.0093	1	U	R.FKESELIHCR.W
9848	84 – 93	440.2246	1317.6521	1317.6499	1.62	1	60	9e-06	1	U	R.FKESELIHCR.W
15317	166 – 180	814.4258	1626.8370	1626.8406	-2.20	2	65	8.6e-07	1	U	K.KKYPGGAFDPLGYSK.D
15320	166 – 180	814.4271	1626.8396	1626.8406	-0.62	2	64	9.6e-07	1	U	K.KKYPGGAFDPLGYSK.D
15321	166 – 180	814.4275	1626.8405	1626.8406	-0.061	2	62	1.6e-06	1	U	K.KKYPGGAFDPLGYSK.D
15322	166 – 180	543.2876	1626.8409	1626.8406	0.17	2	14	0.049	1	U	K.KKYPGGAFDPLGYSK.D
15323	166 – 180	543.2877	1626.8414	1626.8406	0.48	2	45	5.8e-05	1	U	K.KKYPGGAFDPLGYSK.D
15324	166 – 180	407.7177	1626.8418	1626.8406	0.75	2	43	8.7e-05	1	U	K.KKYPGGAFDPLGYSK.D
15325	166 – 180	407.7178	1626.8419	1626.8406	0.84	2	37	0.00034	1	U	K.KKYPGGAFDPLGYSK.D
15328	166 – 180	407.7180	1626.8429	1626.8406	1.42	2	8	0.19	1	U	K.KKYPGGAFDPLGYSK.D
15329	166 – 180	543.2887	1626.8442	1626.8406	2.26	2	61	1.8e-06	1	U	K.KKYPGGAFDPLGYSK.D
13376	167 – 180	750.3797	1498.7449	1498.7456	-0.50	1	81	2.6e-08	1	U	K.KYPGGAFDPLGYSK.D
13377	167 – 180	500.5891	1498.7455	1498.7456	-0.069	1	27	0.0028	1	U	K.KYPGGAFDPLGYSK.D
13378	167 – 180	750.3801	1498.7457	1498.7456	0.082	1	69	7.3e-07	1	U	K.KYPGGAFDPLGYSK.D
13379	167 – 180	500.5893	1498.7459	1498.7456	0.20	1	27	0.003	1	U	K.KYPGGAFDPLGYSK.D
13380	167 – 180	750.3806	1498.7466	1498.7456	0.67	1	49	2.4e-05	1	U	K.KYPGGAFDPLGYSK.D
13381	167 – 180	500.5896	1498.7470	1498.7456	0.91	1	33	0.00086	1	U	K.KYPGGAFDPLGYSK.D
13382	167 – 180	500.5900	1498.7481	1498.7456	1.63	1	6	0.25	1	U	K.KYPGGAFDPLGYSK.D
13384	167 – 180	500.5907	1498.7502	1498.7456	3.07	1	38	0.00025	1	U	K.KYPGGAFDPLGYSK.D
17999	167 – 183	920.4680	1838.9214	1838.9203	0.60	2	59	3.3e-06	1	U	K.KYPGGAFDPLGYSKDPK.K
18000	167 – 183	613.9811	1838.9216	1838.9203	0.70	2	16	0.033	1	U	K.KYPGGAFDPLGYSKDPK.K
18001	167 – 183	613.9811	1838.9216	1838.9203	0.72	2	6	0.26	1	U	K.KYPGGAFDPLGYSKDPK.K
18002	167 – 183	613.9811	1838.9216	1838.9203	0.72	2	25	0.0065	1	U	K.KYPGGAFDPLGYSKDPK.K
18003	167 – 183	920.4685	1838.9224	1838.9203	1.15	2	54	8.7e-06	1	U	K.KYPGGAFDPLGYSKDPK.K
18004	167 – 183	460.7379	1838.9225	1838.9203	1.21	2	18	0.021	1	U	K.KYPGGAFDPLGYSKDPK.K
18008	167 – 183	460.7382	1838.9239	1838.9203	1.96	2	14	0.18	1	U	K.KYPGGAFDPLGYSKDPK.K
10837	168 – 180	686.3328	1370.6510	1370.6507	0.26	0	70	2.6e-07	1	U	K.YPGGAFDPLGYSK.D

10838	168 – 180	686.3332	1370.6517	1370.6507	0.79	0	80	3e-08	1	U	K.YPGGAFDPLGYSK.D
10839	168 – 180	686.3342	1370.6538	1370.6507	2.28	0	37	0.00031	1	U	K.YPGGAFDPLGYSK.D
16379	168 – 183	856.4205	1710.8265	1710.8253	0.69	1	55	6.5e-06	1	U	K.YPGGAFDPLGYSKDPK.K
16380	168 – 183	856.4211	1710.8277	1710.8253	1.40	1	27	0.0032	1	U	K.YPGGAFDPLGYSKDPK.K
16383	168 – 183	571.2848	1710.8327	1710.8253	4.29	1	18	0.023	1	U	K.YPGGAFDPLGYSKDPK.K
18005	168 – 184	460.7379	1838.9226	1838.9203	1.28	2	2	0.59	1	U	K.YPGGAFDPLGYSKDPKK.F
875	184 – 189	397.2262	792.4378	792.4381	-0.39	1	2	0.68	1	U	K.KFEELK.L
878	184 – 189	397.2265	792.4384	792.4381	0.34	1	19	0.092	1	U	K.KFEELK.L
879	184 – 189	397.2267	792.4388	792.4381	0.88	1	22	0.11	1	U	K.KFEELK.L
880	184 – 189	397.2268	792.4390	792.4381	1.13	1	3	0.57	1	U	K.KFEELK.L
881	184 – 189	397.2268	792.4391	792.4381	1.21	1	13	0.15	1	U	K.KFEELK.L
3105	190 – 197	479.2947	956.5749	956.5767	-1.88	2	11	0.55	1		K.LKEIKNGR.L
3126	190 – 197	479.7874	957.5603	957.5607	-0.45	2	13	1	2		K.LKEIKNGR.L + Deamidated (NQ)
3127	190 – 197	479.7877	957.5609	957.5607	0.27	2	40	0.0019	1		K.LKEIKNGR.L + Deamidated (NQ)

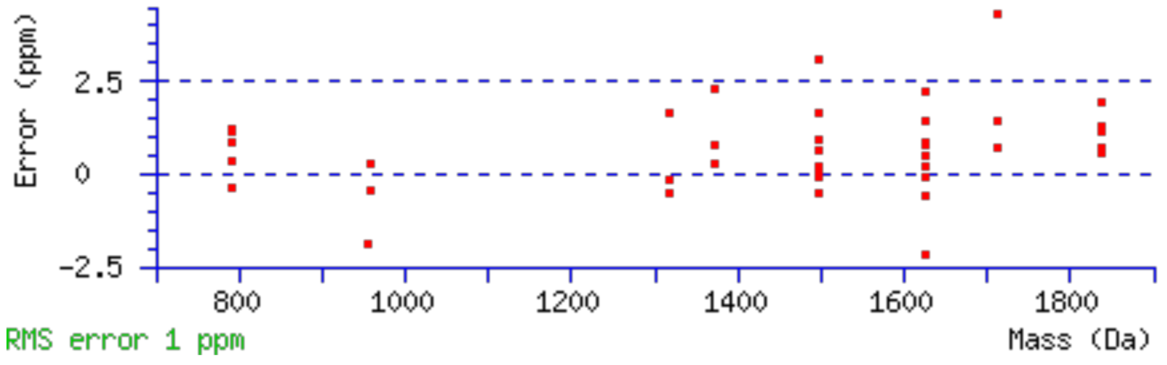

Mascot: http://www.matrixscience.com/
