# Supplementary material for: A novel chloroplast super-complex consisting of the ATP synthase and photosystem I reaction center
Source: PLoS One. 2020 Aug 20;15(8):e0237569. doi: 10.1371/journal.pone.0237569 (PMC7444523; doi:10.1371/journal.pone.0237569)
Supplement: S1 Raw images — (PDF) [file pone.0237569.s002.pdf]

Fig. 2B: Clear Native PAGE showing the high molecular weight super-complex band isolated from the sucrose density gradient, running at ~1.1 MDa.

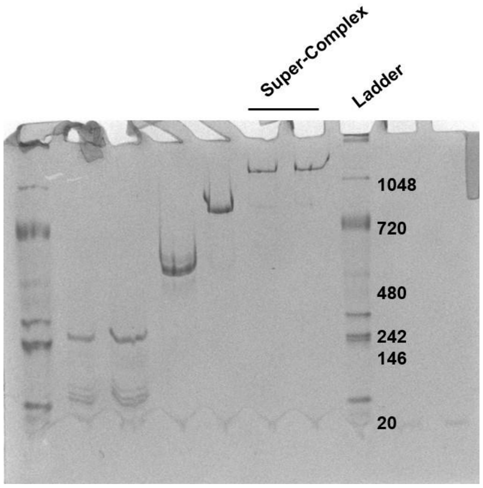

Fig. 2C: Silver-stained 2D SDS PAGE showing the individual components of the high molecular weight super-complex fraction obtained from the sucrose density gradient

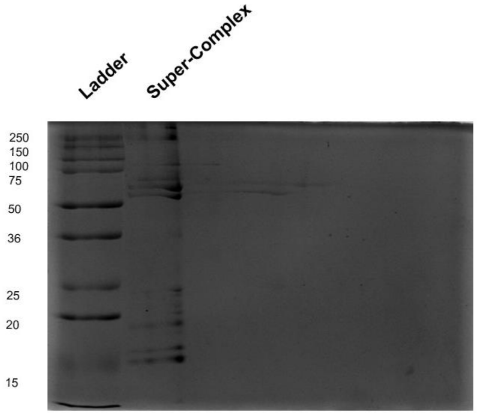

Fig. 2D: Western Blot for ATP synthase using anti-ATPa antibody (PhytoAB).

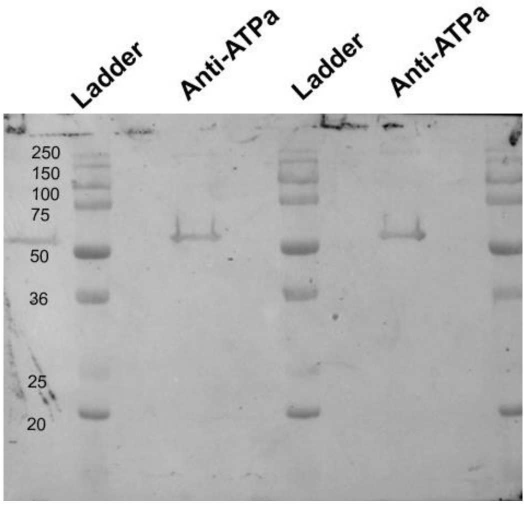

Fig.2E: Western Blot for PSI using anti-PsaA antibody (PhytoAB).

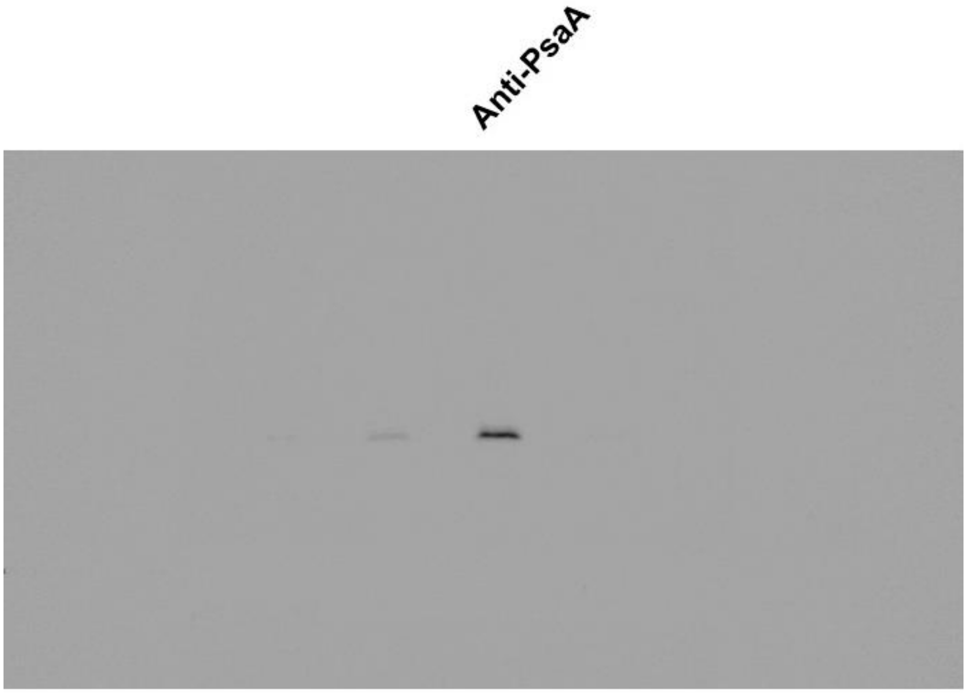

Fig. 2F: Western Blot for FNR using anti-FNR antibody (PhytoAB)

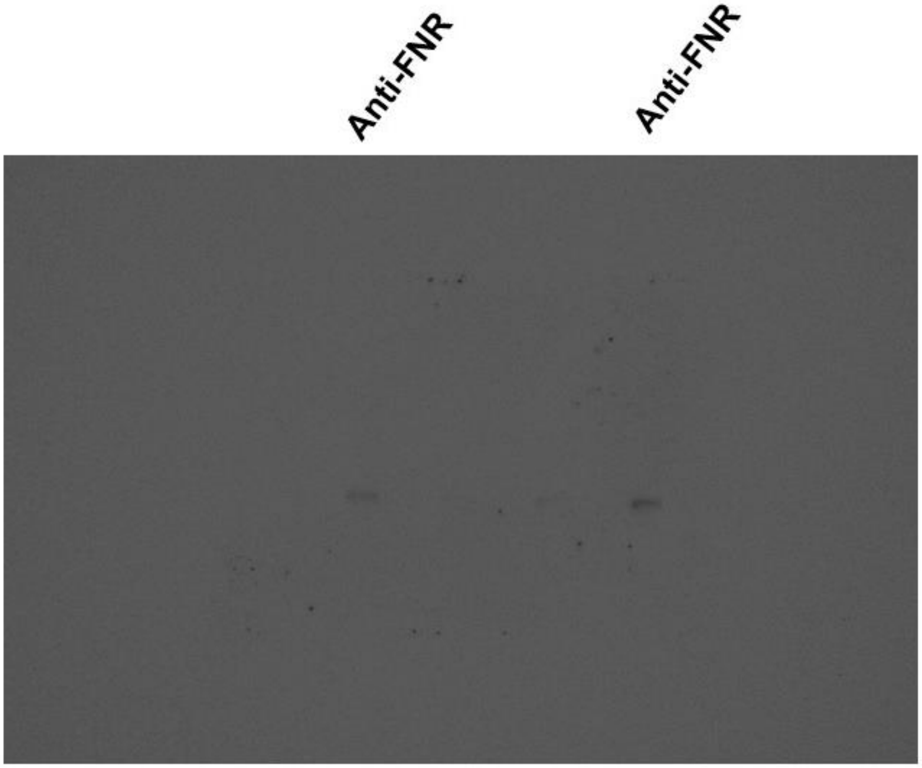
Anti-FNR
Anti-FNR
